# Supplementary material for: The Osteoarthritis Thumb Therapy (OTTER) II Trial: a study protocol for a three-arm multi-centre randomised placebo controlled trial of the clinical effectiveness and efficacy and cost-effectiveness of splints for symptomatic thumb base osteoarthritis
Source: BMJ Open. 2019 Oct 22;9(10):e028342. doi: 10.1136/bmjopen-2018-028342 (PMC6830636; doi:10.1136/bmjopen-2018-028342)
Supplement: Supplementary data [file bmjopen-2018-028342supp002.pdf]

Appendix 1

NHS Recruitment Sites

Pulvertaft Hand Centre, Royal Derby Hospital, Derby Teaching Hospitals NHS Foundation Trust, Derby, UK

Hand Therapy Department, Dorset County Hospital, Dorset County Hospital NHS Foundation Trust, Dorchester, UK

Hand Therapy Unit, Poole Hospital, Poole Hospital NHS Foundation Trust, Poole, UK

Plastic Surgery Rehabilitation, Royal Devon and Exeter Hospital, Royal Devon and Exeter NHS Foundation Trust, Exeter, UK

Occupational Therapy Department, North Devon District Hospital, North Devon Healthcare NHS Trust, Barnstaple, UK

Rheumatology Therapy Department, Royal National Hospital for Rheumatic Diseases, Royal United Hospitals Bath NHS Foundation Trust, Bath, UK

Therapies Department, Royal United Hospitals, Royal United Hospitals Bath NHS Foundation Trust, Bath, UK

Occupational Therapy Department, Haywood Hospital, Midlands Partnership NHS Foundation Trust, Stoke-on-Trent, UK

Pennine MSK Partnership Ltd., Integrated Care Centre, Oldham, UK

Clinical Therapies Department, Bassetlaw Hospital, Doncaster and Bassetlaw Teaching Hospitals NHS Foundation Trust, Worksop, UK

Bassetlaw Locality Retford Primary Care Centre, Doncaster and Bassetlaw Teaching Hospitals NHS Foundation Trust, Retford, UK

Therapy Department, Yeovil Hospital, Yeovil District Hospital NHS Foundation Trust, Yeovil, UK

Hand Therapy, Basingstoke and North Hampshire Hospital, Hampshire Hospitals NHS Foundation Trust, Basingstoke, UK

Therapy Department, Royal Hampshire County Hospital, Hampshire Hospitals NHS Foundation Trust, Winchester, UK

Occupational Therapy Department, Queen Alexandra Hospital, Portsmouth Hospitals NHS Trust, Cosham, UK

Hand Therapy Unit, The Robert Jones and Agnes Hunt Orthopaedic Hospital, The Robert Jones and Agnes Hunt Orthopaedic Hospital NHS Foundation Trust, Oswestry, UK

Hand Therapy, The Royal Free Hospital, Royal Free London NHS Foundation Trust, London, UK

Appendix 2

Self-Management Package:

Hand Exercise Booklet *(see separate supplementary pdf file as too large a file to include)*

Appendix 2

Self-Management Package: Joint Protection Booklet

Osteoarthritis Thumb Base Therapy Trial

OTTER trial

Osteoarthritis
Joint Protection Booklet

Information and advice about reducing pain and protecting your joints

OTTER II trial – REC ref: 16/SC/0188

Osteoarthritis

You have been given this booklet because you have pain or discomfort in one or more of your joints.

Osteoarthritis is a common cause of joint pain effecting 20% of the general population aged over 55 years.

Osteoarthritis can affect many aspects of your everyday life. This booklet has been designed with the help of clinicians in response to questions asked by patients with Osteoarthritis including advice about how to carry on with your normal life as much as possible whilst reducing pain and protecting your joints from further damage.

This booklet contains information on the following:

1. What is Osteoarthritis?
2. Protecting your joints
3. Dealing with fatigue
4. Diet
5. Holistic Medicine

1. What is Osteoarthritis?

A 'normal' joint is where two or more bones meet. The joint allows the bones to move freely but within limits.

Osteoarthritis is a disease that affects the body's joints. The surfaces within the joint are damaged so the joint does not move as smoothly as it should. The main symptoms are pain and sometimes stiffness.

When a joint develops Osteoarthritis, some of the cartilage covering the ends of the bones gradually roughens and becomes thin and the bone underneath thickens. All the tissues within the joint become more active than normal, as if the body is trying to repair the damage.

Sometimes the body's repairs are quite successful and the changes inside the joint will not cause much pain. If the Osteoarthritis becomes severe, the cartilage can become so thin that it no longer covers the ends of the bones. The bones start to rub against each other and eventually can start to wear away. The loss of cartilage, the wearing of bone and sometimes bony spurs can alter the shape of the joint, forcing the bones out of their normal alignment.

2. Protecting your joints

Joint protection can reduce joint damage, preserve range of motion, and lessen Osteoarthritis pain by reducing the general stress and strain on the joint. Making changes early can help avoid joint problems becoming worse in the future.

Become more aware of how you use the joints that ache, both at home and at work. For example, try watching your actions while you use your hands – for example when you make a hot drink:

- What is happening to your fingers while you are turning the tap? Are they being pushed towards the little finger?
- What happens to your thumbs as you take the lid of the coffee jar? Is there pressure or aching at the base of your thumb?
- What is happening to your wrist and fingers as you lift the kettle? Can you feel aching or pulling at these joints?

You might already have tried picking up the kettle with two hands when your hands are painful, but it is important to do this **all the time**, not just when your hands are hurting. This is an example of joint protection.

The following principles may help you:

- **Respect pain.** If you are experiencing pain after an activity, you must consider that you have been too active or have done too much.
- **Pace your activities throughout the day.** Spread physically hard jobs, such as housework or mowing the lawn, at intervals through the day, rather than tackling them all at once.
- **Avoid any activity that causes pain and find a better way of accomplishing the task.** Avoid a tight grip that strains joints and muscles. Avoid a prolonged or continuous grip. Small joints can take little weight, so gripping a pen or cutlery can be hard work. **Holding a larger object involves more joints, so it spreads the grip.** Picking up an object with two hands halves the load on each hand.

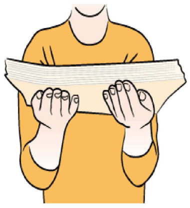

- **Make handles larger with padding,** for example, sponge tubing for pipe lagging. A solid surface is harder to grip than a soft surface, also a larger surface area means less strain. Wear padded gloves when gardening.
- **Use adaptive devices**. There are many devices available on the market to make it easier for you to carry out everyday tasks. Below are examples of some which may make your life easier and reduce the stress on your thumb joint:
    - **Spring loaded scissors** reduce the load on your thumb.

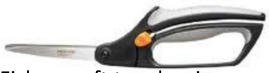

Fiskars soft touch scissors

    - **Jar Keys** can really make a difference as they break the vacuum when opening jars.

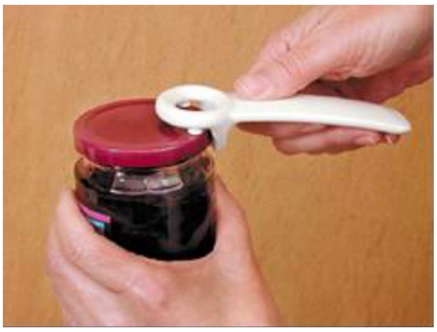

– **L-shaped knifes** do not use the thumb at all.

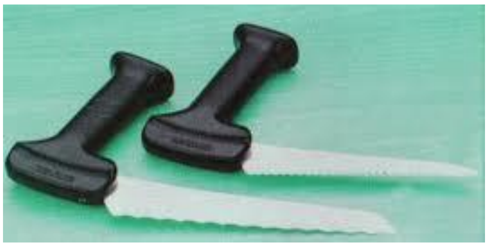

– **Ring pull can openers** like a 'magi pull' available from Lakeland can make opening cans much easier.

– As we age the natural stickiness of our hands decrease and are hands become dryer, this can lead us to have to use 20% more grip strength to open jars, etc. Using a **damp dish cloth** can reduce the amount of grip strength you need to use.

– When straining vegetables, instead of lifting and tipping the pan, **place vegetables in a wire basket in the pan to cook**. When ready they can be lifted and drained in the basket. Leave the saucepan to cool before moving it.

– When using the kettle, **use a plastic jug to fill the kettle from the tap** and only fill the kettle up as required. A **kettle tipper can be used**, or a small lightweight travel kettle. Also, kettles with the handle over the top have been reported to be easier to lift using two hands rather than the kettles with the handle on the side.

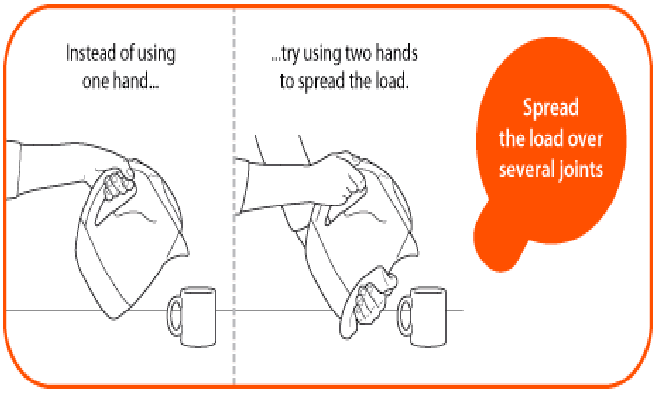

3. Dealing with fatigue

At times people with arthritis may experience fatigue. Fatigue is a feeling of weariness, but it is more extreme than simple tiredness. It can affect you physically, making your limbs seem heavy and causing you to feel exhausted, but it can also affect your concentration and

motivation. People who experience fatigue may find they struggle to do even small tasks. It often comes on for no apparent reason and without warning. There are many things that can cause fatigue including those listed below:

- Anaemia, which often accompanies inflammation.
- Other long term conditions such as diabetes or thyroid disease.
- Some drugs used to treat arthritis which may cause drowsiness or loss of concentration.
- Pain, especially if it is long term weak muscles, which mean you have to use more energy to do everyday tasks.
- Overdoing things or carrying on with activities for too long.
- Sleep disturbances as a result of pain, late nights or sleeping too much in the day.
- Stress and anxiety.
- Low mood or depression.
- Poor diet or hunger.

What can you do to help yourself?

- **Talk to your GP** or Rheumatology team about getting support or a **review of your medication**.
- Use the **four 'Ps'** – **P**roblem Solving, **P**lanning, **P**rioritising and **P**acing.
- **Tell family, friends and colleagues** about your fatigue so that they can understand and help if needed.
- Gradually **increase your physical exercise**. This will improve your general well-being, strength and energy levels.
- **Deal with stress or anxiety**.
- **Talk to your GP** if you have low mood.
- Improve your **sleeping habits**.
- Eat a **healthy diet**.

4. Diet

Whilst it is not the cause of Osteoarthritis and thumb base pain, being overweight can affect your health in many ways. If you are overweight then losing weight will help reduce the strain on your joints, increase your sense of well-being and reduce your risk of other health problems.

Apart from **reducing sugar** and **fat**, **taking regular exercise,** and ensuring that you **have your five a day of fruit and vegetables**, it has been shown that **increasing** your **intake** of particular vitamins and minerals, particularly **Calcium, Vitamin D** and **Iron**, **eating less saturated fat** and incorporating more **good fats** in your diet such as **Omega 3** can improve symptoms of Arthritis. See table below for a list of good and bad fats.

Saturated Fats<br>(Bad Fats)<br>**Avoid**	**Polyunsaturated Fats<br>(Fats that can increase inflammation)<br>Avoid**	**Monounsaturated Fats<br>(Neutral fats but contain a high amount of calories)<br>Limit your intake**
• Full fat dairy products<br>• Processed foods (for example, cakes, biscuits and pastry)<br>• Chips, if fried in animal fat<br>• Foods cooked using ghee (clarified butter)	• Softer Fats and oils<br>• Corn or sunflower sources of oil	• Olive oil<br>• Rapeseed oil

Omega-3 Polyunsaturated Fatty Acids

These are important to keep in your diet. **Omega-3** has been found to be of benefit to people with Arthritis. It can be found in free range eggs, oily fish and fish oil supplements. The table below shows a list of some oily fish which you should aim to eat at least **twice a week**, but **no more than four times a week**.

**Oily Fish<br>Eat at least two portions a week, but no more than four**		
• Anchovies	• Sprats	• Kippers
• Herring	• Trout	• Pilchards
• Mackerel	• Whitebait	• Sardines
• Salmon	• Eel	• Swordfish

• Tuna (fresh not tinned)

Supplements

There are a range of supplements you can purchase that have been shown to have some effect on pain and inflammation caused by arthritis, these include **Fish Oils** (not to be confused with fish liver oil), **Glucosamine** (check with your GP before taking if you have diabetes), **Vitamin D, Calcium** and **Iron**.

Supplements are a good way to boost your intake of these beneficial substances but they should not be used in place of a healthy diet.

5.Holistic Medicine

Many people who have Arthritis find benefit in using some forms of complimentary or alternative medicine alongside their usual medication.

It is important that you **always discuss any complementary or alternative medicine with your GP** or the Rheumatology team before embarking on a treatment.

There are many alternatives to main stream medicine that may be of benefit to people with arthritis, these include:

- Acupuncture
- The Alexander technique
- Aromatherapy
- Copper bracelets
- Herbal medicine
- Massage
- Relaxation and hypnosis

Holistic medicine incorporating techniques such as these are generally safe but ensure that your therapist is legally registered before starting any treatment.

There is a lot more information on these general techniques and treatments on the Arthritis Research UK website (http://www.arthritisresearchuk.org/).

This booklet includes information written and published by Arthritis Research UK.

Additional information can be obtained from the Arthritis Research UK website (http://www.arthritisresearchuk.org/).

Information was also reproduced with the kind permission of Sarah Bradley and Kirsty Bancroft of Poole NHS Trust, and Christina Macleod of Hampshire Hospitals NHS Trust.

Notes:

Appendix 2

Self-Management Package: Arthritis Research UK Osteoarthritis Information Booklet *(see separate pdf as large file)*

Appendix 2

Self-Management Package: Facilitators and Barriers to Engaging with Self-Management Principles

1. Participant's ID number: _ _ - _ _ - _ _ _ _

2. My general exercise goal is:

3. My specific exercise goal – What am I going to do?

4. My Confidence

How confident am I that **I will achieve** my specific exercise goal? Please circle the appropriate number below.

Not at all confident 1 2 3 4 5 6 7 8 9 10 **Extremely confident**

5. My Commitment

How committed am I to **achieving** my specific exercise goal? Please circle the appropriate number below.

Not at all committed 1 2 3 4 5 6 7 8 9 10 **Extremely committed**

6. My Exercise Action Plan

It is important to measure and record your progress, so that you can see when you are succeeding as well as to work out what you can change if your plan is not working.

Where am I going to do the exercises? ....

When am I going to do the exercises? ....

**Patient**	**Practitioner**
I will do the exercises – my Specific Exercise Goal – and record my progress in my Exercise Diary and bring the Exercise Diary to my next consultation.	I will discuss with you your exercises (Specific Exercise Goal) and your Exercise Diary and clarify any questions you have.
**Signature:**	**Signature:** ....
**Date:**	**Date:** ....

Barriers and Facilitators to doing Exercise

**Unhelpful Things/Barriers**	**Helpful Things/Facilitators**
**Places and things:** Is there anything about the things around me or the places I am in that makes it difficult to do the exercises – my Specific Exercise Goal? What **can I do** to change this?	**Places and things:** Is there anything about the things around me or the places I am in that makes it easier to do the exercises – my Specific Exercise Goal? What **can I do** to use these helpful things?
**People:** Are there any people I spend time with who make it difficult to do the exercises – my Specific Exercise Goal? What **can I do** to change this?	**People:** Are there any people I spend time with who make it easier to do the exercises – my Specific Exercise Goal? What **can I do** to ask them to help me?
**Thoughts and feelings:** Is there anything that I am thinking and/or feeling that makes it difficult to do the exercises – my Specific Exercise Goal? What **can I do** to change this?	**Thoughts and feelings:** Is there anything that I am thinking and/or feeling that makes it easier to do the exercises – my Specific Exercise Goal? What **can I do** to encourage these thoughts and feelings?

Appendix 2

Self-Management Package: Exercise Diary

**Participant's ID number:** _ _ - _ _ - _ _ _ _	**Date of Occupational therapy/Physiotherapy Appointment when given the exercises: Date:**	_ _/_ _/_ _ _ _ (day/month/year)

Please do your hand exercises at least 3 times a week for at least 20 minutes each time. Please record how many times you did your exercise programme each day. If you didn't do your exercises please write **0**

**Week**	**You can record notes or dates in this column, however you like, to help you keep track of which week you are in.**	**How many times did you do your exercises today?** Write **0** if not at all.						
		**Day 1**	**Day 2**	**Day 3**	**Day 4**	**Day 5**	**Day 6**	**Day 7**
**Week 1**								
**Week 2**								
**Week 3**								
**Week 4**								
**Week 5**								
**Week 6**								
**Week 7**								
**Week 8**								
**Week 9**								
**Week 10**								
**Week 11**								
**Week 12**								

Appendix 3

Verum Splint Decision Protocol

PURPOSE:

This OTTER splint decision guideline is to be used for supporting your decision to issue the verum splint for those patients allocated to the second arm in the OTTER trial.

INSTRUCTIONS:

1. **The option for the OTTER trial is to provide either**

- PROCOOL THUMB CMC RESTRICTION SPLINT
  Code: PTRS/(with the remainder of the code relating to the size/ laterality).

OR

- Orfilight 2.5mm 3/32" micro perforated (beige) trouser leg splint (made from a pre-cut template which will be provided)

Trial participants will wear this option for 12 weeks.

2. Splint decision clinical guidelines

We recommend that the choice of the verum splint is made in collaboration and agreement with the trial participant. It is important to ensure that the trial participants know and see that they do have a choice of splint to wear for 12 weeks.

We recommend that the Pro-cool thumb splint is issued when:

a) there is a generalised ache that interferes with function
b) there is mild CMCJ instability
c) a more rigid splint is impractical

We recommend that a tailor made thermoplastic "trousers splint" is made when

a) mechanical joint pain is a consistent feature, suggesting instability
b) There are high demands placed on the thumb, either in work or leisure

3 Splint prescription guidelines

It is recommended that the participant is informed that the splint is worn during ADls that aggravate their pain. As a guideline patients should be aiming to wear their splints for a minimum of 6 hours a day, during waking hours only (not to be worn over- night). This time

can be made up of individual splint wear periods and interspersed with active hand use. It is not expected that patients wear their splint for 6 hours at a time or in one go.

Appendix 4

Splint Wear Guide for Participants (Verum and Placebo Groups)

OTTER II Patient Splint Wear Guidelines

As part of the OTTER Trial you have been given a thumb splint by your therapist. It is important that you wear this splint over the next 12 weeks.

We ask that you wear your splint:

- During all your daily tasks that cause you thumb pain
- For a minimum of 6 hours a day
- During the day only and do not wear overnight or when asleep during the night.

Thank you for following these guidelines.
Please do not hesitate to contact your therapist if you have any questions about how or when to wear your trial splint.

Appendix 5

Facilitators and Barriers to Engaging with Splint Wear

---

1. Participant's ID number: _ _ - _ _ - _ _ _ _

---

2. My general splint wearing goal is:

....

....

....

---

3. My specific splint wearing goal – What am I going to do?

....

....

....

....

---

4. My Confidence

How confident am I that **I will achieve** my specific splint wearing goal? Please circle the appropriate number below.

Not at all confident 1 2 3 4 5 6 7 8 9 10 **Extremely confident**

---

5. My Commitment

How committed am I to **achieving** my specific splint wearing goal? Please circle the appropriate number below.

Not at all committed 1 2 3 4 5 6 7 8 9 10 **Extremely committed**

---

6. My Splint Wearing Action Plan

It is important to measure and record your progress, so that you can see when you are succeeding as well as to work out what you can change if your plan is not working.

Where am I going to wear the splint? ……………………………………

When am I going to wear the splint? ……………………………………

**Patient**	**Practitioner**
I will wear my splint – my Specific Splint Wearing Goal – and record my progress using my Splint Wearing Diary, and bring the Splint Wearing Diary to my next consultation.	I will discuss with you your splint wearing regime (Specific Splint Wearing Goal) and your Splint Wearing Diary and clarify any questions you have.
**Signature:** ……………………	**Signature:** ……………………
**Date:** ……………………	**Date:** ……………………

Barriers and Facilitators to Wearing the Splint

Unhelpful Things/Barriers	Helpful Things/Facilitators
**Places and things:**<br>Is there anything about the things around me or the places I am in that makes it difficult to wear the splint – my Specific Splint Wearing Goal? What **can I do** to change this?	**Places and things:**<br>Is there anything about the things around me or the places I am in that makes it easier to wear the splint – my Specific Splint Wearing Goal? What **can I do** to use these helpful things?
**People:**<br>Are there any people I spend time with who make it difficult to wear the splint – my Specific Splint Wearing Goal? What **can I do** to change this?	**People:**<br>Are there any people I spend time with who make it easier to wear the splint – my Specific Splint Wearing Goal? What **can I do** to ask them to help me?
**Thoughts and feelings:**<br>Is there anything that I am thinking and/or feeling that makes it difficult to wear the splint – my Specific Splint Wearing Goal? What **can I do** to change this?	**Thoughts and feelings:**<br>Is there anything that I am thinking and/or feeling that makes it easier to wear the splint – my Specific Splint Wearing Goal? What **can I do** to encourage these thoughts and feelings?

Appendix 6

Splint Wear Diary

Participant's ID number: _ _ - _ _ - _ _ _ _

Date of Occupational therapy/Physiotherapy Appointment when given the splint. Date: _ _/_ _/_ _ _ _ (day/month/year)

Please wear your splint as explained in the **OTTER Patient Splint Wear Guidelines**

Please record how many hours you wore your splint each day. If you didn't wear your splint please write **0**

Week	You can record notes or dates in this column, however you like, to help you keep track of which week you are in.	How many hours did you wear your splint today? Write **0** if not at all.						
		Day 1	Day 2	Day 3	Day 4	Day 5	Day 6	Day 7
Week 1								
Week 2								
Week 3								
Week 4								
Week 5								
Week 6								
Week 7								
Week 8								
Week 9								
Week 10								
Week 11								
Week 12								

Appendix 7

Placebo Splint Decision Protocol

PURPOSE:

This splint decision protocol is to be used for supporting your decision to issue one of the two DMO splints for those patients allocated to the third arm in the OTTER trial.

INSTRUCTIONS:

1. **The option for the OTTER trial is to provide either**

1 A Thumb Sleeve Lite DMO splint with cut out thumb sections and a wrist strap - beige

OR

2 A Thumb Sleeve DMO splint with a solid thumb component and a wrist strap - black

Trial participants will be asked to wear this option for 12 weeks.

2. **Splint decision clinical guidelines**

We recommend that the choice of splint is made in collaboration with the trial participant. Please refer to your training on delivering this dynamic splint option to maintain outcome expectancy.

It is important that participants know that they do have a choice of splint to wear for 12 weeks and we encourage that the two DMO options are shown to them to consider.

We recommend that patients agree one type of splint design to use in collaboration with their therapist. We suggest that you agree with the participant to provide the splint design that they feel fits more comfortably, the one that appears the most appealing and the one which they would prefer to wear.

3. Splint prescription guidelines

It is recommended that the participant is informed that the splint is worn during ADls that aggravate their pain. As a guideline patients should be aiming to wear their splints for a minimum of 6 hours a day, during waking hours only (<u>not</u> to be worn over- night). This time can be made up of individual

splint wear periods and interspersed with active hand use. It is not expected that patients wear their splint for 6 hours at a time or in one go.

4. Fit

Both DMO splint designs should be fitted such that the wrist strap is proximal to the ulnar styloid and does not provide any support or pressure around the wrist joint.

Appendix 8

Data and Safety Monitoring Committee Members

Anisur Rahman (Chair), Department of Rheumatology, University College London, London, UK

Ross Wilkie, Arthritis Research UK Primary Care Centre, Keele University, UK

Ranjit Lall, Warwick Clinical Trials Unit, University of Warwick, Coventry, UK

Appendix 9

Trial Steering Committee Members

Sarah Dean (Chair), University of Exeter Medical School, University of Exeter, Exeter, UK

Cathy Ball, Kennedy Institute of Rheumatology, University of Oxford, Oxford, UK

Lindsay Bearne, Faculty of Life Sciences & Medicine, King's College London, London, UK

Elaine Dennison, MRC Lifecourse Epidemiology Unit, University of Southampton, Southampton, UK

Corinne Hutt Greenyer, School of Health Sciences, University of Southampton, Southampton, UK

Jo Adams, School of Health Sciences, University of Southampton, Southampton, UK
