# Supplementary material for: The Osteoarthritis Thumb Therapy (OTTER) II Trial: a study protocol for a three-arm multi-centre randomised placebo controlled trial of the clinical effectiveness and efficacy and cost-effectiveness of splints for symptomatic thumb base osteoarthritis
Source: BMJ Open. 2019 Oct 22;9(10):e028342. doi: 10.1136/bmjopen-2018-028342 (PMC6830636; doi:10.1136/bmjopen-2018-028342)
Supplement: Supplementary data [file bmjopen-2018-028342supp001.pdf]

Arthritis Research UK

Condition
Osteoarthritis

Osteoarthritis

This booklet provides information and answers to your questions about this condition.

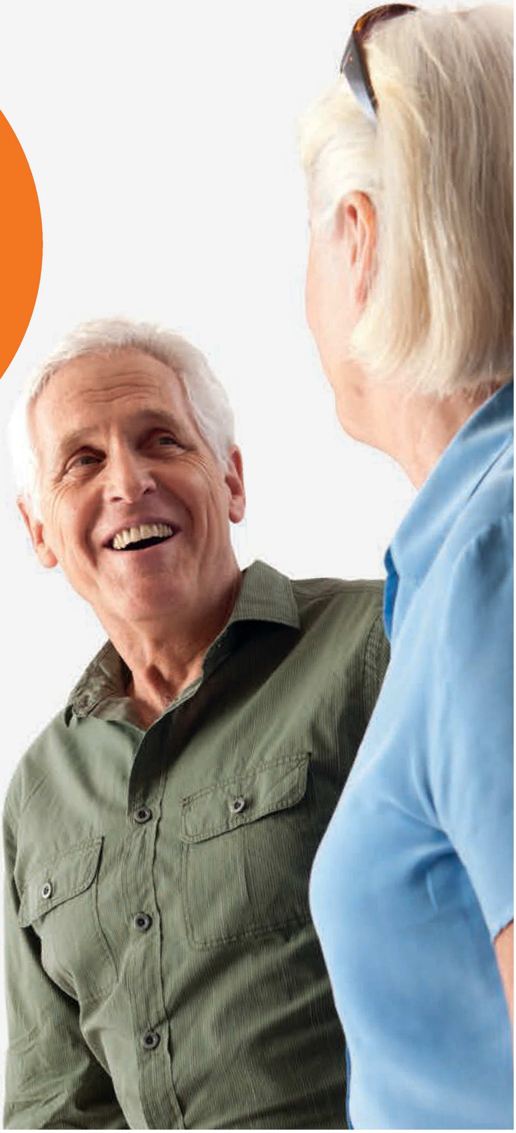

Arthritis Research UK produce and print our booklets entirely from charitable donations.

What is osteoarthritis?

Osteoarthritis is by far the most common form of joint disease. It causes pain and stiffness in the joints and affects approximately 8 million people in the UK. In this booklet we'll explain how osteoarthritis develops, what causes it and how it can be treated. We'll also give some hints and tips to help you manage your arthritis and suggest where you can find out more.

At the back of this booklet you'll find a brief glossary of medical words – we've underlined these when they're first used.

www.arthritisresearchuk.org

Arthritis Research UK
Osteoarthritis

What's inside?

2 Osteoarthritis at a glance

4 How does a normal joint work?

4 What is osteoarthritis?

6 What are the symptoms of osteoarthritis?

7 What causes osteoarthritis?

8 Which joints are affected?
- The knee
- The hip
- The hand
- The back and neck
- The foot

11 What is the outlook?

12 What are the possible complications?

12 How is osteoarthritis diagnosed?
- What tests are there?

14 What can I do to help myself?
- Exercise
- Weight management
- Tablets and creams
- Reducing the strain on your joints
- Complementary medicine

20 What treatments are there for osteoarthritis?
- Capsaicin cream
- Drugs
- Steroid injections
- Transcutaneous electrical nerve stimulation (TENS)
- Surgery

22 Self-help and daily living

23 Research and new developments

24 Glossary

26 Where can I find out more?

32 We're here to help

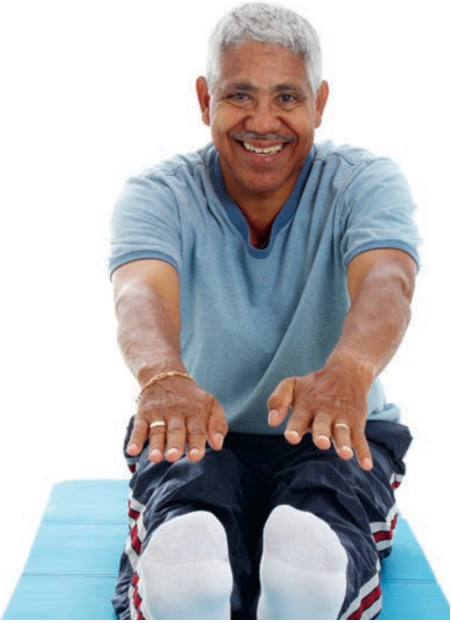

At a glance
Osteoarthritis

Osteoarthritis is a condition that affects the joints, causing pain and stiffness.

What is osteoarthritis?

Osteoarthritis is a condition that affects the joints, causing pain and stiffness. It's by far the most common form of joint disease, affecting people all over the world and approximately 8 million people in the UK.

What are the symptoms?

Symptoms of osteoarthritis can include:

- pain
- stiffness
- a grating or grinding sensation (crepitus) when the joint moves
- swelling (either hard or soft)
- not being able to use the affected joint normally, which can make it difficult to do certain activities (for example climbing stairs).

Who gets it?

Almost anyone can get osteoarthritis but it's most likely if:

- you're in your late 40s or older
- you're a woman
- your parents have had osteoarthritis
- you're overweight
- you've had a previous joint injury
- you have a physically demanding job where you make repetitive movements
- your joints have been damaged by another disease, for example gout or rheumatoid arthritis.

Arthritis Research UK
Osteoarthritis

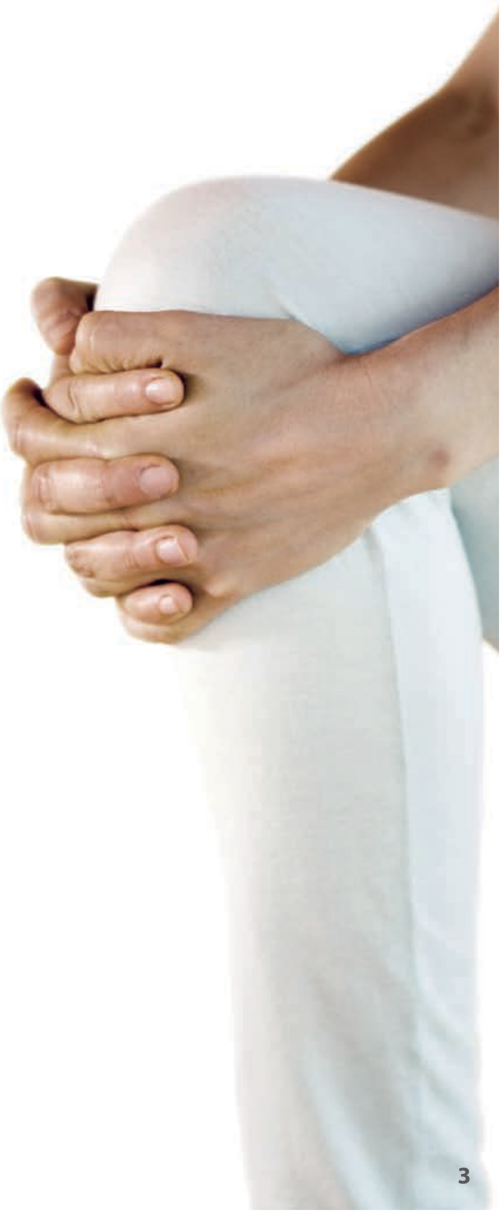

What can I do to help myself?

There are several ways you can help yourself, including:

- exercising regularly (both muscle-strengthening and general aerobic exercise)
- reducing stress on the affected joint (for example by pacing activities, using a walking stick or wearing suitable footwear)
- losing weight if you're overweight
- using over-the-counter painkillers such as paracetamol or low-dose ibuprofen, or pain-relieving creams, gels or sprays.

What treatments are there?

If you still have pain after trying self-help measures, your doctor may recommend the following:

- advice from a physiotherapist about exercise plans and gradually increasing your aerobic exercise
- capsaicin cream
- stronger painkillers, for example tramadol
- steroid injections into the painful joint
- surgery, including joint replacement.

How does a normal joint work?

A joint is where two or more bones meet (see Figure 1). The joint allows the bones to move freely but within controlled limits. The knees have additional rings of cartilage between the bones. These are called menisci – they act a bit like shock absorbers to spread the load more evenly across the joint.

What is osteoarthritis?

Osteoarthritis is a disease that affects your joints. The surfaces within your joints become damaged so the joint doesn't move as smoothly as it should (see Figures 2 and 3). The condition is sometimes called arthrosis or osteoarthrosis. Older terms are degenerative joint disease or wear and tear.

When a joint develops osteoarthritis, some of the cartilage covering the ends of the bones gradually roughens and becomes thin, and the bone underneath thickens. All the tissues within the joint become more active than normal – as if your body is trying to repair the damage:

- The bone at the edge of the joint grows outwards, forming bony spurs called osteophytes.
- The synovium may thicken and produce extra fluid, which then causes the joint to swell.
- The capsule and ligaments slowly thicken and contract as if they were trying to stabilise the joint.

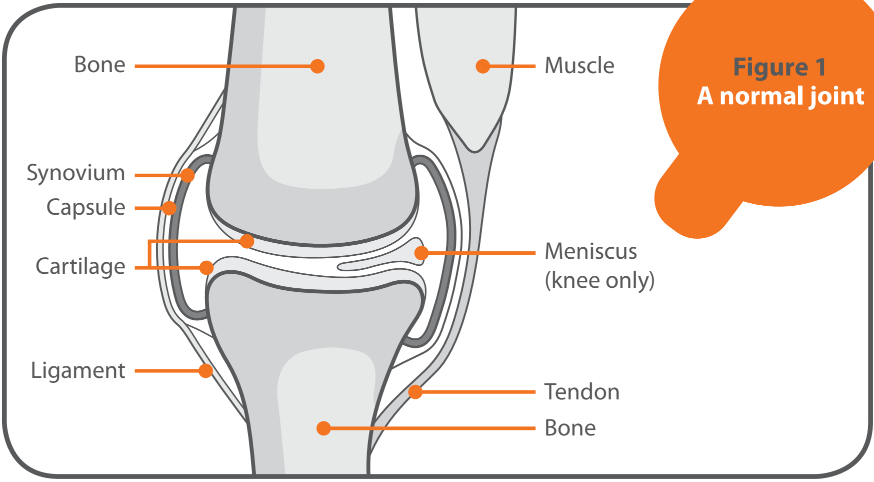

Figure 1
A normal joint

Arthritis Research UK
Osteoarthritis

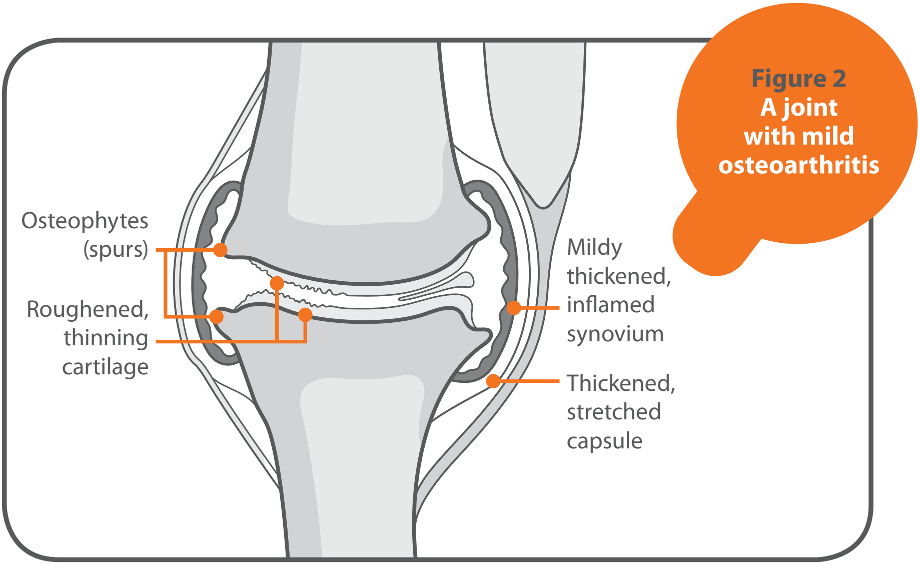

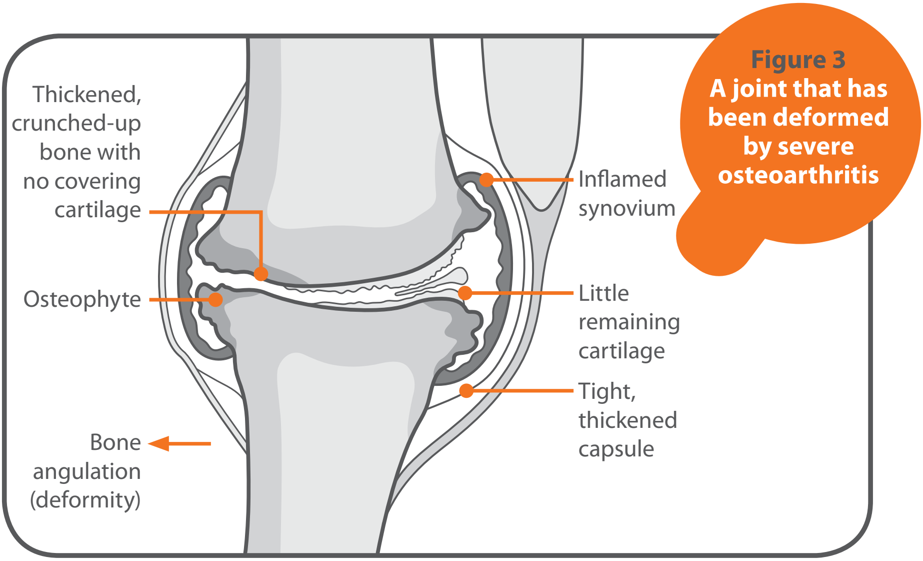

Adams J, *et al. BMJ Open* 2019; 9:e028342. doi: 10.1136/bmjopen-2018-028342

Sometimes your body's repairs are quite successful and the changes inside the joint won't cause pain or problems. In severe osteoarthritis, the cartilage can become so thin that it no longer covers the ends of your bones. The bones start to rub against each other and eventually start to wear away. The loss of cartilage, the wearing of bone and the bony spurs can alter the shape of the joint, forcing the bones out of their normal position.

What are the symptoms of osteoarthritis?

The main symptoms of osteoarthritis are pain and sometimes stiffness in the affected joints. The pain tends to be worse when you move the joint or at the end of the day, and it may make it difficult to get to sleep. Your joints may feel stiff after rest, but this usually wears off after a minute or two as you get moving.

If you have severe osteoarthritis, you may feel pain more often. The joint may not move as freely or as far as normal, and it may creak or crunch as you move. Sometimes it may give way because your muscles have weakened or the joint structure has become less stable, although exercises to strengthen your muscles can help to prevent this.

You may notice that the affected joint looks swollen. The swelling may be hard (caused by osteophytes) or soft (caused by synovial thickening and extra fluid in the joint), and the muscles around the joint may look thin or wasted.

Figure 4 Risk factors for osteoarthritis

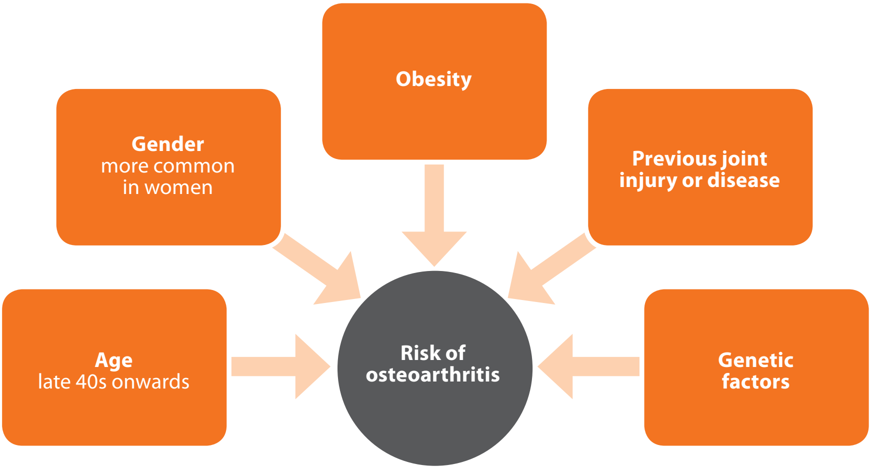

Adams J, *et al. BMJ Open* 2019; 9:e028342. doi: 10.1136/bmjopen-2018-028342

Arthritis Research UK
Osteoarthritis

Symptoms often vary for no obvious reason – you'll probably have good and bad spells. Some people find that changes in the weather make the pain worse, especially damp weather along with falling atmospheric pressure. Others find the pain varies depending on how active they've been.

In more severe cases, the pain may be constant. It may prevent you from sleeping and cause difficulties in your daily activities; for example, osteoarthritis in the knee or hip can make it difficult to climb stairs or get up from a chair.

What causes osteoarthritis?

There are many factors that can increase your risk of osteoarthritis, and it's often a combination of these that leads to the condition (see Figure 4):

- **Age** – Osteoarthritis usually starts from the late 40s onwards. We don't fully understand why it's more common in older people, but it might be due to factors like the muscles weakening and the body being less able to heal itself, or gradual wearing out of the joint with time.
- **Gender** – For most joints, especially the knees and hands, osteoarthritis is more common and more severe in women.
- **Obesity** – Being overweight is an important factor in causing osteoarthritis, especially in the knee. It also increases the chances of osteoarthritis becoming progressively worse.

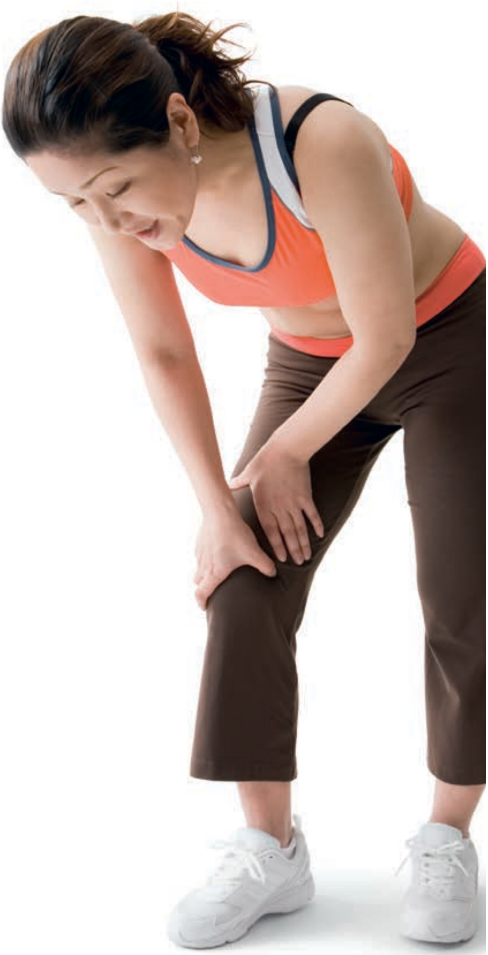

- **Joint injury** – A major injury or operation on a joint may lead to osteoarthritis in that joint later in life. Normal activity and exercise don't cause osteoarthritis, but very hard, repetitive activity or physically demanding jobs can increase your risk.
- **Joint abnormalities** – If you were born with abnormalities or developed them in childhood, it can lead to earlier and more severe osteoarthritis than usual. Perthes' disease of the hips is an example.
- **Genetic factors** – Nodal osteoarthritis, which particularly affects the hands of middle-aged women, runs strongly in families, although it's not yet clear which genes are involved. And some rare forms of osteoarthritis which start at an earlier age are linked with genes that affect collagen (an essential part of cartilage). Genetic factors play a smaller, but still significant, part in osteoarthritis of the hip and knee.
- **Other types of joint disease** – Sometimes osteoarthritis is a result of damage from a different kind of joint disease, such as rheumatoid arthritis or gout.

Which joints are affected?

Almost any joint can develop osteoarthritis, especially if it's been badly injured, but it most often affects the knees, hips, hands, spine and big toes (see Figure 5):

The knee

Osteoarthritis of the knee is very common. This is probably because your knee has to take extreme stresses, twists and turns.

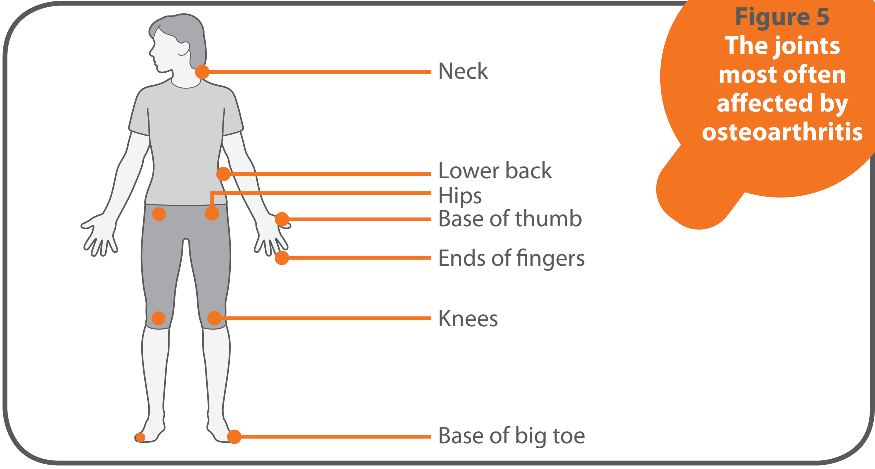

Figure 5 The joints most often affected by osteoarthritis

Any joint can be affected by osteoarthritis.

Osteoarthritis can affect the main surfaces of the knee joint and also the cartilage underneath your kneecap (patella).

You're most likely to feel pain at the front and sides of your knee. If the osteoarthritis is severe, your knees may become bent and bowed. Your knee joint may also become unstable so that it gives way when you put weight on it. This is usually because of muscle weakness in the thigh but sometimes because of damage to the ligaments.

Osteoarthritis of the knee is twice as common in women as in men and it usually affects both knees. It causes most problems from the late 50s onwards. A number of factors can increase the risk of osteoarthritis of the knee, for example:

- being overweight
- having nodal osteoarthritis (particularly in women)
- a previous sporting injury (such as a torn meniscus or ligament)
- an operation to remove torn cartilage (meniscectomy).

See Arthritis Research UK booklet *Osteoarthritis of the knee.*

The hip

Osteoarthritis of the hip is also very common and can affect either one or both hips. The pain is most likely to be deep at the front of your groin, but you may also feel pain at the side and front of your thigh, in your buttock or down to your knee (this is called referred pain).

If you have severe hip osteoarthritis, you may find the affected leg seems a little shorter than the other because of the bone on either side of the joint being crunched up.

Men and women are equally likely to develop hip osteoarthritis, and it usually starts from the late 40s onwards. The risk may be greater if you had hip problems at birth (congenital dislocation) or abnormal hip development in childhood, such as Perthes' disease. Physical work such as farming may also increase the risk; however, there's often no obvious cause.

The hand

Osteoarthritis of the hands usually occurs as part of nodal osteoarthritis. This mainly affects women and often starts in your 40s or 50s, around the time of the menopause. It usually affects the base of your thumb

and the joints at the ends of your fingers, although other finger joints can also be affected. At times these joints become swollen and tender, especially when the condition first appears.

Over several years, firm knobbly swellings form on the finger joints. These are caused by osteophytes and are known as Heberden's nodes when they're at the end joints of your fingers (see Figure 6) or Bouchard's nodes when they're at the mid-finger joints. Once the nodes are fully formed, the pain and tenderness often improve. Although the fingers are knobbly and sometimes slightly bent, they usually still work well. Arthritis at the base of your thumb may cause longer-lasting problems.

Having nodal osteoarthritis in middle age means you're more likely to develop osteoarthritis of the knee, and possibly other joints, as you get older. Nodal osteoarthritis tends to run in families much more than other forms of osteoarthritis and it's especially likely to be passed from mother to daughter. It's not yet known which genes are involved so it's not possible to test for this.

The back and neck

Changes that affect the bones of your spine and the discs between the bones are often called spondylosis, but they're very similar to the changes caused by osteoarthritis in other joints. X-rays show that spondylosis is extremely common, but it's not the most common cause of back or neck pain and often doesn't cause any problems at all.

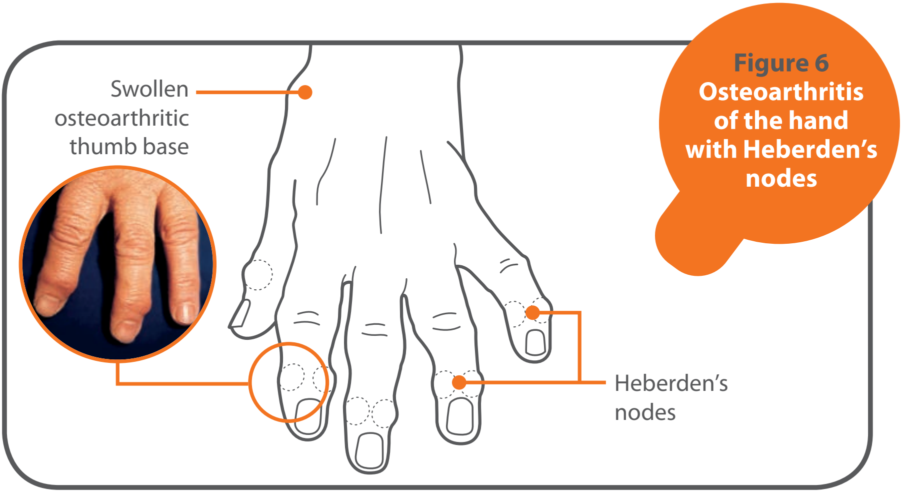

Figure 6 Osteoarthritis of the hand with Heberden's nodes

Photography used with kind permission of Elsevier. © Elsevier 2011. Taken from *Rheumatology*, fifth edition. Edited by Marc C Hochberg, Alan J Silman, Josef S Smolen, Michael E Weinblatt and Michael H Weisman.

See Arthritis Research UK booklets
Back pain; Neck pain.

The foot

Osteoarthritis of the foot generally affects the joint at the base of your big toe. Eventually your toe may become stiff (hallux rigidus), which can make it difficult and painful to walk, or bent (hallux valgus), which can lead to painful bunions (see Figure 7).

Osteoarthritis of the mid-foot is also quite common, especially in older people, and may cause an obvious bony swelling (osteophyte) on the top of your mid-foot. Ankle osteoarthritis is least common and may cause your heel to move to an unusual angle.

See Arthritis Research UK booklets
Feet, footwear and arthritis.

What is the outlook?

It's impossible to predict how osteoarthritis will develop for any one person. It can sometimes develop over just a year or two and cause a lot of damage to a joint, which may then cause some deformity or disability. But more often osteoarthritis is a slow process that develops over many years and results in fairly small changes in just part of the joint. This doesn't mean it won't be painful, but it's less likely to cause severe deformity or disability. Sometimes the condition reaches a peak a few years after the symptoms start and then remains the same, or it may even improve.

Unlike rheumatoid arthritis, osteoarthritis doesn't affect other parts of your body – it's purely a joint disease. Also, because there's very little, if any, inflammation in osteoarthritic joints, the condition doesn't

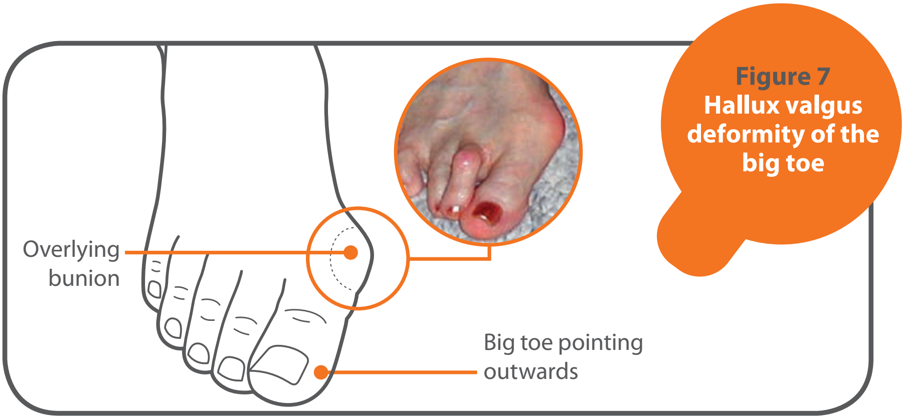

Figure 7
Hallux valgus deformity of the big toe

Photography used with kind permission of Elsevier. © Elsevier 2011. Taken from *Rheumatology*, fifth edition. Edited by Marc C Hochberg, Alan J Silman, Josef S Smolen, Michael E Weinblatt and Michael H Weisman.

make you feverish or unwell. However, some people with osteoarthritis will develop other illnesses purely by chance.

What are the possible complications?

The changes in cartilage that occur with osteoarthritis can encourage crystals to form within the joint. Gout is a common type of inflammatory arthritis, which is caused by high levels of uric acid that lead to sodium urate crystals forming in and around joints. If you have both osteoarthritis and a high serum uric acid, you're at an increased risk of developing gout. The base of the big toe is a very common site for a painful attack of gout, and this is partly because this joint is the most common joint in the foot to be affected by osteoarthritis.

See Arthritis Research UK booklet *Gout.*

Osteoarthritis doesn't lead to rheumatoid arthritis or other types of joint disease.

Osteoarthritis can also encourage calcium pyrophosphate crystals to form in the cartilage. This is called calcification or chondrocalcinosis. It can happen in any joint, with or without osteoarthritis, but it's most likely to occur in a knee already affected by osteoarthritis, especially in older people. The crystals will show up in x-rays and they can also be seen under a microscope in samples of fluid taken from the joint.

Osteoarthritis tends to become more severe more quickly when there are calcium crystals present. Sometimes the crystals can shake loose from the cartilage, causing a sudden attack of very painful swelling called acute calcium pyrophosphate crystal arthritis (acute CPP crystal arthritis), which was sometimes previously called 'pseudogout'.

See Arthritis Research UK booklet *Calcium crystal diseases including acute CPP crystal arthritis (pseudogout) and acute calcific tendinitis.*

How is osteoarthritis diagnosed?

It's very important to get an accurate diagnosis if you think you have arthritis. There are many different types of arthritis and some need very different treatments. Osteoarthritis is usually diagnosed based on your symptoms and the physical signs that your doctor finds when examining your joints. The signs your doctor will be checking for are:

Arthritis Research UK
Osteoarthritis

- tenderness over the joint
- creaking or grating of the joint (crepitus)
- bony swelling
- excess fluid
- restricted movement
- joint instability
- thinning of the muscles that support the joint.

What tests are there?

There's no blood test for osteoarthritis, although your doctor may suggest you have them to help rule out other types of arthritis.

X-rays are the most useful test to confirm osteoarthritis, although they often won't be needed. An x-ray may show changes such as bony spurs or narrowing of the space between the bones. They'll also show whether there are any calcium deposits within the joint.

However, x-rays can't really show how much pain or disability you're likely to have. Some people have a lot of pain from fairly minor joint damage, while others have little pain from more severe damage.

Rarely, a magnetic resonance imaging (MRI) scan of the knee can be helpful. This will show the soft tissues (for example cartilage, tendons, muscles) and changes in the bone that can't be seen on a standard x-ray. Its main use is to identify another joint or bone problem in someone whose symptoms aren't typical of osteoarthritis, for example, in a person with a torn knee meniscus that causes intermittent 'locking' of the knee.

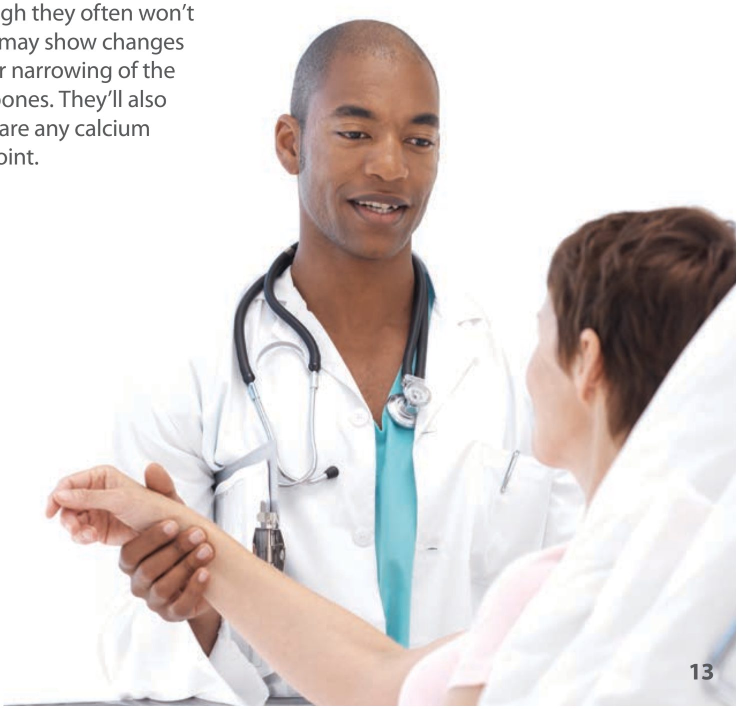

What can I do to help myself?

There's no cure for osteoarthritis as yet, but there's a lot that you can do to improve your symptoms. Self-help measures play a very important part in relieving the pain and stiffness, and reducing the chances of your arthritis becoming worse.

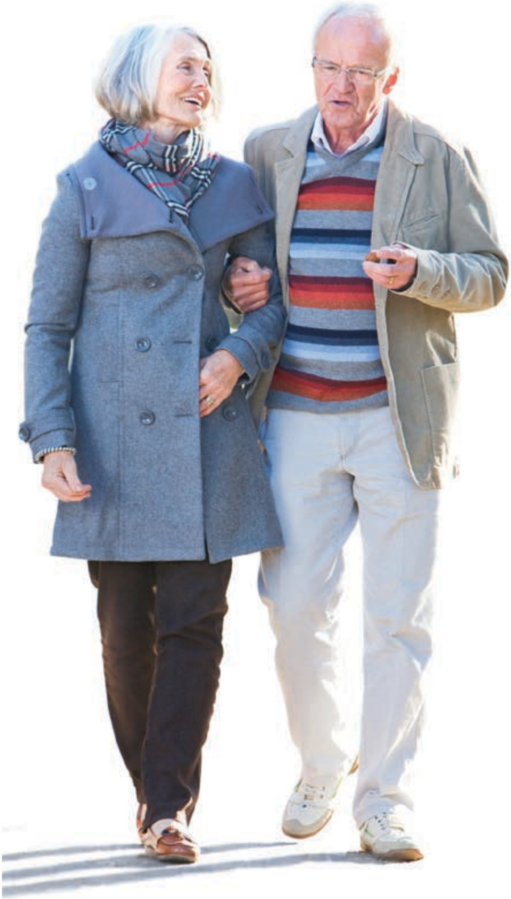

Exercise

It's very important to keep your joints moving. You'll need to find the right balance between rest and exercise – most people with osteoarthritis find that too much activity increases their pain while too little makes their joints stiffen up. Little and often is usually the best approach to exercise if you have osteoarthritis.

There are two main types of exercise that you'll need to do:

Strengthening exercises will improve the strength and tone of the muscles that control the affected joint. Osteoarthritis can weaken these muscles. This is particularly important for the thigh (quadriceps) muscles if you have osteoarthritis of the knee. Regular exercising of the muscles, such as straight-leg raises (see the pull-out section at the back of the booklet), helps to stabilise and protect the joint and has also been shown to reduce pain. It's also particularly helpful in preventing the knee giving way and reducing the chances of stumbling or falling.

Because knee and hip osteoarthritis may come to affect both sides of your body, and because both legs work as a unit when you walk, it's helpful to do strengthening exercises on the muscles on the other leg, even if that knee or hip isn't causing symptoms, and also to do hip exercises if you have knee osteoarthritis (and vice versa).

Arthritis Research UK
Osteoarthritis

Aerobic exercise is any exercise that increases your pulse rate and makes you a bit short of breath (for example a brisk walk, swimming or using an exercise bike). Regular aerobic exercise should help you sleep better, is good for your general health and well-being, and can also reduce pain by raising the levels of pain-relieving hormones called endorphins.

A physiotherapist can advise you on the best exercises for the type of osteoarthritis you have, but you'll need to build them into your daily routine to get the most from them. You can also talk to your GP about the Exercise on Prescription scheme that's available in some areas.

Swimming can be very good for osteoarthritis. Because the water supports the weight of your body, you won't be putting a lot of strain on your joints as you exercise. Your physiotherapist may also recommend special exercises in a hydrotherapy pool. This can help get muscles and joints working better and, because the water is warmer than in a typical swimming pool, it can be very soothing and relaxing.

T'ai chi is another type of exercise that has been shown to be helpful at reducing the pain from osteoarthritis. Many people find that regular t'ai chi also makes them feel better in other ways, for example, less stressed during the day and better able to sleep at night.

See Arthritis Research UK booklets
Hydrotherapy and arthritis; Keep moving; Physiotherapy and arthritis.

Weight management

There's a great deal of evidence that being overweight increases the strain on your joints – especially your knees. Research shows that being overweight or obese not only increases your risk of developing osteoarthritis but also makes it more likely that your arthritis will get worse over time.

Because of the way the joints work, the force put through your knees when you walk, especially on stairs and slopes, can be several times your actual body weight. Losing even a small amount of weight can make a big difference to the strain on weight-bearing joints.

No special diet has been shown to help with osteoarthritis, but if you need to lose some weight we would recommend a balanced, reduced-calorie diet combined with regular exercise.

See Arthritis Research UK booklet
Diet and arthritis.

Tablets and creams

There are a number of tablets and creams that can help osteoarthritis symptoms, and because they work in different ways you can combine different treatments if you need to. Your pharmacist can advise you and supply paracetamol, and some low-dose tablets and creams without a prescription.

Anti-inflammatory creams and gels are especially helpful for osteoarthritis of the knee or hand.

Painkillers (analgesics) and non-steroidal anti-inflammatory drugs (NSAIDs)

Painkillers (analgesics) often help with the pain and stiffness, although they don't affect the arthritis itself and won't repair damage to the joint. They're best used occasionally when the pain is very bad or when you're likely to be exercising. Paracetamol is usually the best and safest painkiller to try first, but make sure you take the right dose as most people take too little. You should try taking 1 g (usually two tablets) three or four times a day. It's best to take them before the pain becomes very bad but you shouldn't take them more often than every four hours.

Combined painkillers (for example co-codamol) contain paracetamol and a second codeine-like drug, and they may be helpful for more severe pain. They're stronger than paracetamol and are therefore more likely to cause side-effects such as constipation or dizziness.

Over-the counter non-steroidal anti-inflammatory drugs (NSAIDs), such as ibuprofen, can also help. You can use these for a short course of treatment (about 5–10 days), but if they've not helped within this time then they're unlikely to. If the pain returns when you stop taking the tablets, try another short course.

If you're already taking NSAID tablets, speak to your doctor about non-NSAID creams (for example capsaicin cream) to avoid taking too much of one type of drug.

! You shouldn't take ibuprofen or aspirin if you're pregnant, or if you have asthma, indigestion or a stomach (gastrointestinal) ulcer, until you've spoken with your doctor or pharmacist.

If you know you're going to be more active than usual, try taking a painkiller before you start to avoid increased pain later.

i **See Arthritis Research UK leaflets**
Non-steroidal anti-inflammatory drugs; Painkillers.

Anti-inflammatory creams and gels

You can apply anti-inflammatory creams and gels directly onto painful joints three times a day. There's no need to rub them in – they absorb through the skin on their own. They're especially helpful for osteoarthritis of the knee or hand but not for deep joints such as the hip. They're extremely well tolerated as very little is absorbed into the bloodstream. If you have trouble taking tablets then anti-inflammatory creams are a particularly

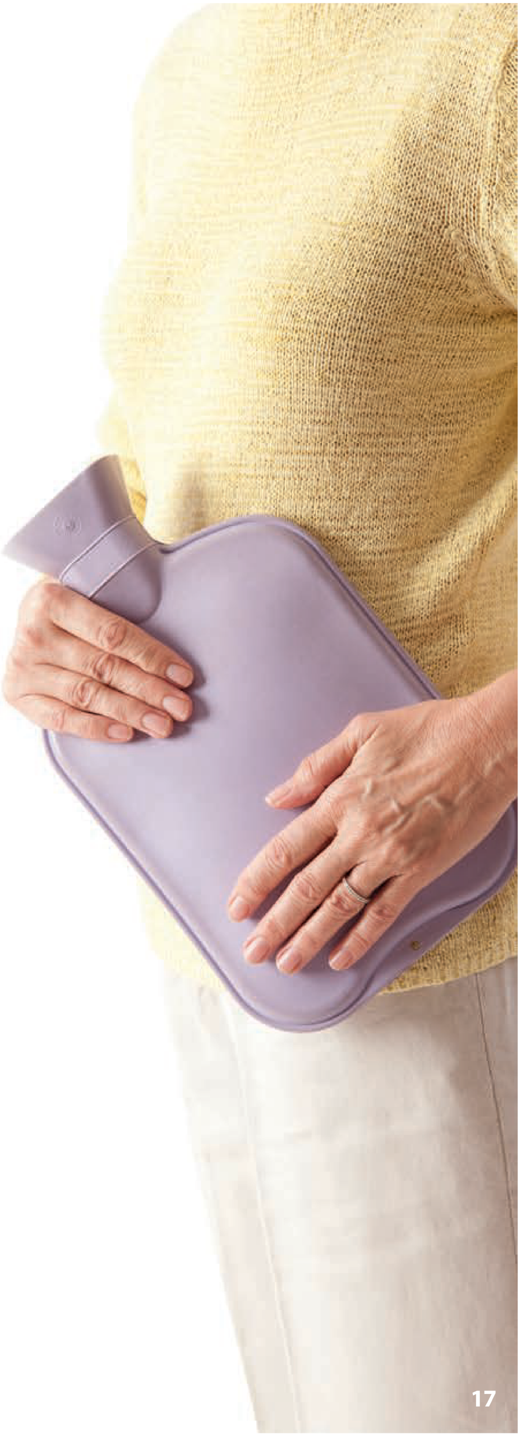

good option to try. You can decide if they help your pain within the first few days of trying them.

Reducing the strain on your joints

Apart from keeping an eye on your weight, there are a number of other ways you can reduce the strain on your joints:

- Pace your activities through the day – don't tackle all the physical jobs at once. Break the harder jobs up into chunks and do something more gentle in between.
- Wear low-heeled shoes with soft, thick soles (trainers are ideal). Thicker soles will act as shock absorbers for your feet, knees, hips and back. High heels will alter the angle of your hips, knees and big toe joints and put additional strain on them.
- Use a walking stick to reduce the weight and stress on a painful hip or knee. A therapist or doctor can advise on the correct length and how to put your weight through the stick when you're putting weight on your affected joint.
- Use the handrail for support when climbing stairs – this is particularly important if you have osteoarthritis of the knee.
- Keep your joints moving – in particular, don't keep an osteoarthritic knee still in a bent position for too long as this will eventually affect the muscles.

Reducing strain on your joints can help with osteoarthritis.

- Think about modifying your home, car or workplace to reduce unnecessary strain on your joints. An occupational therapist can advise you on how to protect your joints and on special equipment or gadgets that will make your daily tasks easier.
- Learn to relax your muscles and get the tension out of your body. A physiotherapist or occupational therapist can advise you on relaxation techniques.
- Sex can sometimes be painful, particularly for women with osteoarthritis of the hips. Trying a different position can often help.

See Arthritis Research UK booklets *Looking after your joints when you have arthritis; Occupational therapy and arthritis; Sex and arthritis.*

Applying warmth to a painful joint often relieves the pain and stiffness of osteoarthritis. Heat lamps are popular, but a hot-water bottle or reheatable pad are just as effective. This can be helpful if you have a flare-up of pain when you've done a bit too much. An ice pack can also help, but don't apply either ice or heat packs directly to your skin.

More evidence to support the use of knee braces for osteoarthritis is becoming available. There are several types that can help to stabilise the kneecap and make it move correctly. You can buy knee braces from sports shops and chemists, but you should speak to your doctor or physiotherapist first. They may also be able to provide braces or recommend the best ones for you.

Complementary medicine

There are many different complementary and herbal remedies that claim to help with arthritis, and some people do feel better when they use some complementary treatments. However, on the whole these treatments aren't recommended for use on the NHS because there's no clear evidence that they work. There's no scientific evidence, for example, that copper bracelets can help ease osteoarthritis-related pain.

Glucosamine and chondroitin

Many people try glucosamine and chondroitin tablets. These are compounds that are normally found in joint cartilage, and some studies suggest that taking supplements may improve the health of damaged cartilage. Other studies, however, don't show any benefit so we still

don't know for sure whether they work or not. Glucosamine and chondroitin, which are similar to each other, are available from your chemist or health food store. You'll need to take a dose of 1.5 g glucosamine sulphate a day, and you may need to take them for several weeks before you can tell whether they're making a difference.

Glucosamine hydrochloride doesn't appear to be effective, so always check that you're taking the sulphate variety.

Most brands of glucosamine are made from shellfish. If you're allergic to shellfish, make sure you use a vegetarian or shellfish-free variety. Glucosamine can affect the level of sugar in your blood, so if you have diabetes you should keep an eye on your blood sugar levels and see your doctor if they increase. You should also see your doctor for regular blood checks if you're taking the blood-thinning drug warfarin.

Homeopathy

Many people are interested in homeopathic remedies, and a number of different types are used for osteoarthritis; however, there's no conclusive scientific evidence that the remedies are effective.

Acupuncture

There's some research showing that acupuncture can sometimes provide relief from arthritis pain, although the effect may be short-lived, which means that you'll need repeat sessions. Other studies show no benefit from acupuncture.

Chiropractic and osteopathy

Manipulation by a chiropractor or osteopath can often help neck and back pain, although the use of manipulation for osteoarthritis in other joints is limited. If you do want to try it, make sure you choose a practitioner who is registered with the appropriate regulatory body.

See Arthritis Research UK booklet and special reports
Complementary and alternative medicine for arthritis; Complementary and alternative medicines for the treatment of rheumatoid arthritis, osteoarthritis and fibromyalgia; Practitioner-based complementary and alternative therapies for the treatment of rheumatoid arthritis, osteoarthritis, fibromyalgia, and low back pain.

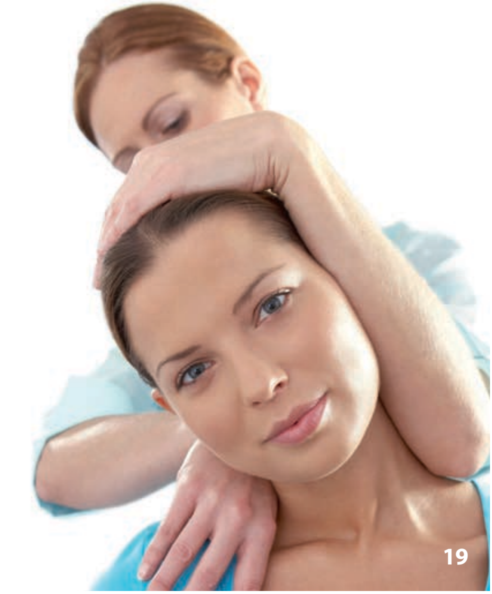

What treatments are there for osteoarthritis?

Many people find that self-help measures, such as those listed above, are enough to help them manage their symptoms, but your healthcare team will be able to suggest other treatments if you need them.

Capsaicin cream

Capsaicin cream is made from the pepper plant (capsicum) and is an effective and very well-tolerated painkiller. It needs to be applied regularly three times each day to be effective and, like NSAID creams and gels, it's particularly useful for knee and hand osteoarthritis. It's only available on prescription.

Most people feel a warming or burning sensation when they first use capsaicin, but this generally wears off after several days. The pain-relieving effect starts after several days of regular use and you should try it for at least two weeks before deciding if it has helped.

Drugs

Painkillers

If you have severe pain and other medications aren't giving enough relief, your doctor may recommend stronger painkillers (or opioids) such as tramadol, nefopam or meptazinol. Stronger painkillers are more likely to have side-effects – especially nausea, dizziness and confusion – so you'll need to see your doctor regularly and report any problems you have with these drugs.

Some opioids (for example fentanyl) can be given as a plaster patch which you place on your skin – these can give pain relief for a number of days.

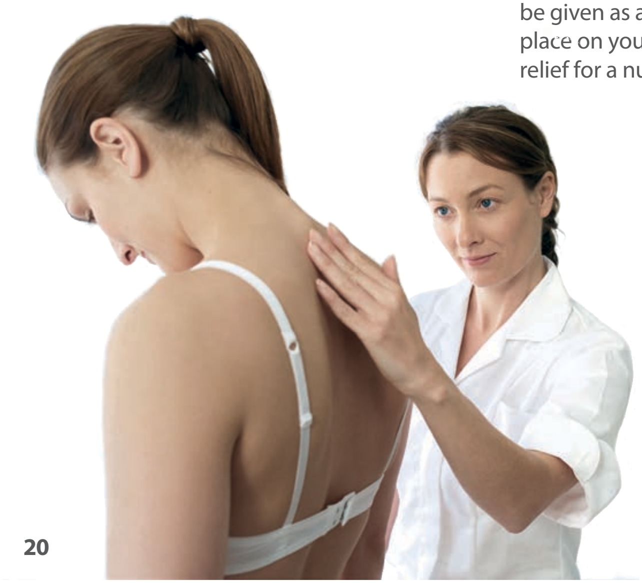

Arthritis Research UK
Osteoarthritis

Non-steroidal anti-inflammatory drugs (NSAIDs)

If inflammation in the joint is causing pain and stiffness, a short course of NSAID tablets (for example naproxen) may be useful.

Like all drugs, NSAIDs can sometimes have side-effects, but your doctor will take precautions to reduce the risk of these – for example, by prescribing the lowest effective dose for the shortest possible period of time.

NSAIDs can cause digestive problems (stomach upsets, indigestion, or damage to the stomach lining) so in most cases they'll be prescribed along with a drug called a proton pump inhibitor (PPI), which will help to protect your stomach.

NSAIDs also carry an increased risk of heart attack or stroke. Although the increased risk is small, your doctor will be cautious about prescribing them if there are other factors that may increase your overall risk – for example, smoking, circulation problems, high blood pressure, high cholesterol or diabetes. NSAIDS can also reduce kidney function so you shouldn't take them if you have known reduced kidney function or are on a water tablet (diuretic).

If you have trouble opening child-proof containers, your pharmacist will put your tablets in a more suitable container for you. Contact us for our special request card which you can hand to your pharmacist with your prescription.

Steroid injections

Steroid injections are sometimes given directly into a particularly painful joint. The injection can start to work within a day or so and may improve pain for several weeks or even months, especially in your knee or thumb. This is mainly used for very painful osteoarthritis, for sudden, painful attacks caused by the shedding of calcium pyrophophate crystals, or to help people through an important event (such as a holiday or family wedding).

See Arthritis Research UK drug leaflet *Local steroid injections.*

Steroid injections can improve pain for several weeks or even months.

Transcutaneous electrical nerve stimulation (TENS)

Some people find that transcutaneous electrical nerve stimulation (TENS) can help to relieve pain, although research evidence on its effectiveness is mixed. A TENS machine is a small electronic device that sends pulses to your nerve endings via pads placed on your skin. It produces a tingling sensation and is thought to alter pain messages sent to the brain. TENS machines are available from pharmacies and other major stores, but a physiotherapist may be able to loan you one to try before you decide whether to buy one.

Surgery

Surgery may be recommended if your pain is very severe or you have mobility problems. Many thousands of hip and knee replacements are performed each year for osteoarthritis, and other joint replacements are becoming increasingly common. Surgery can be very good for easing pain in cases where other treatments haven't given enough relief.

Sometimes keyhole surgery techniques may be used to wash out loose fragments of bone and other tissue from your knee – this is called arthroscopic lavage and it's not recommended unless your knee locks.

See Arthritis Research UK booklets *Foot and ankle surgery; Hand and wrist surgery; Hip replacement surgery; Knee replacement surgery; Shoulder and elbow joint replacement.*

Self-help and daily living

Sleep

If pain is a problem at night, heat may help. Try a hot bath before going to bed, or use a hot-water bottle, wheat bag (which you can heat in a microwave) or electric blanket. Taking a painkiller two hours before going to bed can ease night-time pain so you can get to sleep more easily.

See Arthritis Research UK booklet *Sleep and arthritis.*

Work

Most people with osteoarthritis are able to continue in their jobs, although you may need to make some alterations to your working environments, especially if you have a physically demanding job. Speak to your employer's occupational health service if they have one, or your local Jobcentre Plus can put you in touch with Disability Employment Advisors who can arrange work assessments. They can advise you on changing the way you work and on equipment that may help you to do your job more easily. If necessary, they can also help with retraining for more suitable work.

See Arthritis Research UK booklet *Work and arthritis.*

Dealing with stress

Living with a long-term condition like osteoarthritis can lower your morale and may affect your sleep. It's important to

tackle problems like these as they could lead to depression and will certainly make the osteoarthritis more difficult to cope with. It often helps to talk about negative feelings, so it could be useful to speak to your healthcare team, or your family and friends. Support groups are also available – your doctor may be able to tell you about organisations in your area.

See Arthritis Research UK booklets and guide *Fatigue and arthritis; Pain and arthritis; Living with long-term pain: a guide to self-management*.

Research and new developments

Research has already shown the importance of exercise and weight management in reducing the pain of osteoarthritis, particularly of the knee. There are many studies going on around the world to find and test new treatments for osteoarthritis. These include studies funded by Arthritis Research UK looking into the benefits of vitamin D (the VIDEO study) and a large national study to find the genes responsible for causing osteoarthritis (the arcOGEN study) which could lead to new therapies. Arthritis Research UK are also funding early trials of stem cell research, which aims to regenerate cartilage using the body's own cells.

Research has already shown the importance of exercise and weight management in reducing the pain of osteoarthritis, particularly of the knee.

Glossary

Acupuncture – a method of obtaining pain relief which originated in China. Very fine needles are inserted, virtually painlessly, at a number of sites (called meridians) but not necessarily at the painful area. Pain relief is obtained by interfering with pain signals to the brain and by causing the release of natural painkillers (called endorphins).

Aerobic exercise – any exercise that increases your pulse rate and makes you a bit short of breath.

Analgesics – painkillers. As well as dulling pain they lower raised body temperature, and most of them reduce inflammation.

Bunion – a bony lump on the side of the big toe caused by hallux valgus. Sometimes a swelling or bursa on the foot is also called a bunion.

Cartilage – a layer of tough, slippery tissue that covers the ends of the bones in a joint. It acts as a shock absorber and allows smooth movement between bones.

Chiropractor – a trained specialist who treats mechanical disorders of the musculoskeletal system, often through spine manipulation or adjustment. The General Chiropractic Council regulates the practice of chiropractic in the UK.

Collagen – the main substance in the white, fibrous connective tissue that's found in tendons, ligaments and cartilage. This very important protein is also found in skin and bone.

Gout – an inflammatory arthritis caused by a reaction to the formation of urate crystals in the joint. Gout comes and goes in severe flare-ups at first, but if not treated it can eventually lead to joint damage. It often affects the big toe.

Hallux rigidus – osteoarthritis of the big toe joint with a stiff, often painful, big toe.

Hallux valgus – a condition in which the big toe pushes across towards the other toes. It can cause deformities such as bunions and hammer toes.

Hydrotherapy – exercises that take place in water (usually a warm, shallow swimming pool or a special hydrotherapy bath) which can improve mobility, help relieve discomfort and promote recovery from injury.

Inflammation – a normal reaction to injury or infection of living tissues. The flow of blood increases, resulting in heat and redness in the affected tissues, and fluid and cells leak into the tissue, causing swelling.

Ligaments – tough, fibrous bands anchoring the bones on either side of a joint and holding the joint together. In the spine they're attached to the vertebrae and restrict spinal movements, therefore giving stability to the back.

Magnetic resonance imaging (MRI) scan – a type of scan that uses high-frequency radio waves in a strong magnetic field to build up pictures of the inside of the body. It works by detecting water molecules in the body's tissue that give

out a characteristic signal in the magnetic field. An MRI scan can show up soft-tissue structures as well as bones.

Manipulation – a type of manual therapy used to adjust parts of the body, joints and muscles to treat stiffness and deformity. It's commonly used in physiotherapy, chiropractic, osteopathy and orthopaedics.

Menisci (singular meniscus) – rings of cartilage, like washers, lying between the cartilage-covered bones in the knee. They act as shock absorbers and help the movement of the joint. Each knee has an inside (medial) and an outside (lateral) meniscus.

Menopause – the time when menstruation ends, usually when a woman is in her 50s. This means the ovaries stop releasing eggs every four weeks, and it's no longer possible to have children. If this happens before the age of 45, it's known as premature menopause.

Nodal osteoarthritis – a form of osteoarthritis that often runs in families, characterised by knobbly finger swellings (Heberden's nodes) and a tendency to get osteoarthritis in several joints (especially knees, big toes).

Non-steroidal anti-inflammatory drugs (NSAIDs) – a large family of drugs prescribed for different kinds of arthritis that reduce inflammation and control pain, swelling and stiffness. Common examples include ibuprofen, naproxen and diclofenac.

Occupational therapist – a trained specialist who uses a range of strategies and specialist equipment to help people to reach their goals and maintain their independence by giving practical advice on equipment, adaptations or by changing the way you do things (such as learning to dress using one handed methods following hand surgery).

Osteopath – a trained specialist who treats spinal and other joint problems by manipulating the muscles and joints in order to reduce tension and stiffness, and so help the spine to move more freely. The General Osteopathic Council regulates the practice of osteopathy in the UK.

Osteophytes – an overgrowth of new bone around the edges of osteoarthritic joints. Spurs of new bone can alter the shape of the joint and may press on nearby nerves.

Perthes' disease – inflammation at the head of the thigh bone (femur) that causes pain and limping, usually in boys aged 5–10 years. It can restrict blood supply to the bone leading to poor growth and deformity and can cause osteoarthritis to develop in later life.

Physiotherapist – a trained specialist who helps to keep your joints and muscles moving, helps ease pain and keeps you mobile.

Proton pump inhibitor (PPI) – a drug that acts on an enzyme in the cells of the stomach to reduce the secretion of gastric acid. They're often prescribed along with non-steroidal anti-inflammatory drugs (NSAIDs) to reduce side-effects from the NSAIDs.

Referred pain – pain that occurs in a different part of the body from that affected by injury or disease (for example, pain in the thigh or knee resulting from osteoarthritis of the hip). This is sometimes called radiated pain.

Rheumatoid arthritis – a common inflammatory disease affecting the joints, particularly the lining of the joint. It most commonly starts in the smaller joints in a symmetrical pattern – that is, for example, in both hands or both wrists at once.

Spondylosis – the term used to describe mechanical or degenerative changes in the small joints in the neck and back. Most of us will have some degeneration in these joints, which can be seen on x-rays, although often these changes don't cause any problems or symptoms.

Synovium – the inner membrane of the joint capsule that produces synovial fluid.

Transcutaneous electrical nerve stimulation (TENS) – a small battery-driven machine which can help to relieve pain. Small pads are applied over the painful area and low-voltage electrical stimulation produces a pleasant tingling sensation, which relieves pain by interfering with pain signals to the brain.

Where can I find out more?

If you've found this information useful you might be interested in these other titles from our range:

Conditions

- *Back pain*
- *Calcium crystal diseases including acute CPP crystal arthritis (pseudogout) and acute calcific tendinitis*
- *Gout*
- *Neck pain*
- *Osteoarthritis of the knee*

Therapies

- *Hydrotherapy and arthritis*
- *Occupational therapy and arthritis*
- *Physiotherapy and arthritis*

Self-help and daily living

- *Complementary and alternative medicine for arthritis*
- *Complementary and alternative medicines for the treatment of rheumatoid arthritis, osteoarthritis and fibromyalgia (63-page special report)*
- *Diet and arthritis*
- *Fatigue and arthritis*
- *Feet, footwear and arthritis*
- *Gardening and arthritis*
- *Keep moving*
- *Living with long-term pain: a guide to self-management*
- *Looking after your joints when you have arthritis*

Arthritis Research UK
Osteoarthritis

- *Pain and arthritis*
- *Practitioner-based complementary and alternative therapies for the treatment of rheumatoid arthritis, osteoarthritis, fibromyalgia, and low back pain (66-page special report)*
- *Sex and arthritis*
- *Sleep and arthritis*
- *What is arthritis?*
- *Work and arthritis*

Surgery

- *Foot and ankle surgery*
- *Hand and wrist surgery*
- *Hip replacement surgery*
- *Knee replacement surgery*
- *Shoulder and elbow joint replacement*

Drug leaflets

- *Painkillers*
- *Local steroid injections*
- *Non-steroidal anti-inflammatory drugs*

You can download all of our booklets and leaflets from our website or order them by contacting:

Arthritis Research UK
Copeman House
St Mary's Court
St Mary's Gate
Chesterfield
Derbyshire S41 7TD
Phone: 0300 790 0400
www.arthritisresearchuk.org

Osteoarthritis guidelines

The National Institute for Health and Clinical Excellence (NICE) issued guidelines to GPs in 2008 on how to best treat osteoarthritis based on available evidence.

The NICE guidance on osteoarthritis is available at www.nice.org.uk/CG59.

Printed copies of the NICE osteoarthritis patient guide can be ordered from 0845 003 7783 or at emailpublications@nice.org.uk quoting reference N1460.

Related organisations

The following organisations may be able to provide additional advice and information:

Arthritis Care
Floor 4, Linen Court
10 East Road
London N1 6AD
Phone: 020 7380 6500
Helpline: 0808 800 4050
Email: info@arthritiscare.org.uk
www.arthritiscare.org.uk

Arthritis and Musculoskeletal Alliance (ARMA)
Bride House
18–20 Bride Lane
London EC4Y 8EE
Phone: 020 7842 0910/11
Email: info@arma.uk.net
arma.uk.net

Adams J, *et al. BMJ Open* 2019; 9:e028342. doi: 10.1136/bmjopen-2018-028342

DIAL Network (formerly Disability Information and Advice Line or Dial UK)
Phone: 01302 310 123
www.scope.org.uk/dial
An independent network of local disability information and advice services run by and for disabled people, part of Scope.

Disabled Living Foundation
380–384 Harrow Road
London W9 2HU
Phone: 020 7289 6111
Helpline: 0845 130 9177
Email: helpline@dlf.org.uk
www.dlf.org.uk

General Chiropractic Council
44 Wicklow Street
London WC1X 9HL
Phone: 020 7713 5155
www.gcc-uk.org

General Osteopathic Council
176 Tower Bridge Road
London SE1 3LU
Phone: 020 7357 6655
www.osteopathy.org.uk

Links to sites and resources provided by third parties are provided for your general information only. We have no control over the contents of those sites or resources and we give no warranty about their accuracy or suitability. You should always consult with you GP or other medical professional.

Adams J, *et al. BMJ Open* 2019; 9:e028342. doi: 10.1136/bmjopen-2018-028342

Notes

Adams J, *et al. BMJ Open* 2019; 9:e028342. doi: 10.1136/bmjopen-2018-028342

Notes

Notes

We're here to help

Arthritis Research UK is the charity leading the fight against arthritis.

We're the UK's fourth largest medical research charity and fund scientific and medical research into all types of arthritis and musculoskeletal conditions.

We're working to take the pain away for sufferers with all forms of arthritis and helping people to remain active. We'll do this by funding high-quality research, providing information and campaigning.

Everything we do is underpinned by research.

We publish over 60 information booklets which help people affected by arthritis to understand more about the condition, its treatment, therapies and how to help themselves.

We also produce a range of separate leaflets on many of the drugs used for arthritis and related conditions. We recommend that you read the relevant leaflet for more detailed information about your medication.

Please also let us know if you'd like to receive our quarterly magazine, *Arthritis Today*, which keeps you up to date with current research and education news, highlighting key projects that we're funding and giving insight into the latest treatment and self-help available.

We often feature case studies and have regular columns for questions and answers, as well as readers' hints and tips for managing arthritis.

Tell us what you think

Please send your views to:
feedback@arthritisresearchuk.org
or write to us at:
Arthritis Research UK, Copeman House, St Mary's Court, St Mary's Gate, Chesterfield, Derbyshire S41 7TQ.

A team of people contributed to this booklet. The original text was written by Prof. Mike Doherty, who has expertise in the subject. It was assessed at draft stage by consultant rheumatology nurse Diana Finney and consultant physiotherapist Kay Stevenson. An **Arthritis Research UK** editor revised the text to make it easy to read, and a non-medical panel, including interested societies, checked it for understanding. An **Arthritis Research UK** medical advisor, Prof. Anisur Rahman, is responsible for the content overall.

Arthritis Research UK

Get involved

You can help to take the pain away from millions of people in the UK by:

- volunteering
- supporting our campaigns
- taking part in a fundraising event
- making a donation
- asking your company to support us
- buying products from our online and high-street shops.

To get more **actively involved,** please call us on **0300 790 0400**, email us at **enquiries@arthritisresearchuk.org** or go to **www.arthritisresearchuk.org**

Arthritis Research UK

Arthritis Research UK
Copeman House
St Mary's Court
St Mary's Gate, Chesterfield
Derbyshire S41 7TD

Tel **0300 790 0400**
calls charged at standard rate

www.arthritisresearchuk.org

Registered Charity No 207711
© Arthritis Research UK 2012
Published November 2012 2025/OA/12-1

Arthritis Research UK

Keeping active with osteoarthritis

It's important to stay active when you have osteoarthritis – exercising will help ease stiffness and stop your muscles becoming weak. As well as the simple exercises in this pull-out, you should choose a form of exercise you enjoy and stick at it.

Swimming, walking, yoga and Pilates are all great options if you have osteoarthritis.

www.arthritisresearchuk.org

Exercises for osteoarthritis

This handy tear-off section contains exercises that are designed to stretch, strengthen and stabilise the structures that support your joints.

Exercises for osteoarthritis

The following exercises are designed to stretch, strengthen and stabilise the structures that support your joints.

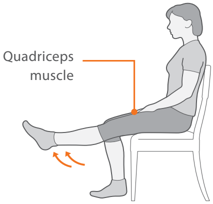

1

Straight-leg raise (sitting): Get into the habit of doing this every time you sit down. Sit well back in the chair with a good posture. Straighten and raise one leg. Hold for a slow count to 10 then slowly lower your leg. Repeat this at least 10 times with each leg. If you can do this easily, try it with light ankle weights and with your toes pointing towards you.

2

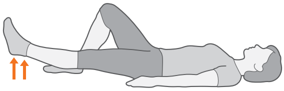

Straight-leg raise (lying): Get into the habit of doing this in the morning and at night while lying in bed. Bend one leg at the knee. Hold your other leg straight and lift your foot just off the bed. Hold for a slow count of five then lower. Repeat five times with each leg every morning and evening.

3

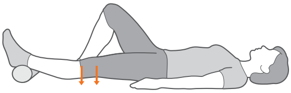

Muscle stretch: Do this at least once a day when lying down. Not only does this exercise help to strengthen the quadriceps muscles, but it also prevents the knee from becoming permanently bent. Place a rolled-up towel under the ankle of the leg to be exercised. Bend the other leg at the knee. Use the muscles of your straight leg to push the back of the knee firmly towards the bed or the floor. Hold for a slow count of five. Repeat at least five times with each leg.

4

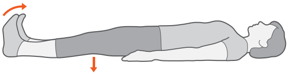

Quad exercise: Pull your toes and ankles towards you, while keeping your leg straight and pushing your knee firmly against the floor. Hold for five seconds and relax. Repeat five times.

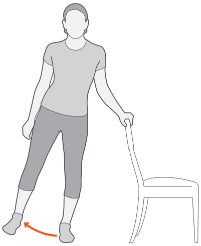

5

Hip abduction: Lift your leg sideways, being careful not to rotate the leg outwards. Hold for five seconds and bring it back slowly, keeping your body straight throughout. Repeat five times on each side. Hold onto a chair or work surface for support.

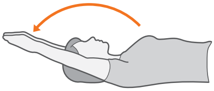

7

Arm stretch (lying): Lie on your back with your arms by your sides. Raise your arms overhead as far as you can and hold for 5–10 seconds. Return your arms to your sides and repeat five times.

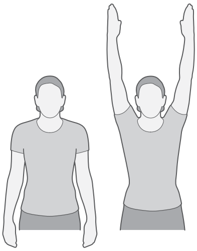

6

Arm stretch (standing): Stand with your arms relaxed at your sides. Raise your arms as far as you can and hold for 5–10 seconds. Lower and repeat five times.

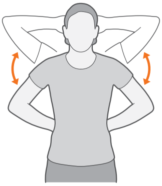

8

Arm lifts: Place your hands behind your head so your elbows are pointing to the sides. Hold for five seconds then place your hands behind your back, keeping your elbows out. Hold for five seconds. Do five sets.
