# Supplementary material for: The Osteoarthritis Thumb Therapy (OTTER) II Trial: a study protocol for a three-arm multi-centre randomised placebo controlled trial of the clinical effectiveness and efficacy and cost-effectiveness of splints for symptomatic thumb base osteoarthritis
Source: BMJ Open. 2019 Oct 22;9(10):e028342. doi: 10.1136/bmjopen-2018-028342 (PMC6830636; doi:10.1136/bmjopen-2018-028342)
Supplement: Supplementary data [file bmjopen-2018-028342supp003.pdf]

Osteoarthritis Thumb Base Therapy Trial

OTTER trial

Thumb Base Pain Exercise Booklet

Information and an exercise programme specifically for people with thumb base pain

OTTER II trial –IRAS 198227

REC ref: 16/SC/0188

OTTER_ExerciseBooklet_V2.0_13Oct2016

Thumb Base Pain

You have been given this booklet because you have pain or discomfort in your thumb joint.

Pain at the base of your thumb can be due to a number of reasons, one of which can be thumb base Osteoarthritis. Pain at the base of your thumb can affect many aspects of your everyday life. This booklet has been designed with the help of clinicians and patients who have pain at the base of the thumb. It describes a common cause of pain in this area, Osteoarthritis.

This booklet focuses specifically on thumb base pain and provides a programme of exercise devised with the help of national and international therapy clinicians and is supported by published research. You have also been given a Joint Protection Booklet which discusses Osteoarthritis generally, and presents ways to enable you to carry on your normal life as much as possible whilst reducing pain and protecting your joints from further damage.

This booklet contains four main sections:

1. Causes of Thumb Osteoarthritis
2. Symptoms of Thumb Osteoarthritis
3. Treatment of Thumb Osteoarthritis
4. Hand Exercises

Adams J, *et al. BMJ Open* 2019; 9:e028342. doi: 10.1136/bmjopen-2018-028342

OTTER trial

1. Causes of Thumb Osteoarthritis

Osteoarthritis in the carpometacarpal joint (CMC joint) at the base of the thumb is the most common cause of pain in this area.

The CMC joint is formed where the metacarpal bone of the thumb meets the trapezium bone of the wrist.

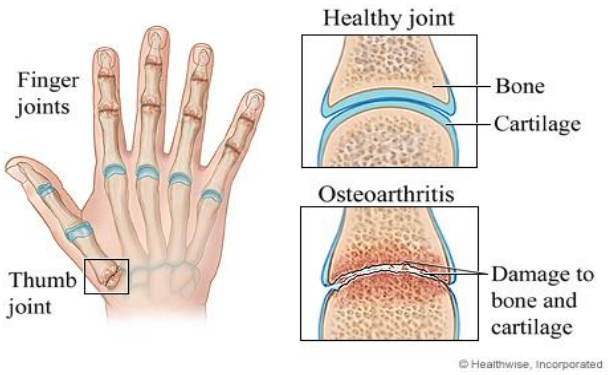

Due to the movement required at the base of the thumb, you rely on your ligaments and bony structures to maintain stability. Damage or overuse can place high loads through the base of the thumb and lead to degeneration. A good point to remember is that any pressures placed through the tip of the thumb during pinching activities are multiplied by around twelve times through the CMC joint.

There are many factors that can lead to pain in the base of the thumb or thumb base Osteoarthritis including a previous injury, repetitive activity of the thumb joint, it can be inherited, and can be affected by gender – women are more likely to have Osteoarthritis than men.

2. Symptoms of Thumb Osteoarthritis

Pain is the primary symptom associated with thumb Osteoarthritis. Initially, pain is present with movement or activity, for example, turning a key, opening a door, lifting a cup. If the Osteoarthritis progresses, pain may be present even during inactivity or rest.

Other symptoms of thumb arthritis include:

- Difficulty gripping objects;
- Swelling, stiffness, or tenderness at the base of the thumb;
- Enlarged appearance and altered posture of the CMC joint;
- Limited range of motion.

3. Treatment of Thumb Osteoarthritis

Early Osteoarthritis of the thumb can be effectively managed using non-surgical treatment options. These treatments aim to reduce the pain caused by wear and repair of the joint:

- Some medicines can help to reduce inflammation, swelling and pain. You should discuss this with your GP.
- Steroid injections can be given into the joint.
- Self-help measures such as joint protection, diet, complementary therapies etc.

If you have worsening symptoms, you should discuss other options with your GP or consultant.

Additional information can be obtained from the Arthritis Research UK website (http://www.arthritisresearchuk.org/).

We acknowledge the input to this booklet of Sarah Bradley and Kirsty Bancroft of Poole NHS Trust and Prof Ingvild Kjeken from the Norwegian National Advisory Unit on Rehabilitation in Rheumatology.

4. Hand Exercises

Exercise has many benefits. It can help to ease stiffness, improve movement in your joints and strengthen muscles.

These hand exercises are for you as part of the OTTER II trial. They should benefit you if you have pain at the base of your thumb. These exercises can reduce thumb pain and stiff joints in your hand and strengthen your thumb muscles.

- To achieve the best results **repeat** these exercises at least **3 times a week for at least 20 minutes each time**.
- It is not unusual to experience some slight discomfort or pain in your thumb after doing these exercises. Any discomfort should stop after 24 hours. If you experience discomfort in your thumb or hand when doing these exercises that does not start to feel better after 24 hours, please contact your therapist.
- Always start with the Warm-Up Exercise. To warm up your hand place your hand in a bowl of warm water and gently move your thumb in a circular direction. After one minute you can then change the direction. Carry out these gentle moves for at least 2 minutes. This will help ease your hand and thumb into exercising.
- Follow the Warm-Up Exercise with Level 1 Exercises. When you can easily do Level 1 Exercises then you can go straight from the warm up to Level 2 Exercises. When you can easily do Level 2 Exercises then you can go straight from the warm up to Level 3 Exercises.
- When you do each exercise look carefully at the joints of your thumb. Make sure that your thumb does not bend backwards (or hyperextend) at the joints. Try to make sure that your thumb joints are kept a little bent (or flexed) as you do each exercise, as this will help protect your thumb.

Level 1 Exercises

Exercise 1

Step 1

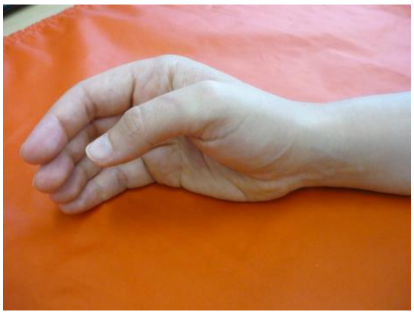

Rest your arm and hand on a table on the little finger side so that your thumb is on top.

Step 2

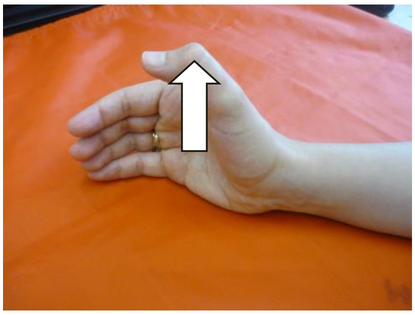

Without help from the other hand lift your thumb upwards as far as possible.
Hold for 10 seconds and repeat up to 10 times.
Make sure that your thumb joints **keep bent** (flexed) when you do this exercise.
Try **not to over extend** any of the thumb joints.

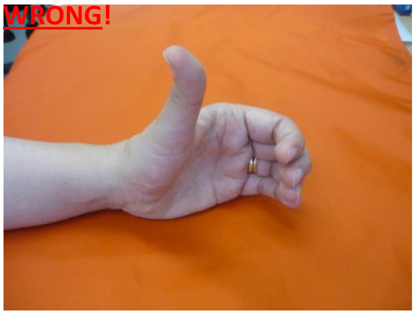

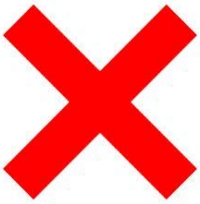

Try **not to over extend** the joints of your thumb. This is **not the right way** to do this exercise.
If your thumb looks like the picture on the left, try the exercise again making sure to **keep your thumb joints bent** (flexed).

<u>Exercise 2</u>

Step 1

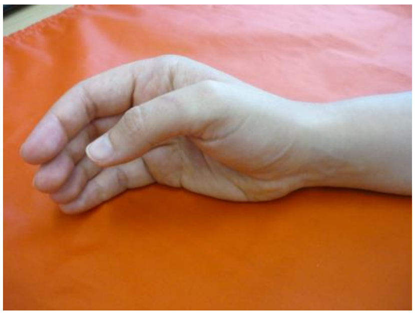

Rest your arm and hand on a table on the little finger side so that your thumb is on top.

Step 2

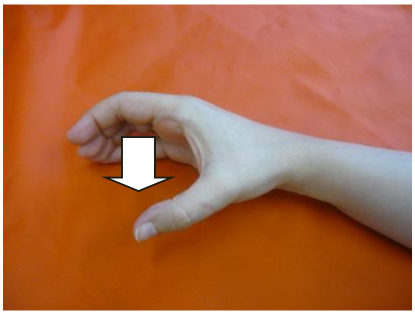

Keeping both joints of the thumb **slightly bent**, keep your thumb as far away from your palm as possible while lifting your thumb up toward the ceiling.
Hold for 10 seconds and repeat up to 10 times.

Adams J, *et al. BMJ Open* 2019; 9:e028342. doi: 10.1136/bmjopen-2018-028342

WRONG!

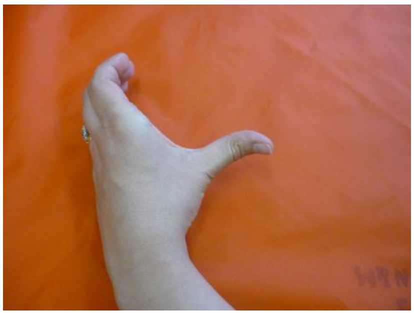

Try **not to bend** the joints of the thumb back.
This is the **wrong way** to do this exercise.

Exercise 3

Place your elbow or back of your hand on a table.
Put your thumb against each fingertip in turn making an 'O' shape.
Make sure your thumb joints are **always slightly bent** (flexed).

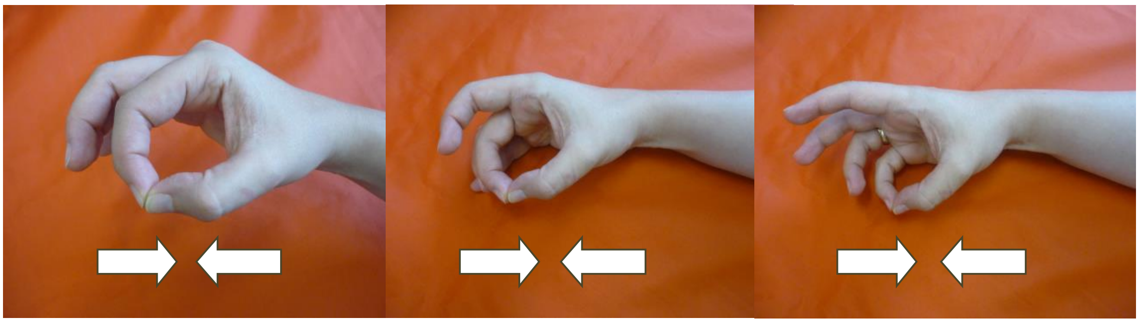

WRONG!

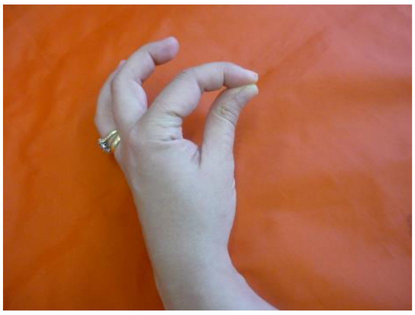

Try **not to bend** the joints of the thumb back.
This is the **wrong way** to do this exercise.

If you can complete Level 1 Exercises easily then move onto Level 2 Exercises. If you cannot complete Level 1 Exercises easily then please do not start Level 2 Exercises

Adams J, *et al. BMJ Open* 2019; 9:e028342. doi: 10.1136/bmjopen-2018-028342

Level 2 Exercises

Exercise 1

First carry out the **Warm-Up Exercise gently moving your thumb in warm water.**

Perform the rest of the Level 2 Exercises with the strongest rubber band that you can comfortably stretch around your thumb and palm.

Exercise 2

Step 1

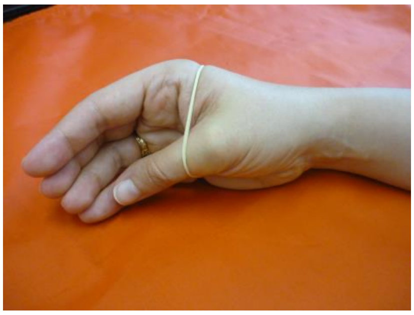

Start with your hand relaxed and a rubber band placed around your hand crossing around the middle of your thumb as in the picture on the left.

Step 2

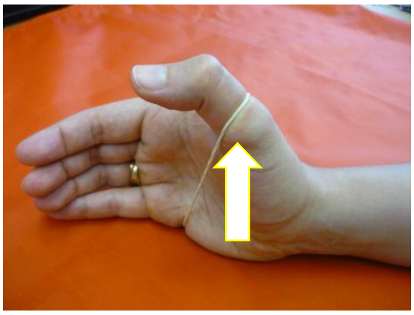

Stretch the band slowly by lifting the thumb upwards as far as possible while keeping the two joints of the thumb slightly bent.

Try to ensure that the wrist **does not bend** forwards during the movement.

Hold for 5 seconds then lower slowly and repeat up to 10 times.

When you can perform this exercise easily, **change** to a stronger band.

WRONG!

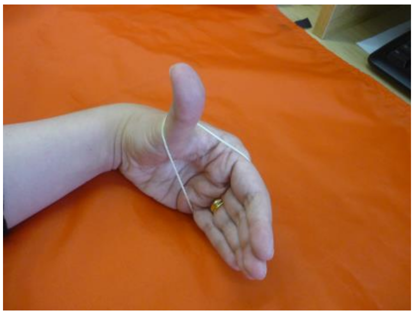

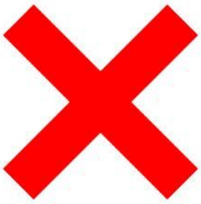

Try **not to bend** the thumb joints back or bring the wrist forward.

This is the **wrong way** to do this exercise.

OTTER trial

Exercise 3

Step 1

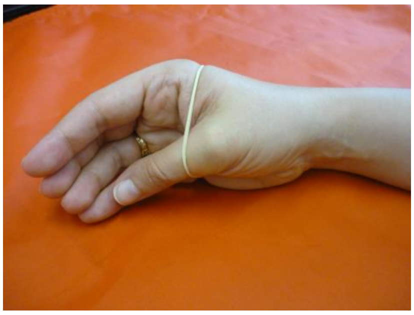

Start with your hand relaxed and a rubber band placed around your hand crossing around the middle of your thumb as in the picture on the left.

Step 2

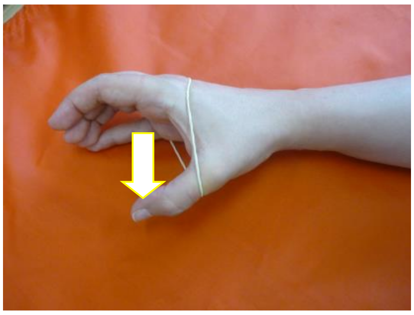

Keep the top **joints of the thumb bent** and lift your thumb up towards your **opposite shoulder** while keeping the thumb as **far away** from the palm as possible.

Step 3

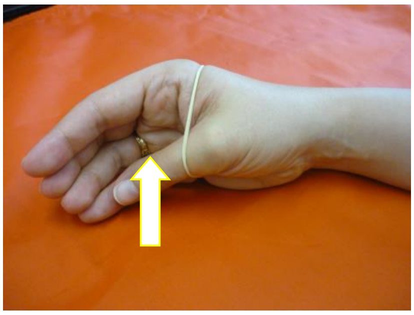

Relax the thumb back to the start position (Step 1) by slowly **lowering the thumb** while keeping both thumb joints a **little bent**.

Try to control this movement and **not let** the band slacken too quickly.

Hold for 5 seconds and repeat up to 10 times.

If you can complete Level 2 Exercises easily then move onto Level 3 Exercises. If you cannot complete these level exercises easily then stop at this level.

Adams J, *et al. BMJ Open* 2019; 9:e028342. doi: 10.1136/bmjopen-2018-028342

Level 3 Exercises

Exercise 1

First carry out the **Warm-Up Exercise**.

Exercise 2: Pinch tasks

Practice tasks where you use your thumb to pinch, for example, writing, holding plates, opening clothes pegs, tearing sheets of paper.

When you do these tasks continue to **keep both joints of the thumb slightly bent**, and the **wrist slightly extended**.

Take time to **look at your thumb** and make an effort to **keep your thumb joints in line**.
Try **not to let them bend backwards**.

The following pictures show the correct and the wrong way to do Pinch task 1.

Pinch Task 1

Practice writing your name and address using the correct thumb position 5 times

CORRECT!

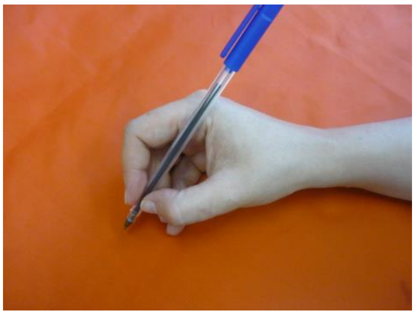

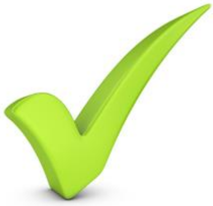

WRONG!

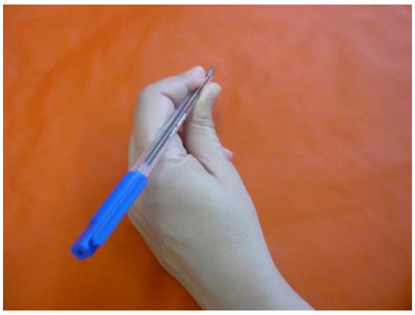

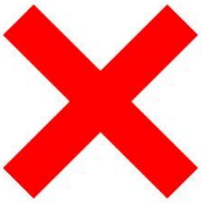

The following pictures show the correct and the wrong way to do Pinch Task 2

<u>Pinch Task 2</u>

Practice tearing a piece of paper in half using the correct thumb position 20 times

CORRECT!

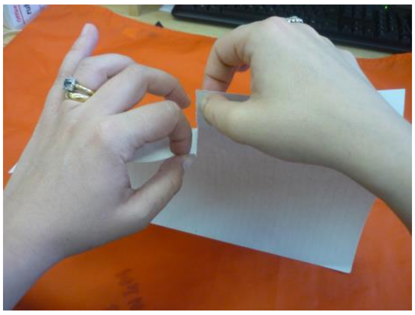

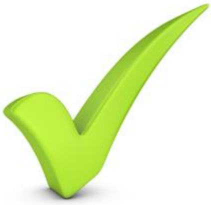

WRONG!

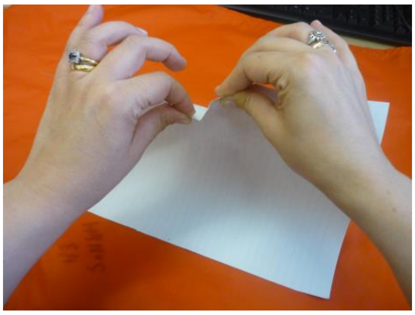

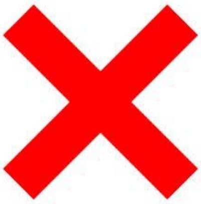

The following pictures show the correct and the wrong way to do Pinch Task 3

<u>Pinch Task 3</u>

Practice holding a clothes peg or a small document clip and squeezing this using the correct thumb position 5 times

CORRECT!

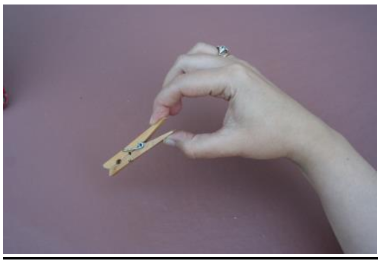

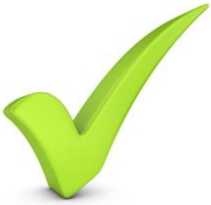

WRONG!

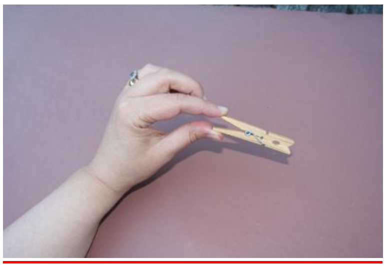

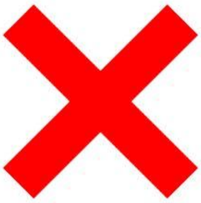

Adams J, *et al*. *BMJ Open* 2019; 9:e028342. doi: 10.1136/bmjopen-2018-028342

The following pictures show the correct and the wrong way to do Pinch Task 4

Pinch Task 4

Practice picking up and holding a serving plate using the correct thumb position for 5 times

CORRECT!

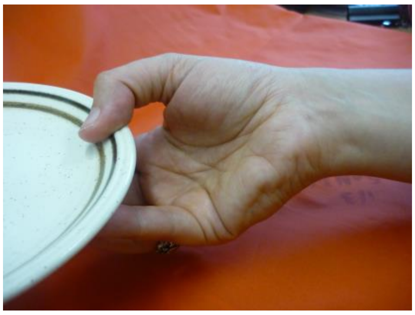

WRONG!

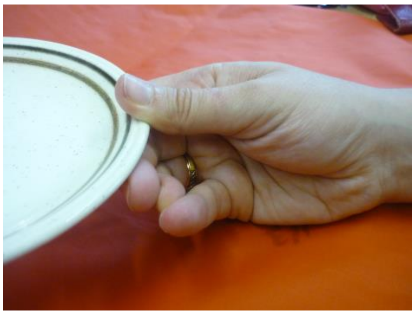

Exercise 3: Grip and turn Tasks

Practice activities which involve turning or twisting, for example, putting nuts on bolts, turning keys in locks, undoing jar tops, turning taps.

During these activities work to maintain a **slight bend (flexion) of both thumb joints** and **avoid the thumb crossing in front of the palm**.

The following pictures show the correct and the wrong way to do Grip and Turn Task 1.

Grip and Turn Task 1

Practice turning a key as if unlocking a door using the correct thumb position 10 times

CORRECT!

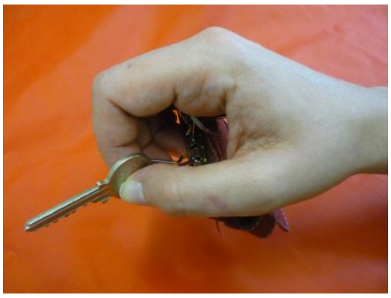

WRONG!

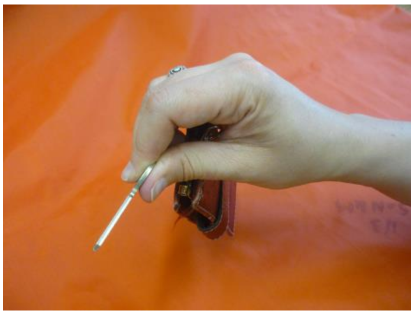

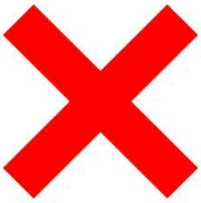

Grip and Turn Task 2

Practice unscrewing and screwing up a bottle top using the correct thumb position five times

CORRECT!

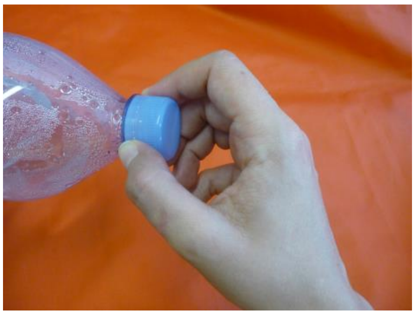

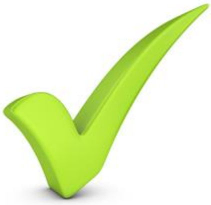

WRONG!

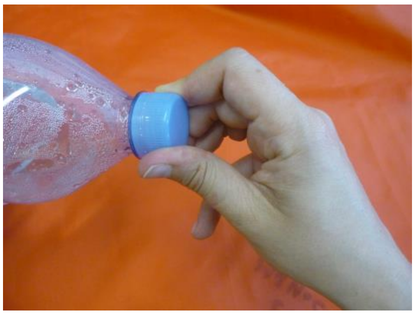

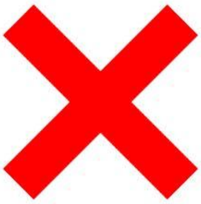

This is the end of the OTTER thumb exercises

If you need to get in touch with the OTTER Trial Research Team, please contact your local occupational therapist or physiotherapist

Adams J, *et al. BMJ Open* 2019; 9:e028342. doi: 10.1136/bmjopen-2018-028342
