# Supplementary material for: The Osteoarthritis Thumb Therapy (OTTER) II Trial: a study protocol for a three-arm multi-centre randomised placebo controlled trial of the clinical effectiveness and efficacy and cost-effectiveness of splints for symptomatic thumb base osteoarthritis
Source: BMJ Open. 2019 Oct 22;9(10):e028342. doi: 10.1136/bmjopen-2018-028342 (PMC6830636; doi:10.1136/bmjopen-2018-028342)
Supplement: Author's manuscript [file bmjopen-2018-028342.draft_revisions.pdf]

BMJ Open is committed to open peer review. As part of this commitment we make the peer review history of every article we publish publicly available.

When an article is published we post the peer reviewers' comments and the authors' responses online. We also post the versions of the paper that were used during peer review. These are the versions that the peer review comments apply to.

The versions of the paper that follow are the versions that were submitted during the peer review process. They are not the versions of record or the final published versions. They should not be cited or distributed as the published version of this manuscript.

BMJ Open is an open access journal and the full, final, typeset and author-corrected version of record of the manuscript is available on our site with no access controls, subscription charges or pay-per-view fees (http://bmjopen.bmj.com).

If you have any questions on BMJ Open's open peer review process please email info.bmjopen@bmj.com

BMJ Open

The Osteoarthritis Thumb Therapy Trial (OTTER II): A study protocol for a three arm multi centre randomised placebo controlled trial into the clinical effectiveness and efficacy of splints for symptomatic thumb base osteoarthritis

Journal:	*BMJ Open*
Manuscript ID	bmjopen-2018-028342
Article Type:	Protocol
Date Submitted by the Author:	12-Dec-2018

Complete List of Authors:	Adams, Joanna; University of Southampton, Faculty of Environment and Life Sciences<br>Barratt, Paula ; University of Southampton, Faculty of Environment and Life Sciences<br>Arden, Nigel; Nuffield Department of Orthopaedics, ;<br>Barbosa Bouças, Sofia; Brunel University , Department of Life Sciences<br>Bradley, Sarah; Poole Hospital NHS Foundation Trust<br>Doherty, Michael ; University of Nottingham<br>Dutton, Susan; University of Oxford, CSM<br>Dziedzic, Krysia; Arthritis Research Campaign National Primary Care Centre<br>Gooberman-Hill, Rachael; University of Bristol, Bristol Medical School<br>Hislop Lennie, Kelly; University of Southampton, Faculty of Environment and Life Sciences<br>Hutt Greenyer , Corinne; University of Southampton, Faculty of Environment and Life Sciences<br>Jansen , Victoria; Royal Derby Hospital, Pulvertaft Hand Centre<br>Luengo-Fernandez, Ramon; University of Oxford, Health Economics Research Centre<br>Meagher, Claire ; University of Southampton, Faculty of Environment and Life Sciences<br>White, Peter; University of Southampton, Faculty of Environment and Life Sciences<br>Williams, Mark; Oxford Brookes University, Sport, Health Sciences and Social Work

Keywords:	Osteoarthritis, REHABILITATION MEDICINE, RHEUMATOLOGY, Efficacy trial

SCHOLARONE™
Manuscripts

The Osteoarthritis Thumb Therapy Trial (OTTER II): A study protocol for a three arm multi centre randomised placebo controlled trial into the clinical effectiveness and efficacy of splints for symptomatic thumb base osteoarthritis

Corresponding author: Jo Adams. School of Health Sciences, Faculty of the Environement and Life Sciecnes, Building 67, University of Southampton, Highfield Campus, University Road, Southampton, SO17 1BJ, UK. Tel: + 44 (0)23 8059 5287. E-mail: ja@soton.ac.uk

Authors:

Jo Adams School of Health Sciences, University of Southampton, Southampton, UK

Paula Barratt School of Health Sciences, University of Southampton, Southampton, UK

Nigel Arden Nuffield Department of Orthopaedics, Rheumatology and Musculoskeletal Sciences (NDORMS), University of Oxford, Oxford, UK

Sofia Barbosa Bouças Dept of Life Sciences, Brunel University London, Uxbridge, UK

Sarah Bradley Hand Therapy Unit, Poole Hospital NHS Trust, Poole, UK

Michael Doherty School of Medicine, University of Nottingham, Nottingham, UK

Susan Dutton Oxford Clinical Trials Research Unit, University of Oxford, Oxford, UK

Krysia Dziedzic Research Institute for Primary Care and Health Sciences, Keele University, Keele, UK

Rachael Gooberman-Hill Elizabeth Blackwell Institute for Health Research, University of Bristol, Bristol, UK

Kelly Hislop School of Health Sciences, University of Southampton, Southampton, UK

Corinne Hutt Greenyer School of Health Sciences, University of Southampton, Southampton, UK

Victoria Jansen Pulvertaft Hand Centre, Royal Derby Hospital, Derby, UK

Ramon Luengo Fernandez Nuffield Dept of Population Health, University of Oxford, Oxford, UK

Claire Meagher School of Health Sciences, University of Southampton, Southampton, UK

Peter White School of Health Sciences, University of Southampton, Southampton, UK

Mark Williams Dept of Sport, Health Sciences and Social Work, Oxford Brookes University, Oxford, UK

On behalf of the OTTER II collaborative group

Word count: 4467 (excl abstract and tables)

For peer review only - http://bmjopen.bmj.com/site/about/guidelines.xhtml

Abstract

Introduction

The economic cost of osteoarthritis (OA) is high. At least 4.4 million people have hand OA in the UK. Symptomatic thumb base OA affects 20% of the population over 55 years, causing more pain, work disability, reduced quality of life and function than OA elsewhere in the hand. Most evidence-based guidelines recommend splinting for hand OA, and splints that support or immobilise the thumb base are routinely used to reduce hand pain and improve hand function despite there being limited evidence to support this. The potential effects of placebo interventions in OA are acknowledged, but few studies investigate the clinical efficacy of rehabilitation interventions nor the impact of any placebo/contextual effects associated with splints in patients with hand OA.

Methods and Analysis

Participants aged 30 years and over with symptomatic thumb base OA will be recruited into the trial from secondary care occupational therapy and physiotherapy centres. Following informed consent, participants will complete a baseline questionnaire and then be randomised into one of three treatment arms: a self-management programme alone, a self-management programme plus a verum thumb splint, or a self-management programme plus a placebo thumb splint. The primary outcome is the AUSCAN hand pain scale. The primary study end point is 8 weeks after baseline. Baseline assessments will be carried out prior to randomisation and outcomes will be collected at 4 weeks; 8 weeks and 12 weeks. Individual qualitative interviews will be conducted with up to 40 participants after 8 weeks to explore perceptions and outcome expectations of verum and placebo splints and exercise.

Ethics and Dissemination

South Central - Oxford C Research Ethics Committee approved this study (16/SC/0188). The findings should contribute to improving the management of thumb base OA and help clinicians and patients make informed decisions about the value of different interventions.

Trial Registration Number: ISRCTN 54744256

For peer review only - http://bmjopen.bmj.com/site/about/guidelines.xhtml

Key words: Osteoarthritis; thumb base; splint; hand pain; hand function; placebo; self-management.

Strengths and Limitations

1. This trial is powered to evaluate the clinical benefit and statistical significance of adding splinting to a self-management programme for people with thumb base OA.

2. This is the first trial to use a placebo thumb splint intervention and will add to the understanding of contextual aspects of such visible physical treatment and self-management programmes for OA.

3. The trial has been informed and designed with the input of patients and expert clinicians and the trial outcome measures have been agreed as meaningful by patients.

4. The 12 week trial follow-up period is limited and longer term follow-up would provide further useful outcome data.

For peer review only

For peer review only - http://bmjopen.bmj.com/site/about/guidelines.xhtml

INTRODUCTION

Osteoarthritis (OA) is a leading cause of pain, disability, healthcare utilisation, and productivity loss in the UK. Each year approximately two million adults visit their GP with symptoms of OA [1]. The disease is more prevalent in women, the incidence increases with age and recent estimates suggest more than 150 million Europeans have radiographic hand OA and 15 million have symptomatic arthritis [2]. Symptomatic thumb base (1st carpo-metacarpal and/or scaphotrapezial joint) OA affects approximately 22% of people aged 50 years and over [3].Thumb base OA is likely to become more prevalent in the future since the incidence increases with age [4] and is identified as a priority for treatment as it causes more pain and disability and is associated with a worse prognosis than other hand sites affected by OA [5].

Patients with thumb base OA present with predominantly mechanical usage-related pain over the thumb base [6] and are more likely to have more persistent pain, work disability and reduction in quality of life and function, and to receive more anti-inflammatory drugs and more splinting than participants with OA affecting other hand joints [7]. Thumb base OA affects entire hand function [8] and the overall impact, particularly in older people, can be substantial, with many experiencing difficulties with daily household, caring, work and leisure activities [9]. Despite the scale of this problem, it appears that both patients and practitioners often believe that there is little that can be done [10].

Traditional therapeutic splinting for thumb base OA is based on biomechanical principles aimed at offloading the dynamic forces occurring during daily functional use on a symptomatic 1st carpometacarpal joint [11]. Many thumb splint designs will either immobilise or support the thumb base. Thumb splints are recommended for patients with thumb base OA [12]. Some evidence based on small, non-powered samples [13 14] show thumb splints can help relieve pain however, evidence to support their effectiveness is not yet fully supported by robust research [15]. There is insufficient evidence to suggest that the combination of splinting delivered alongside hand exercises is more effective than hand exercises alone [16-18]. To date, there has also been no examination of any

For peer review only - http://bmjopen.bmj.com/site/about/guidelines.xhtml

contextual and non-specific patient-practitioner interaction effects associated with splinting in thumb base OA. If this is substantial, optimising the non-specific effects of treatment could improve treatment effectiveness and overall care of people with OA in a safe and cost effective way [19 20]. This randomised controlled trial (RCT) protocol, was designed in accordance with the SPIRIT checklist [21], to evaluate the effectiveness, efficacy and cost effectiveness of thumb splints for people with thumb base OA.

The primary objective of this trial is to determine the clinical effectiveness and efficacy of thumb splints when added to a self-management programme for people with symptomatic thumb base OA. This trial questions:

1. Is there a benefit of adding a thumb base splint to a self-management programme for people with thumb base OA?

2. Is there a difference in benefit between adding a verum or a placebo thumb base splint to a self-management programme for people with thumb base OA?

3. What are patients' views and experiences of the effectiveness, acceptability and adherence to the trial interventions?

METHODS AND ANALYSIS

Patient and Public Involvement

Exploring the effectiveness of orthotic devices for use by patients living with osteoarthritis was identified as a priority area for investigation by an Arthritis Resaerch UK patient stakeholder committee. Two patient and public group meetings were carried out to inform the choice of meaningful trial outcome measures, intervention components and help to inform co-design a placebo splint design [22]. A national Delphi study with therapy clinicians and patients was conducted, to agree trial processes [23 24] and consider treatment burden from both clinical and patient's view points. We conducted a focus group to explore the support required by NHS therapy clinicians when knowingly delivering placebo splints was completed [25]. A pilot study across five NHS recruitment sites to test feasibility and safety and the convincing delivery of a newly designed placebo splint was conducted and reported [26]. The education and support needs identified by therapy clinicians taking

For peer review only - http://bmjopen.bmj.com/site/about/guidelines.xhtml

part in a placebo controlled RCT was sought and recorded [27]. This informed the trial training for clinicians. Our patient partner and co-author (CHG) provided a patient's perspective in developing recruitment strategies, study conduct and lay dissemination routes.

Trial Design and Setting

OTTER II is a pragmatic, multi-centred, single (participant) blind, superiority randomised controlled clinical effectiveness and efficacy trial. People with symptomatic thumb base OA reporting moderate to severe thumb base pain, will be equally allocated to one of three groups: Group A: Eight weeks of a self-management programme, Group B: Eight weeks of a self-management programme plus a verum splint, or Group C: Eight weeks of a self-management programme plus a placebo splint. Follow up for all groups will be for 12 weeks from baseline.

Participants will be recruited from referrals to Occupational Therapy or Physiotherapy (Therapy) departments from new, current and review patients of Rheumatology, Orthopaedic, Hand Surgery Units and General Practice at 17 NHS recruitment sites (Appendix 1).

Participants

Participants will be screened for recruitment into the trial using inclusion and exclusion criteria as shown in Table 1.

**Inclusion Criteria**	
1	Aged 30 years and over.
2	At least moderate hand pain (AUSCAN [28] hand pain score >5) and moderate functional hand disability (AUSCAN [28]hand functional disability score >9).
3	Show signs and symptoms of thumb base OA on clinical enquiry and examination, specifically: hard tissue enlargement of the first carpometacarpal joint (CMCJ) OR squaring of the thumb base OR pain that worsens when pinching OR pain that worsens on span grip (e.g. opening a jar) OR crepitus on movement OR reduction in thumb base range of movement OR positive thumb adduction provocation test [29] OR positive thumb extension provocation test [29] OR pain on palpation of the dorso-radial aspect of the thumb CMCJ.
4	No other household member participating in the trial.
5	Able to give written informed consent.

For peer review only - http://bmjopen.bmj.com/site/about/guidelines.xhtml

6	Available to attend Occupational Therapy/Physiotherapy/Hand Therapy sessions.
**Exclusion Criteria**	
**1**	Consultation with therapy department or treatment for this thumb problem (excluding pain killers and anti-inflammatories) in the previous six months.
**2**	Intra-articular joint injection to wrist, fingers or thumb in the previous two months.
**3**	Fractures or significant injury or surgery to the wrist or hand within the previous six months.
**4**	Red flags i.e. history of serious illness or disease. i.e. any other diagnosed rheumatic conditions: gout, psoriatic arthritis, ankylosing spondylitis, connective tissue disorders (systemic lupus, systemic sclerosis), resulting in inflammatory arthritis in the hand/s, or, progressive neurological signs, or acute swollen hand joint.
**5**	Diagnosis of dementia or other significant disorder likely to affect communication.
**6**	Already received thumb splints for thumb base OA.
**7**	Skin disease that may interfere or contraindicate splint wear.
**8**	Participant of a drug or medical device trial in the last 12 weeks.

Table 1. OTTER II Trial patient inclusion and exclusion criteria

Interventions

The trial's three intervention arms all include 90 minutes of direct therapy intervention with a qualified therapist experienced in treating patients with hand OA. This will be delivered in a 60-minute baseline appointment, when the interventions listed below will be delivered. A 30-minute follow up intervention at week four will be conducted, where progress is reviewed and any necessary adjustments made. A final third hospital visit at week eight is for finalisation of trial procedures only and includes no direct therapy intervention.

The three intervention arms, delivered at the baseline appointment are:

<u>Group A - An optimal self-management programme</u>

The self-management programme includes:

1. Teaching standardised evidence based hand exercises and provision of a trial-specific hand exercise booklet.

For peer review only - http://bmjopen.bmj.com/site/about/guidelines.xhtml

2. Provision of a trial-specific booklet about joint protection, activity pacing and general advice about OA followed by a discussion with the therapist of the content.

3. Provision of the Arthritis Research UK Osteoarthritis information booklet.

4. A discussion with the therapist of the facilitators and barriers to engaging with self-management principles.

5. A patient hand exercise diary.

<u>Group B - Optimal self-management programme plus a verum thumb base splint</u>

Group B participants receive:

1. The optimal self-management programme as detailed above in Group A.

2. A verum splint. Therapists and participants will be given an option of two different splints, with the choice guided by a standardised Splint Decision Protocol.

The splinting options informed by the study's design and development stage will be:

- Procool Thumb CMC Restriction splint (black) (Ref PTRS).

Or

- Orfilight 2.5mm 3/32" micro perforated (beige) trouser leg splint (custom made by the therapist from a pre-cut standardised trial template and standardised strapping protocol).

Both splints will be delivered with instructions on how to wear and use the splint. The Procool thumb CMC restriction splint comes in packaging with a label providing details of the manufacturer and washing instructions.

3. An information sheet to outline when the splint should be worn

4. A discussion with the therapist of the facilitators and barriers to engaging with splint wear.

5. A patient splint wear diary.

<u>Group C - Optimal self-management programme plus a placebo thumb base splint</u>

Group C participants receive:

1. The self-management programme as detailed above in Group A.

2. A placebo splint. There will be the choice of two designs of placebo splint. These are made in a lightweight nylon, secured around the wrist and have phalangeal components but no basal thumb

For peer review only - http://bmjopen.bmj.com/site/about/guidelines.xhtml

joint support. One is black and the other is beige to match the verum splint options and three sizes will be manfucatured. They have been designed with no known active component and none was detectable during testing [30]. A standardised Splint Decision Protocol about which splint will be most appropriate to issue in which situation will be used. The placebo splints arrive in packaging with a label providing details of the manufacturer, washing instructions, and a lifestyle education leaflet about how to position and wear the splint. Both splints will be delivered with instructions on how to wear and use the splint.

3. An information sheet to outline when the splint should be worn
4. A discussion with the therapist of the facilitators and barriers to engaging with splint wear.
5. A patient splint wear diary

Concomitant care

Any relevant contralateral thumb treatment for participants will be delayed until the end of the trial. There will be no alteration in participants' general concomitant care whilst on the trial, . Additional treatments e.g. joint injection, surgery and reported purchase or use of own splints during the study period will be captured on self-report questionnaires. Criteria for modifying allocated interventions is detailed within the OTTER II Trial Safety Information.

Investigator Training

Trial training visits will be conducted by the OTTER II trial management team (JA, PB, PW) to carry out standardised instruction and demonstration for OTTER II research clinicians prior to the start of patient recruitment. Training will cover clinician blinding procedures, delivery of eligibility tests, interventions, maintaining participant blinding, reporting of safety events, data entry and use of Case Report Forms (CRFs). Sites will also be visited by the trial team (JA, MW, CM) to provide support and guidance on maximising participant recruitment through liaison with surgery and community health teams, and to conduct quality assurance evaluations of intervention delivery as required.

A page of the trial website which is only available to site staff (not participants or the public) will provide training videos on the standardised delivery of all the trial interventions. All clinicians will complete Good Clinical Practice training.

For peer review only - http://bmjopen.bmj.com/site/about/guidelines.xhtml

Participant identification and baseline assessment

Potential participants will receive a letter about the study and a Participant Information Sheet at the hospital clinic or by post. For interested patients an out-patient therapy department appointment will be made at Week 0 (Baseline). Following eligibility screening, formal written consent will be given and participants will complete the baseline assessments.

Randomisation

Eligible patients will be enrolled into the study via the Oxford Clinical Trials Research Unit (OCTRU) online randomisation service and this system will record eligibility and stratification data. Participants will then be randomised to a treatment arm and this constitutes the date of start of treatment. Participants will only be randomised once and for the treatment of one thumb. For participants with symptoms of bilateral thumb base OA the most painful thumb will be treated first within the trial. If at the end of the trial participants require treatment on a contralateral thumb then participants will be invited back by the clinical service to undergo routine (i.e. non-trial) clinical intervention at the end of the trial.

Randomisation will be on a 1:1:1 basis. The first 30 participants will be allocated to their treatment using simple randomisation. Subsequently, a validated computer randomisation program with a minimisation algorithm using a probabilistic element will be used. This randomisation will be stratified by centre, baseline AUSCAN hand pain score (Category 1= AUSCAN hand pain score 6 to 12 and Category 2= AUSCAN hand pain score 13 to 20) and treated hand dominance, to ensure treatment groups are balanced for potential predictors of outcome.

The Participant Information Sheet will state that an element of the treatment may be placebo. This approach will support participant blinding, in addition to the training that treating therapists will receive about delivering placebos and responding to participant questions. The success of participant blinding will be assessed via patient questionnaires at 12 weeks.

Treatment and follow up

For peer review only - http://bmjopen.bmj.com/site/about/guidelines.xhtml

Figure 1 flowchart shows the participants' progress through the trial. After randomisation participants will receive their allocated intervention during a 60 minute appointment (week 0). All participants in each treatment arm will receive a 10 minute telephone call at two weeks from the therapist to check for adherence to the self-management programme and to discuss any problems identified by the participant as relevant. In addition for Group B and C participants the therapist will check splint wear and comfort as per good practice guidelines. Participants will attend for a 30 minutes appointment 4 weeks after randomisation, to review treatments and make adjustments where necessary. The final hospital visit occurs 8 weeks after randomisation. A questionnaire will be posted to each participant at 12 weeks to collect follow-up patient reported outcomes. After the 12 week follow-up has been completed (study end point) all patients will be offered the option of a further follow up appointment for any further care that is needed.

Qualitative Interviews

Between weeks 8 and 12 a qualitative telephone interview will take place for a subgroup of patients in intervention arm B and C. Purposive sampling will be used and based on male to female ratio (as per the study sample); age and AUSCAN hand pain index at baseline. Interviews will be conducted with up to 40 participants. Interviews will be semi-structured, using a topic guide informed by patient partners, to elicit experience of splint and self-management interventions, splint preference, adherence and views about reasons for effectiveness. There will be a password for each participant that has agreed to take part in the interview study so that the participant can be correctly identifed on the telephone. Consent for participation in the qualitative interview will be sought at the time of entry into the main study.

The schedule of enrolment, interventions and a summary of assessments are shown in **Table 2**. Detailed information about baseline assessments and outcome measures are listed in **Table 3.**

For peer review only - http://bmjopen.bmj.com/site/about/guidelines.xhtml

	STUDY PERIOD							
	**Enrolment**	**Allocation**	**Post-allocation**					**Close-out**
**TIMEPOINT:**	**0**	**0**	**2 wks**	**4 wks**	**8 wks**	**9-11 wks**	**12 wks**	**After 12 wks**
**ENROLMENT:**								
**Eligibility screen***	X							
**Informed consent***	X							
**Randomisation/ Allocation**		X						
**INTERVENTIONS:**								
**Optimal NHS self-management care**		X						
**Optimal NHS self-management care plus verum splint**		X						
**Optimal NHS self-management care plus placebo splint**		X						
**Telephone call check of progress**			X					
**Review of self-management care and splint wear (if applicable)**				X				
**ASSESSMENTS:**								
**Baseline Assessments: See Table 3**	X							
**Outcomes: Grip Ability Test [31]**				X	X			
**Outcomes: See Table 3**					X			
**Outcomes at follow up: See Table 3**							X	

For peer review only - http://bmjopen.bmj.com/site/about/guidelines.xhtml

**Exercise adherence**		◆——	———	———	———	———	——◆	
**Splint adherence**		◆——	———	———	———	———	——◆	
**Qualitative Interviews (selected participants)**						X		
**Patient request for further treatment**								X

Table 2. Schedule of enrolment, interventions, and assessments

*The eligibility screen and informed consent can optionally be carried out prior to time point 0.

Measure	Screening	Baseline	4 weeks	8 weeks	12 weeks
**Work Productivity and Activity Impairment Questionnaire (WPAI) [32]**		✓		✓	✓
**Generic Quality of Life (SF12-V2) [33]**		✓		✓	✓
**EuroQol 5 Dimensions 5-Levels questionnaire (EQ5D-5L) [34]**		✓		✓	✓
**AUSCAN hand stiffness [28]**		✓		✓	✓
**Michigan Hand Questionnaire (MHQ) [35]**		✓		✓	✓
**Thumb pain over the last week**		✓		✓	✓
**Disability of the arm, shoulder, hand questionnaire (DASH) [36]**		✓		✓	✓

For peer review only - http://bmjopen.bmj.com/site/about/guidelines.xhtml

**Leisure section only**					
**Arthritis Self-Efficacy Pain Scale [37]**		✓		✓	✓
**AUSCAN hand pain [28]**	✓			✓	✓
**AUSCAN hand function [28]**	✓			✓	✓
**Global assessment of change [38]**				✓	✓
**Health utilisation questionnaire**				✓	✓
**The objective clinician assessed Grip Ability Test (GAT) [31]**		✓	✓	✓	

Table 3. Baseline assessment and outcome measures. The AUSCAN hand index for pain [28] recorded at baseline and 8 weeks is the primary outcome measure.

Sample Size

The sample size has been calculated based on undertaking a global Analysis of Covariance (ANCOVA) for the primary outcome, AUSCAN hand pain [28] at 8 weeks, adjusting for the baseline pain score and stratification factors (including centre and treated hand dominance) across all three treatment arms using the power and sample size package, PASS 11 (PASS 11. NCSS, LLC. Kaysville, Utah, USA. www.ncss.com). Assuming 80% power, a 5% 2-sided significance in order to detect a standardised mean difference of 0.4 (a moderate effect size based on a difference in the AUSCAN hand pain score of 2 points and assuming a standard deviation of 5, based on data from the OTTER Pilot study), requires 92 participants per arm. Allowing a 20% loss to follow-up at 8 weeks inflates this to 115 participants per arm, giving a total of 345 participants.

The sample size has taken into account the global comparison of the null hypothesis that there is no difference between the three treatment arms, and pairwise comparisons will only be undertaken if

For peer review only

For peer review only - http://bmjopen.bmj.com/site/about/guidelines.xhtml

this global comparison is statistically significant. No further adjustment for the sample size has been undertaken to allow for multiple testing.

Clustering is not a consideration in sample size as "Centre" is a stratification factor, and although there may be more than one therapist per centre, this should largely ensure balance across treatment arms within each centre. The Data and Safety Monitoring Committee (DSMC) will review the assumptions underlying this sample size calculation after approximately 50% of the participants have been recruited and followed up for 8 weeks, if still within the recruiting period.

Recruitment strategy

Recruitment targets and procedures have been tested in the pilot study [26] and are based on these data. Recruitment start dates for the collaborating sites will be staggered over a 6-month period.

Data collection, management and analysis

The trial will use a series of CRFs to record trial activities. RCT site staff will ensure that each CRF is completed properly and stored in the Investigator's Site File (ISF). Site staff will make photocopies and post CRFs to the OTTER Trial team using FREEPOST envelopes, according to a standard operating procedure (SOP), so that both sites and the trial centre will have a copy of CRFs.

Participants will complete questionnaires at baseline, 8 weeks and 12 weeks. Participants will be asked to record the frequency for which hand exercises were completed for at least 20 minutes using a daily diary. Splint wear diaries will be used to capture hours per day of splint wear. Participants will be provided with FREEPOST envelopes in order to return questionnaires and diaries to the trial centre.

Where postal follow-up questionnaires are not received when expected, first they will be chased by post, and if there is no response key endpoint data will be collected over the phone. Up to three phone calls will be made to obtain key endpoint data. If participants wish to withdraw from their

For peer review only - http://bmjopen.bmj.com/site/about/guidelines.xhtml

For peer review only

randomised intervention, they will still be included in the trial follow-up, unless they request to be excluded from follow-up. Where available, reasons for withdrawal from follow-up will be collected.

The OTTER Research Team will make copies of CRFs, questionnaires and diaries created by or received directly at the trial centre and post them to the relevant site to be filed in the ISF. The original documents will be secured in the Trial Master File.

Eligibility and stratification data will be entered directly onto the OCTRU online randomisation system by site staff. All other data will be entered into the trial database by the OTTER Research Team. The OTTER trial team will check each CRF for completeness and where appropriate contact the site for missing data. A Data Management and Sharing Plan contains fully comprehensive information about all aspects of data management. All data will be processed according to the Data Protection Act 2018. All study specific documents, except for the signed consent form and letters to participants, will refer to the participant with a unique study participant number/code and not by name.

Quantitative data will be stored on an OpenClinica trial-specific database prepared and managed by OCTRU. Qualitative data will be stored in QRS NVivo and word documents. The trial database is designed by OCTRU and has inbuilt data validation checks and a trial management system for managing data queries. Peer review of data entry will be conducted on 5% of CRFs. Data discrepancies will be reviewed on a regular basis to help clean the data. All data will be securely stored, backed up and managed in accordance with the University of Southampton and the University of Oxford research data management policies.

All study data will be stored securely; only accessible by authorised personnel agreed by the Principal Investigator, Legal services at the University of Southampton and The OTTER II Trial Steering Committee. Participant identifiable data will be stored separately from study data. Trial documentation will be retained for 10 years after completion of study-related activities either in paper or electronic format.

For peer review only - http://bmjopen.bmj.com/site/about/guidelines.xhtml

Audio-recordings from the interviews will be transcribed verbatim, and participants allocated an ID number. The text of the interviewer notes from the telephone interviews will be anonymised and linked to the interview through the ID number. Names of participants, the names of any people discussed, health care employees and hospitals will be removed from transcripts and replaced with pseudonymns. Participants will be sent a letter to thank them for taking part in the telephone interview. A 'future use of interview' question is included on the consent form which gives copyright to the Universities of Southampton and Oxford to use the material in research, teaching, publications and broadcasting.

Statistical analysis

This three-arm trial will first assess the global comparison of differences between the three treatment arms at the 5% level. The primary (global) Null Hypothesis is that there is no difference between the three arms. Only if this null hypothesis is rejected at the 5% (2-sided) significance level will the pairwise comparisons be carried out in order to explore where the difference lies:

· Verum splint + self-management programme (Group B) versus self-management programme alone (Group A)

· Placebo splint + self-management programme (Group C) versus self-management progamme alone (Group A)

· Verum splint + self-management programme (Group B) versus placebo splint + self-management programme (Group C)

It is anticipated that there will be an adjustment for multiple testing at this stage, and Bonferroni or a less conservative method will be used. A Statistical Analysis Plan will provide full details of all planned analyses.

The statistical analysis of the primary outcome, AUSCAN hand pain score, will be performed using ANCOVA, adjusting for baseline pain and stratification factors. Continuous secondary outcomes will be analysed using similar methods to the primary outcome. The unadjusted secondary binary

For peer review only - http://bmjopen.bmj.com/site/about/guidelines.xhtml

variables will be compared using chi-squared tests with logistic regression being used to adjust for stratification and important prognostic factors in a multi-variable framework.

Baseline characteristics for the three groups will be presented. Baseline characteristics of participants who completed the trial and those who dropped out will be presented and explored in order to ascertain patterns of loss to follow-up. Subgroups will be examined to look for consistency of any observed treatment effects (using interactions). This analysis will be considered of an exploratory nature potentially providing hypotheses for future studies. The study is not powered to examine these in detail and will not therefore report p-values for these.

Qualitative analysis

Forty interviews will be carried out by a researcher blind to participant treatment allocation. Audio-recordings of the 40 interviews will be transcribed verbatim, anonymised and imported into analysis software QRS NVivo. Using a thematic approach [39] data will be inductively coded, codes grouped to create categories and a descriptive account will be produced. Up to 10 interviews will be double coded to increase the validity of the findings. The account will provide structured descriptions of participants' experience of self-management and splints, including reasons for their views about effectiveness as well as information about acceptability and adherence.

Quality of Life Analysis

The EQ-5D-5L [40] will be administered to participants at baseline, 8 weeks and 12 weeks. Responses will be converted into utilities using tariffs estimated from a representative sample of the UK population. Survival information collected from the trial will be combined with EQ-5D utilities to generate Quality Adjusted Life-Years (QALYs).

Economic Evaluation and Analysis

The perspective adopted in the economic analysis will be that of the NHS. Costs associated with the following healthcare resource categories over the 8 week intervention period and follow-up period (12 weeks) will be included.

For peer review only - http://bmjopen.bmj.com/site/about/guidelines.xhtml

· Intervention provision (including: splints and clinical staff time required for splinting); and

· Primary care contacts, including surgery and home visits by GPs, nurses, and out-of-hours medical services, and community therapists; and

· Hospital care services, including scheduled and unscheduled inpatient admissions, surgery, accident and emergency visits and outpatient care contacts.

Primary and hospital care resource use will be obtained from patient questionnaires administered at 8 weeks and at 12 week follow-up. Healthcare resources will be valued using unit cost schedules such as Personal Social Services Research Unit and NHS Reference costs. Costs associated with splints and other disposables will be obtained from the manufacturers and the NHS Supply Chain catalogue. Using the WPAI [32] the number of work days lost by study participants and the impact that OA had on the levels of productivity/activity and unpaid work will also be measured over both study periods.

An economic evaluation adherent to guidelines for good economic evaluation practice will be undertaken. A within-trial cost-utility analysis will explore the incremental cost per QALY gained by splinting of the thumb base and a self-management programme when compared to: 1) placebo-splinting and a self-management programme; and 2) a self-management programme alone. The analyses will be conducted at eight weeks and at 12 weeks. Cost and effect results will be reported as means with standard deviations, with mean differences between the two patient groups reported alongside 95% confidence intervals. Depending on the amount of missing cost and quality of life data, missing data will be imputed using recommended multiple imputation methods [41], with results from this analysis being presented as an additional sensitivity analysis.

Incremental cost-effectiveness will be calculated by dividing the difference in costs by the difference in effects. Uncertainty around the incremental cost-effectiveness ratio will be explored using non-parametric bootstrapping. A supplementary economic evaluation including non-NHS costs will be conducted in an additional sensitivity analysis. This will include costs of impaired productivity/activity and, any work and non-work days (e.g. leisure or non-paid work) lost due to illness will be valued using mean average wages for those in employment. For participants who are retired or those not in

For peer review only - http://bmjopen.bmj.com/site/about/guidelines.xhtml

employment, loss in activity levels (e.g. leisure, caring or non-paid work activities) due to illness will be valued using minimum wages.

ETHICS AND DISSEMINATION

The OTTER II trial will undergo regular monitoring. A standard risk assessment will be conducted and a risk-based proportionate monitoring plan will be put in place, which will include central monitoring.

Adverse event and safety oversight will comply with OCTRU SOPs. Adverse Responses/Reactions, Adverse Device Effects, Serious Adverse Responses/Reactions, Serious Adverse Device Effects, Suspected Unexpected Serious Adverse Responses/Reactions (SUSARs) and Device Deficiencies will be recorded. Sites will be required to report all serious events that are related to a trial intervention within 24 hours and all related serious adverse events must be assessed for causality and reason for seriousness. All Device Deficiencies, Adverse Reactions and Adverse Device Effects must be reported to the trial team within 2 weeks. All Serious Adverse Event safety monitoring forms will be passed on to the Trial's Nominated Clinician who will perform an independent assessment of causality and will also perform assessment of expectedness based on what is known and documented about the intervention/device. Any SUSARs will be reported to the Research Ethics Committee (REC) within 15 days of the Trials Office being made aware of the event.

An independent DSMC (Appendix 2) will be convened with two expert clinicians and a statistician to regularly review accumulating data in order to assess patient safety and study conduct following the recommendations of the DAMOCLES study [42] with full details being provided in a charter. They will advise the Trial Steering Committee (TSC) (Appendix 3) as to whether recruitment should stop early, which is only likely to occur if either intervention is shown to be unsafe for patients. As this is a low risk study no interim efficacy comparative analyses are planned and no safety issues are expected. The OTTER II trial team will have access to the final trial datset and there will be no public access to patient level data or the statistical code used within the trial.

For peer review only - http://bmjopen.bmj.com/site/about/guidelines.xhtml

We believe this to be the first fully powered placebo controlled splinting trial around thumb base OA. Study results will be disseminated to rheumatology, hand therapy, occupational therapy and physiotherapy national and international conferences and submitted for consideration in international and national academic and professional conferences and journals. Lay publications written in accessible language will be provided to charitable, community and patient facing publications as recommended by our patient and public involvement partners. Study participants will be provided regular updates of the study progress through the OTTER II study website.

For peer review only

For peer review only - http://bmjopen.bmj.com/site/about/guidelines.xhtml

Authors' contributions:

Jo Adams: Principal investigator, conceived research project and first author of paper

Paula Barratt: Trial manager, written and reviewed trial protocol and co-author

Nigel Arden: Trial team member contributed to original protocol and co-applicant and co-author

Sofia Barbosa Bouças: Trial team member contributed to pilot development and original protocol, co-applicant and co-author

Sarah Bradley: Trial team member contributed to original protocol and co-applicant and co-author

Michael Doherty: Trial team member contributed to original protocol and co-applicant and co-author

Susan Dutton: Trial team member contributed to original protocol, statistical lead, co-applicant and co-author

Krysia Dziedzic: Trial team member contributed to original protocol and co-applicant and co-author

Rachael Gooberman-Hill: Trial team member contributed to original protocol and co-applicant and co-author

Kelly Hislop: Trial team member contributed to pilot development and original protocol, co-applicant and co-author

Corinne Hutt Greenyer: OTTER patient partner, co-applicant, co-author, reviewed and revised paper

For peer review only

For peer review only - http://bmjopen.bmj.com/site/about/guidelines.xhtml

Vic Jansen: Trial team member contributed to revision and clinical review of original protocol and co-author

Ramon Luengo Fernadez: Trial team member contributed to original protocol and co-applicant and co-author, led health economic input

Claire Meagher: Trial team member, reviewed and revised qualitative component and co-author

Peter White: Trial team member contributed to original protocol and placebo development and co-applicant and co-author

Mark Williams: Trial team member contributed to original protocol reviewed and revised paper

Funding statement: 'This work was supported by Arthritis Research UK Grant Ref 21019 and the funder is listed and complies with the SHERPA JULIET guidelines
http://v2.sherpa.ac.uk/id/funder/14

Competing interests statement. The authors and wider OTTER II trial team have no competing interests

Acknowledgements.
The authors wish to acknowledge the OTTER II collaborative group:

Derby Teaching Hospitals NHS Foundation Trust: Victoria Jansen, Helen McKenna, Ellen Bramall, Carole Henderson, Chloe Kirk, Diane Langford, James Turner, Anna Selby, Linda Tozer, Navdeep Johal, Fernando Parrales, Madina Asif, Kat Hill, Stefania Wigelsworth
Doncaster and Bassetlaw Teaching Hospitals NHS Foundation Trust: Su McIlwaine, Elaine Bonser, Lynn Houghton, Ryan Roberts
Dorset County Hospital NHS Foundation Trust: Maree Dethick-Jones, Sheena Colhoun, Louise Clark, Liz Barnish, Simone Caddy, Josie Goodsell, Sarah Horton, Laura Howell, Simon Sharpe
Hampshire Hospitals NHS Foundation Trust: Kevin Spear, Christina Macleod, Libby Denman, Becky Shaylor, Kathy Whalley, Mark Pulley, Hannah Bolger
Midlands Partnership NHS Foundation Trust: Carol Graham, Nicky Edwards, Jane Rivers-Latham, Yvonne Salt, Tilly Grocott, Alison Williams, Sarah Gibson, Lisa Oakley, Susan Thompson, Susan Woodroffe, Laura Denny, Amy Thompson, Natalie Wheat
North Devon Healthcare NHS Trust: Jo Harness, Jane Hunt, Henrietta Clay, Becky Holbrook, Lucia Stancombe, Martin Howard, Nicholas McGuirk, Mark Bryce

For peer review only - http://bmjopen.bmj.com/site/about/guidelines.xhtml

Pennine MSK Partnership Ltd: Jill Firth, Kathy Kinsey, Helen Light, Charlotte Critchley, David Pilbury, Danielle Burke, Karen Partridge, Tracy Parry, Norah Handley, Kath Spencer
Poole Hospital NHS Foundation Trust: Sarah Bradley, Corinna Rogers, Paula Reynolds, Bridget Ellis, Sharon Page
Portsmouth Hospitals NHS Trust: Caroline Mountain, Gemma Willis, Catherine Coleman, Catherine Kirby, Paula White, Glenn Lake, Julie Williams, Marie White
Royal Devon and Exeter NHS Foundation Trust: Suzannah Blake, Abigail Owen, Emily Rogers, Claire Hughes, Cresta Browning, Jacqueline Fowler, Cristina Burke-Trees
Royal Free London NHS Foundation Trust: Juliette Bray, Nikki Burr, Meera Anadkat, Cherry Kilbride, Francesca Gowing
Royal United Hospitals Bath NHS Foundation Trust: Sandi Derham, Jessica Chipps, Suzanne Green, Helen Gordon-Johnson, Mark Sheriff, Susan Greene, Michelle Lawrence, Belinda Jones, Jack Spence, Ali Champion, Emily Anson
The Robert Jones and Agnes Hunt Orthopaedic Hospital NHS Foundation Trust: Tracy Jones, Michelle Jones, Julie Steen, Daniel Griffiths, Sally van Liefland, Jayne Edwards, Linda Griffith
Yeovil District Hospital NHS Foundation Trust: Helen Truman, Frances Campbell, Helen Jones, Jennifer Harrison, Vickie Ridley, Sue Chesterman, Joanna Allison, Tressy Pitt-Kerby, Kate Beesley

The authors also wish to acknowledge the OTTER II Trial Administrator: Carrie Fanning, University of Southampton, Southampton, UK.

Legend for Figure 1:

Figure 1. Participants' process throughout the trial

For peer review only

For peer review only - http://bmjopen.bmj.com/site/about/guidelines.xhtml

References

1. Abhishek A, Doherty M. Clinical Features of Osteoarthritis In: Watts R, Conaghan P, Denton C, et al., eds. Oxford Textbook of Rheumatology (4 ed). Oxford Oxford University Press, 2013.
2. Maheu E, Berenbaum F, . Time for new outcome measures in hand osteoarthritis? . F Nat Clin Pract Rheumatol 2009;50 (3):136-38
3. Marshall M, Peat G, Nicholls E, et al. Subsets of symptomatic hand osteoarthritis in community-dwelling older adults in the United Kingdom: prevalence, inter-relationships, risk factor profiles and clinical characteristics at baseline and 3-years Osteoarthritis and Cartilage2013;21(11):1674-84
4. Haugen IK, Englund M, Aliabadi P, et al. Prevlance, incidence and progression of hand osteoarthritis in the general population:the Framingham osteoarthritis study. 2011 doi: 10.1136/ard.2011.150078[published Online First: Epub Date].
5. Bijsterbosch J, Visser W, Kroon H, et al. Thumb base involvement in symptomatic hand osteoarthritis is associated with more pain and functional disability. Ann Rheum Dis 2010;69(3):585-87 doi: 10.1136/ard.2009.104562. Epub 2010 Feb 2[published Online First: Epub Date].
6. Zhang W, Doherty M, Leeb B, et al. EULAR evidence-based recommendations for the diagnosis of hand osteoarthritis: report of a task force of ESCISIT. Ann Rheum Dis 2009;68:8-17 doi:10.1136/ard.2007.084772[published Online First: Epub Date].
7. Spacek E, Poiraudeau S, Fayad F, et al. Disability induced by hand osteoarthritis: are patients with more symptoms at digits 2-5 interphalangeal joints different from those with more symptoms at the base of the thumb? Osteoarthritis and Cartilage 2004;12:366-73
8. Poole JU, Pellegrini VD, Jr. Arthritis of the thumb basal joint complex. Journal of hand therapy : official journal of the American Society of Hand Therapists 2000;13(2):91-107
9. Zhang Y, Niu J, Kelly-Hayes M, et al. Prevalence of symptomatic hand osteoarthritis and its impact on functional status among the elderly: The Framingham Study. Am J Epidemiol 2002;156(11):1021-7
10. Hill S, Dziedzic K, Ong B. The functional and psychological impact of hand osteoarthritis. Chronic Illness 2010;6:101-10
11. Rannou F, Dimet J, Boutron I, et al. Splint for base-of-thumb osteoarthritis: a randomized trial. Annals of Internal Medicine 2009;150(10):661-69
12. Kloppenburg M, Kroon FP, Blanco FJ, et al. 2018 update of the EULAR recommendations for the management of hand osteoarthritis. Annals of the Rheumatic Diseases 2018 doi: 10.1136/annrheumdis-2018-213826[published Online First: Epub Date]|.
13. Arazpour M, Soflaei M, Ahmadi Bani M, et al. The effect of thumb splinting on thenar muscles atrophy, pain, and function in subjects with thumb carpometacarpal joint osteoarthritis. Prosthetics and Orthotics International 2016;41(4):379-86 doi: 10.1177/0309364616664149[published Online First: Epub Date].
14. Hermann M, Nilsen T, Eriksen CS, et al. Effects of a soft prefabricated thumb orthosis in carpometacarpal osteoarthritis. Scandinavian Journal of Occupational Therapy 2014;21(1):31-39 doi: 10.3109/11038128.2013.851735 published Online First: Epub Date].
15. Zhang W, Doherty M, Leeb BF, et al. EULAR evidence based recommendations for the management of hand osteoarthritis: report of a Task Force of the EULAR Standing

For peer review only - http://bmjopen.bmj.com/site/about/guidelines.xhtml

Committee for International Clinical Studies Including Therapeutics (ESCISIT). Ann Rheum Dis 2007;66(3):377-88
16. Kjeken I, Smedslund G, Moe RH, et al. Systematic Review of Design and Effects of Splints and Exercise Programs in Hand Osteoarthritis. Arthritis Care & Research 2011;63(6):834-48 doi: 10.1002/acr.20427[published Online First: Epub Date].
17. Ye L, Kalichman L, Spittle A, et al. Effects of rehabilitative interventions on pain, function and physical impairments in people with hand osteoarthritis: a systematic review. Arthritis Research & Therapy 2011;13(1) doi: 10.1186/ar3254[published Online First: Epub Date].
18. Sanchez K, Jourdan C, Rannou F, et al. Assessing Orthosis:Practical Examples. In: Boutron I, Ravaud P, Moher D, eds. Randomized Clinical trials of Nonpharmacological Treatments: Chapman and Hall/CRC Biostatistics Series 2012:295-307.
19. White P, Bishop F, Prescott P, et al. Practice, practitioner, or placebo? A multifactorial, mixed-methods randomized controlled trial of acupuncture Pain 2012;153(2):455-62
20. Finniss D, Kaptchuk T, Miller F, et al. Biological,clinical, and ethical advances of placebo effects The Lancet 2010;375:868-695
21. Chan A-W, Tetzlaff JM, Gøtzsche PC, et al. SPIRIT 2013 Explanation and Elaboration: Guidance for protocols of clinical trials. BMJ 2013;346 e7586 doi: https://doi.org/10.1136/bmj.e7586[published Online First: Epub Date].
22. Gooberman Hill R, Jinks C, Barbosa Boucas S, et al. Designing a placebo device: involving service users in clinical trial design. Health Expectations 2013;16(4):e100–e10 doi: doi:10.1111/hex.12043[published Online First: Epub Date].
23. Barbosa Boucas S, Hislop K, Dziedic K, et al. Defining optimal NHS occupational therapy treatment, individualized splint and placebo splint for patients with thumb base OA: a Delphi study. Rheumatology 2013a;52(S1):i105
24. Barbosa Boucas S, Hislop Lennie K, Dziedzic K, et al. Differences between service providers and users when defining feasible optimal NHS occupational therapy treatment for patients with thumb base OA: Results from a Delphi study. Annals of the Rheumatic Diseases 2013b**;72**(S3):779-80
25. Jones L, White P, Donovan-Hall M, et al. The Thoughts and Feelings Held by Clinicians about the delivery of a Placebo Thumb Splint in an Osteoarthritis Rehabilitation Trial. Hand Therapy 2013;18(3):77-83
26. Adams J, Barbosa Bouças S, Hislop K, et al. The Effectiveness and Efficacy of Splints for Thumb Base Osteoarthritis: A Pilot Randomized Controlled Trial. Rheumatology 2014;53(S1):i41-i42 doi: 10.1093/rheumatology/keu090.005[published Online First: Epub Date]|.
27. Adams J, Barratt P, Bradley S, et al. Participating in a musculoskeletal randomised controlled trial: identification of education training needs by occupational therapists and physiotherapists in the uk. Annals of the Rheumatic Diseases 2017;76(S2):1508-08 doi: 10.1136/annrheumdis-2017-eular.1095[published Online First: Epub Date].
28. Bellamy N, Campbell J, Haraoui B, et al. Clinimetric properties of the AUSCAN Osteoarthritis Hand Index: an evaluation of reliability, validity and responsiveness. Osteoarthritis Cartilage 2002;10(11):863-9
29. Gelberman RH, Boone S, Osei DA, et al. Trapeziometacarpal Arthritis: A Prospective Clinical Evaluation of the Thumb Adduction and Extension Provocative Tests. J Hand Surg Am 2015;40(7):1285-91 doi: 10.1016/j.jhsa.2015.04.012[published Online First: Epub Date].

For peer review only - http://bmjopen.bmj.com/site/about/guidelines.xhtml

30. Loyley M, Davis L. An investigation of the pressure, temperature and functional impact of thumb base splints for people with thumb based pain [MSc research dissertation paper]. University of Southampton 2017.
31. Dellhag B, Bjelle A. A grip ability test for use in rheumatology practice. The Journal of Rheumatology 1995;22(8):1559-65
32. Reilly MC, Zbrozek AS, Dukes EM. The validity and reproducibility of a work productivity and activity impairment instrument. PharmacoEconomics 1993;4(5):353-65
33. Ware JE, Kosinski M, Keller SD. A 12-item short-form health survey, construction of scales and preliminary tests of reliability and validity. Medical care 1996;34(3):220-33
34. Euroqol Group. Euroqol: a new facility for the measurement of health-related quality of life. Health Policy 1990;16:199-208
35. Chung KC, Pillsbury MS, Walters MR, et al. Reliability and validity testing of the Michigan Hand Outcomes Questionnaire The Journal of Hand Surgery 1998;23A(4):575-87
36. Hudak PL, Amadio PC, Bombardier C. Development of an upper extremity outcome measure: The DASH (Disabilities of the Arm, Shoulder and Hand). American Journal of Industrial Medicine 1996;29:602-08
37. Lorig K, Chastain R, Ung E, et al. Development and evaluation of a scale to measure self-efficacy in people with arthritis. Arthritis Rheum 1989;32(1):37-44
38. Pham T, van der Heijde D, Altman RD, et al. OMERACT-OARSI initiative: Osteoarthritis Research Society International set of responder criteria for osteoarthritis clinical trials revisited. Osteoarthritis Cartilage 2004;12(5):389-99
39. Charmaz K. Constructing grounded theory: a practical guide through qualitative analysis London Sage 2006.
40. EuroQol Group--a new facility for the measurement of health-related quality of life. Health Policy 1990;16(3):199-208
41. Royston P. Multiple imputation of missing values. Stata Journal 2004;4:227-41
42. DAMOCLES Study Group NHS Health Technology Assessment Programme. A proposed charter for clinical trial data monitoring committees: helping them to do their job well. Lancet 2005;365:711-22

For peer review only

For peer review only - http://bmjopen.bmj.com/site/about/guidelines.xhtml

OTTER II Trial: flow of participants

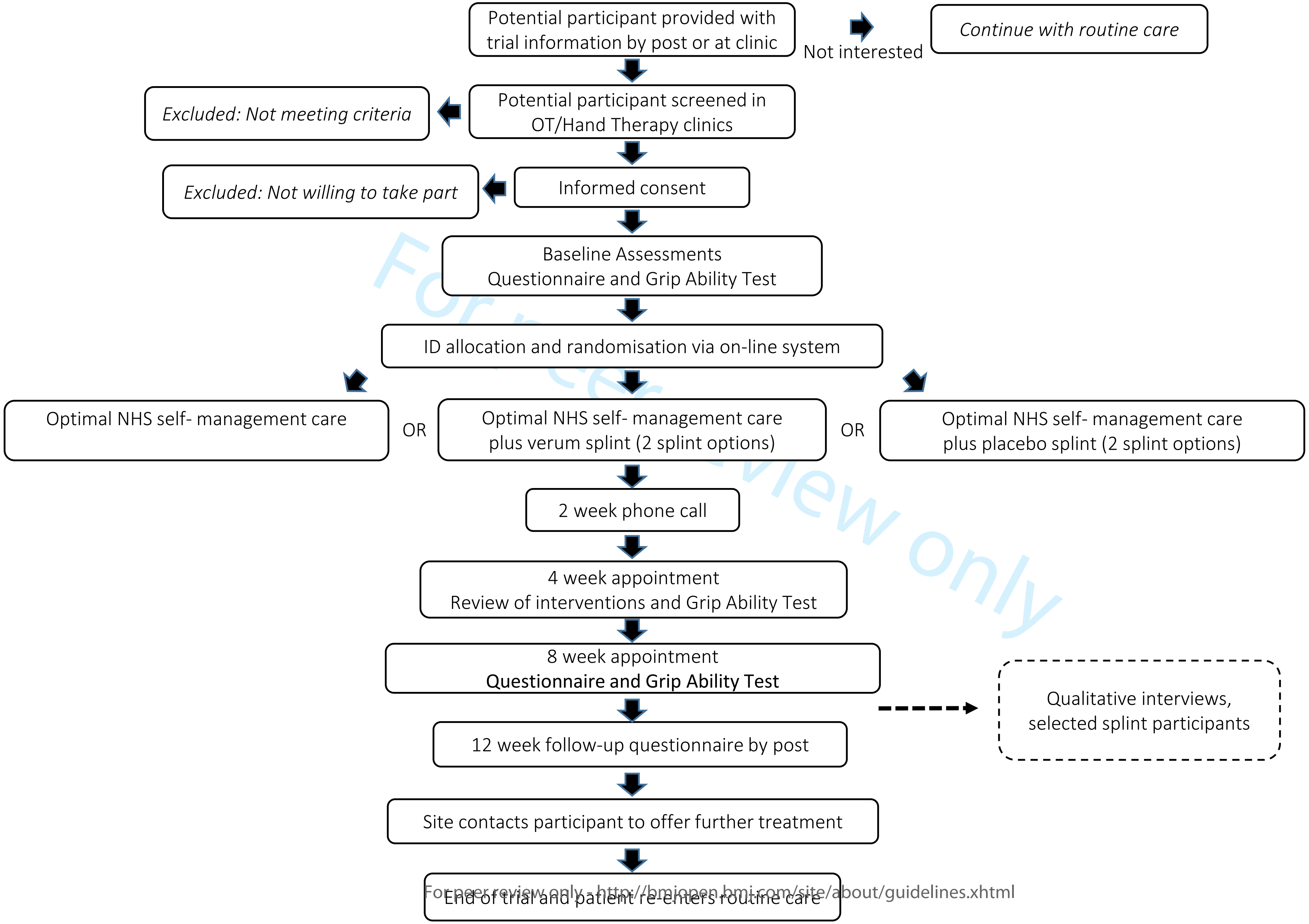

For peer review only - http://bmjopen.bmj.com/site/about/guidelines.xhtml

APPENDICES

Appendix 1

NHS recruitment sites

Pulvertaft Hand Centre, Royal Derby Hospital, Derby Teaching Hospitals NHS Foundation Trust, Derby, UK

Hand Therapy Department, Dorset County Hospital, Dorset County Hospital NHS Foundation Trust, Dorchester, UK

Hand Therapy Unit, Poole Hospital, Poole Hospital NHS Foundation Trust, Poole, UK

Plastic Surgery Rehabilitation, Royal Devon and Exeter Hospital, Royal Devon and Exeter NHS Foundation Trust, Exeter, UK

Occupational Therapy Department, North Devon District Hospital, North Devon Healthcare NHS Trust, Barnstaple, UK

Rheumatology Therapy Department, Royal National Hospital for Rheumatic Diseases, Royal United Hospitals Bath NHS Foundation Trust, Bath, UK

Therapies Department, Royal United Hospitals, Royal United Hospitals Bath NHS Foundation Trust, Bath, UK

Occupational Therapy Department, Haywood Hospital, Midlands Partnership NHS Foundation Trust, Stoke-on-Trent, UK

Pennine MSK Partnership Ltd., Integrated Care Centre, Oldham, UK

Clinical Therapies Department, Bassetlaw Hospital, Doncaster and Bassetlaw Teaching Hospitals NHS Foundation Trust, Worksop, UK

Bassetlaw Locality Retford Primary Care Centre, Doncaster and Bassetlaw Teaching Hospitals NHS Foundation Trust, Retford, UK

Therapy Department, Yeovil Hospital, Yeovil District Hospital NHS Foundation Trust, Yeovil, UK

Hand Therapy, Basingstoke and North Hampshire Hospital, Hampshire Hospitals NHS Foundation Trust, Basingstoke, UK

Therapy Department, Royal Hampshire County Hospital, Hampshire Hospitals NHS Foundation Trust, Winchester, UK

Occupational Therapy Department, Queen Alexandra Hospital, Portsmouth Hospitals NHS Trust, Cosham, UK

For peer review only

Hand Therapy Unit, The Robert Jones and Agnes Hunt Orthopaedic Hospital, The Robert Jones and Agnes Hunt Orthopaedic Hospital NHS Foundation Trust, Oswestry, UK

Hand Therapy, The Royal Free Hospital, Royal Free London NHS Foundation Trust, London, UK

Appendix 2

DSMC members

Anisur Rahman (Chair), Department of Rheumatology, University College London, London, UK

Ross Wilkie, Arthritis Research UK Primary Care Centre, Keele University, UK

Ranjit Lall, Warwick Clinical Trials Unit, University of Warwick, Coventry, UK

Appendix 3

TSC members

Sarah Dean (Chair), University of Exeter Medical School, University of Exeter, Exeter, UK

Cathy Ball, Kennedy Institute of Rheumatology, University of Oxford, Oxford, UK

Lindsay Bearne, Faculty of Life Sciences & Medicine, King's College London, London, UK

Elaine Dennison, MRC Lifecourse Epidemiology Unit, University of Southampton, Southampton, UK

Corinne Hutt Greenyer, School of Health Sciences, University of Southampton, Southampton, UK

Jo Adams, School of Health Sciences, University of Southampton, Southampton, UK

For peer review only

STANDARD PROTOCOL ITEMS: RECOMMENDATIONS FOR INTERVENTIONAL TRIALS

The Osteoarthritis Thumb Therapy Trial (OTTER II)

SPIRIT 2013 Checklist: Recommended items to address in a clinical trial protocol and related documents*

Section/item	Item No	Description	Addressed on page number
**Administrative information**			
Title	1	Descriptive title identifying the study design, population, interventions, and, if applicable, trial acronym	1___________
Trial registration	2a	Trial identifier and registry name. If not yet registered, name of intended registry	3___________
	2b	All items from the World Health Organization Trial Registration Data Set	Complete____
Protocol version	3	Date and version identifier	V14.0 dated 23 Aug 2018
Funding	4	Sources and types of financial, material, and other support	23___________
Roles and responsibilities	5a	Names, affiliations, and roles of protocol contributors	1, 22, 23_______
	5b	Name and contact information for the trial sponsor	University of Southampton rgoinfo@soton.ac.uk
	5c	Role of study sponsor and funders, if any, in study design; collection, management, analysis, and interpretation of data; writing of the report; and the decision to submit the report for publication, including whether they will have ultimate authority over any of these activities	None_________

For peer review only

	5d	Composition, roles, and responsibilities of the coordinating centre, steering committee, endpoint adjudication committee, data management team, and other individuals or groups overseeing the trial, if applicable (see Item 21a for data monitoring committee)	Coordinating centre staff (chief investigator, trial manager, trial administrator, qualitative researcher) will oversee the trial in collaboration with the Oxford trials unit (OCTRU), the co-applicants, the sites, the sponsor, oversight committees (DSMC, TSC and TMG) and regulatory bodies.
**Introduction**			
Background and rationale	6a	Description of research question and justification for undertaking the trial, including summary of relevant studies (published and unpublished) examining benefits and harms for each intervention	4,5__________
	6b	Explanation for choice of comparators	4,5___________
Objectives	7	Specific objectives or hypotheses	5____________
Trial design	8	Description of trial design including type of trial (eg, parallel group, crossover, factorial, single group), allocation ratio, and framework (eg, superiority, equivalence, noninferiority, exploratory)	6____________
**Methods: Participants, interventions, and outcomes**			
Study setting	9	Description of study settings (eg, community clinic, academic hospital) and list of countries where data will be collected. Reference to where list of study sites can be obtained	2, 25, 26____

For peer review only

Eligibility criteria	10	Inclusion and exclusion criteria for participants. If applicable, eligibility criteria for study centres and individuals who will perform the interventions (eg, surgeons, psychotherapists)	6, 7, ___
Interventions	11a	Interventions for each group with sufficient detail to allow replication, including how and when they will be administered	7, 8, 9________
	11b	Criteria for discontinuing or modifying allocated interventions for a given trial participant (eg, drug dose change in response to harms, participant request, or improving/worsening disease)	9 _______
	11c	Strategies to improve adherence to intervention protocols, and any procedures for monitoring adherence (eg, drug tablet return, laboratory tests)	8,9,15, ___
	11d	Relevant concomitant care and interventions that are permitted or prohibited during the trial	9, 10_________
Outcomes	12	Primary, secondary, and other outcomes, including the specific measurement variable (eg, systolic blood pressure), analysis metric (eg, change from baseline, final value, time to event), method of aggregation (eg, median, proportion), and time point for each outcome. Explanation of the clinical relevance of chosen efficacy and harm outcomes is strongly recommended	2, 3, 5, 12, 13, 14 _____________
Participant timeline	13	Time schedule of enrolment, interventions (including any run-ins and washouts), assessments, and visits for participants. A schematic diagram is highly recommended (see Figure)	12, 13________
Sample size	14	Estimated number of participants needed to achieve study objectives and how it was determined, including clinical and statistical assumptions supporting any sample size calculations	14, 15________
Recruitment	15	Strategies for achieving adequate participant enrolment to reach target sample size	9,15 ___________

Methods: Assignment of interventions (for controlled trials)

Allocation:

Sequence generation	16a	Method of generating the allocation sequence (eg, computer-generated random numbers), and list of any factors for stratification. To reduce predictability of a random sequence, details of any planned restriction (eg, blocking) should be provided in a separate document that is unavailable to those who enrol participants or assign interventions	10__________

Allocation concealment mechanism	16b	Mechanism of implementing the allocation sequence (eg, central telephone; sequentially numbered, opaque, sealed envelopes), describing any steps to conceal the sequence until interventions are assigned	10__________
Implementation	16c	Who will generate the allocation sequence, who will enrol participants, and who will assign participants to interventions	7, 10_________
Blinding (masking)	17a	Who will be blinded after assignment to interventions (eg, trial participants, care providers, outcome assessors, data analysts), and how	6, 9, 10,18_______
	17b	If blinded, circumstances under which unblinding is permissible, and procedure for revealing a participant's allocated intervention during the trial	n/a__________

Methods: Data collection, management, and analysis

Data collection methods	18a	Plans for assessment and collection of outcome, baseline, and other trial data, including any related processes to promote data quality (eg, duplicate measurements, training of assessors) and a description of study instruments (eg, questionnaires, laboratory tests) along with their reliability and validity, if known. Reference to where data collection forms can be found, if not in the protocol	9, 11, 13, 14, 15, 16, 17
	18b	Plans to promote participant retention and complete follow-up, including list of any outcome data to be collected for participants who discontinue or deviate from intervention protocols	16
Data management	19	Plans for data entry, coding, security, and storage, including any related processes to promote data quality (eg, double data entry; range checks for data values). Reference to where details of data management procedures can be found, if not in the protocol	16, 17_________
Statistical methods	20a	Statistical methods for analysing primary and secondary outcomes. Reference to where other details of the statistical analysis plan can be found, if not in the protocol	17, 18, 19, 20
	20b	Methods for any additional analyses (eg, subgroup and adjusted analyses)	18__________
	20c	Definition of analysis population relating to protocol non-adherence (eg, as randomised analysis), and any statistical methods to handle missing data (eg, multiple imputation)	17, 19

Methods: Monitoring

Data monitoring	21a	Composition of data monitoring committee (DMC); summary of its role and reporting structure; statement of whether it is independent from the sponsor and competing interests; and reference to where further details about its charter can be found, if not in the protocol. Alternatively, an explanation of why a DMC is not needed	20, 26. The DSMC in independent from the sponsor and all members will provide competing interest statements.
	21b	Description of any interim analyses and stopping guidelines, including who will have access to these interim results and make the final decision to terminate the trial	16____________
Harms	22	Plans for collecting, assessing, reporting, and managing solicited and spontaneously reported adverse events and other unintended effects of trial interventions or trial conduct	20____________
Auditing	23	Frequency and procedures for auditing trial conduct, if any, and whether the process will be independent from investigators and the sponsor	Subject to audit by national bodies, the trials unit and the sponsor
**Ethics and dissemination**			
Research ethics approval	24	Plans for seeking research ethics committee/institutional review board (REC/IRB) approval	3______________
Protocol amendments	25	Plans for communicating important protocol modifications (eg, changes to eligibility criteria, outcomes, analyses) to relevant parties (eg, investigators, REC/IRBs, trial participants, trial registries, journals, regulators)	Once approved by sponsor/REC/HRA as appropriate, important modifications will be communicated to site research teams and R&D offices by email.

For peer review only

Consent or assent	26a	Who will obtain informed consent or assent from potential trial participants or authorised surrogates, and how (see Item 32)	Consent will be obtained by those delegated the task by the local PI at the site.
	26b	Additional consent provisions for collection and use of participant data and biological specimens in ancillary studies, if applicable	11____________
Confidentiality	27	How personal information about potential and enrolled participants will be collected, shared, and maintained in order to protect confidentiality before, during, and after the trial	16, 17________
Declaration of interests	28	Financial and other competing interests for principal investigators for the overall trial and each study site	23____________
Access to data	29	Statement of who will have access to the final trial dataset, and disclosure of contractual agreements that limit such access for investigators	17_____________
Ancillary and post-trial care	30	Provisions, if any, for ancillary and post-trial care, and for compensation to those who suffer harm from trial participation	11____________
Dissemination policy	31a	Plans for investigators and sponsor to communicate trial results to participants, healthcare professionals, the public, and other relevant groups (eg, via publication, reporting in results databases, or other data sharing arrangements), including any publication restrictions	21____________
	31b	Authorship eligibility guidelines and any intended use of professional writers	Described in trial Publication Plan
	31c	Plans, if any, for granting public access to the full protocol, participant-level dataset, and statistical code	n/a_____________ _

Appendices

For peer review only - http://bmjopen.bmj.com/site/about/guidelines.xhtml

Informed consent materials	32	Model consent form and other related documentation given to participants and authorised surrogates	Not included in this submission – available on request ______________
Biological specimens	33	Plans for collection, laboratory evaluation, and storage of biological specimens for genetic or molecular analysis in the current trial and for future use in ancillary studies, if applicable	n/a_____________ _

*It is strongly recommended that this checklist be read in conjunction with the SPIRIT 2013 Explanation & Elaboration for important clarification on the items. Amendments to the protocol should be tracked and dated. The SPIRIT checklist is copyrighted by the SPIRIT Group under the Creative Commons "Attribution-NonCommercial-NoDerivs 3.0 Unported" license.

For peer review only

BMJ Open

The Osteoarthritis Thumb Therapy Trial (OTTER II): A study protocol for a three arm multi centre randomised placebo controlled trial of the clinical effectiveness and efficacy and cost effectiveness of splints for symptomatic thumb base osteoarthritis

Journal:	*BMJ Open*
Manuscript ID	bmjopen-2018-028342.R1
Article Type:	Protocol
Date Submitted by the Author:	20-Mar-2019

Complete List of Authors:	Adams, Jo; University of Southampton, Faculty of Environment and Life Sciences<br>Barratt, Paula ; University of Southampton, Faculty of Environment and Life Sciences<br>Arden, Nigel; Nuffield Department of Orthopaedics, ;<br>Barbosa Bouças, Sofia; Brunel University , Department of Life Sciences<br>Bradley, Sarah; Poole Hospital NHS Foundation Trust<br>Doherty, Michael ; University of Nottingham<br>Dutton, Susan; University of Oxford, CSM<br>Dziedzic, Krysia; Arthritis Research Campaign National Primary Care Centre<br>Gooberman-Hill, Rachael; University of Bristol, Bristol Medical School<br>Hislop Lennie, Kelly; University of Southampton, Faculty of Environment and Life Sciences<br>Hutt Greenyer , Corinne; University of Southampton, Faculty of Environment and Life Sciences<br>Jansen , Victoria; Royal Derby Hospital, Pulvertaft Hand Centre<br>Luengo-Fernandez, Ramon; University of Oxford, Health Economics Research Centre<br>Meagher, Claire ; University of Southampton, Faculty of Environment and Life Sciences<br>White, Peter; University of Southampton, Faculty of Environment and Life Sciences<br>Williams, Mark; Oxford Brookes University, Sport, Health Sciences and Social Work

**Primary Subject Heading**:	Rehabilitation medicine
Secondary Subject Heading:	Rheumatology
Keywords:	Osteoarthritis, REHABILITATION MEDICINE, RHEUMATOLOGY, Efficacy trial

The Osteoarthritis Thumb Therapy Trial (OTTER II): A study protocol for a three arm multi centre randomised placebo controlled trial of the clinical effectiveness and efficacy and cost effectiveness of splints for symptomatic thumb base osteoarthritis

Corresponding author: Jo Adams. School of Health Sciences, Faculty of the Environement and Life Sciecnes, Building 67, University of Southampton, Highfield Campus, University Road, Southampton, SO17 1BJ, UK. Tel: + 44 (0)23 8059 5287. E-mail: ja@soton.ac.uk

Authors:

Jo Adams School of Health Sciences, University of Southampton, Southampton, UK

Paula Barratt School of Health Sciences, University of Southampton, Southampton, UK

Nigel Arden Nuffield Department of Orthopaedics, Rheumatology and Musculoskeletal Sciences (NDORMS), University of Oxford, Oxford, UK

Sofia Barbosa Bouças Dept of Life Sciences, Brunel University London, Uxbridge, UK

Sarah Bradley Hand Therapy Unit, Poole Hospital NHS Trust, Poole, UK

Michael Doherty School of Medicine, University of Nottingham, Nottingham, UK

Susan Dutton Oxford Clinical Trials Research Unit, University of Oxford, Oxford, UK

Krysia Dziedzic Research Institute for Primary Care and Health Sciences, Keele University, Keele, UK

Rachael Gooberman-Hill Elizabeth Blackwell Institute for Health Research, University of Bristol, Bristol, UK

Kelly Hislop School of Health Sciences, University of Southampton, Southampton, UK

Corinne Hutt Greenyer School of Health Sciences, University of Southampton, Southampton, UK

Victoria Jansen Pulvertaft Hand Centre, Royal Derby Hospital, Derby, UK

Ramon Luengo Fernandez Nuffield Dept of Population Health, University of Oxford, Oxford, UK

Claire Meagher School of Health Sciences, University of Southampton, Southampton, UK

Peter White School of Health Sciences, University of Southampton, Southampton, UK

Mark Williams Dept of Sport, Health Sciences and Social Work, Oxford Brookes University, Oxford, UK

On behalf of the OTTER II collaborative group

Word count: 4467 (excl abstract and tables)

For peer review only - http://bmjopen.bmj.com/site/about/guidelines.xhtml

Abstract

Introduction

The economic cost of osteoarthritis (OA) is high. At least 4.4 million people have hand OA in the UK. Symptomatic thumb base OA affects 20% of people over 55 years, causing more pain, work and functional disability than OA elsewhere in the hand. Most evidence-based guidelines recommend splinting for hand OA. Splints that support or immobilise the thumb base are routinely used despite there being limited evidence on their effectivenss. The potential effects of placebo interventions in OA are acknowledged, but few studies investigate the clinical efficacy of rehabilitation interventions nor the impact of any placebo effects associated with splints.

Methods and Analysis

Participants aged 30 years and over with symptomatic thumb base OA will be recruited into the trial from secondary care occupational therapy and physiotherapy centres. Following informed consent, participants will complete a baseline questionnaire and then be randomised into one of three treatment arms: a self-management programme, a self-management programme plus a verum thumb splint, or a self-management programme plus a placebo thumb splint. The primary outcome is the AUSCAN hand pain scale. The study end point is 8 weeks after baseline. Baseline assessments will be carried out prior to randomisation and outcomes collected at 4; 8 and 12 weeks. Cost effectiveness analysis will be conducted and individual qualitative interviews conducted with up to 40 participants after 8 weeks to explore perceptions and outcome expectations of verum and placebo splints and exercise.

Ethics and Dissemination

South Central - Oxford C Research Ethics Committee approved this study (16/SC/0188). The findings will be disseminated to health professional conferences, journals and lay publications for patient organisations. The research will contribute to improving the management of thumb base OA and help clinicians and patients make informed decisions about the value of different interventions.

Trial Registration Number: ISRCTN 54744256

For peer review only - http://bmjopen.bmj.com/site/about/guidelines.xhtml

Key words: Osteoarthritis; thumb base; splint; hand pain; hand function; placebo; self-management.

Strengths and Limitations

1. This trial is powered to evaluate the clinical benefit and statistical significance of adding splinting to a self-management programme for people with thumb base OA.

2. This is the first trial to use a placebo thumb splint intervention and will add to the understanding of contextual aspects of such visible physical treatment and self-management programmes for OA.

3. The trial has been informed and designed with the input of patients and expert clinicians and the trial outcome measures have been agreed as meaningful by patients.

4. The 12 week trial follow-up period is limited and longer term follow-up would provide further useful outcome data.

For peer review only - http://bmjopen.bmj.com/site/about/guidelines.xhtml

INTRODUCTION

Osteoarthritis (OA) is a leading cause of pain, disability, healthcare utilisation, and productivity loss in the UK. Each year approximately two million adults visit their GP with symptoms of OA [1]. Osteoarthritis is more prevalent in women, the incidence increases with age and recent estimates suggest more than 150 million Europeans have radiographic hand OA and 15 million have symptomatic arthritis [2]. Symptomatic thumb base (1st carpometacarpal and/or scaphotrapezial joint) OA affects approximately 22% of people aged 50 years and over [3].Thumb base OA is likely to become more prevalent in the future since the incidence increases with age [4] and is identified as a priority for treatment as it causes more pain and disability and is associated with a worse prognosis than other hand sites affected by OA [5].

Patients with thumb base OA present with predominantly mechanical usage-related pain over the thumb base [6] and are more likely to have more pain, work disability and reduction in quality of life and function, and to receive more anti-inflammatory drugs and more splinting than participants with OA affecting other hand joints[5] [7] [8] [9]

Thumb base OA affects entire hand function [10] and the overall impact, particularly in older people, can be substantial, with many experiencing difficulties with daily household, caring, work and leisure activities [11]. Despite the scale of this problem, it appears that both patients and practitioners often believe that there is little that can be done [12].

Therapeutic splinting for thumb base OA aims to minimise or eliminate motion at the thumb carpometacarpal joint [13] in order to prevent joint deterioration and/or deformity, decrease pain, and increase overall hand function [14]. The process of designing thumb splints currently lacks detailed reporting but biomechanical principles have been applied to one design to off load the dynamic forces occurring during functional hand use on a symptomatic carpometacarpal joint [15]. It is known that stabilisation of the carpometacarpal joint to reduce pain levels does impacts on hand functionality [16] Currently, thumb splints are recommended for patients with thumb base OA [17]. However, the efficacy of splinting based on this approach has not yet been established and forms the rationale for this trial. Some evidence based on small, non-powered samples [18 19] show thumb

For peer review only - http://bmjopen.bmj.com/site/about/guidelines.xhtml

splints can help relieve pain however, evidence to support their effectiveness is not yet fully supported by robust research [20]. There is insufficient evidence to suggest that the combination of splinting delivered alongside hand exercises is more effective than hand exercises alone [21] [22] [23]. To date, there has also been no examination of any contextual and non-specific patient-practitioner interaction effects associated with splinting in thumb base OA. If this is substantial, optimising the non-specific effects of treatment could improve treatment effectiveness and overall care of people with OA in a safe and cost effective way [24 25]. This randomised controlled trial (RCT) protocol, was designed in accordance with the SPIRIT checklist [26], to evaluate the effectiveness, efficacy and cost effectiveness of thumb splints for people with thumb base OA.

The primary objective of this trial is to determine the clinical effectiveness and efficacy of thumb splints when added to a self-management programme for people with symptomatic thumb base OA. This trial questions:

1. Is there a benefit of adding a thumb base splint to a self-management programme for people with thumb base OA?

2. Is there a difference in benefit between adding a verum or a placebo thumb base splint to a self-management programme for people with thumb base OA?

3. What are patients' views and experiences of the effectiveness, acceptability and adherence to the trial interventions?

METHODS AND ANALYSIS

Patient and Public Involvement

Exploring the effectiveness of orthotic devices for use by patients living with osteoarthritis was identified as a priority area for investigation by an Arthritis Research UK patient stakeholder committee. Two patient and public group meetings were carried out with 8 patient partners living with hand OA to listen to patient experiences of living with hand OA, explore intervention components of the trial and help to inform co-design a placebo splint design [27]. Patient partners discussed with the team what splints they thought should be included in a trial, which splint designs that found most credible and what outcomes were important to them in their daily lives. A national

For peer review only - http://bmjopen.bmj.com/site/about/guidelines.xhtml

Delphi study with therapy clinicians and patients was conducted, to inform and agree trial processes [28 29]. Clinicians tended to want more focused time efficient measures where patients usually hoped for more comprehensive intervention that was beyond the time allocated to NHS out-patient provision. This helped to consider treatment burden from both clinical and patient's view points. This involvement at an early stage ensured that clinicians felt that they had contributed and had ownership in the trial design and patient input ensured that patients' perspectives had been integral to the study design hopefully making the trial relevant and appealing to prospective patients. We conducted a focus group to explore the support required by NHS therapy clinicians when knowingly delivering placebo splints was completed [30]. A pilot study across five NHS recruitment sites to test recruitment and procedural feasibility and safety and the convincing delivery of a newly designed placebo splint was conducted and reported [31]. The education and support needs identified by therapy clinicians taking part in a placebo controlled RCT was sought and recorded [32]. This informed the trial training for clinicians. Our patient partner and co-author (CHG) provided a patient's perspective in developing recruitment strategies, study conduct and lay dissemination routes.

Trial Design and Setting

OTTER II is a pragmatic, multi-centred, single (participant) blind, superiority randomised controlled clinical effectiveness and efficacy trial. People with symptomatic thumb base OA reporting moderate to severe thumb base pain, will be equally allocated to one of three groups: Group A: Eight weeks of a self-management programme, Group B: Eight weeks of a self-management programme plus a verum splint, or Group C: Eight weeks of a self-management programme plus a placebo splint. Follow up for all groups will be for 12 weeks from baseline. The study will run from 28th February 2017 to 14th March 2019

Participants will be recruited consecutively from referrals to Occupational Therapy or Physiotherapy (Therapy) departments from new, current and review patients of Rheumatology, Orthopaedic, Hand Surgery Units and General Practice at 17 NHS recruitment sites (Appendix 1). Intervention will be delivered by a qualified OTTER II trained occupational therapist or physiotherapist who has worked

For peer review only - http://bmjopen.bmj.com/site/about/guidelines.xhtml

independently in a clinical role treating patients with hand OA and who works within a therapy department that accepts referrals for thumb base OA patients.

Participants

Participant inclusion criteria were decided upon iteratively with our rheumatologists, clinicians and hand surgeons in relation to the literature about prevalence, incidence rates and current treatment by therapy departments in the UK. Clinical tests, that contributed to inclusion criteria, were selected following: review of literature; consideration of the sensitivity and specificity of each test; examination of the practicality and feasibility of the testing procedure for the collaborating clinicians working across different UK hospitals. As there is no clear consensus on the longevity of the impact of intra articular steroid injections into the 1st CMCJ [33] [34] [35] we used local clinical NHS protocols relating to the length of time suggested to repeat 1st CMCJ steroid injections, to inform our inclusion criteria.

Consecutive potential participants will be screened and assessed for recruitment into the trial by the collaborating OTTER II trial trained therapy clinicians using inclusion and exclusion criteria as shown in Table 1. The charactieristics of participants who fulfil inclusion criteria and who decline to take part will be recorded.

**Inclusion Criteria**	
1	Aged 30 years and over.
2	At least moderate hand pain (AUSCAN [36] hand pain score >5) and moderate functional hand disability (AUSCAN [36]hand functional disability score >9).
3	Show signs and symptoms of thumb base OA on clinical enquiry and examination, specifically: hard tissue enlargement of the first carpometacarpal joint (CMCJ) OR squaring of the thumb base OR pain that worsens when pinching OR pain that worsens on span grip (e.g. opening a jar) OR crepitus on movement OR reduction in thumb base range of movement OR positive thumb adduction provocation test [37] OR positive thumb extension provocation test [37] OR pain on palpation of the dorso-radial aspect of the thumb CMCJ.
4	No other household member participating in the trial.
5	Able to give written informed consent.

For peer review only - http://bmjopen.bmj.com/site/about/guidelines.xhtml

6	Available to attend Occupational Therapy/Physiotherapy/Hand Therapy sessions.
**Exclusion Criteria**	
**1**	Consultation with therapy department or treatment for this thumb problem (excluding pain killers and anti-inflammatories) in the previous six months.
**2**	Intra-articular joint injection to wrist, fingers or thumb in the previous two months.
**3**	Fractures or significant injury or surgery to the wrist or hand within the previous six months.
**4**	Red flags i.e. history of serious illness or disease. i.e. any other diagnosed rheumatic conditions: gout, psoriatic arthritis, ankylosing spondylitis, connective tissue disorders (systemic lupus, systemic sclerosis), resulting in inflammatory arthritis in the hand/s, or, progressive neurological signs, or acute swollen hand joint.
**5**	Diagnosis of dementia or other significant disorder likely to affect communication.
**6**	Already received thumb splints for thumb base OA.
**7**	Skin disease that may interfere or contraindicate splint wear.
**8**	Participant of a drug or medical device trial in the last 12 weeks.

Table 1. OTTER II Trial patient inclusion and exclusion criteria

Interventions

The trial's three intervention arms all include 90 minutes of direct therapy intervention delivered by a qualified OTTER II trained occupational therapist or physiotherapist who has worked independently in a clinical role treating patients with hand OA and who works within a therapy department that accepts referrals for thumb base OA patients.

The direct therapy intervention will be delivered in a 60- minute baseline appointment at an NHS secondary care hospital or clinic, when the interventions listed below will be delivered. A 30-minute follow up intervention at week four will be conducted, where progress is reviewed and any necessary adjustments made. A final third hospital visit at week eight is for finalisation of trial procedures only and includes no direct therapy intervention.

The three intervention arms, delivered at the baseline appointment are:

For peer review only - http://bmjopen.bmj.com/site/about/guidelines.xhtml

Group A - An optimal self-management programme

The self-management programme (Appendix 2) includes:

1. Teaching standardised hand exercises (developed from an evidence based review [38]and provision of a trial-specific hand exercise booklet.

2. Provision of a trial-specific booklet about joint protection, activity pacing and general advice about OA followed by a discussion with the therapist of the content.

3. Provision of the Arthritis Research UK Osteoarthritis information booklet.

4. A discussion with the therapist of the facilitators and barriers to engaging with self-management principles.

5. A patient hand exercise diary.

Group B - Optimal self-management programme plus a verum thumb base splint

Group B participants receive:

1. The optimal self-management programme as detailed above in Group A.

2. A verum splint. Therapists and participants will be given an option of two different splints, with the choice guided by a standardised Splint Decision Protocol (Appendix 3).

The splinting options informed by the study's design and development stage will be:

- Procool Thumb CMC Restriction splint (black) (Ref PTRS).

Or

- Orfilight 2.5mm 3/32" micro perforated (beige) trouser leg splint (custom made by the therapist from a pre-cut standardised trial template and standardised strapping protocol).

Both splints will be delivered with instructions on how to wear and use the splint (Appendix 4). The Procool thumb CMC restriction splint comes in packaging with a label providing details of the manufacturer and washing instructions.

3. A discussion with the therapist of the facilitators and barriers to engaging with splint wear (Appendix 5).

4. A patient splint wear diary (Appendix 6).

For peer review only - http://bmjopen.bmj.com/site/about/guidelines.xhtml

Group C - Optimal self-management programme plus a placebo thumb base splint

Group C participants receive:

1. The self-management programme as detailed above in Group A.

2. A placebo splint. There will be the choice of two designs of placebo splint. These are made in a lightweight nylon, secured around the wrist and have phalangeal components but no basal thumb joint support. One is black and the other is beige to match the verum splint options and three sizes will be manfucatured. They have been designed with no known active component and none was detectable during testing [39]. A standardised Splint Decision Protocol about which splint will be most appropriate to issue in which situation will be used (Appendix 7). The placebo splints arrive in packaging with a label providing details of the manufacturer, washing instructions, and a lifestyle education leaflet about how to position and wear the splint. Both splints will be delivered with instructions on how to wear and use the splint.

3. An information sheet to outline when the splint should be worn (Appendix 4)

4. A discussion with the therapist of the facilitators and barriers to engaging with splint wear. (Appendix 5).

5. A patient splint wear diary (Appendix 6)

Trained OTTER II clinicians will deliver a standardised self management package of care using their clinical judgement to apply the content for each individual patient. Participants in group A will receive more time spent on self management than participants in group B and C.

Concomitant care

Any relevant contralateral thumb treatment for participants will be delayed until the end of the trial. There will be no alteration in participants' general concomitant care whilst on the trial, . Additional treatments e.g. joint injection, surgery and reported purchase or use of own splints during the study period will be captured on self-report questionnaires. Criteria for modifying allocated interventions is detailed within the OTTER II Trial Safety Information.

Investigator Training

For peer review only - http://bmjopen.bmj.com/site/about/guidelines.xhtml

Trial training visits will be conducted by the OTTER II trial management team (JA, PB, PW) to carry out standardised instruction and demonstration for OTTER II research clinicians prior to the start of patient recruitment. Training will cover procedures for maintaining clinician blinding, delivery of eligibility tests, interventions, maintaining participant blinding, reporting of safety events, data entry and use of Case Report Forms (CRFs). Sites will also be visited by the trial team (JA, MW, CM) to provide support and guidance on maximising participant recruitment through liaison with surgery and community health teams, and to conduct quality assurance evaluations of intervention delivery as required.

A page of the trial website which is only available to site staff (not participants or the public) will provide training videos on the standardised delivery of all the trial interventions. All clinicians will complete the National Institute of Health Research Good Clinical Practice (GCP) training inorder to have the skills and knowledge necessary to comply with the international ethical, scientific and practical standards to which all clinical research is conducted. Compliance with GCP provides public assurance that the rights, safety and wellbeing of research participants are protected and that research data are reliable. [40].

Participant identification and baseline assessment

Potential participants will receive a letter about the study and a Participant Information Sheet at the hospital clinic or by post. For interested patients an out-patient therapy department appointment will be made at Week 0 (Baseline). Following eligibility screening, formal written consent will be sought and given and participants will complete the baseline assessments. Consent will be taken by an NHS therapist trained in OTTER II Trial procedures.

Randomisation

Eligible patients will be enrolled into the study via the Oxford Clinical Trials Research Unit (OCTRU) online randomisation service and this system will record eligibility and stratification data.

When participants are randomised to a treatment arm this constitutes the date of start of treatment. Participants will only be randomised once and for the treatment of one thumb. For participants with symptoms of bilateral thumb base OA the most painful thumb will be treated first within the trial. If

For peer review only - http://bmjopen.bmj.com/site/about/guidelines.xhtml

at the end of the trial participants require treatment on a contralateral thumb then participants will be invited back by the clinical service to undergo routine (i.e. non-trial) clinical intervention at the end of the trial.

Randomisation will be on a 1:1:1 basis. The first 30 participants will be randomised using a simple random list with varying block sizes to seed the subsequent minimisation algorithm. This random list is generated by the trial statistician and concealed from all other members of the trial team to ensure future treatment allocations cannot be predicted. Subsequent participants will be randomised using a validated computer randomisation program (RRAMP) with a minimisation algorithm, which will ensure balanced treatment allocations across the stratification factors (centre, baseline AUSCAN hand pain score (Category 1= AUSCAN pain score 6 to 12 and Category 2= AUSCAN hand pain score 13 to 20) and treated hand dominance). The minimisation system includes a random element (0.8) to minimise predictability of treatment allocations for new participants.

Participants will be blind to treatment allocation. Strategies to maximise participant blinding include, training for trial clinicians in how to present and discuss the treatment arms positively without disclosing other treatment options and routine assessment included in the quality assurance trial monitoring visits of clinician's communication and interaction in presenting treatment options with participants. The Participant Information Sheet (PIS) has been carefully worded to state that an element of the self- management intervention may be placebo but does not state what aspect this may be. The study will be described as a comparison of self-management interventions and not divulging detail about placebo splinting in any trial of clinician communication. Trial participants will not receive group intervention and therefore cannot compare their interventions received and we will not include participants who have already received thumb base splints. All of these strategies will help to maintain participant blinding. Contamination between treatment arms will be limited by identifying on hospital notes that the patient is part of the OTTER II Trial and therefore should not receive any further hand therapy treatment from the hospital team. It is not possible to prevent participants allocated to Arm A or B purchasing their own splints during the trial period, however, the data will be captured in the final study questionnaire and will be used to assess any degree of contamination.The OTTER II placebo splints will not be able to purchased privately.

For peer review only - http://bmjopen.bmj.com/site/about/guidelines.xhtml

This approach will support participant blinding, in addition to the training that treating therapists will receive about delivering placebos and responding to participant questions. The success of participant blinding will be assessed via patient questionnaires at 12 weeks.

Treatment and follow up

Figure 1 flowchart shows the participants' progress through the trial. After randomisation participants will receive their allocated intervention during a 60 minute appointment (week 0). All participants in each treatment arm will receive a 10 minute telephone call at two weeks from the therapist to check for adherence to the self-management programme and to discuss any problems identified by the participant as relevant. In addition for Group B and C participants the therapist will check splint wear and comfort. Participants will attend for a 30 minutes appointment 4 weeks after randomisation, to review treatments and make adjustments where necessary and an assessor blind to treatment allocation will administer the objective Grip Ability Test [41] (final hospital visit occurs 8 weeks after randomisation. A paper questionnaire will be posted to each participant at 12 weeks to collect follow-up patient reported outcomes. After the 12 week follow-up has been completed (study end point) all patients will be offered the option of a further follow up appointment for any further care that is needed. If patients have received verum splints then they may keep these, if patients have received placebo splints or self maangement alone then they will be offered the opportunity to receive a verum splint if required.

Qualitative Interviews

Between weeks 8 and 12 a qualitative telephone interview will take place for a subgroup of patients in intervention arm B and C. The researcher will be blinded for the qualitative interviews in order to prevent bias related to treatment allocation during data collection. In order to make this practical, the researcher carrying out data collection will be independent from the RCT and will be unfamiliar with the splints and differences between them. The methodological approach for the qualitative study will be a framework analysis. Purposive sampling will be used and based on male to female

For peer review only - http://bmjopen.bmj.com/site/about/guidelines.xhtml
For peer review only

ratio (as per the study sample); age and AUSCAN hand pain index at baseline. Interviews will be conducted with up to 40 participants. Interviews will be semi-structured, using a topic guide informed by patient partners, to elicit experience of splint and self-management interventions, splint preference, adherence and views about reasons for effectiveness. There will be a password for each participant that has agreed to take part in the interview study so that the participant can be correctly identifed on the telephone. Consent for participation in the qualitative interview will be sought and given at the time of entry into the main study.

The schedule of enrolment, interventions and a summary of assessments are shown in **Table 2**. Detailed information about baseline assessments and outcome measures are listed in **Table 3.**

For peer review only

For peer review only - http://bmjopen.bmj.com/site/about/guidelines.xhtml

	STUDY PERIOD							
	Enrolment	Allocation	Post-allocation					Close-out
TIMEPOINT:	Baseline Day 0	Baseline Day 0	2 wks	4 wks	8 wks	9-11 wks	12 wks	After 12 wks
ENROLMENT:								
Eligibility screen*	X							
Informed consent*	X							
Randomisation/ Allocation		X						
INTERVENTIONS:								
Optimal NHS self-management care		X	—	—	—			
Optimal NHS self-management care plus verum splint		X	—	—	—			
Optimal NHS self-management care plus placebo splint		X	—	—	—			
Telephone call check of progress			X					
Review of self-management care and splint wear (if applicable)				X				
ASSESSMENTS:								
Baseline Assessments: See Table 3	X							
Outcomes: Grip Ability Test [41]				X	X			
Outcomes: See Table 3					X			
Outcomes at follow up: See Table 3							X	

For peer review only - http://bmjopen.bmj.com/site/about/guidelines.xhtml

**Exercise adherence**		◆———	———	———	———	———	———◆	
**Splint adherence**		◆———	———	———	———	———	———◆	
**Qualitative Interviews (selected participants)**						X		
**Patient request for further treatment**								X

Table 2. Schedule of enrolment, interventions, and assessments

*The eligibility screen and informed consent can optionally be carried out prior to time point 0.

**Measure**	**Screening**	**Baseline**	**4 weeks**	**8 weeks**	**12 weeks**
**Work Productivity and Activity Impairment Questionnaire (WPAI)** [42]		✓		✓	✓
**Generic Quality of Life (SF12-V2)** [43]		✓		✓	✓
**EuroQol 5 Dimensions 5-Levels questionnaire (EQ5D-5L)** [44]		✓		✓	✓
**AUSCAN hand stiffness** [36]		✓		✓	✓
**Michigan Hand Questionnaire (MHQ)** [45]		✓		✓	✓
**Thumb pain over the last week**		✓		✓	✓
**Disability of the arm, shoulder, hand questionnaire (DASH)** [46]		✓		✓	✓

For peer review only - http://bmjopen.bmj.com/site/about/guidelines.xhtml

For peer review only

**Leisure section only**					
**Arthritis Self-Efficacy Pain Scale** [47]		✓		✓	✓
**AUSCAN hand pain** [36]	✓			✓	✓
**AUSCAN hand function** [36]	✓			✓	✓
**Global assessment of change** [48]				✓	✓
**Health utilisation questionnaire**				✓	✓
**The objective clinician assessed Grip Ability Test (GAT)** [41]		✓	✓	✓	

Table 3. Baseline assessment and outcome measures.

The Global Assessment of Change [48] question adapted for the OTTER II study asks "With respect to your thumb base pain how would you describe yourself now as compared to the start of your OTTER trial therapy treatment" The answer is given on a 5 point Likert scale, that that response range from "very much worse", "worse", over "no change" to "better" and "completely recovered". The AUSCAN hand index for pain [36] recorded at baseline and 8 weeks is the primary outcome measure. The CRFs for this study can be obtained from the first author (JA)

Sample Size

The sample size has been calculated based on undertaking a global Analysis of Covariance (ANCOVA) for the primary outcome, AUSCAN hand pain [36] at 8 weeks, adjusting for the baseline pain score and stratification factors (including centre and treated hand dominance) across all three treatment arms using the power and sample size package, PASS 11 (PASS 11. NCSS, LLC. Kaysville, Utah, USA. www.ncss.com). Assuming 80% power, a 5% 2-sided significance in order to detect a standardised mean difference of 0.4 (a moderate effect size [49]based on a difference in the AUSCAN hand pain score of 2 points and assuming a standard deviation of 5, based on data from the OTTER pilot study

For peer review only

For peer review only - http://bmjopen.bmj.com/site/about/guidelines.xhtml

[31], requires 92 participants per arm. Allowing a 20% loss to follow-up at 8 weeks inflates this to 115 participants per arm, giving a total of 345 participants.

The sample size has taken into account the global comparison of the null hypothesis that there is no difference between the three treatment arms, and pairwise comparisons will only be undertaken if this global comparison is statistically significant. No further adjustment for the sample size has been undertaken to allow for multiple testing.

Clustering is not a consideration in sample size as "Centre" is a stratification factor, and although there may be more than one therapist per centre, this should largely ensure balance across treatment arms within each centre. The Data and Safety Monitoring Committee (DSMC) will review the assumptions underlying this sample size calculation after approximately 50% of the participants have been recruited and followed up for 8 weeks, if still within the recruiting period.

Recruitment strategy

Recruitment targets and procedures have been tested in the pilot study [31] and are based on these data. Recruitment start dates for the collaborating sites will be staggered over a 6-month period.

Data collection, management and analysis

The trial will use a series of CRFs to record trial activities and RCT site staff will ensure that each CRF is completed properly and stored in the Investigator's Site File (ISF). Site staff will make photocopies and post CRFs to the OTTER Trial team using FREEPOST envelopes, according to a standard operating procedure (SOP), so that both sites and the trial centre will have a copy of CRFs.

Participants will complete questionnaires at baseline, 8 weeks and 12 weeks. Participants will be asked to record the frequency for which hand exercises were completed for at least 20 minutes using a daily diary. Splint wear diaries will be used to capture hours per day of splint wear and will contribute to per protocol analysis of data. Participants will be provided with FREEPOST envelopes in order to return questionnaires and diaries to the trial centre.

For peer review only - http://bmjopen.bmj.com/site/about/guidelines.xhtml

Where postal follow-up questionnaires are not received when expected, first they will be chased by post, and if there is no response key endpoint data will be collected over the phone. Up to three phone calls will be made to obtain key endpoint data. If participants wish to withdraw from their randomised intervention, they will still be included in the trial follow-up, unless they request to be excluded from follow-up. Where available, reasons for withdrawal from follow-up will be collected.

The OTTER Research Team will make copies of CRFs, questionnaires and diaries created by or received directly at the trial centre and post them to the relevant site to be filed in the ISF. The original documents will be secured in the Trial Master File.

Eligibility and stratification data will be entered directly onto the OCTRU online randomisation system by site staff. All other data will be entered into the trial database by the OTTER Research Team. The OTTER trial team will check each CRF for completeness and where appropriate contact the site for missing data. A Data Management and Sharing Plan contains fully comprehensive information about all aspects of data management. All data will be processed according to the Data Protection Act 2018. All study specific documents, except for the signed consent form and letters to participants, will refer to the participant with a unique study participant number/code and not by name.

Quantitative data will be stored on an OpenClinica trial-specific database prepared and managed by OCTRU. Qualitative data will be stored in QRS NVivo and word documents. The database has inbuilt data validation checks and a trial management system for managing data queries. Peer review of data entry will be conducted on 5% of CRFs. Data discrepancies will be reviewed on a regular basis to help clean the data. All data will be securely stored only accessible by authorised personnel agreed by the Principal Investigator, Legal services at the University of Southampton and The OTTER II Trial Steering Committee. Data will be backed up, and participant identifiable data will be stored separately from study data. Trial documentation will be retained for 10 years after completion of study-related activities and managed in accordance with the University of Southampton and the University of Oxford research data management policies.

For peer review only - http://bmjopen.bmj.com/site/about/guidelines.xhtml

For peer review only

Audio-recordings from the interviews will be transcribed verbatim, and participants allocated an ID number. The text of the interviewer notes from the telephone interviews will be anonymised and linked to the interview through the ID number. Names of participants, the names of any people discussed, health care employees and hospitals will be removed from transcripts and replaced with pseudonymns. Participants will be sent a letter to thank them for taking part in the telephone interview. A 'future use of interview' question is included on the consent form which gives copyright to the Universities of Southampton and Oxford to use the material in research, teaching, publications and broadcasting.

Statistical analysis

The statistical analysis will be carried out by the OTTER II Trial OCTRU trial statistician. As is usual practice for the Oxford Cinical Trials Unit the statistician will not be blind to treatment allocation. There are rigourous checks and balances in place to ensure that the trial statistician cannot bias outcome.

This three-arm trial will first assess the global comparison of differences between the three treatment arms at the 5% level. The primary (global) Null Hypothesis is that there is no difference between the three arms. Only if this null hypothesis is rejected at the 5% (2-sided) significance level will the pairwise comparisons be carried out in order to explore where the difference lies:

· Verum splint + self-management programme (Group B) versus self-management programme alone (Group A)

· Placebo splint + self-management programme (Group C) versus self-management progamme alone (Group A)

· Verum splint + self-management programme (Group B) versus placebo splint + self-management programme (Group C)

It is anticipated that there will be an adjustment for multiple testing at this stage, and Bonferroni or a less conservative method will be used. The main analysis will be intention to treat. A per-protocol

For peer review only - http://bmjopen.bmj.com/site/about/guidelines.xhtml

analysis of the primary endpoint will be performed as part of the sensitivity analyses. A Statistical Analysis Plan will provide full details of all planned analyses.

The statistical analysis of the primary outcome, AUSCAN hand pain score, will be performed using ANCOVA, adjusting for baseline pain and stratification factors. Continuous secondary outcomes will be analysed using similar methods to the primary outcome. The unadjusted secondary binary variables will be compared using chi-squared tests with logistic regression being used to adjust for stratification and important prognostic factors in a multi-variable framework.

Baseline characteristics for the three groups will be presented using the appropriate descriptive statistics. Baseline characteristics of participants who completed the trial and those who dropped out will be presented and explored in order to ascertain patterns of loss to follow-up. Subgroups will be examined to look for consistency of any observed treatment effects (using interactions). This analysis will be considered of an exploratory nature potentially providing hypotheses for future studies. The study is not powered to examine these in detail and will not therefore report p-values for these.

Qualitative analysis

Forty interviews will be carried out by a researcher blind to participant treatment allocation. Audio-recordings of the 40 interviews will be transcribed verbatim, anonymised and imported into analysis software QRS NVivo. The researcher will conduct primary coding of the narrative data and the five stages of framework analysis as described by Ritchie and Lewis [50] will be followed: familiarisation, identifying a thematic framework, indexing, charting and mapping/ interpretation. Using a thematic approach [51] data will be inductively coded, codes grouped to create categories and a descriptive account will be produced. Up to 10 interviews will be double coded to increase the validity of the findings. Double coding means more than one researcher independently assigning pre-specified codes to the qualitative data. NVivo software (QSR International) will be used to facilitate the double coding process. This allows for coding comparisons to be run between different coders. The results are returned according to the Kappa coefficient score and the percentage of agreement between coders. After double coding transcripts, we plan to use this coding comparison for

For peer review only - http://bmjopen.bmj.com/site/about/guidelines.xhtml

discussion and reflection on the data. The numeric measure will be used as a method of comparison to gauge if there is agreement and understanding of the definitions of the codes between team members. Any discrepancies identified will then be discussed until an agreement is reached and any differences resolved. The account will provide structured descriptions of participants' experience of self-management and splints, including reasons for their views about effectiveness as well as information about acceptability and adherence.

Quality of Life Analysis

The EQ-5D-5L [52] will be administered to participants at baseline, 8 weeks and 12 weeks. Responses will be converted into utilities using tariffs estimated from a representative sample of the UK population [53]. Survival information collected from the trial will be combined with EQ-5D utilities to generate Quality Adjusted Life-Years (QALYs).

Economic Evaluation and Analysis

The perspective adopted in the economic analysis will be that of the NHS. Costs associated with the following healthcare resource categories over the 8 week intervention period and follow-up period (12 weeks) will be included.

· Intervention provision (including: splints and clinical staff time required for splinting); and

· Primary care contacts, including surgery and home visits by GPs, nurses, and out-of-hours medical services, and community therapists; and

· Hospital care services, including scheduled and unscheduled inpatient admissions, surgery, accident and emergency visits and outpatient care contacts.

Primary and hospital care resource use will be obtained from patient questionnaires administered at 8 weeks and at 12 week follow-up. Healthcare resources will be valued using unit cost schedules such as Personal Social Services Research Unit and NHS Reference costs. Costs associated with splints and other disposables will be obtained from the manufacturers and the NHS Supply Chain catalogue. Using the WPAI [42] the number of work days lost by study participants and the impact that OA had on the levels of productivity/activity and unpaid work will also be measured over both study periods.

For peer review only - http://bmjopen.bmj.com/site/about/guidelines.xhtml

An economic evaluation adherent to guidelines for good economic evaluation practice [54] will be undertaken A within-trial cost-utility analysis will explore the incremental cost per QALY gained by splinting of the thumb base and a self-management programme when compared to: 1) placebo-splinting and a self-management programme; and 2) a self-management programme alone. The analyses will be conducted at eight weeks and at 12 weeks. Cost and effect results will be reported as means with standard deviations, with mean differences between the two patient groups reported alongside 95% confidence intervals. Depending on the amount of missing cost and quality of life data, missing data will be imputed using recommended multiple imputation methods [55], with results from this analysis being presented as an additional sensitivity analysis.

Incremental cost-effectiveness will be calculated by dividing the difference in costs by the difference in effects. Uncertainty around the incremental cost-effectiveness ratio will be explored using non-parametric bootstrapping. A supplementary economic evaluation including non-NHS costs will be conducted in an additional sensitivity analysis. This will include costs of impaired productivity/activity and, any work and non-work days (e.g. leisure or non-paid work) lost due to illness will be valued using mean average wages for those in employment. For participants who are retired or those not in employment, loss in activity levels (e.g. leisure, caring or non-paid work activities) due to illness will be valued using minimum wages.

ETHICS AND DISSEMINATION

South Central - Oxford C Research Ethics Committee approved the study and the OTTER II trial will undergo regular monitoring. The quality assurance evaluation of trial processes and intervention fidelity using a trial monitoring template will be carried out on 50% of the recruiting sites by the research team. Should there be no noted issues no follow-up monitoring will be conducted. There are no criteria that will flag a need to initiate a quality evaluation, however, continual scrutiny of the CRFs received from sites by the study team, will alert the research team to possible issues (e.g. incomplete completion of CRFs, missing data or mis-randomisation) that may subsequently require

For peer review only

For peer review only - http://bmjopen.bmj.com/site/about/guidelines.xhtml

quality assurance evaluation. A standard risk assessment will be conducted and a risk-based proportionate monitoring plan will be put in place, which will include central monitoring.

Adverse event and safety oversight will comply with OCTRU SOPs. Adverse Responses/Reactions, Adverse Device Effects, Serious Adverse Responses/Reactions, Serious Adverse Device Effects, Suspected Unexpected Serious Adverse Responses/Reactions (SUSARs) and Device Deficiencies will be recorded as study outcomes. Sites will be required to report all serious events that are related to a trial intervention within 24 hours and all related serious adverse events must be assessed for causality and reason for seriousness. All Device Deficiencies, Adverse Reactions and Adverse Device Effects must be reported to the trial team within 14 days. All Serious Adverse Event safety monitoring forms will be passed on to the Trial's Nominated Clinician who will perform an independent assessment of causality and will also perform assessment of expectedness based on what is known and documented about the intervention/device. Any SUSARs will be reported to the Research Ethics Committee within 15 days of the Trials Office being made aware of the event

An independent DSMC (Appendix 9) will be convened with two expert clinicians and a statistician to regularly review accumulating data in order to assess patient safety and study conduct following the recommendations of the DAMOCLES study [56] with full details being provided in a charter. They will advise the Trial Steering Committee (Appendix 10) as to whether recruitment should stop early, which is only likely to occur if either intervention is shown to be unsafe for patients. As this is a low risk study no interim efficacy comparative analyses are planned and no safety issues are expected. The OTTER II trial team will have access to the final trial dataset and there will be no public access to patient level data or the statistical code used within the trial.

We believe this to be the first fully powered placebo controlled splinting trial exploring the effectiveness and efficacy of splints in symptomatic thumb base OA. Study results will be disseminated to rheumatology, hand therapy, occupational therapy and physiotherapy national and international conferences and submitted for consideration in international and national academic and professional conferences and journals. Lay publications written in accessible language will be provided to charitable, community and patient facing publications as recommended by our patient

For peer review only - http://bmjopen.bmj.com/site/about/guidelines.xhtml

and public involvement partners. Study participants will be provided regular updates of the study progress through the OTTER II study website.

For peer review only

For peer review only - http://bmjopen.bmj.com/site/about/guidelines.xhtml

Authors' contributions:

Jo Adams: Principal investigator, conceived research project and first author of paper

Paula Barratt: Trial manager, written and reviewed trial protocol and co-author

Nigel Arden: Trial team member contributed to original protocol and co-applicant and co-author

Sofia Barbosa Bouças: Trial team member contributed to pilot development and original protocol, co-applicant and co-author

Sarah Bradley: Trial team member contributed to original protocol and co-applicant and co-author

Michael Doherty: Trial team member contributed to original protocol and co-applicant and co-author

Susan Dutton: Trial team member contributed to original protocol, statistical lead, co-applicant and co-author

Krysia Dziedzic: Trial team member contributed to original protocol and co-applicant and co-author

Rachael Gooberman-Hill: Trial team member contributed to original protocol and co-applicant and co-author

Kelly Hislop: Trial team member contributed to pilot development and original protocol, co-applicant and co-author

Corinne Hutt Greenyer: OTTER patient partner, co-applicant, co-author, reviewed and revised paper

For peer review only - http://bmjopen.bmj.com/site/about/guidelines.xhtml

Vic Jansen: Trial team member contributed to revision and clinical review of original protocol and co-author

Ramon Luengo Fernadez: Trial team member contributed to original protocol and co-applicant and co-author, led health economic input

Claire Meagher: Trial team member, reviewed and revised qualitative component and co-author

Peter White: Trial team member contributed to original protocol and placebo development and co-applicant and co-author

Mark Williams: Trial team member contributed to original protocol reviewed and revised paper

Funding statement: 'This work was supported by Arthritis Research UK Grant Ref 21019 and the funder is listed and complies with the SHERPA JULIET guidelines

http://v2.sherpa.ac.uk/id/funder/14

Competing interests statement. The authors and wider OTTER II trial team have no competing interests

Acknowledgements.

The authors wish to acknowledge the OTTER II collaborative group:

Derby Teaching Hospitals NHS Foundation Trust: Victoria Jansen, Helen McKenna, Ellen Bramall, Carole Henderson, Chloe Kirk, Diane Langford, James Turner, Anna Selby, Linda Tozer, Navdeep Johal, Fernando Parrales, Madina Asif, Kat Hill, Stefania Wigelsworth

Doncaster and Bassetlaw Teaching Hospitals NHS Foundation Trust: Su McIlwaine, Elaine Bonser, Lynn Houghton, Ryan Roberts

Dorset County Hospital NHS Foundation Trust: Maree Dethick-Jones, Sheena Colhoun, Louise Clark, Liz Barnish, Simone Caddy, Josie Goodsell, Sarah Horton, Laura Howell, Simon Sharpe

Hampshire Hospitals NHS Foundation Trust: Kevin Spear, Christina Macleod, Libby Denman, Becky Shaylor, Kathy Whalley, Mark Pulley, Hannah Bolger

Midlands Partnership NHS Foundation Trust: Carol Graham, Nicky Edwards, Jane Rivers-Latham, Yvonne Salt, Tilly Grocott, Alison Williams, Sarah Gibson, Lisa Oakley, Susan Thompson, Susan Woodroffe, Laura Denny, Amy Thompson, Natalie Wheat

North Devon Healthcare NHS Trust: Jo Harness, Jane Hunt, Henrietta Clay, Becky Holbrook, Lucia Stancombe, Martin Howard, Nicholas McGuirk, Mark Bryce

For peer review only - http://bmjopen.bmj.com/site/about/guidelines.xhtml

For peer review only

Pennine MSK Partnership Ltd: Jill Firth, Kathy Kinsey, Helen Light, Charlotte Critchley, David Pilbury, Danielle Burke, Karen Partridge, Tracy Parry, Norah Handley, Kath Spencer
Poole Hospital NHS Foundation Trust: Sarah Bradley, Corinna Rogers, Paula Reynolds, Bridget Ellis, Sharon Page
Portsmouth Hospitals NHS Trust: Caroline Mountain, Gemma Willis, Catherine Coleman, Catherine Kirby, Paula White, Glenn Lake, Julie Williams, Marie White
Royal Devon and Exeter NHS Foundation Trust: Suzannah Blake, Abigail Owen, Emily Rogers, Claire Hughes, Cresta Browning, Jacqueline Fowler, Cristina Burke-Trees
Royal Free London NHS Foundation Trust: Juliette Bray, Nikki Burr, Meera Anadkat, Cherry Kilbride, Francesca Gowing
Royal United Hospitals Bath NHS Foundation Trust: Sandi Derham, Jessica Chipps, Suzanne Green, Helen Gordon-Johnson, Mark Sheriff, Susan Greene, Michelle Lawrence, Belinda Jones, Jack Spence, Ali Champion, Emily Anson
The Robert Jones and Agnes Hunt Orthopaedic Hospital NHS Foundation Trust: Tracy Jones, Michelle Jones, Julie Steen, Daniel Griffiths, Sally van Liefland, Jayne Edwards, Linda Griffith
Yeovil District Hospital NHS Foundation Trust: Helen Truman, Frances Campbell, Helen Jones, Jennifer Harrison, Vickie Ridley, Sue Chesterman, Joanna Allison, Tressy Pitt-Kerby, Kate Beesley

The authors also wish to acknowledge the OTTER II Trial Administrator: Carrie Fanning, University of Southampton, Southampton, UK.

Legend for Figure 1:

Figure 1. Participants' process throughout the trial

For peer review only - http://bmjopen.bmj.com/site/about/guidelines.xhtml

References

1. Abhishek A, Doherty M. Clinical Features of Osteoarthritis In: Watts R, Conaghan P, Denton C, et al., eds. Oxford Textbook of Rheumatology (4 ed). Oxford Oxford University Press, 2013.
2. Maheu E, Berenbaum F, . Time for new outcome measures in hand osteoarthritis? . F Nat Clin Pract Rheumatol 2009;**5():.**(3):136-38
3. Marshall M, Peat G, Nicholls E, et al. Subsets of symptomatic hand osteoarthritis in community-dwelling older adults in the United Kingdom: prevalence, inter-relationships, risk factor profiles and clinical characteristics at baseline and 3-years
Osteoarthritis and Cartilage
2013;**21**(11):1674-84
4. Haugen IK, Englund M, Aliabadi P, et al. Prevlance, incidence and progression of hand osteoarthritis in the general population:the Framingham osteoarthritis study. 2011 doi: 10.1136/ard.2011.150078[published Online First: Epub Date]|.
5. Bijsterbosch J, Visser W, Kroon H, et al. Thumb base involvement in symptomatic hand osteoarthritis is associated with more pain and functional disability. Ann Rheum Dis 2010;**69**(3):585-87 doi: doi: 10.1136/ard.2009.104562. Epub 2010 Feb 2[published Online First: Epub Date]|.
6. Zhang W, Doherty M, Leeb B, et al. EULAR evidence-based recommendations for the diagnosis of hand osteoarthritis: report of a task force of ESCISIT. Ann Rheum Dis 2009;**68**:8-17 doi: doi:10.1136/ard.2007.084772
[published Online First: Epub Date]|.
7. Spacek E, Poiraudeau S, Fayad F, et al. Disability induced by hand osteoarthritis: are patients with more symptoms at digits 2-5 interphalangeal joints different from those with more symptoms at the base of the thumb?
. Osteoarthritis and Cartilage 2004;**12**:366-73
8. Kwok WY, Vliet Vlieland TPM, Rosendaal FR, et al. Limitations in daily activities are the major determinant of reduced health-related quality of life in patients with hand osteoarthritis. Annals of the Rheumatic Diseases 2011;**70**(2):334-36 doi: 10.1136/ard.2010.133603[published Online First: Epub Date]|.
9. Van Den Ende C, Stukstette M, Dekker J, et al. Thumb base involvement in established hand osteoarthritis Annals of the Rheumatic Diseases 2013;**72**(S3):784 doi: 10.1136/annrheumdis-2013[published Online First: Epub Date]|.
10. Poole JU, Pellegrini VD, Jr. Arthritis of the thumb basal joint complex. Journal of hand therapy : official journal of the American Society of Hand Therapists 2000;**13**(2):91-107
11. Zhang Y, Niu J, Kelly-Hayes M, et al. Prevalence of symptomatic hand osteoarthritis and its impact on functional status among the elderly: The Framingham Study. Am J Epidemiol 2002;**156**(11):1021-7
12. Hill S, Dziedzic K, Ong B. The functional and psychological impact of hand osteoarthritis. Chronic Illness 2010;**6**:101-10
13. Bani MA, Arazpour M, Curran S. Design and construction of custom-made neoprene thumb carpo-metacarpal orthosis with thermoplastic stabilization for first carpo-metacarpal joint osteoarthritis. Journal of Hand Therapy 2013;**26**(3):279-81 doi: https://doi.org/10.1016/j.jht.2013.01.005[published Online First: Epub Date]|.
14. Grenier M-L, Mendonca R, Dalley P. The effectiveness of orthoses in the conservative management of thumb CMC joint osteoarthritis: An analysis of functional pinch

For peer review only - http://bmjopen.bmj.com/site/about/guidelines.xhtml

strength. Journal of Hand Therapy 2016;**29**(3):307-13 doi: https://doi.org/10.1016/j.jht.2016.02.004[published Online First: Epub Date]|.
15. Colditz JC. The biomechanics of a thumb carpometacarpal immobilization splint: Design and fitting. Journal of Hand Therapy 2000;**13**(3):228-35 doi: 10.1016/S0894-1130(00)80006-X[published Online First: Epub Date]|.
16. Hamann N, Heidemann J, Heinrich K, et al. Stabilization effectiveness and functionality of different thumb orthoses in female patients with first carpometacarpal joint osteoarthritis. Clinical Biomechanics 2014;**29**(10):1170-76 doi: https://doi.org/10.1016/j.clinbiomech.2014.09.007[published Online First: Epub Date]|.
17. Kloppenburg M, Kroon FP, Blanco FJ, et al. 2018 update of the EULAR recommendations for the management of hand osteoarthritis. Annals of the Rheumatic Diseases 2018 doi: 10.1136/annrheumdis-2018-213826[published Online First: Epub Date]|.
18. Arazpour M, Soflaei M, Ahmadi Bani M, et al. The effect of thumb splinting on thenar muscles atrophy, pain, and function in subjects with thumb carpometacarpal joint osteoarthritis. Prosthetics and Orthotics International 2016;**41**(4):379-86 doi: 10.1177/0309364616664149[published Online First: Epub Date]|.
19. Hermann M, Nilsen T, Eriksen CS, et al. Effects of a soft prefabricated thumb orthosis in carpometacarpal osteoarthritis. Scandinavian Journal of Occupational Therapy 2014;**21**(1):31-39 doi: 10.3109/11038128.2013.851735[published Online First: Epub Date]|.
20. Zhang W, Doherty M, Leeb BF, et al. EULAR evidence based recommendations for the management of hand osteoarthritis: report of a Task Force of the EULAR Standing Committee for International Clinical Studies Including Therapeutics (ESCISIT). Ann Rheum Dis 2007;**66**(3):377-88
21. Kjeken I, Darre S, Smedslund G, et al. Effect of assistive technology in hand osteoarthritis: a randomised controlled trial. Annals of the Rheumatic Diseases 2011;**70**(8):1447-52 doi: 10.1136/ard.2010.148668[published Online First: Epub Date]|.
22. Bertozzi L, Valdes K, Vanti C, et al. Investigation of the effect of conservative interventions in thumb carpometacarpal osteoarthritis: systematic review and meta-analysis. Disability and rehabilitation 2015;**37**(22):2025-43 doi: 10.3109/09638288.2014.996299[published Online First: Epub Date]|.
23. Sanchez K, Jourdan C, Rannou F, et al. Assessing Orthosis:Practical Examples. In: Boutron I, Ravaud P, Moher D, eds. Randomized Clinical trials of Nonpharmacological Treatments: Chapman and Hall/CRC Biostatistics Series 2012:295-307.
24. White P, Bishop F, Prescott P, et al. Practice, practitioner, or placebo? A multifactorial, mixed-methods randomized controlled trial of acupuncture
Pain 2012;**153**(2):455-62
25. Finniss D, Kaptchuk T, Miller F, et al. Biological,clinical, and ethical advances of palcebo effects The Lancet 2010;**375**:868-695
26. Chan A-W, Tetzlaff JM, Gøtzsche PC, et al. SPIRIT 2013 Explanation and Elaboration: Guidance for protocols of clinical trials. Bmj 2013;**346**
e7586 doi: https://doi.org/10.1136/bmj.e7586[published Online First: Epub Date]|.
27. Gooberman Hill R, Jinks C, Barbosa Boucas S, et al. Designing a placebo device: involving service users in clinical trial design. Health Expectations 2013;**16**(4):e100–e10 doi: doi:10.1111/hex.12043[published Online First: Epub Date]|.

For peer review only - http://bmjopen.bmj.com/site/about/guidelines.xhtml

28. Barbosa Boucas S, Hislop K, Dziedic K, et al. Defining optimal NHS occupational therapy treatment, individualized splint and placebo splint for patients with thumb base OA: a Delphi study. Rheumatology 2013a;**52**(S1):i105
29. Barbosa Boucas S, Hislop Lennie K, Dziedzic K, et al. Differences between service providers and users when defining feasible optimal NHS occupational therapy treatment for patients with thumb base OA: Results from a Delphi study. Annals of the Rheumatic Diseases 2013b;**72**(S3):779-80
30. Jones L, White P, Donovan-Hall M, et al. The Thoughts and Feelings Held by Clinicians about the delivery of a Placebo Thumb Splint in an Osteoarthritis Rehabilitation Trial. Hand Therapy 2013;**18**(3):77-83
31. Adams J, Barbosa Bouças S, Hislop K, et al. The Effectiveness and Efficacy of Splints for Thumb Base Osteoarthritis: A Pilot Randomized Controlled Trial. Rheumatology 2014;**53**(suppl_1):i41-i42 doi: 10.1093/rheumatology/keu090.005[published Online First: Epub Date]|.
32. Adams J, Barratt P, Bradley S, et al. Participating in a musculoskeletal randomised controlled trial: identification of education training needs by occupational therapists and physiotherapists in the uk. Annals of the Rheumatic Diseases 2017;**76**(Suppl 2):1508-08 doi: 10.1136/annrheumdis-2017-eular.1095[published Online First: Epub Date]|.
33. Gossec L, Dougados M. Intra-articular treatments in osteoarthritis: from the symptomatic to the structure modifying. Annals of the Rheumatic Diseases 2004;**63**(5):478 doi: 10.1136/ard.2003.013771[published Online First: Epub Date]|.
34. Trellu S, Dadoun S, Berenbaum F, et al. Intra-articular injections in thumb osteoarthritis: A systematic review and meta-analysis of randomized controlled trials. Joint Bone Spine 2015;**82**(5):315-19 doi: https://doi.org/10.1016/j.jbspin.2015.02.002[published Online First: Epub Date]|.
35. Heyworth BE, Lee JH, Kim PD, et al. Hylan versus corticosteroid versus placebo for treatment of basal joint arthritis: a prospective, randomized, double-blinded clinical trial. J Hand Surg Am 2008;**33**(1):40-8 doi: 10.1016/j.jhsa.2007.10.009[published Online First: Epub Date]|.
36. Bellamy N, Campbell J, Haraoui B, et al. Clinimetric properties of the AUSCAN Osteoarthritis Hand Index: an evaluation of reliability, validity and responsiveness. Osteoarthritis Cartilage 2002;**10**(11):863-9
37. Gelberman RH, Boone S, Osei DA, et al. Trapeziometacarpal Arthritis: A Prospective Clinical Evaluation of the Thumb Adduction and Extension Provocative Tests. J Hand Surg Am 2015;**40**(7):1285-91 doi: 10.1016/j.jhsa.2015.04.012[published Online First: Epub Date]|.
38. Kjeken I, Smedslund G, Moe RH, et al. Systematic Review of Design and Effects of Splints and Exercise Programs in Hand Osteoarthritis. Arthritis Care & Research 2011;**63**(6):834-48 doi: 10.1002/acr.20427[published Online First: Epub Date]|.
39. Loyley M, Davis L. An investigation of the pressure, temperature and functional impact of thumb base splints for people with thumb based pain [MSc research dissertation paper]. University of Southampton 2017.
40. NIHR. Good Clinical Practice Secondary Good Clinical Practice 2019. https://www.nihr.ac.uk/our-research-community/clinical-research-staff/learning-and-development/national-directory/good-clinical-practice/.

For peer review only - http://bmjopen.bmj.com/site/about/guidelines.xhtml

41. Dellhag B, Bjelle A. A grip ability test for use in rheumatology practice. The Journal of Rheumatology 1995;**22**(8):1559-65
42. Reilly MC, Zbrozek AS, Dukes EM. The validity and reproducibility of a work productivity and activity impairment instrument. PharmacoEconomics 1993;**4**(5):353-65
43. Ware JE, Kosinski M, Keller SD. A 12-item short-form health survey, construction of scales and preliminary tests of reliability and validity. . Medical care 1996;**34**(3):220-33
44. Euroqol Group. Euroqol: a new facility for the measurement of health-related quality of life. Health Policy 1990;**16**:199-208
45. Chung KC, Pillsbury MS, Walters MR, et al. Reliability and validity testing of the Michigan Hand Outcomes Questionnaire The Journal of Hand Surgery 1998;**23A**(4):575-87
46. Hudak PL, Amadio PC, Bombardier C. Development of an upper extremity outcome measure: The DASH (Disabilities of the Arm, Shoulder and Hand). American Journal of Industrial Medicine 1996;**29**:602-08
47. Lorig K, Chastain R, Ung E, et al. Development and evaluation of a scale to measure self-efficacy in people with arthritis. Arthritis Rheum 1989;**32**(1):37-44
48. Pham T, van der Heijde D, Altman RD, et al. OMERACT-OARSI initiative: Osteoarthritis Research Society International set of responder criteria for osteoarthritis clinical trials revisited. Osteoarthritis Cartilage 2004;**12**(5):389-99
49. J. C. *Statistical Power Analysis for the Behavioral Sciences*. New York Routledge Academic

1988.
50. Ritchie J, Lewis J. *Qualitative research practice: a guide for social science students and researchers*: Sage, 2003.
51. Charmaz K. *Constructing grounded theory: a practical guide through qualitative analysis* London Sage 2006.
52. EuroQol--a new facility for the measurement of health-related quality of life. Health Policy 1990;**16**(3):199-208
53. van Hout B, Janssen MF, Feng YS, et al. Interim scoring for the EQ-5D-5L to EQ-5D-3L value sets. Value Health 2012;**15**:708-15
54. Drummond MF, Sculpher MJ, Claxton K, et al. *Methods for the Economic Evaluation of Health Care Programmes*. 4th Edition ed: Oxford University Press, 2015.
55. Royston P. Multiple imputation of missing values. Stata Journal 2004;**4**:227-41
56. DAMOCLES Study Group NHS Health Technology Assessment Programme. A proposed charter for clinical trial data monitoring committees: helping them to do their job well. Lancet 2005;**365**:711-22

For peer review only - http://bmjopen.bmj.com/site/about/guidelines.xhtml

OTTER II Trial: flow of participants

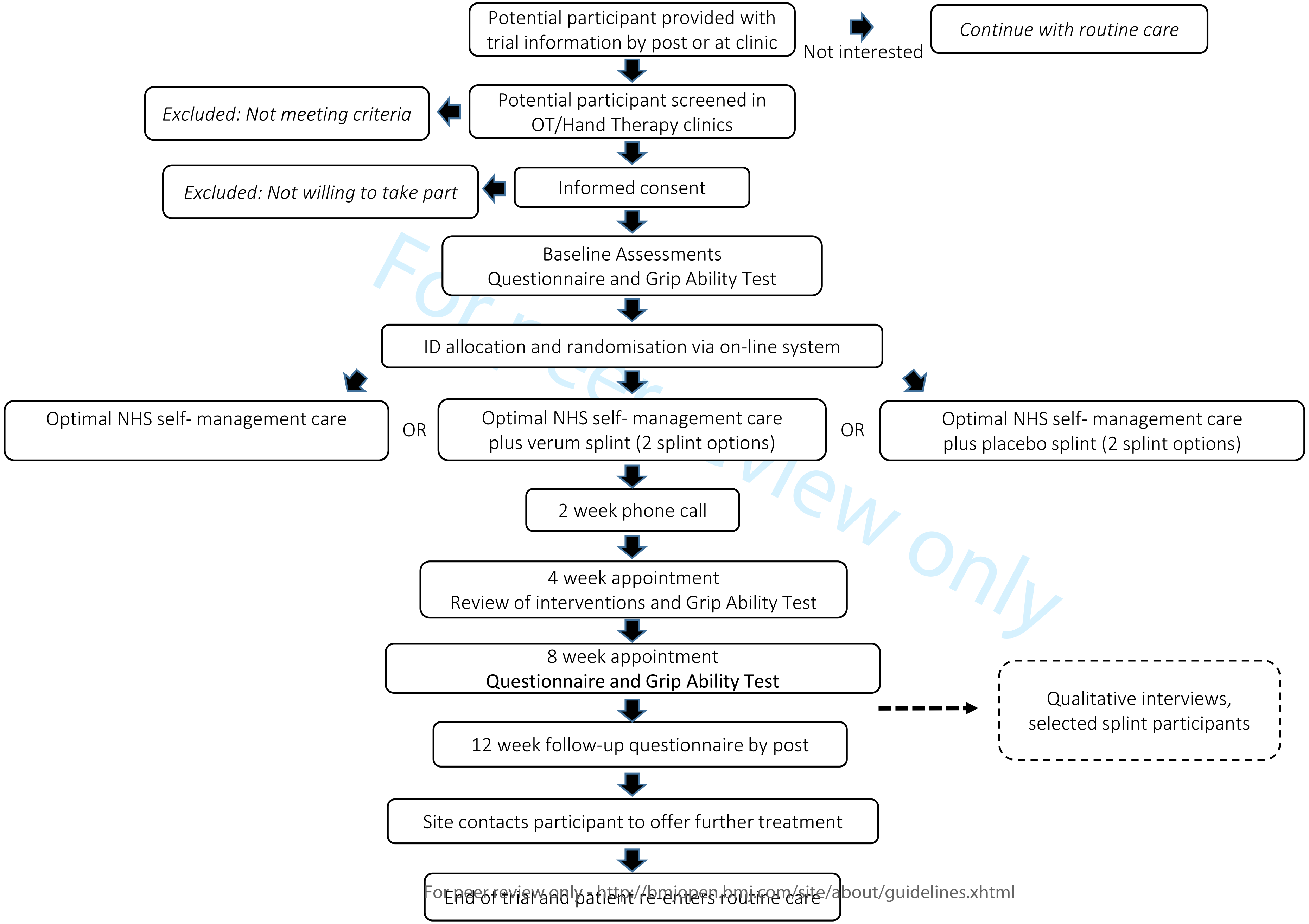

For peer review only

Appendix 1

NHS Recruitment Sites

Pulvertaft Hand Centre, Royal Derby Hospital, Derby Teaching Hospitals NHS Foundation Trust, Derby, UK

Hand Therapy Department, Dorset County Hospital, Dorset County Hospital NHS Foundation Trust, Dorchester, UK

Hand Therapy Unit, Poole Hospital, Poole Hospital NHS Foundation Trust, Poole, UK

Plastic Surgery Rehabilitation, Royal Devon and Exeter Hospital, Royal Devon and Exeter NHS Foundation Trust, Exeter, UK

Occupational Therapy Department, North Devon District Hospital, North Devon Healthcare NHS Trust, Barnstaple, UK

Rheumatology Therapy Department, Royal National Hospital for Rheumatic Diseases, Royal United Hospitals Bath NHS Foundation Trust, Bath, UK

Therapies Department, Royal United Hospitals, Royal United Hospitals Bath NHS Foundation Trust, Bath, UK

Occupational Therapy Department, Haywood Hospital, Midlands Partnership NHS Foundation Trust, Stoke-on-Trent, UK

Pennine MSK Partnership Ltd., Integrated Care Centre, Oldham, UK

Clinical Therapies Department, Bassetlaw Hospital, Doncaster and Bassetlaw Teaching Hospitals NHS Foundation Trust, Worksop, UK

Bassetlaw Locality Retford Primary Care Centre, Doncaster and Bassetlaw Teaching Hospitals NHS Foundation Trust, Retford, UK

Therapy Department, Yeovil Hospital, Yeovil District Hospital NHS Foundation Trust, Yeovil, UK

Hand Therapy, Basingstoke and North Hampshire Hospital, Hampshire Hospitals NHS Foundation Trust, Basingstoke, UK

Therapy Department, Royal Hampshire County Hospital, Hampshire Hospitals NHS Foundation Trust, Winchester, UK

Occupational Therapy Department, Queen Alexandra Hospital, Portsmouth Hospitals NHS Trust, Cosham, UK

Hand Therapy Unit, The Robert Jones and Agnes Hunt Orthopaedic Hospital, The Robert Jones and Agnes Hunt Orthopaedic Hospital NHS Foundation Trust, Oswestry, UK

For peer review only

Hand Therapy, The Royal Free Hospital, Royal Free London NHS Foundation Trust, London, UK

For peer review only

Appendix 2

Self-Management Package:

Hand Exercise Booklet *(see separate supplementary pdf file as too large a file to include)*

For peer review only

Appendix 2

Self-Management Package: Joint Protection Booklet

Osteoarthritis Thumb Base Therapy Trial

OTTER trial

Osteoarthritis Joint Protection Booklet

Information and advice about reducing pain and protecting your joints

OTTER II trial – REC ref: 16/SC/0188

For peer review only

Osteoarthritis

You have been given this booklet because you have pain or discomfort in one or more of your joints.

Osteoarthritis is a common cause of joint pain effecting 20% of the general population aged over 55 years.

Osteoarthritis can affect many aspects of your everyday life. This booklet has been designed with the help of clinicians in response to questions asked by patients with Osteoarthritis including advice about how to carry on with your normal life as much as possible whilst reducing pain and protecting your joints from further damage.

This booklet contains information on the following:

1. What is Osteoarthritis?

2. Protecting your joints

3. Dealing with fatigue

4. Diet

5. Holistic Medicine

For peer review only

1. What is Osteoarthritis?

A ‘normal’ joint is where two or more bones meet. The joint allows the bones to move freely but within limits.

Osteoarthritis is a disease that affects the body’s joints. The surfaces within the joint are damaged so the joint does not move as smoothly as it should. The main symptoms are pain and sometimes stiffness.

When a joint develops Osteoarthritis, some of the cartilage covering the ends of the bones gradually roughens and becomes thin and the bone underneath thickens. All the tissues within the joint become more active than normal, as if the body is trying to repair the damage.

Sometimes the body’s repairs are quite successful and the changes inside the joint will not cause much pain. If the Osteoarthritis becomes severe, the cartilage can become so thin that it no longer covers the ends of the bones. The bones start to rub against each other and eventually can start to wear away. The loss of cartilage, the wearing of bone and sometimes bony spurs can alter the shape of the joint, forcing the bones out of their normal alignment.

2. Protecting your joints

Joint protection can reduce joint damage, preserve range of motion, and lessen Osteoarthritis pain by reducing the general stress and strain on the joint. Making changes early can help avoid joint problems becoming worse in the future.

Become more aware of how you use the joints that ache, both at home and at work. For example, try watching your actions while you use your hands – for example when you make a hot drink:

- What is happening to your fingers while you are turning the tap? Are they being pushed towards the little finger?
- What happens to your thumbs as you take the lid of the coffee jar? Is there pressure or aching at the base of your thumb?
- What is happening to your wrist and fingers as you lift the kettle? Can you feel aching or pulling at these joints?

For peer review only

You might already have tried picking up the kettle with two hands when your hands are painful, but it is important to do this **all the time**, not just when your hands are hurting. This is an example of joint protection.

For peer review only

The following principles may help you:

- **Respect pain.** If you are experiencing pain after an activity, you must consider that you have been too active or have done too much.
- **Pace your activities throughout the day.** Spread physically hard jobs, such as housework or mowing the lawn, at intervals through the day, rather than tackling them all at once.
- **Avoid any activity that causes pain and find a better way of accomplishing the task.** Avoid a tight grip that strains joints and muscles. Avoid a prolonged or continuous grip. Small joints can take little weight, so gripping a pen or cutlery can be hard work. **Holding a larger object involves more joints, so it spreads the grip.** Picking up an object with two hands halves the load on each hand.

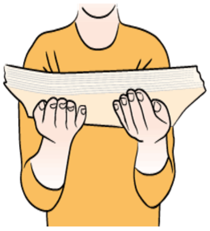

- **Make handles larger with padding,** for example, sponge tubing for pipe lagging. A solid surface is harder to grip than a soft surface, also a larger surface area means less strain. Wear padded gloves when gardening.
- **Use adaptive devices**. There are many devices available on the market to make it easier for you to carry out everyday tasks. Below are examples of some which may make your life easier and reduce the stress on your thumb joint:
    - **Spring loaded scissors** reduce the load on your thumb.

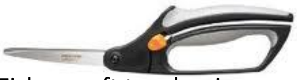

Fiskars soft touch scissors

    - **Jar Keys** can really make a difference as they break the vacuum when opening jars.

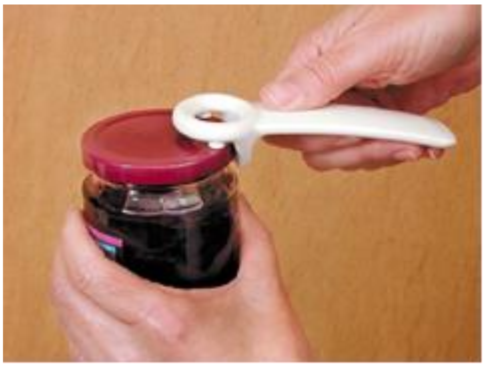

For peer review only

- **L-shaped knifes** do not use the thumb at all.

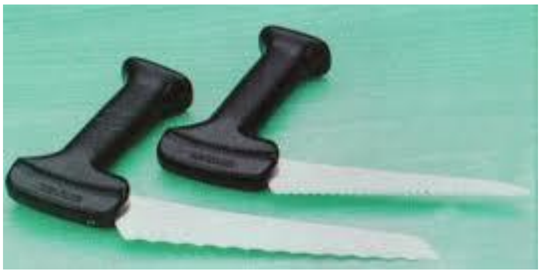

- **Ring pull can openers** like a 'magi pull' available from Lakeland can make opening cans much easier.
- As we age the natural stickiness of our hands decrease and are hands become dryer, this can lead us to have to use 20% more grip strength to open jars, etc. Using a **damp dish cloth** can reduce the amount of grip strength you need to use.
- When straining vegetables, instead of lifting and tipping the pan, **place vegetables in a wire basket in the pan to cook**. When ready they can be lifted and drained in the basket. Leave the saucepan to cool before moving it.
- When using the kettle, **use a plastic jug to fill the kettle from the tap** and only fill the kettle up as required. A **kettle tipper can be used**, or a small lightweight travel kettle. Also, kettles with the handle over the top have been reported to be easier to lift using two hands rather than the kettles with the handle on the side.

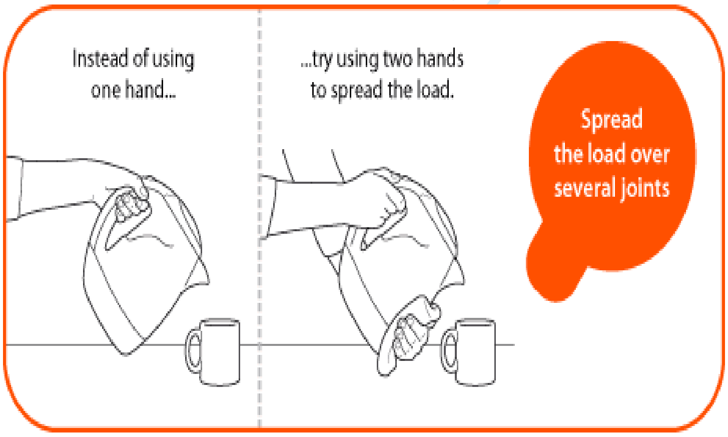

3. Dealing with fatigue

At times people with arthritis may experience fatigue. Fatigue is a feeling of weariness, but it is more extreme than simple tiredness. It can affect you physically, making your limbs seem heavy and causing you to feel exhausted, but it can also affect your concentration and

For peer review only - http://bmjopen.bmj.com/site/about/guidelines.xhtml

motivation. People who experience fatigue may find they struggle to do even small tasks. It often comes on for no apparent reason and without warning. There are many things that can cause fatigue including those listed below:

- Anaemia, which often accompanies inflammation.
- Other long term conditions such as diabetes or thyroid disease.
- Some drugs used to treat arthritis which may cause drowsiness or loss of concentration.
- Pain, especially if it is long term weak muscles, which mean you have to use more energy to do everyday tasks.
- Overdoing things or carrying on with activities for too long.
- Sleep disturbances as a result of pain, late nights or sleeping too much in the day.
- Stress and anxiety.
- Low mood or depression.
- Poor diet or hunger.

What can you do to help yourself?

- **Talk to your GP** or Rheumatology team about getting support or a **review of your medication**.
- Use the **four 'Ps'** – **P**roblem Solving, **P**lanning, **P**rioritising and **P**acing.
- **Tell family, friends and colleagues** about your fatigue so that they can understand and help if needed.
- Gradually **increase your physical exercise**. This will improve your general well-being, strength and energy levels.
- **Deal with stress or anxiety**.
- **Talk to your GP** if you have low mood.
- Improve your **sleeping habits**.
- Eat a **healthy diet**.

For peer review only

4. Diet

Whilst it is not the cause of Osteoarthritis and thumb base pain, being overweight can affect your health in many ways. If you are overweight then losing weight will help reduce the strain on your joints, increase your sense of well-being and reduce your risk of other health problems.

Apart from **reducing sugar** and **fat**, **taking regular exercise,** and ensuring that you **have your five a day of fruit and vegetables**, it has been shown that **increasing** your **intake** of particular vitamins and minerals, particularly **Calcium, Vitamin D** and **Iron**, **eating less saturated fat** and incorporating more **good fats** in your diet such as **Omega 3** can improve symptoms of Arthritis. See table below for a list of good and bad fats.

Saturated Fats (Bad Fats) **Avoid**	Polyunsaturated Fats (Fats that can increase inflammation) **Avoid**	Monounsaturated Fats (Neutral fats but contain a high amount of calories) **Limit your intake**
• Full fat dairy products<br>• Processed foods (for example, cakes, biscuits and pastry)<br>• Chips, if fried in animal fat<br>• Foods cooked using ghee (clarified butter)	• Softer Fats and oils<br>• Corn or sunflower sources of oil	• Olive oil<br>• Rapeseed oil

Omega-3 Polyunsaturated Fatty Acids

These are important to keep in your diet. **Omega-3** has been found to be of benefit to people with Arthritis. It can be found in free range eggs, oily fish and fish oil supplements. The table below shows a list of some oily fish which you should aim to eat at least **twice a week**, but **no more than four times a week**.

Oily Fish **Eat at least two portions a week, but no more than four**		
• Anchovies	• Sprats	• Kippers
• Herring	• Trout	• Pilchards
• Mackerel	• Whitebait	• Sardines
• Salmon	• Eel	• Swordfish

For peer review only

• Tuna (fresh not tinned)

Supplements

There are a range of supplements you can purchase that have been shown to have some effect on pain and inflammation caused by arthritis, these include **Fish Oils** (not to be confused with fish liver oil), **Glucosamine** (check with your GP before taking if you have diabetes), **Vitamin D, Calcium** and **Iron**.

Supplements are a good way to boost your intake of these beneficial substances but they should not be used in place of a healthy diet.

For peer review only

5.Holistic Medicine

Many people who have Arthritis find benefit in using some forms of complimentary or alternative medicine alongside their usual medication.

It is important that you **always discuss any complementary or alternative medicine with your GP** or the Rheumatology team before embarking on a treatment.

There are many alternatives to main stream medicine that may be of benefit to people with arthritis, these include:

- Acupuncture
- The Alexander technique
- Aromatherapy
- Copper bracelets
- Herbal medicine
- Massage
- Relaxation and hypnosis

Holistic medicine incorporating techniques such as these are generally safe but ensure that your therapist is legally registered before starting any treatment.

There is a lot more information on these general techniques and treatments on the Arthritis Research UK website (http://www.arthritisresearchuk.org/).

This booklet includes information written and published by Arthritis Research UK.

Additional information can be obtained from the Arthritis Research UK website (http://www.arthritisresearchuk.org/).

Information was also reproduced with the kind permission of Sarah Bradley and Kirsty Bancroft of Poole NHS Trust, and Christina Macleod of Hampshire Hospitals NHS Trust.

Notes:

For peer review only

Appendix 2

Self-Management Package: Arthritis Research UK Osteoarthritis Information Booklet *(see separate pdf as large file)*

For peer review only

Appendix 2

Self-Management Package: Facilitators and Barriers to Engaging with Self-Management Principles

---

1. Participant's ID number: _ _ - _ _ - _ _ _ _

---

2. My general exercise goal is: ....

....

....

....

---

3. My specific exercise goal – <u>What</u> am I going to do? ....

....

....

....

---

4. My Confidence

How confident am I that **I <u>will</u> achieve** my specific exercise goal? Please <u>circle</u> the appropriate number below.

Not at all confident 1 2 3 4 5 6 7 8 9 10 **Extremely confident**

---

5. My Commitment

How committed am I to **achieving** my specific exercise goal? Please <u>circle</u> the appropriate number below.

Not at all committed 1 2 3 4 5 6 7 8 9 10 **Extremely committed**

---

6. My Exercise Action Plan

It is important to measure and record your progress, so that you can see when you are succeeding as well as to work out what you can change if your plan is not working.

Where am I going to do the exercises? ....

When am I going to do the exercises? ....

**Patient**	**Practitioner**
I will do the exercises – my Specific Exercise Goal – and record my progress in my Exercise Diary and bring the Exercise Diary to my next consultation.	I will discuss with you your exercises (Specific Exercise Goal) and your Exercise Diary and clarify any questions you have.
**Signature:**	**Signature:** ....
**Date:**	**Date:** ....

For peer review only

Barriers and Facilitators to doing Exercise

Unhelpful Things/Barriers	Helpful Things/Facilitators
**Places and things:**<br>Is there anything about the things around me or the places I am in that makes it difficult to do the exercises – my Specific Exercise Goal? What **can I do** to change this?	**Places and things:**<br>Is there anything about the things around me or the places I am in that makes it easier to do the exercises – my Specific Exercise Goal? What **can I do** to use these helpful things?
**People:**<br>Are there any people I spend time with who make it difficult to do the exercises – my Specific Exercise Goal? What **can I do** to change this?	**People:**<br>Are there any people I spend time with who make it easier to do the exercises – my Specific Exercise Goal? What **can I do** to ask them to help me?
**Thoughts and feelings:**<br>Is there anything that I am thinking and/or feeling that makes it difficult to do the exercises – my Specific Exercise Goal? What **can I do** to change this?	**Thoughts and feelings:**<br>Is there anything that I am thinking and/or feeling that makes it easier to do the exercises – my Specific Exercise Goal? What **can I do** to encourage these thoughts and feelings?

For peer review only

Appendix 2

Self-Management Package: Exercise Diary

Participant's ID number: _ _ - _ _ - _ _ _ _ **Date of Occupational therapy/Physiotherapy Appointment when given the exercises: Date:** _ _/_ _/_ _ _ _ (day/month/year)

Please do your hand exercises at least 3 times a week for at least 20 minutes each time. Please record how many times you did your exercise programme each day. If you didn't do your exercises please write 0

**Week**	**You can record notes or dates in this column, however you like, to help you keep track of which week you are in.**	**How many times did you do your exercises today?** Write **0** if not at all.						
		**Day 1**	**Day 2**	**Day 3**	**Day 4**	**Day 5**	**Day 6**	**Day 7**
**Week 1**								
**Week 2**								
**Week 3**								
**Week 4**								
**Week 5**								
**Week 6**								
**Week 7**								
**Week 8**								
**Week 9**								
**Week 10**								
**Week 11**								
**Week 12**								

iew only

For peer review only

Appendix 3

Verum Splint Decision Protocol

PURPOSE:

This OTTER splint decision guideline is to be used for supporting your decision to issue the verum splint for those patients allocated to the second arm in the OTTER trial.

INSTRUCTIONS:

1. **The option for the OTTER trial is to provide either**

- PROCOOL THUMB CMC RESTRICTION SPLINT
  Code: PTRS/(with the remainder of the code relating to the size/ laterality).

OR

- Orfilight 2.5mm 3/32" micro perforated (beige) trouser leg splint (made from a pre-cut template which will be provided)

Trial participants will wear this option for 12 weeks.

2. Splint decision clinical guidelines

We recommend that the choice of the verum splint is made in collaboration and agreement with the trial participant. It is important to ensure that the trial participants know and see that they do have a choice of splint to wear for 12 weeks.

We recommend that the Pro-cool thumb splint is issued when:

a) there is a generalised ache that interferes with function
b) there is mild CMCJ instability
c) a more rigid splint is impractical

We recommend that a tailor made thermoplastic “trousers splint” is made when

a) mechanical joint pain is a consistent feature, suggesting instability
b) There are high demands placed on the thumb, either in work or leisure

3 Splint prescription guidelines

It is recommended that the participant is informed that the splint is worn during ADls that aggravate their pain. As a guideline patients should be aiming to wear their splints for a minimum of 6 hours a day, during waking hours only (not to be worn over- night). This time

For peer review only

can be made up of individual splint wear periods and interspersed with active hand use. It is not expected that patients wear their splint for 6 hours at a time or in one go.

Appendix 4

Splint Wear Guide for Participants (Verum and Placebo Groups)

OTTER II Patient Splint Wear Guidelines

As part of the OTTER Trial you have been given a thumb splint by your therapist. It is important that you wear this splint over the next 12 weeks.

We ask that you wear your splint:

- ❖ During all your daily tasks that cause you thumb pain
- ❖ For a minimum of 6 hours a day
- ❖ During the day only and do not wear overnight or when asleep during the night.

Thank you for following these guidelines.
Please do not hesitate to contact your therapist if you have any questions about how or when to wear your trial splint.

For peer review only

Appendix 5

Facilitators and Barriers to Engaging with Splint Wear

---

1. Participant's ID number: _ _ - _ _ - _ _ _ _

---

2. My general splint wearing goal is:

....

....

....

---

3. My specific splint wearing goal – <u>What</u> am I going to do?

....

....

....

....

---

4. My Confidence

How confident am I that **I will achieve** my specific splint wearing goal? Please circle the appropriate number below.

Not at all confident 1 2 3 4 5 6 7 8 9 10 **Extremely confident**

---

5. My Commitment

How committed am I to **achieving** my specific splint wearing goal? Please circle the appropriate number below.

Not at all committed 1 2 3 4 5 6 7 8 9 10 **Extremely committed**

---

6. My Splint Wearing Action Plan

For peer review only

It is important to measure and record your progress, so that you can see when you are succeeding as well as to work out what you can change if your plan is not working.

Where am I going to wear the splint? ....

When am I going to wear the splint? ....

Patient

I will wear my splint – my Specific Splint Wearing Goal – and record my progress using my Splint Wearing Diary, and bring the Splint Wearing Diary to my next consultation.

Signature: ....

Date: ....

Practitioner

I will discuss with you your splint wearing regime (Specific Splint Wearing Goal) and your Splint Wearing Diary and clarify any questions you have.

Signature: ....

Date: ....

For peer review only

For peer review only

Barriers and Facilitators to Wearing the Splint

Unhelpful Things/Barriers

Places and things:

Is there anything about the things around me or the places I am in that makes it difficult to wear the splint – my Specific Splint Wearing Goal? What **can I do** to change this?

People:

Are there any people I spend time with who make it difficult to wear the splint – my Specific Splint Wearing Goal? What **can I do** to change this?

Thoughts and feelings:

Is there anything that I am thinking and/or feeling that makes it difficult to wear the splint – my Specific Splint Wearing Goal? What **can I do** to change this?

Helpful Things/Facilitators

Places and things:

Is there anything about the things around me or the places I am in that makes it easier to wear the splint – my Specific Splint Wearing Goal? What **can I do** to use these helpful things?

People:

Are there any people I spend time with who make it easier to wear the splint – my Specific Splint Wearing Goal? What **can I do** to ask them to help me?

Thoughts and feelings:

Is there anything that I am thinking and/or feeling that makes it easier to wear the splint – my Specific Splint Wearing Goal? What **can I do** to encourage these thoughts and feelings?

For peer review only

Appendix 6

Splint Wear Diary

Participant's ID number: _ _ - _ _ - _ _ _ _

Date of Occupational therapy/Physiotherapy Appointment when given the splint. Date: _ _/_ _/_ _ _ _ (day/month/year)

Please wear your splint as explained in the **OTTER Patient Splint Wear Guidelines**

Please record how many hours you wore your splint each day. If you didn't wear your splint please write **0**

Week	You can record notes or dates in this column, however you like, to help you keep track of which week you are in.	How many hours did you wear your splint today? Write 0 if not at all.						
		Day 1	Day 2	Day 3	Day 4	Day 5	Day 6	Day 7
Week 1								
Week 2								
Week 3								
Week 4								
Week 5								
Week 6								
Week 7								
Week 8								
Week 9								
Week 10								
Week 11								
Week 12								

Appendix 7

Placebo Splint Decision Protocol

PURPOSE:

This splint decision protocol is to be used for supporting your decision to issue one of the two DMO splints for those patients allocated to the third arm in the OTTER trial.

INSTRUCTIONS:

1. **The option for the OTTER trial is to provide either**

1 A Thumb Sleeve Lite DMO splint with cut out thumb sections and a wrist strap - beige

OR

2 A Thumb Sleeve DMO splint with a solid thumb component and a wrist strap - black

Trial participants will be asked to wear this option for 12 weeks.

2. **Splint decision clinical guidelines**

We recommend that the choice of splint is made in collaboration with the trial participant. Please refer to your training on delivering this dynamic splint option to maintain outcome expectancy.

It is important that participants know that they do have a choice of splint to wear for 12 weeks and we encourage that the two DMO options are shown to them to consider.

We recommend that patients agree one type of splint design to use in collaboration with their therapist. We suggest that you agree with the participant to provide the splint design that they feel fits more comfortably, the one that appears the most appealing and the one which they would prefer to wear.

3. Splint prescription guidelines

It is recommended that the participant is informed that the splint is worn during ADls that aggravate their pain. As a guideline patients should be aiming to wear their splints for a minimum of 6 hours a day, during waking hours only (not to be worn over- night). This time can be made up of individual

For peer review only

splint wear periods and interspersed with active hand use. It is not expected that patients wear their splint for 6 hours at a time or in one go.

4. Fit

Both DMO splint designs should be fitted such that the wrist strap is proximal to the ulnar styloid and does not provide any support or pressure around the wrist joint.

For peer review only

Appendix 8

Data and Safety Monitoring Committee Members

Anisur Rahman (Chair), Department of Rheumatology, University College London, London, UK

Ross Wilkie, Arthritis Research UK Primary Care Centre, Keele University, UK

Ranjit Lall, Warwick Clinical Trials Unit, University of Warwick, Coventry, UK

For peer review only

Appendix 9

Trial Steering Committee Members

Sarah Dean (Chair), University of Exeter Medical School, University of Exeter, Exeter, UK

Cathy Ball, Kennedy Institute of Rheumatology, University of Oxford, Oxford, UK

Lindsay Bearne, Faculty of Life Sciences & Medicine, King's College London, London, UK

Elaine Dennison, MRC Lifecourse Epidemiology Unit, University of Southampton, Southampton, UK

Corinne Hutt Greenyer, School of Health Sciences, University of Southampton, Southampton, UK

Jo Adams, School of Health Sciences, University of Southampton, Southampton, UK

For peer review only

Arthritis Research UK

Condition
Osteoarthritis

Osteoarthritis

This booklet provides information and answers to your questions about this condition.

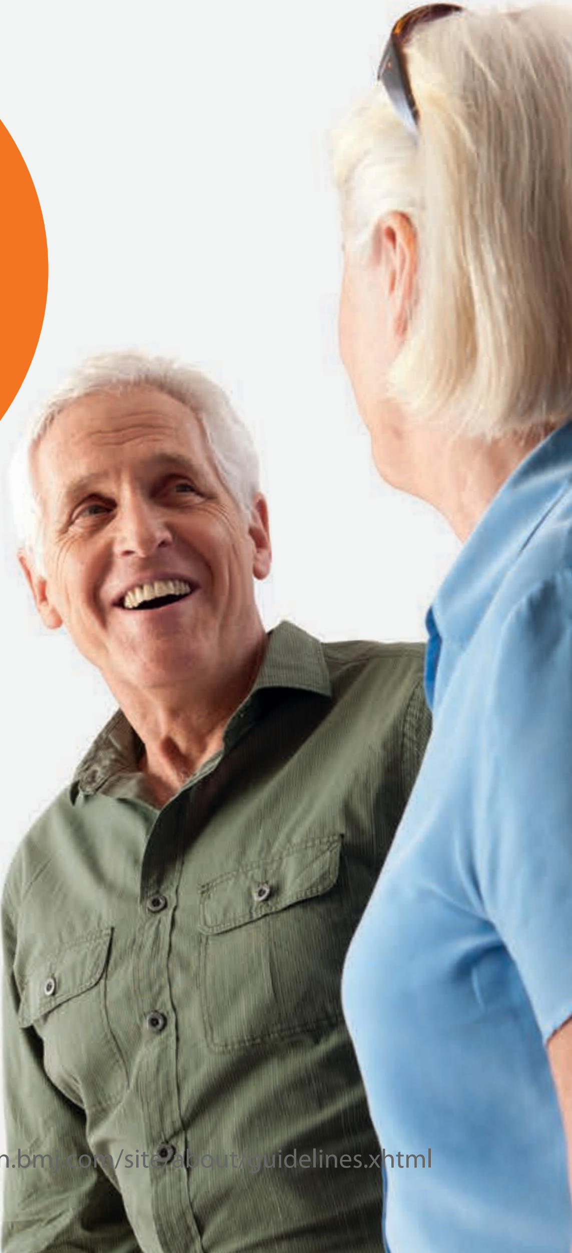

Arthritis Research UK produce and print our booklets entirely from charitable donations.

What is osteoarthritis?

Osteoarthritis is by far the most common form of joint disease. It causes pain and stiffness in the joints and affects approximately 8 million people in the UK. In this booklet we'll explain how osteoarthritis develops, what causes it and how it can be treated. We'll also give some hints and tips to help you manage your arthritis and suggest where you can find out more.

At the back of this booklet you'll find a brief glossary of medical words – we've underlined these when they're first used.

www.arthritisresearchuk.org

For peer review only - http://bmjopen.bmj.com/site/about/guidelines.xhtml

Arthritis Research UK
Osteoarthritis

What's inside?

2 Osteoarthritis at a glance

4 How does a normal joint work?

4 What is osteoarthritis?

6 What are the symptoms of osteoarthritis?

7 What causes osteoarthritis?

8 Which joints are affected?
– The knee
– The hip
– The hand
– The back and neck
– The foot

11 What is the outlook?

12 What are the possible complications?

12 How is osteoarthritis diagnosed?
– What tests are there?

14 What can I do to help myself?
– Exercise
– Weight management
– Tablets and creams
– Reducing the strain on your joints
– Complementary medicine

20 What treatments are there for osteoarthritis?
– Capsaicin cream
– Drugs
– Steroid injections
– Transcutaneous electrical nerve stimulation (TENS)
– Surgery

22 Self-help and daily living

23 Research and new developments

24 Glossary

26 Where can I find out more?

32 We're here to help

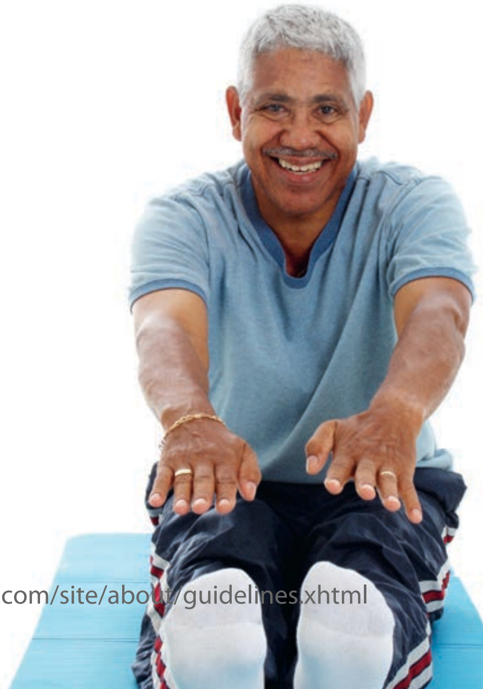

For peer review only - http://bmjopen.bmj.com/site/about/guidelines.xhtml

At a glance
Osteoarthritis

Osteoarthritis is a condition that affects the joints, causing pain and stiffness.

What is osteoarthritis?

Osteoarthritis is a condition that affects the joints, causing pain and stiffness. It's by far the most common form of joint disease, affecting people all over the world and approximately 8 million people in the UK.

What are the symptoms?

Symptoms of osteoarthritis can include:

- pain
- stiffness
- a grating or grinding sensation (crepitus) when the joint moves
- swelling (either hard or soft)
- not being able to use the affected joint normally, which can make it difficult to do certain activities (for example climbing stairs).

Who gets it?

Almost anyone can get osteoarthritis but it's most likely if:

- you're in your late 40s or older
- you're a woman
- your parents have had osteoarthritis
- you're overweight
- you've had a previous joint injury
- you have a physically demanding job where you make repetitive movements
- your joints have been damaged by another disease, for example gout or rheumatoid arthritis.

For peer review only

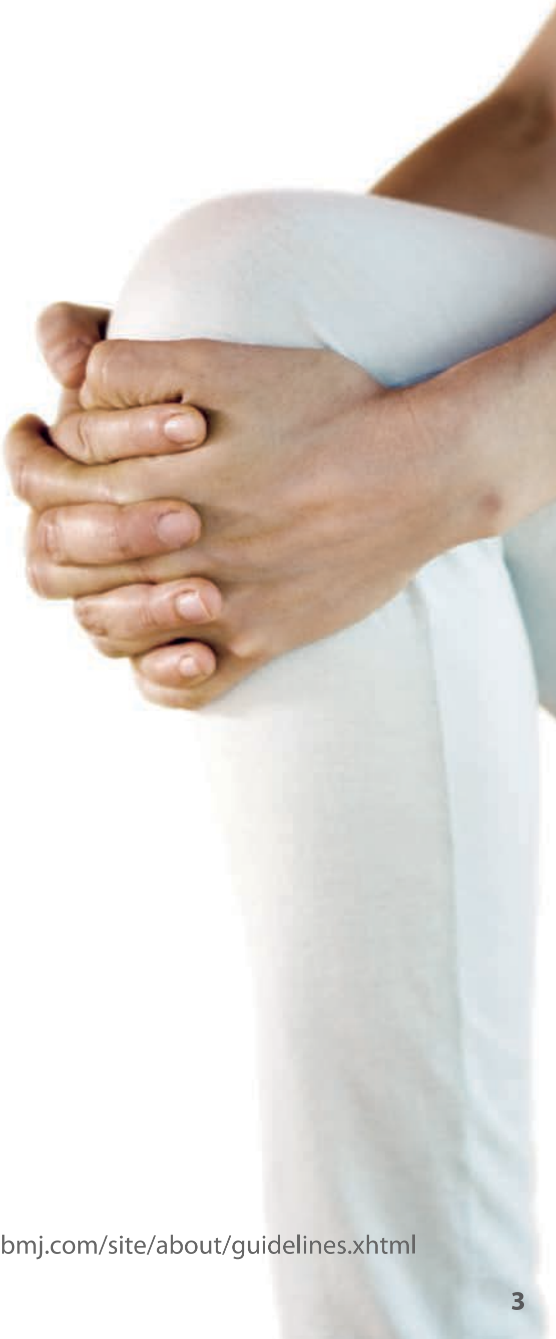

What can I do to help myself?

There are several ways you can help yourself, including:

- exercising regularly (both muscle-strengthening and general aerobic exercise)
- reducing stress on the affected joint (for example by pacing activities, using a walking stick or wearing suitable footwear)
- losing weight if you're overweight
- using over-the-counter painkillers such as paracetamol or low-dose ibuprofen, or pain-relieving creams, gels or sprays.

What treatments are there?

If you still have pain after trying self-help measures, your doctor may recommend the following:

- advice from a physiotherapist about exercise plans and gradually increasing your aerobic exercise
- capsaicin cream
- stronger painkillers, for example tramadol
- steroid injections into the painful joint
- surgery, including joint replacement.

For peer review only - http://bmjopen.bmj.com/site/about/guidelines.xhtml

How does a normal joint work?

A joint is where two or more bones meet (see Figure 1). The joint allows the bones to move freely but within controlled limits. The knees have additional rings of cartilage between the bones. These are called menisci – they act a bit like shock absorbers to spread the load more evenly across the joint.

What is osteoarthritis?

Osteoarthritis is a disease that affects your joints. The surfaces within your joints become damaged so the joint doesn't move as smoothly as it should (see Figures 2 and 3). The condition is sometimes called arthrosis or osteoarthrosis. Older terms are degenerative joint disease or wear and tear.

When a joint develops osteoarthritis, some of the cartilage covering the ends of the bones gradually roughens and becomes thin, and the bone underneath thickens. All the tissues within the joint become more active than normal – as if your body is trying to repair the damage:

- The bone at the edge of the joint grows outwards, forming bony spurs called osteophytes.
- The synovium may thicken and produce extra fluid, which then causes the joint to swell.
- The capsule and ligaments slowly thicken and contract as if they were trying to stabilise the joint.

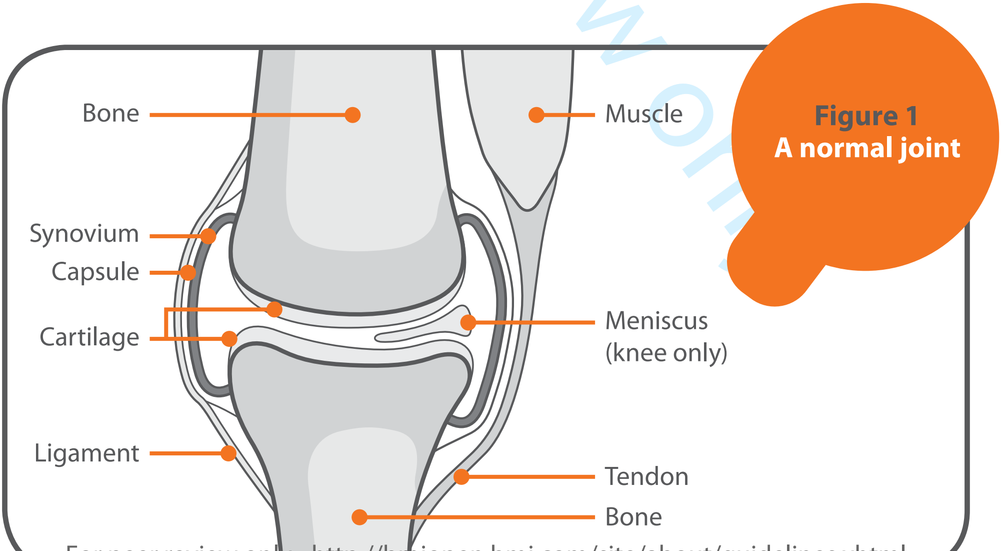

Figure 1
A normal joint

For peer review only - http://bmjopen.bmj.com/site/about/guidelines.xhtml

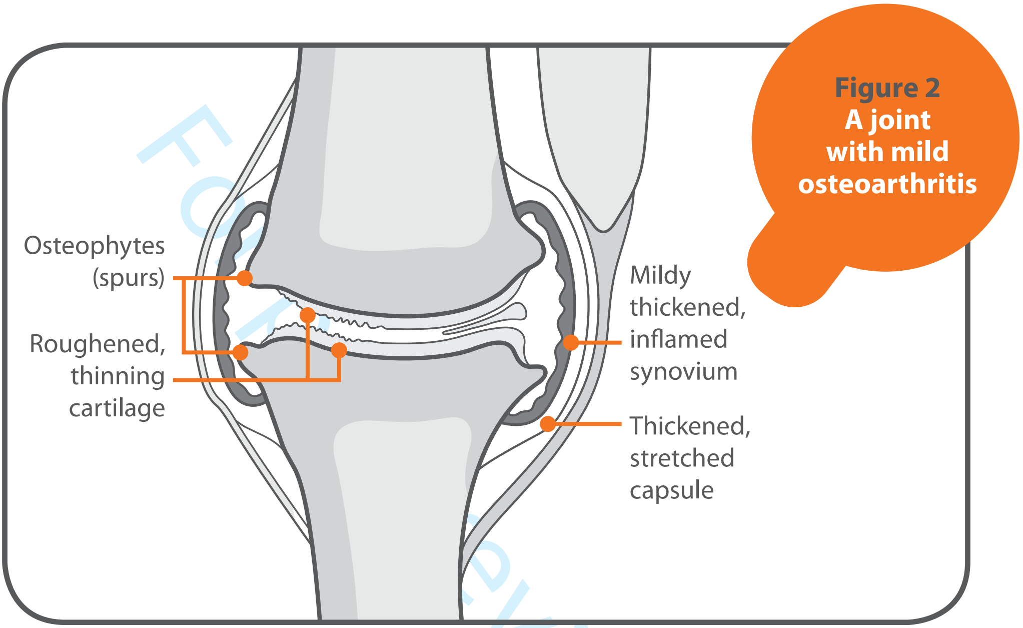

Figure 2
A joint with mild osteoarthritis

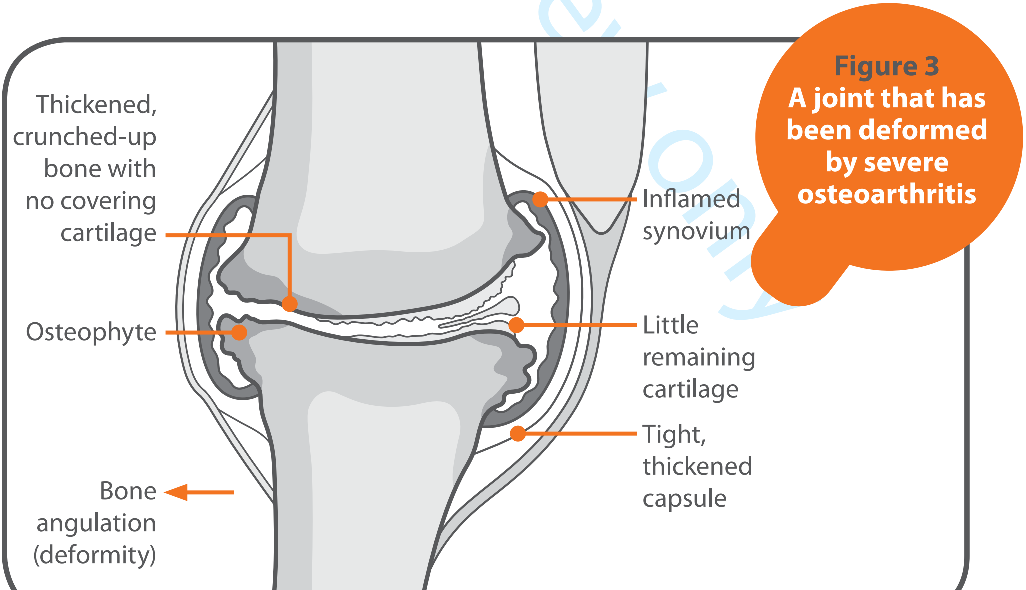

Figure 3
A joint that has been deformed by severe osteoarthritis

For peer review only - http://bmjopen.bmj.com/site/about/guidelines.xhtml

Sometimes your body's repairs are quite successful and the changes inside the joint won't cause pain or problems. In severe osteoarthritis, the cartilage can become so thin that it no longer covers the ends of your bones. The bones start to rub against each other and eventually start to wear away. The loss of cartilage, the wearing of bone and the bony spurs can alter the shape of the joint, forcing the bones out of their normal position.

What are the symptoms of osteoarthritis?

The main symptoms of osteoarthritis are pain and sometimes stiffness in the affected joints. The pain tends to be worse when you move the joint or at the end of the day, and it may make it difficult to get to sleep. Your joints may feel stiff after rest, but this usually wears off after a minute or two as you get moving.

If you have severe osteoarthritis, you may feel pain more often. The joint may not move as freely or as far as normal, and it may creak or crunch as you move. Sometimes it may give way because your muscles have weakened or the joint structure has become less stable, although exercises to strengthen your muscles can help to prevent this.

You may notice that the affected joint looks swollen. The swelling may be hard (caused by osteophytes) or soft (caused by synovial thickening and extra fluid in the joint), and the muscles around the joint may look thin or wasted.

Figure 4 Risk factors for osteoarthritis

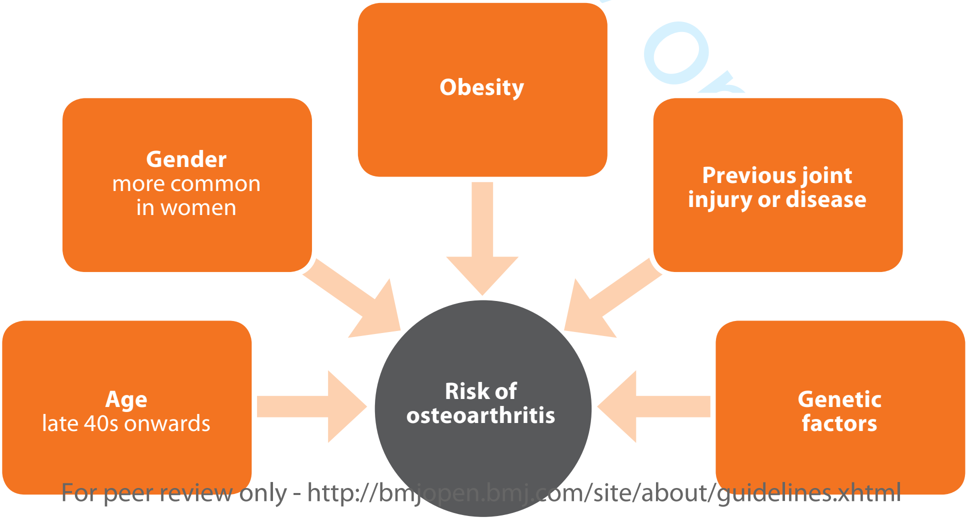

For peer review only - http://bmjopen.bmj.com/site/about/guidelines.xhtml

Symptoms often vary for no obvious reason – you'll probably have good and bad spells. Some people find that changes in the weather make the pain worse, especially damp weather along with falling atmospheric pressure. Others find the pain varies depending on how active they've been.

In more severe cases, the pain may be constant. It may prevent you from sleeping and cause difficulties in your daily activities; for example, osteoarthritis in the knee or hip can make it difficult to climb stairs or get up from a chair.

What causes osteoarthritis?

There are many factors that can increase your risk of osteoarthritis, and it's often a combination of these that leads to the condition (see Figure 4):

- **Age** – Osteoarthritis usually starts from the late 40s onwards. We don't fully understand why it's more common in older people, but it might be due to factors like the muscles weakening and the body being less able to heal itself, or gradual wearing out of the joint with time.
- **Gender** – For most joints, especially the knees and hands, osteoarthritis is more common and more severe in women.
- **Obesity** – Being overweight is an important factor in causing osteoarthritis, especially in the knee. It also increases the chances of osteoarthritis becoming progressively worse.

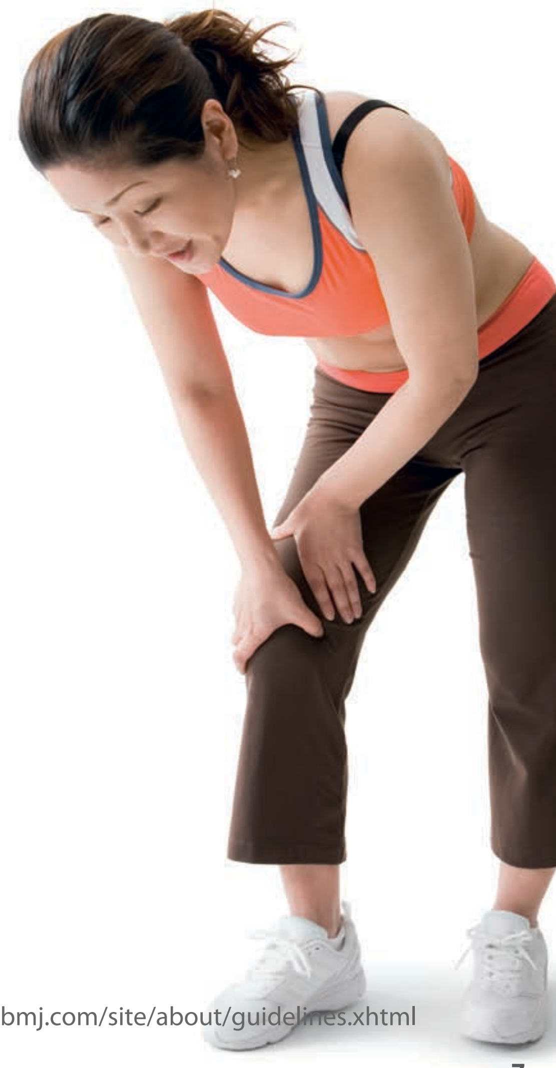

For peer review only - http://bmjopen.bmj.com/site/about/guidelines.xhtml

- **Joint injury** – A major injury or operation on a joint may lead to osteoarthritis in that joint later in life. Normal activity and exercise don't cause osteoarthritis, but very hard, repetitive activity or physically demanding jobs can increase your risk.
- **Joint abnormalities** – If you were born with abnormalities or developed them in childhood, it can lead to earlier and more severe osteoarthritis than usual. Perthes' disease of the hips is an example.
- **Genetic factors** – Nodal osteoarthritis, which particularly affects the hands of middle-aged women, runs strongly in families, although it's not yet clear which genes are involved. And some rare forms of osteoarthritis which start at an earlier age are linked with genes that affect collagen (an essential part of cartilage). Genetic factors play a smaller, but still significant, part in osteoarthritis of the hip and knee.
- **Other types of joint disease** – Sometimes osteoarthritis is a result of damage from a different kind of joint disease, such as rheumatoid arthritis or gout.

Which joints are affected?

Almost any joint can develop osteoarthritis, especially if it's been badly injured, but it most often affects the knees, hips, hands, spine and big toes (see Figure 5):

The knee

Osteoarthritis of the knee is very common. This is probably because your knee has to take extreme stresses, twists and turns.

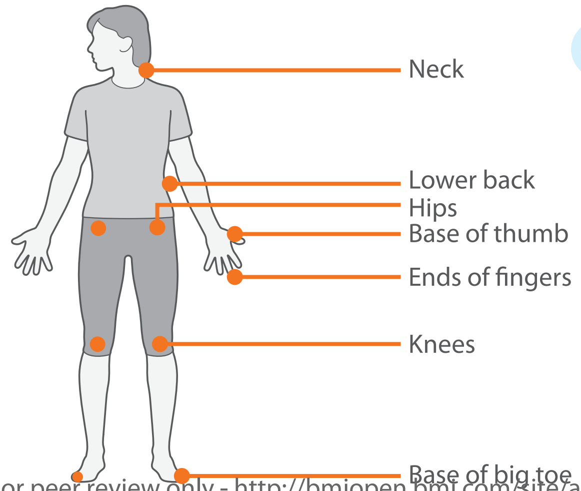

Figure 5 The joints most often affected by osteoarthritis

For peer review only - http://bmjopen.bmj.com/site/about/guidelines.xhtml

Any joint can be affected by osteoarthritis.

Osteoarthritis can affect the main surfaces of the knee joint and also the cartilage underneath your kneecap (patella).

You're most likely to feel pain at the front and sides of your knee. If the osteoarthritis is severe, your knees may become bent and bowed. Your knee joint may also become unstable so that it gives way when you put weight on it. This is usually because of muscle weakness in the thigh but sometimes because of damage to the ligaments.

Osteoarthritis of the knee is twice as common in women as in men and it usually affects both knees. It causes most problems from the late 50s onwards. A number of factors can increase the risk of osteoarthritis of the knee, for example:

- being overweight
- having nodal osteoarthritis (particularly in women)
- a previous sporting injury (such as a torn meniscus or ligament)
- an operation to remove torn cartilage (meniscectomy).

See Arthritis Research UK booklet *Osteoarthritis of the knee.*

The hip

Osteoarthritis of the hip is also very common and can affect either one or both hips. The pain is most likely to be deep at the front of your groin, but you may also feel pain at the side and front of your thigh, in your buttock or down to your knee (this is called referred pain).

If you have severe hip osteoarthritis, you may find the affected leg seems a little shorter than the other because of the bone on either side of the joint being crunched up.

Men and women are equally likely to develop hip osteoarthritis, and it usually starts from the late 40s onwards. The risk may be greater if you had hip problems at birth (congenital dislocation) or abnormal hip development in childhood, such as Perthes' disease. Physical work such as farming may also increase the risk; however, there's often no obvious cause.

The hand

Osteoarthritis of the hands usually occurs as part of nodal osteoarthritis. This mainly affects women and often starts in your 40s or 50s, around the time of the menopause. It usually affects the base of your thumb

For peer review only - http://bmjopen.bmj.com/site/about/guidelines.xhtml

and the joints at the ends of your fingers, although other finger joints can also be affected. At times these joints become swollen and tender, especially when the condition first appears.

Over several years, firm knobbly swellings form on the finger joints. These are caused by osteophytes and are known as Heberden's nodes when they're at the end joints of your fingers (see Figure 6) or Bouchard's nodes when they're at the mid-finger joints. Once the nodes are fully formed, the pain and tenderness often improve. Although the fingers are knobbly and sometimes slightly bent, they usually still work well. Arthritis at the base of your thumb may cause longer-lasting problems.

Having nodal osteoarthritis in middle age means you're more likely to develop osteoarthritis of the knee, and possibly other joints, as you get older. Nodal osteoarthritis tends to run in families much more than other forms of osteoarthritis and it's especially likely to be passed from mother to daughter. It's not yet known which genes are involved so it's not possible to test for this.

The back and neck

Changes that affect the bones of your spine and the discs between the bones are often called spondylosis, but they're very similar to the changes caused by osteoarthritis in other joints. X-rays show that spondylosis is extremely common, but it's not the most common cause of back or neck pain and often doesn't cause any problems at all.

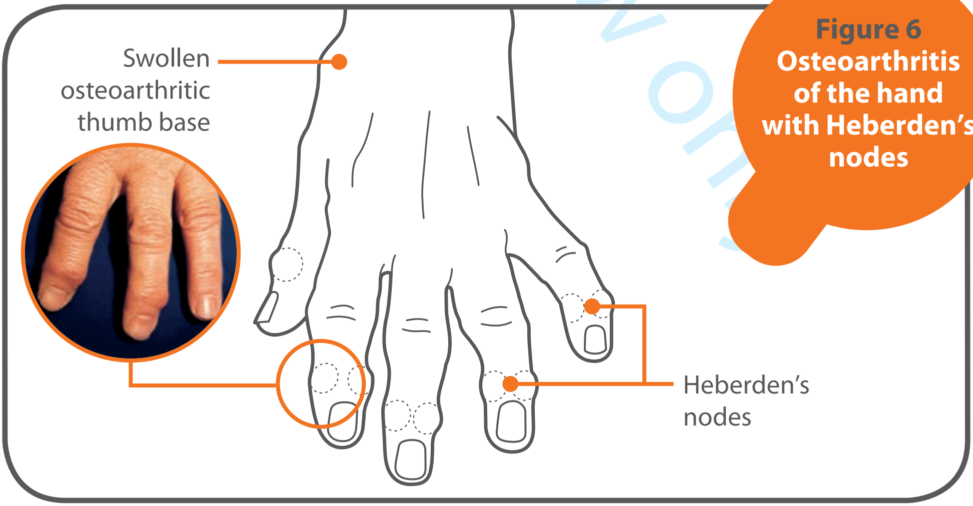

Figure 6 Osteoarthritis of the hand with Heberden's nodes

Photography used with kind permission of Elsevier. © Elsevier 2011. Taken from *Rheumatology*, fifth edition. Edited by Marc C Hochberg, Alan J Silman, Josef S Smolen, Michael E Weinblatt and Michael H Weisman.

For peer review only - http://bmjopen.bmj.com/site/about/guidelines.xhtml

See Arthritis Research UK booklets *Back pain; Neck pain.*

The foot

Osteoarthritis of the foot generally affects the joint at the base of your big toe. Eventually your toe may become stiff (hallux rigidus), which can make it difficult and painful to walk, or bent (hallux valgus), which can lead to painful bunions (see Figure 7).

Osteoarthritis of the mid-foot is also quite common, especially in older people, and may cause an obvious bony swelling (osteophyte) on the top of your mid-foot. Ankle osteoarthritis is least common and may cause your heel to move to an unusual angle.

See Arthritis Research UK booklets *Feet, footwear and arthritis.*

What is the outlook?

It's impossible to predict how osteoarthritis will develop for any one person. It can sometimes develop over just a year or two and cause a lot of damage to a joint, which may then cause some deformity or disability. But more often osteoarthritis is a slow process that develops over many years and results in fairly small changes in just part of the joint. This doesn't mean it won't be painful, but it's less likely to cause severe deformity or disability. Sometimes the condition reaches a peak a few years after the symptoms start and then remains the same, or it may even improve.

Unlike rheumatoid arthritis, osteoarthritis doesn't affect other parts of your body – it's purely a joint disease. Also, because there's very little, if any, inflammation in osteoarthritic joints, the condition doesn't

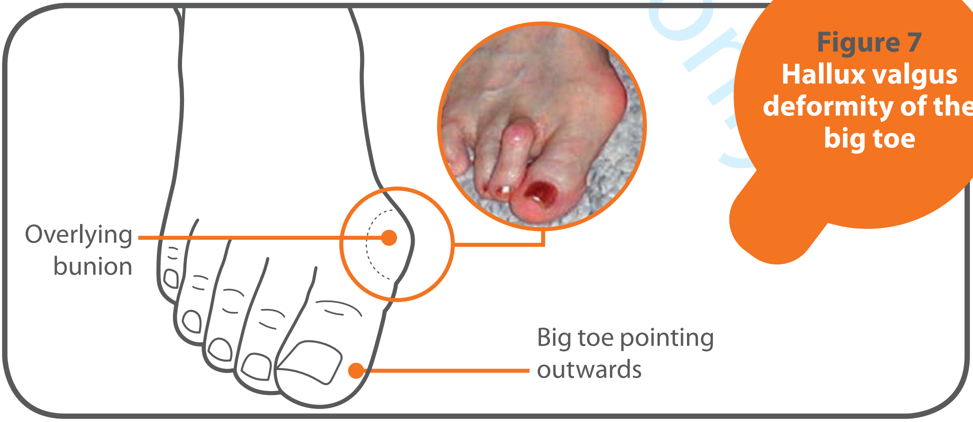

Figure 7 Hallux valgus deformity of the big toe

Photography used with kind permission of Elsevier. © Elsevier 2011. Taken from *Rheumatology*, fifth edition. Edited by Marc C Hochberg, Alan J Silman, Josef S Smolen, Michael E Weinblatt and Michael H Weisman.

For peer review only - http://bmjopen.bmj.com/site/about/guidelines.xhtml

make you feverish or unwell. However, some people with osteoarthritis will develop other illnesses purely by chance.

What are the possible complications?

The changes in cartilage that occur with osteoarthritis can encourage crystals to form within the joint. Gout is a common type of inflammatory arthritis, which is caused by high levels of uric acid that lead to sodium urate crystals forming in and around joints. If you have both osteoarthritis and a high serum uric acid, you're at an increased risk of developing gout. The base of the big toe is a very common site for a painful attack of gout, and this is partly because this joint is the most common joint in the foot to be affected by osteoarthritis.

See Arthritis Research UK booklet *Gout.*

Osteoarthritis doesn't lead to rheumatoid arthritis or other types of joint disease

Osteoarthritis can also encourage calcium pyrophosphate crystals to form in the cartilage. This is called calcification or chondrocalcinosis. It can happen in any joint, with or without osteoarthritis, but it's most likely to occur in a knee already affected by osteoarthritis, especially in older people. The crystals will show up in x-rays and they can also be seen under a microscope in samples of fluid taken from the joint.

Osteoarthritis tends to become more severe more quickly when there are calcium crystals present. Sometimes the crystals can shake loose from the cartilage, causing a sudden attack of very painful swelling called acute calcium pyrophosphate crystal arthritis (acute CPP crystal arthritis), which was sometimes previously called 'pseudogout'.

See Arthritis Research UK booklet *Calcium crystal diseases including acute CPP crystal arthritis (pseudogout) and acute calcific tendinitis.*

How is osteoarthritis diagnosed?

It's very important to get an accurate diagnosis if you think you have arthritis. There are many different types of arthritis and some need very different treatments. Osteoarthritis is usually diagnosed based on your symptoms and the physical signs that your doctor finds when examining your joints. The signs your doctor will be checking for are:

For peer review only

For peer review only - http://bmjopen.bmj.com/site/about/guidelines.xhtml

- tenderness over the joint
- creaking or grating of the joint (crepitus)
- bony swelling
- excess fluid
- restricted movement
- joint instability
- thinning of the muscles that support the joint.

What tests are there?

There's no blood test for osteoarthritis, although your doctor may suggest you have them to help rule out other types of arthritis.

X-rays are the most useful test to confirm osteoarthritis, although they often won't be needed. An x-ray may show changes such as bony spurs or narrowing of the space between the bones. They'll also show whether there are any calcium deposits within the joint.

However, x-rays can't really show how much pain or disability you're likely to have. Some people have a lot of pain from fairly minor joint damage, while others have little pain from more severe damage.

Rarely, a magnetic resonance imaging (MRI) scan of the knee can be helpful. This will show the soft tissues (for example cartilage, tendons, muscles) and changes in the bone that can't be seen on a standard x-ray. Its main use is to identify another joint or bone problem in someone whose symptoms aren't typical of osteoarthritis, for example, in a person with a torn knee meniscus that causes intermittent 'locking' of the knee.

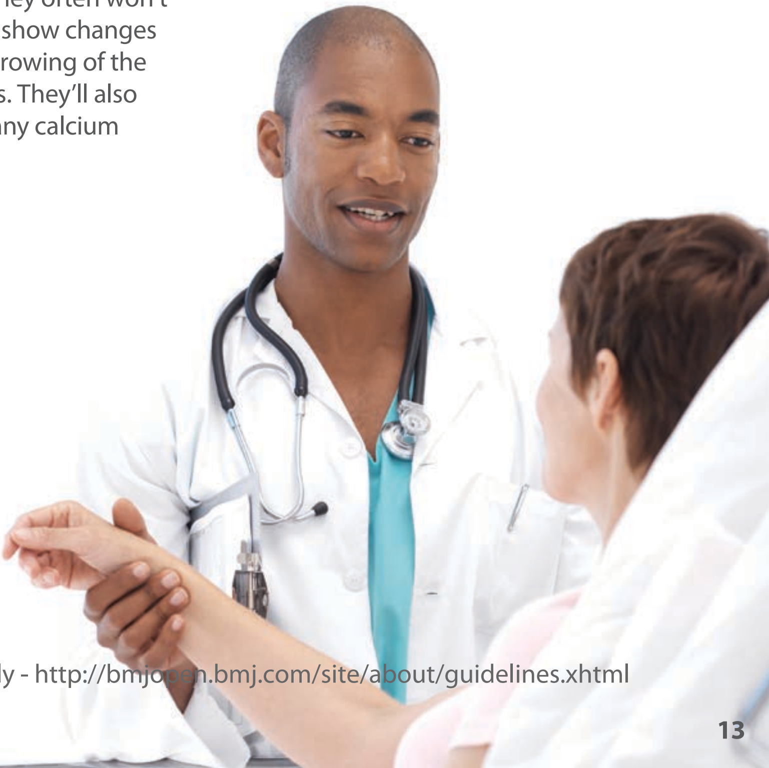

For peer review only - http://bmjopen.bmj.com/site/about/guidelines.xhtml

What can I do to help myself?

There's no cure for osteoarthritis as yet, but there's a lot that you can do to improve your symptoms. Self-help measures play a very important part in relieving the pain and stiffness, and reducing the chances of your arthritis becoming worse.

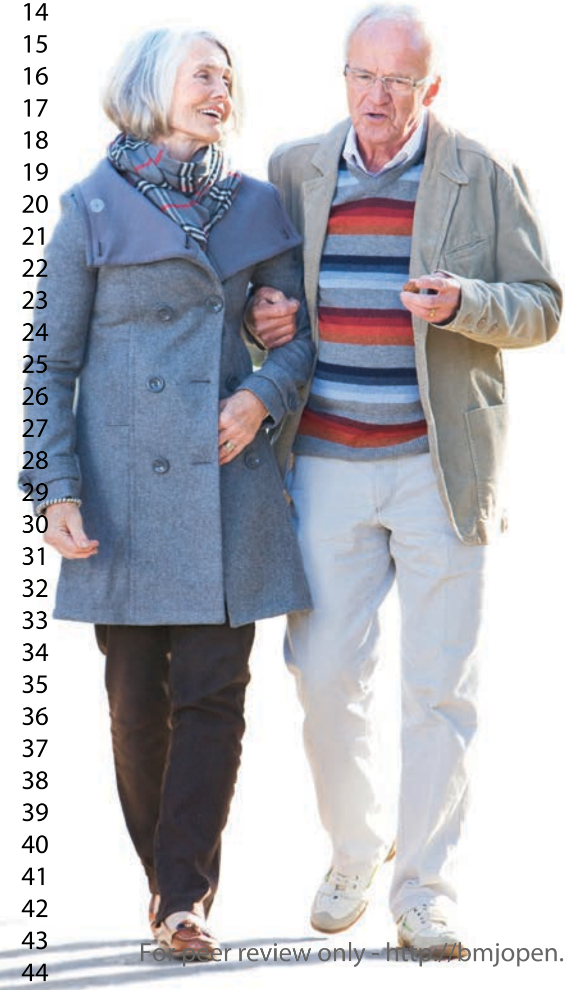

Exercise

It's very important to keep your joints moving. You'll need to find the right balance between rest and exercise – most people with osteoarthritis find that too much activity increases their pain while too little makes their joints stiffen up. Little and often is usually the best approach to exercise if you have osteoarthritis.

There are two main types of exercise that you'll need to do:

Strengthening exercises will improve the strength and tone of the muscles that control the affected joint. Osteoarthritis can weaken these muscles. This is particularly important for the thigh (quadriceps) muscles if you have osteoarthritis of the knee. Regular exercising of the muscles, such as straight-leg raises (see the pull-out section at the back of the booklet), helps to stabilise and protect the joint and has also been shown to reduce pain. It's also particularly helpful in preventing the knee giving way and reducing the chances of stumbling or falling.

Because knee and hip osteoarthritis may come to affect both sides of your body, and because both legs work as a unit when you walk, it's helpful to do strengthening exercises on the muscles on the other leg, even if that knee or hip isn't causing symptoms, and also to do hip exercises if you have knee osteoarthritis (and vice versa).

For peer review only - http://bmjopen.bmj.com/site/about/guidelines.xhtml

Arthritis Research UK
Osteoarthritis

Aerobic exercise is any exercise that increases your pulse rate and makes you a bit short of breath (for example a brisk walk, swimming or using an exercise bike). Regular aerobic exercise should help you sleep better, is good for your general health and well-being, and can also reduce pain by raising the levels of pain-relieving hormones called endorphins.

A physiotherapist can advise you on the best exercises for the type of osteoarthritis you have, but you'll need to build them into your daily routine to get the most from them. You can also talk to your GP about the Exercise on Prescription scheme that's available in some areas.

Swimming can be very good for osteoarthritis. Because the water supports the weight of your body, you won't be putting a lot of strain on your joints as you exercise. Your physiotherapist may also recommend special exercises in a hydrotherapy pool. This can help get muscles and joints working better and, because the water is warmer than in a typical swimming pool, it can be very soothing and relaxing.

T'ai chi is another type of exercise that has been shown to be helpful at reducing the pain from osteoarthritis. Many people find that regular t'ai chi also makes them feel better in other ways, for example, less stressed during the day and better able to sleep at night.

See Arthritis Research UK booklets
Hydrotherapy and arthritis; Keep moving; Physiotherapy and arthritis.

Weight management

There's a great deal of evidence that being overweight increases the strain on your joints – especially your knees. Research shows that being overweight or obese not only increases your risk of developing osteoarthritis but also makes it more likely that your arthritis will get worse over time.

Because of the way the joints work, the force put through your knees when you walk, especially on stairs and slopes, can be several times your actual body weight. Losing even a small amount of weight can make a big difference to the strain on weight-bearing joints.

No special diet has been shown to help with osteoarthritis, but if you need to lose some weight we would recommend a balanced, reduced-calorie diet combined with regular exercise.

See Arthritis Research UK booklet
Diet and arthritis.

Tablets and creams

There are a number of tablets and creams that can help osteoarthritis symptoms, and because they work in different ways you can combine different treatments if you need to. Your pharmacist can advise you and supply paracetamol, and some low-dose tablets and creams without a prescription.

For peer review only - http://bmjopen.bmj.com/site/about/guidelines.xhtml

Anti-inflammatory creams and gels are especially helpful for osteoarthritis of the knee or hand.

Painkillers (analgesics) and non-steroidal anti-inflammatory drugs (NSAIDs)

Painkillers (analgesics) often help with the pain and stiffness, although they don't affect the arthritis itself and won't repair damage to the joint. They're best used occasionally when the pain is very bad or when you're likely to be exercising. Paracetamol is usually the best and safest painkiller to try first, but make sure you take the right dose as most people take too little. You should try taking 1 g (usually two tablets) three or four times a day. It's best to take them before the pain becomes very bad but you shouldn't take them more often than every four hours.

Combined painkillers (for example co-codamol) contain paracetamol and a second codeine-like drug, and they may be helpful for more severe pain. They're stronger than paracetamol and are therefore more likely to cause side-effects such as constipation or dizziness.

Over-the counter non-steroidal anti-inflammatory drugs (NSAIDs), such as ibuprofen, can also help. You can use these for a short course of treatment (about 5–10 days), but if they've not helped within this time then they're unlikely to. If the pain returns when you stop taking the tablets, try another short course.

If you're already taking NSAID tablets, speak to your doctor about non-NSAID creams (for example capsaicin cream) to avoid taking too much of one type of drug.

! You shouldn't take ibuprofen or aspirin if you're pregnant, or if you have asthma, indigestion or a stomach (gastrointestinal) ulcer, until you've spoken with your doctor or pharmacist.

If you know you're going to be more active than usual, try taking a painkiller before you start to avoid increased pain later.

---

i **See Arthritis Research UK leaflets** *Non-steroidal anti-inflammatory drugs; Painkillers.*

Anti-inflammatory creams and gels

You can apply anti-inflammatory creams and gels directly onto painful joints three times a day. There's no need to rub them in – they absorb through the skin on their own. They're especially helpful for osteoarthritis of the knee or hand but not for deep joints such as the hip. They're extremely well tolerated as very little is absorbed into the bloodstream. If you have trouble taking tablets then anti-inflammatory creams are a particularly

For peer review only - http://bmjopen.bmj.com/site/about/guidelines.xhtml

good option to try. You can decide if they help your pain within the first few days of trying them.

Reducing the strain on your joints

Apart from keeping an eye on your weight, there are a number of other ways you can reduce the strain on your joints:

- Pace your activities through the day – don't tackle all the physical jobs at once. Break the harder jobs up into chunks and do something more gentle in between.
- Wear low-heeled shoes with soft, thick soles (trainers are ideal). Thicker soles will act as shock absorbers for your feet, knees, hips and back. High heels will alter the angle of your hips, knees and big toe joints and put additional strain on them.
- Use a walking stick to reduce the weight and stress on a painful hip or knee. A therapist or doctor can advise on the correct length and how to put your weight through the stick when you're putting weight on your affected joint.
- Use the handrail for support when climbing stairs – this is particularly important if you have osteoarthritis of the knee.
- Keep your joints moving – in particular, don't keep an osteoarthritic knee still in a bent position for too long as this will eventually affect the muscles.

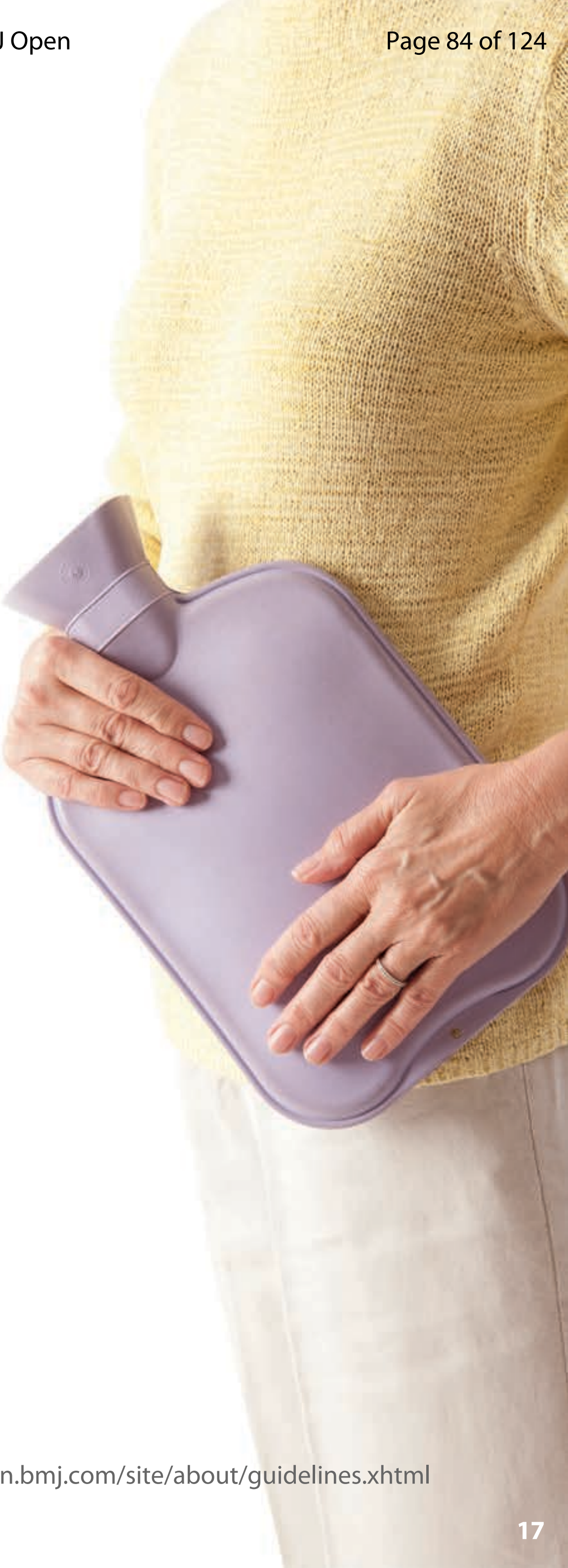

For peer review only - http://bmjopen.bmj.com/site/about/guidelines.xhtml

Reducing strain on your joints can help with osteoarthritis.

- Think about modifying your home, car or workplace to reduce unnecessary strain on your joints. An occupational therapist can advise you on how to protect your joints and on special equipment or gadgets that will make your daily tasks easier.
- Learn to relax your muscles and get the tension out of your body. A physiotherapist or occupational therapist can advise you on relaxation techniques.
- Sex can sometimes be painful, particularly for women with osteoarthritis of the hips. Trying a different position can often help.

See Arthritis Research UK booklets *Looking after your joints when you have arthritis; Occupational therapy and arthritis; Sex and arthritis.*

Applying warmth to a painful joint often relieves the pain and stiffness of osteoarthritis. Heat lamps are popular, but a hot-water bottle or reheatable pad are just as effective. This can be helpful if you have a flare-up of pain when you've done a bit too much. An ice pack can also help, but don't apply either ice or heat packs directly to your skin.

More evidence to support the use of knee braces for osteoarthritis is becoming available. There are several types that can help to stabilise the kneecap and make it move correctly. You can buy knee braces from sports shops and chemists, but you should speak to your doctor or physiotherapist first. They may also be able to provide braces or recommend the best ones for you.

Complementary medicine

There are many different complementary and herbal remedies that claim to help with arthritis, and some people do feel better when they use some complementary treatments. However, on the whole these treatments aren't recommended for use on the NHS because there's no clear evidence that they work. There's no scientific evidence, for example, that copper bracelets can help ease osteoarthritis-related pain.

Glucosamine and chondroitin

Many people try glucosamine and chondroitin tablets. These are compounds that are normally found in joint cartilage, and some studies suggest that taking supplements may improve the health of damaged cartilage. Other studies, however, don't show any benefit so we still

For peer review only - http://bmjopen.bmj.com/site/about/guidelines.xhtml

don't know for sure whether they work or not. Glucosamine and chondroitin, which are similar to each other, are available from your chemist or health food store. You'll need to take a dose of 1.5 g glucosamine sulphate a day, and you may need to take them for several weeks before you can tell whether they're making a difference.

Glucosamine hydrochloride doesn't appear to be effective, so always check that you're taking the sulphate variety.

Most brands of glucosamine are made from shellfish. If you're allergic to shellfish, make sure you use a vegetarian or shellfish-free variety. Glucosamine can affect the level of sugar in your blood, so if you have diabetes you should keep an eye on your blood sugar levels and see your doctor if they increase. You should also see your doctor for regular blood checks if you're taking the blood-thinning drug warfarin.

Homeopathy

Many people are interested in homeopathic remedies, and a number of different types are used for osteoarthritis; however, there's no conclusive scientific evidence that the remedies are effective.

Acupuncture

There's some research showing that acupuncture can sometimes provide relief from arthritis pain, although the effect may be short-lived, which means that you'll need repeat sessions. Other studies show no benefit from acupuncture.

Chiropractic and osteopathy

Manipulation by a chiropractor or osteopath can often help neck and back pain, although the use of manipulation for osteoarthritis in other joints is limited. If you do want to try it, make sure you choose a practitioner who is registered with the appropriate regulatory body.

See Arthritis Research UK booklet and special reports
Complementary and alternative medicine for arthritis; Complementary and alternative medicines for the treatment of rheumatoid arthritis, osteoarthritis and fibromyalgia; Practitioner-based complementary and alternative therapies for the treatment of rheumatoid arthritis, osteoarthritis, fibromyalgia, and low back pain.

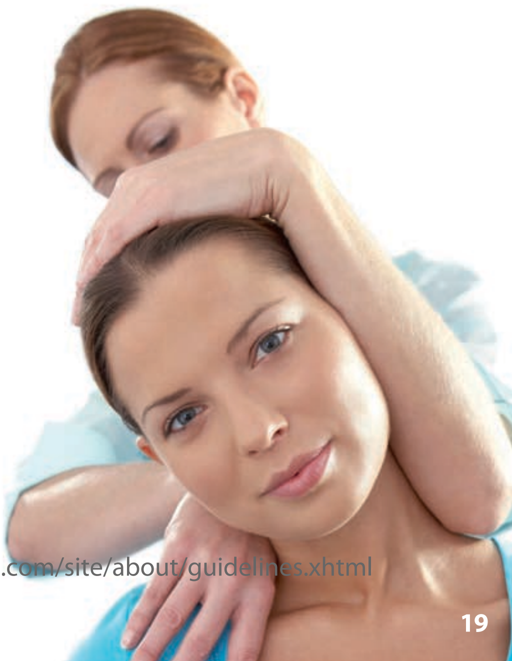

For peer review only - http://bmjopen.bmj.com/site/about/guidelines.xhtml

What treatments are there for osteoarthritis?

Many people find that self-help measures, such as those listed above, are enough to help them manage their symptoms, but your healthcare team will be able to suggest other treatments if you need them.

Capsaicin cream

Capsaicin cream is made from the pepper plant (capsicum) and is an effective and very well-tolerated painkiller. It needs to be applied regularly three times each day to be effective and, like NSAID creams and gels, it's particularly useful for knee and hand osteoarthritis. It's only available on prescription.

Most people feel a warming or burning sensation when they first use capsaicin, but this generally wears off after several days. The pain-relieving effect starts after several days of regular use and you should try it for at least two weeks before deciding if it has helped.

Drugs

Painkillers

If you have severe pain and other medications aren't giving enough relief, your doctor may recommend stronger painkillers (or opioids) such as tramadol, nefopam or meptazinol. Stronger painkillers are more likely to have side-effects – especially nausea, dizziness and confusion – so you'll need to see your doctor regularly and report any problems you have with these drugs.

Some opioids (for example fentanyl) can be given as a plaster patch which you place on your skin – these can give pain relief for a number of days.

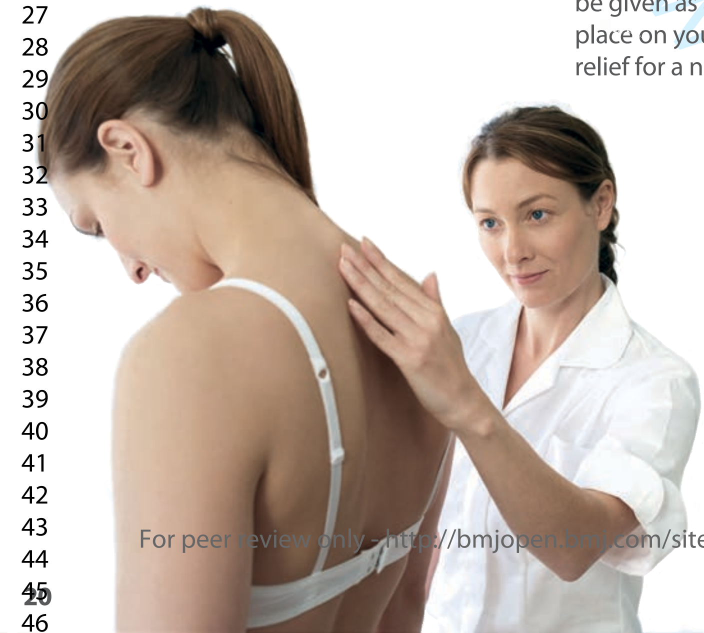

For peer review only - http://bmjopen.bmj.com/site/about/guidelines.xhtml

Non-steroidal anti-inflammatory drugs (NSAIDs)

If inflammation in the joint is causing pain and stiffness, a short course of NSAID tablets (for example naproxen) may be useful.

Like all drugs, NSAIDs can sometimes have side-effects, but your doctor will take precautions to reduce the risk of these – for example, by prescribing the lowest effective dose for the shortest possible period of time.

NSAIDs can cause digestive problems (stomach upsets, indigestion, or damage to the stomach lining) so in most cases they'll be prescribed along with a drug called a proton pump inhibitor (PPI), which will help to protect your stomach.

NSAIDs also carry an increased risk of heart attack or stroke. Although the increased risk is small, your doctor will be cautious about prescribing them if there are other factors that may increase your overall risk – for example, smoking, circulation problems, high blood pressure, high cholesterol or diabetes. NSAIDS can also reduce kidney function so you shouldn't take them if you have known reduced kidney function or are on a water tablet (diuretic).

! If you have trouble opening child-proof containers, your pharmacist will put your tablets in a more suitable container for you. Contact us for our special request card which you can hand to your pharmacist with your prescription.

Steroid injections

Steroid injections are sometimes given directly into a particularly painful joint. The injection can start to work within a day or so and may improve pain for several weeks or even months, especially in your knee or thumb. This is mainly used for very painful osteoarthritis, for sudden, painful attacks caused by the shedding of calcium pyrophophate crystals, or to help people through an important event (such as a holiday or family wedding).

i **See Arthritis Research UK drug leaflet** *Local steroid injections.*

Steroid injections can improve pain for several weeks or even months.

For peer review only - http://bmjopen.bmj.com/site/about/guidelines.xhtml

For peer review only

Transcutaneous electrical nerve stimulation (TENS)

Some people find that transcutaneous electrical nerve stimulation (TENS) can help to relieve pain, although research evidence on its effectiveness is mixed. A TENS machine is a small electronic device that sends pulses to your nerve endings via pads placed on your skin. It produces a tingling sensation and is thought to alter pain messages sent to the brain. TENS machines are available from pharmacies and other major stores, but a physiotherapist may be able to loan you one to try before you decide whether to buy one.

Surgery

Surgery may be recommended if your pain is very severe or you have mobility problems. Many thousands of hip and knee replacements are performed each year for osteoarthritis, and other joint replacements are becoming increasingly common. Surgery can be very good for easing pain in cases where other treatments haven't given enough relief.

Sometimes keyhole surgery techniques may be used to wash out loose fragments of bone and other tissue from your knee – this is called arthroscopic lavage and it's not recommended unless your knee locks.

See Arthritis Research UK booklets
Foot and ankle surgery; Hand and wrist surgery; Hip replacement surgery; Knee replacement surgery; Shoulder and elbow joint replacement.

Self-help and daily living

Sleep

If pain is a problem at night, heat may help. Try a hot bath before going to bed, or use a hot-water bottle, wheat bag (which you can heat in a microwave) or electric blanket. Taking a painkiller two hours before going to bed can ease night-time pain so you can get to sleep more easily.

See Arthritis Research UK booklet
Sleep and arthritis.

Work

Most people with osteoarthritis are able to continue in their jobs, although you may need to make some alterations to your working environments, especially if you have a physically demanding job. Speak to your employer's occupational health service if they have one, or your local Jobcentre Plus can put you in touch with Disability Employment Advisors who can arrange work assessments. They can advise you on changing the way you work and on equipment that may help you to do your job more easily. If necessary, they can also help with retraining for more suitable work.

See Arthritis Research UK booklet
Work and arthritis.

Dealing with stress

Living with a long-term condition like osteoarthritis can lower your morale and may affect your sleep. It's important to

For peer review only - http://bmjopen.bmj.com/site/about/guidelines.xhtml

tackle problems like these as they could lead to depression and will certainly make the osteoarthritis more difficult to cope with. It often helps to talk about negative feelings, so it could be useful to speak to your healthcare team, or your family and friends. Support groups are also available – your doctor may be able to tell you about organisations in your area.

See Arthritis Research UK booklets and guide *Fatigue and arthritis; Pain and arthritis; Living with long-term pain: a guide to self-management.*

Research and new developments

Research has already shown the importance of exercise and weight management in reducing the pain of osteoarthritis, particularly of the knee. There are many studies going on around the world to find and test new treatments for osteoarthritis. These include studies funded by Arthritis Research UK looking into the benefits of vitamin D (the VIDEO study) and a large national study to find the genes responsible for causing osteoarthritis (the arcOGEN study) which could lead to new therapies. Arthritis Research UK are also funding early trials of stem cell research, which aims to regenerate cartilage using the body's own cells.

Research has already shown the importance of exercise and weight management in reducing the pain of osteoarthritis, particularly of the knee.

For peer review only - http://bmjopen.bmj.com/site/about/guidelines.xhtml

Glossary

Acupuncture – a method of obtaining pain relief which originated in China. Very fine needles are inserted, virtually painlessly, at a number of sites (called meridians) but not necessarily at the painful area. Pain relief is obtained by interfering with pain signals to the brain and by causing the release of natural painkillers (called endorphins).

Aerobic exercise – any exercise that increases your pulse rate and makes you a bit short of breath.

Analgesics – painkillers. As well as dulling pain they lower raised body temperature, and most of them reduce inflammation.

Bunion – a bony lump on the side of the big toe caused by hallux valgus. Sometimes a swelling or bursa on the foot is also called a bunion.

Cartilage – a layer of tough, slippery tissue that covers the ends of the bones in a joint. It acts as a shock absorber and allows smooth movement between bones.

Chiropractor – a trained specialist who treats mechanical disorders of the musculoskeletal system, often through spine manipulation or adjustment. The General Chiropractic Council regulates the practice of chiropractic in the UK.

Collagen – the main substance in the white, fibrous connective tissue that's found in tendons, ligaments and cartilage. This very important protein is also found in skin and bone.

Gout – an inflammatory arthritis caused by a reaction to the formation of urate crystals in the joint. Gout comes and goes in severe flare-ups at first, but if not treated it can eventually lead to joint damage. It often affects the big toe.

Hallux rigidus – osteoarthritis of the big toe joint with a stiff, often painful, big toe.

Hallux valgus – a condition in which the big toe pushes across towards the other toes. It can cause deformities such as bunions and hammer toes.

Hydrotherapy – exercises that take place in water (usually a warm, shallow swimming pool or a special hydrotherapy bath) which can improve mobility, help relieve discomfort and promote recovery from injury.

Inflammation – a normal reaction to injury or infection of living tissues. The flow of blood increases, resulting in heat and redness in the affected tissues, and fluid and cells leak into the tissue, causing swelling.

Ligaments – tough, fibrous bands anchoring the bones on either side of a joint and holding the joint together. In the spine they're attached to the vertebrae and restrict spinal movements, therefore giving stability to the back.

Magnetic resonance imaging (MRI) scan – a type of scan that uses high-frequency radio waves in a strong magnetic field to build up pictures of the inside of the body. It works by detecting water molecules in the body's tissue that give

For peer review only - http://bmjopen.bmj.com/site/about/guidelines.xhtml

out a characteristic signal in the magnetic field. An MRI scan can show up soft-tissue structures as well as bones.

Manipulation – a type of manual therapy used to adjust parts of the body, joints and muscles to treat stiffness and deformity. It's commonly used in physiotherapy, chiropractic, osteopathy and orthopaedics.

Menisci (singular meniscus) – rings of cartilage, like washers, lying between the cartilage-covered bones in the knee. They act as shock absorbers and help the movement of the joint. Each knee has an inside (medial) and an outside (lateral) meniscus.

Menopause – the time when menstruation ends, usually when a woman is in her 50s. This means the ovaries stop releasing eggs every four weeks, and it's no longer possible to have children. If this happens before the age of 45, it's known as premature menopause.

Nodal osteoarthritis – a form of osteoarthritis that often runs in families, characterised by knobbly finger swellings (Heberden's nodes) and a tendency to get osteoarthritis in several joints (especially knees, big toes).

Non-steroidal anti-inflammatory drugs (NSAIDs) – a large family of drugs prescribed for different kinds of arthritis that reduce inflammation and control pain, swelling and stiffness. Common examples include ibuprofen, naproxen and diclofenac.

Occupational therapist – a trained specialist who uses a range of strategies and specialist equipment to help people to reach their goals and maintain their independence by giving practical advice on equipment, adaptations or by changing the way you do things (such as learning to dress using one handed methods following hand surgery).

Osteopath – a trained specialist who treats spinal and other joint problems by manipulating the muscles and joints in order to reduce tension and stiffness, and so help the spine to move more freely. The General Osteopathic Council regulates the practice of osteopathy in the UK.

Osteophytes – an overgrowth of new bone around the edges of osteoarthritic joints. Spurs of new bone can alter the shape of the joint and may press on nearby nerves.

Perthes' disease – inflammation at the head of the thigh bone (femur) that causes pain and limping, usually in boys aged 5–10 years. It can restrict blood supply to the bone leading to poor growth and deformity and can cause osteoarthritis to develop in later life.

Physiotherapist – a trained specialist who helps to keep your joints and muscles moving, helps ease pain and keeps you mobile.

For peer review only - http://bmjopen.bmj.com/site/about/guidelines.xhtml

Proton pump inhibitor (PPI) – a drug that acts on an enzyme in the cells of the stomach to reduce the secretion of gastric acid. They're often prescribed along with non-steroidal anti-inflammatory drugs (NSAIDs) to reduce side-effects from the NSAIDs.

Referred pain – pain that occurs in a different part of the body from that affected by injury or disease (for example, pain in the thigh or knee resulting from osteoarthritis of the hip). This is sometimes called radiated pain.

Rheumatoid arthritis – a common inflammatory disease affecting the joints, particularly the lining of the joint. It most commonly starts in the smaller joints in a symmetrical pattern – that is, for example, in both hands or both wrists at once.

Spondylosis – the term used to describe mechanical or degenerative changes in the small joints in the neck and back. Most of us will have some degeneration in these joints, which can be seen on x-rays, although often these changes don't cause any problems or symptoms.

Synovium – the inner membrane of the joint capsule that produces synovial fluid.

Transcutaneous electrical nerve stimulation (TENS) – a small battery-driven machine which can help to relieve pain. Small pads are applied over the painful area and low-voltage electrical stimulation produces a pleasant tingling sensation, which relieves pain by interfering with pain signals to the brain.

Where can I find out more?

If you've found this information useful you might be interested in these other titles from our range:

Conditions

- *Back pain*
- *Calcium crystal diseases including acute CPP crystal arthritis (pseudogout) and acute calcific tendinitis*
- *Gout*
- *Neck pain*
- *Osteoarthritis of the knee*

Therapies

- *Hydrotherapy and arthritis*
- *Occupational therapy and arthritis*
- *Physiotherapy and arthritis*

Self-help and daily living

- *Complementary and alternative medicine for arthritis*
- *Complementary and alternative medicines for the treatment of rheumatoid arthritis, osteoarthritis and fibromyalgia (63-page special report)*
- *Diet and arthritis*
- *Fatigue and arthritis*
- *Feet, footwear and arthritis*
- *Gardening and arthritis*
- *Keep moving*
- *Living with long-term pain: a guide to self-management*
- *Looking after your joints when you have arthritis*

For peer review only - http://bmjopen.bmj.com/site/about/guidelines.xhtml

Arthritis Research UK
Osteoarthritis

- *Pain and arthritis*
- *Practitioner-based complementary and alternative therapies for the treatment of rheumatoid arthritis, osteoarthritis, fibromyalgia, and low back pain (66-page special report)*
- *Sex and arthritis*
- *Sleep and arthritis*
- *What is arthritis?*
- *Work and arthritis*

Surgery

- *Foot and ankle surgery*
- *Hand and wrist surgery*
- *Hip replacement surgery*
- *Knee replacement surgery*
- *Shoulder and elbow joint replacement*

Drug leaflets

- *Painkillers*
- *Local steroid injections*
- *Non-steroidal anti-inflammatory drugs*

You can download all of our booklets and leaflets from our website or order them by contacting:

Arthritis Research UK
Copeman House
St Mary's Court
St Mary's Gate
Chesterfield
Derbyshire S41 7TD
Phone: 0300 790 0400
www.arthritisresearchuk.org

Osteoarthritis guidelines

The National Institute for Health and Clinical Excellence (NICE) issued guidelines to GPs in 2008 on how to best treat osteoarthritis based on available evidence.

The NICE guidance on osteoarthritis is available at www.nice.org.uk/CG59.

Printed copies of the NICE osteoarthritis patient guide can be ordered from 0845 003 7783 or at emailpublications@nice.org.uk quoting reference N1460.

Related organisations

The following organisations may be able to provide additional advice and information:

Arthritis Care
Floor 4, Linen Court
10 East Road
London N1 6AD
Phone: 020 7380 6500
Helpline: 0808 800 4050
Email: info@arthritiscare.org.uk
www.arthritiscare.org.uk

Arthritis and Musculoskeletal Alliance (ARMA)
Bride House
18–20 Bride Lane
London EC4Y 8EE
Phone: 020 7842 0910/11
Email: info@arma.uk.net
arma.uk.net

For peer review only

DIAL Network (formerly Disability Information and Advice Line or Dial UK)
Phone: 01302 310 123
www.scope.org.uk/dial
An independent network of local disability information and advice services run by and for disabled people, part of Scope.

Disabled Living Foundation
380–384 Harrow Road
London W9 2HU
Phone: 020 7289 6111
Helpline: 0845 130 9177
Email: helpline@dlf.org.uk
www.dlf.org.uk

General Chiropractic Council
44 Wicklow Street
London WC1X 9HL
Phone: 020 7713 5155
www.gcc-uk.org

General Osteopathic Council
176 Tower Bridge Road
London SE1 3LU
Phone: 020 7357 6655
www.osteopathy.org.uk

Links to sites and resources provided by third parties are provided for your general information only. We have no control over the contents of those sites or resources and we give no warranty about their accuracy or suitability. You should always consult with you GP or other medical professional.

For peer review only - http://bmjopen.bmj.com/site/about/guidelines.xhtml

Notes

For peer review only

For peer review only - http://bmjopen.bmj.com/site/about/guidelines.xhtml

Notes

For peer review only

Notes

For peer review only

For peer review only - http://bmjopen.bmj.com/site/about/guidelines.xhtml

We're here to help

Arthritis Research UK is the charity leading the fight against arthritis.

We're the UK's fourth largest medical research charity and fund scientific and medical research into all types of arthritis and musculoskeletal conditions.

We're working to take the pain away for sufferers with all forms of arthritis and helping people to remain active. We'll do this by funding high-quality research, providing information and campaigning.

Everything we do is underpinned by research.

We publish over 60 information booklets which help people affected by arthritis to understand more about the condition, its treatment, therapies and how to help themselves.

We also produce a range of separate leaflets on many of the drugs used for arthritis and related conditions. We recommend that you read the relevant leaflet for more detailed information about your medication.

Please also let us know if you'd like to receive our quarterly magazine, *Arthritis Today*, which keeps you up to date with current research and education news, highlighting key projects that we're funding and giving insight into the latest treatment and self-help available.

We often feature case studies and have regular columns for questions and answers, as well as readers' hints and tips for managing arthritis.

Tell us what you think

Please send your views to:
feedback@arthritisresearchuk.org
or write to us at:
Arthritis Research UK, Copeman House, St Mary's Court, St Mary's Gate, Chesterfield, Derbyshire S41 7TQ.

A team of people contributed to this booklet. The original text was written by Prof. Mike Doherty, who has expertise in the subject. It was assessed at draft stage by consultant rheumatology nurse Diana Finney and consultant physiotherapist Kay Stevenson. An **Arthritis Research UK** editor revised the text to make it easy to read, and a non-medical panel, including interested societies, checked it for understanding. An **Arthritis Research UK** medical advisor, Prof. Anisur Rahman, is responsible for the content overall.

For peer review only - http://bmjopen.bmj.com/site/about/guidelines.xhtml

Arthritis Research UK

Get involved

You can help to take the pain away from millions of people in the UK by:

- volunteering
- supporting our campaigns
- taking part in a fundraising event
- making a donation
- asking your company to support us
- buying products from our online and high-street shops.

To get more **actively involved,** please call us on **0300 790 0400**, email us at **enquiries@arthritisresearchuk.org** or go to **www.arthritisresearchuk.org**

Arthritis Research UK

Arthritis Research UK
Copeman House
St Mary's Court
St Mary's Gate, Chesterfield
Derbyshire S41 7TD

Tel **0300 790 0400**
calls charged at standard rate

www.arthritisresearchuk.org

Registered Charity No 207711
© Arthritis Research UK 2012
Published November 2012 2025/OA/12-1

Arthritis Research UK

Keeping active with osteoarthritis

It's important to stay active when you have osteoarthritis – exercising will help ease stiffness and stop your muscles becoming weak. As well as the simple exercises in this pull-out, you should choose a form of exercise you enjoy and stick at it.

Swimming, walking, yoga and Pilates are all great options if you have osteoarthritis.

www.arthritisresearchuk.org

For peer review only - http://bmjopen.bmj.com/site/about/guidelines.xhtml

Exercises for osteoarthritis

This handy tear-off section contains exercises that are designed to stretch, strengthen and stabilise the structures that support your joints.

For peer review only - http://bmjopen.bmj.com/site/about/guidelines.xhtml

Exercises for osteoarthritis

The following exercises are designed to stretch, strengthen and stabilise the structures that support your joints.

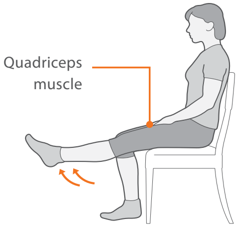

1

Straight-leg raise (sitting): Get into the habit of doing this every time you sit down. Sit well back in the chair with a good posture. Straighten and raise one leg. Hold for a slow count to 10 then slowly lower your leg. Repeat this at least 10 times with each leg. If you can do this easily, try it with light ankle weights and with your toes pointing towards you.

2

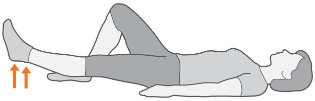

Straight-leg raise (lying): Get into the habit of doing this in the morning and at night while lying in bed. Bend one leg at the knee. Hold your other leg straight and lift your foot just off the bed. Hold for a slow count of five then lower. Repeat five times with each leg every morning and evening.

3

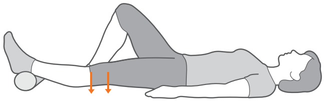

Muscle stretch: Do this at least once a day when lying down. Not only does this exercise help to strengthen the quadriceps muscles, but it also prevents the knee from becoming permanently bent. Place a rolled-up towel under the ankle of the leg to be exercised. Bend the other leg at the knee. Use the muscles of your straight leg to push the back of the knee firmly towards the bed or the floor. Hold for a slow count of five. Repeat at least five times with each leg.

4

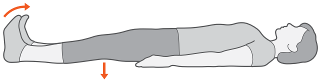

Quad exercise: Pull your toes and ankles towards you, while keeping your leg straight and pushing your knee firmly against the floor. Hold for five seconds and relax. Repeat five times.

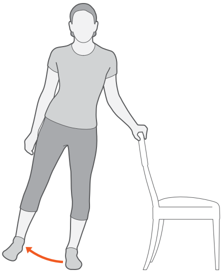

5

Hip abduction: Lift your leg sideways, being careful not to rotate the leg outwards. Hold for five seconds and bring it back slowly, keeping your body straight throughout. Repeat five times on each side. Hold onto a chair or work surface for support.

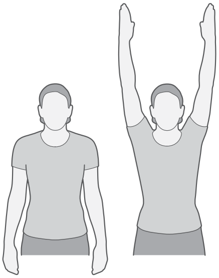

6

Arm stretch (standing): Stand with your arms relaxed at your sides. Raise your arms as far as you can and hold for 5–10 seconds. Lower and repeat five times.

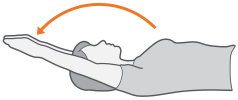

7

Arm stretch (lying): Lie on your back with your arms by your sides. Raise your arms overhead as far as you can and hold for 5–10 seconds. Return your arms to your sides and repeat five times.

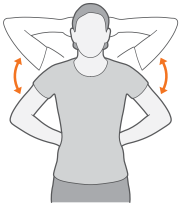

8

Arm lifts: Place your hands behind your head so your elbows are pointing to the sides. Hold for five seconds then place your hands behind your back, keeping your elbows out. Hold for five seconds. Do five sets.

UNIVERSITY OF Southampton

Arthritis Research UK

NHS National Institute for Health Research

Clinical Research Network
Supporting research to make patients, and the NHS, better

Osteoarthritis Thumb Base Therapy Trial

OTTER trial

Thumb Base Pain Exercise Booklet

Information and an exercise programme specifically for people with thumb base pain

OTTER II trial –IRAS 198227

REC ref: 16/SC/0188

For peer review only - http://bmjopen.bmj.com/site/about/guidelines.xhtml

Thumb Base Pain

You have been given this booklet because you have pain or discomfort in your thumb joint.

Pain at the base of your thumb can be due to a number of reasons, one of which can be thumb base Osteoarthritis. Pain at the base of your thumb can affect many aspects of your everyday life. This booklet has been designed with the help of clinicians and patients who have pain at the base of the thumb. It describes a common cause of pain in this area, Osteoarthritis.

This booklet focuses specifically on thumb base pain and provides a programme of exercise devised with the help of national and international therapy clinicians and is supported by published research. You have also been given a Joint Protection Booklet which discusses Osteoarthritis generally, and presents ways to enable you to carry on your normal life as much as possible whilst reducing pain and protecting your joints from further damage.

This booklet contains four main sections:

1. Causes of Thumb Osteoarthritis
2. Symptoms of Thumb Osteoarthritis
3. Treatment of Thumb Osteoarthritis
4. Hand Exercises

For peer review only

For peer review only - http://bmjopen.bmj.com/site/about/guidelines.xhtml

OTTER trial

1. Causes of Thumb Osteoarthritis

Osteoarthritis in the carpometacarpal joint (CMC joint) at the base of the thumb is the most common cause of pain in this area.

The CMC joint is formed where the metacarpal bone of the thumb meets the trapezium bone of the wrist.

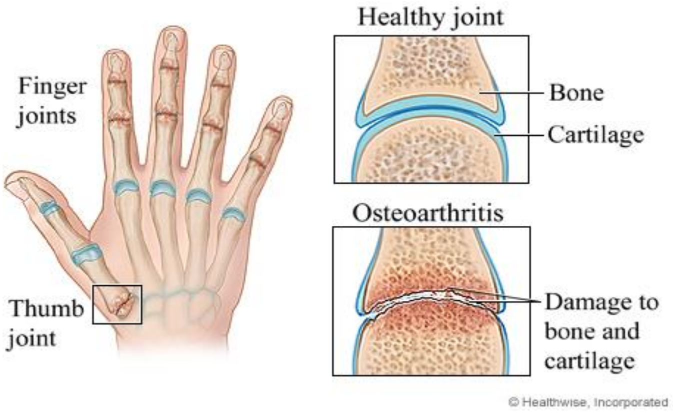

Due to the movement required at the base of the thumb, you rely on your ligaments and bony structures to maintain stability. Damage or overuse can place high loads through the base of the thumb and lead to degeneration. A good point to remember is that any pressures placed through the tip of the thumb during pinching activities are multiplied by around twelve times through the CMC joint.

There are many factors that can lead to pain in the base of the thumb or thumb base Osteoarthritis including a previous injury, repetitive activity of the thumb joint, it can be inherited, and can be affected by gender – women are more likely to have Osteoarthritis than men.

2. Symptoms of Thumb Osteoarthritis

Pain is the primary symptom associated with thumb Osteoarthritis. Initially, pain is present with movement or activity, for example, turning a key, opening a door, lifting a cup. If the Osteoarthritis progresses, pain may be present even during inactivity or rest.

Other symptoms of thumb arthritis include:

- Difficulty gripping objects;
- Swelling, stiffness, or tenderness at the base of the thumb;
- Enlarged appearance and altered posture of the CMC joint;
- Limited range of motion.

For peer review only - http://bmjopen.bmj.com/site/about/guidelines.xhtml

3. Treatment of Thumb Osteoarthritis

Early Osteoarthritis of the thumb can be effectively managed using non-surgical treatment options. These treatments aim to reduce the pain caused by wear and repair of the joint:

- Some medicines can help to reduce inflammation, swelling and pain. You should discuss this with your GP.
- Steroid injections can be given into the joint.
- Self-help measures such as joint protection, diet, complementary therapies etc.

If you have worsening symptoms, you should discuss other options with your GP or consultant.

For peer review only

Additional information can be obtained from the Arthritis Research UK website (http://www.arthritisresearchuk.org/).

We acknowledge the input to this booklet of Sarah Bradley and Kirsty Bancroft of Poole NHS Trust and Prof Ingvild Kjeken from the Norwegian National Advisory Unit on Rehabilitation in Rheumatology.

For peer review only - http://bmjopen.bmj.com/site/about/guidelines.xhtml

4. Hand Exercises

Exercise has many benefits. It can help to ease stiffness, improve movement in your joints and strengthen muscles.

These hand exercises are for you as part of the OTTER II trial. They should benefit you if you have pain at the base of your thumb. These exercises can reduce thumb pain and stiff joints in your hand and strengthen your thumb muscles.

- To achieve the best results **repeat** these exercises at least **3 times a week for at least 20 minutes each time**.
- It is not unusual to experience some slight discomfort or pain in your thumb after doing these exercises. Any discomfort should stop after 24 hours. If you experience discomfort in your thumb or hand when doing these exercises that does not start to feel better after 24 hours, please contact your therapist.
- Always start with the Warm-Up Exercise. To warm up your hand place your hand in a bowl of warm water and gently move your thumb in a circular direction. After one minute you can then change the direction. Carry out these gentle moves for at least 2 minutes. This will help ease your hand and thumb into exercising.
- Follow the Warm-Up Exercise with Level 1 Exercises. When you can easily do Level 1 Exercises then you can go straight from the warm up to Level 2 Exercises. When you can easily do Level 2 Exercises then you can go straight from the warm up to Level 3 Exercises.
- When you do each exercise look carefully at the joints of your thumb. Make sure that your thumb does not bend backwards (or hyperextend) at the joints. Try to make sure that your thumb joints are kept a little bent (or flexed) as you do each exercise, as this will help protect your thumb.

Level 1 Exercises

Exercise 1

Step 1

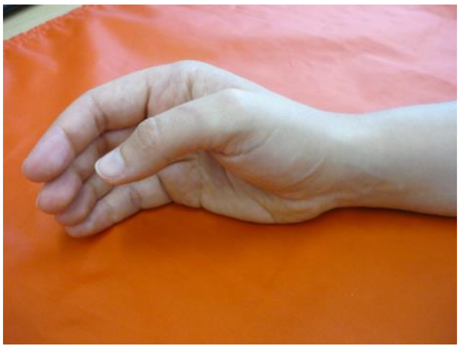

Rest your arm and hand on a table on the little finger side so that your thumb is on top.

For peer review only

Step 2

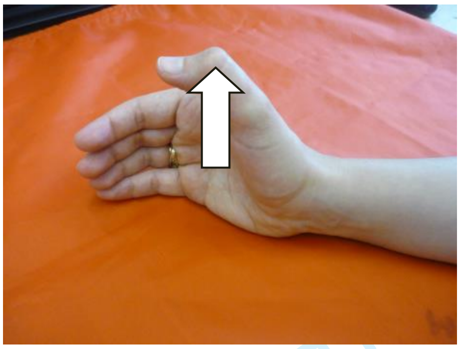

Without help from the other hand lift your thumb upwards as far as possible.

Hold for 10 seconds and repeat up to 10 times.

Make sure that your thumb joints **keep bent** (flexed) when you do this exercise.

Try **not to over extend** any of the thumb joints.

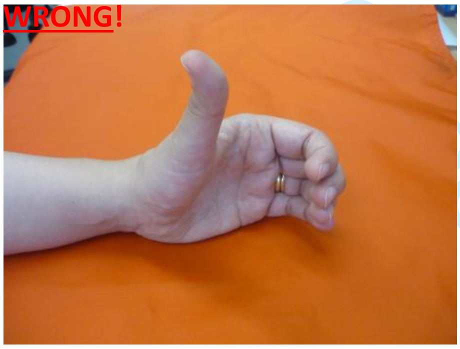

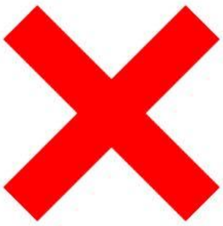

Try **not to over extend** the joints of your thumb. This is **not the right way** to do this exercise.

If your thumb looks like the picture on the left, try the exercise again making sure to **keep your thumb joints bent** (flexed).

Exercise 2

Step 1

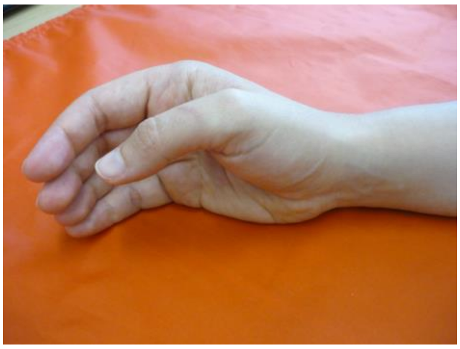

Rest your arm and hand on a table on the little finger side so that your thumb is on top.

Step 2

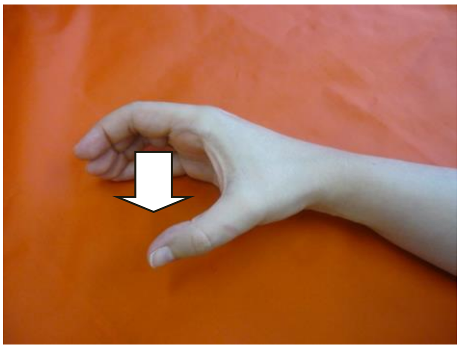

Keeping both joints of the thumb **slightly bent**, keep your thumb as far away from your palm as possible while lifting your thumb up toward the ceiling.

Hold for 10 seconds and repeat up to 10 times.

For peer review only - http://bmjopen.bmj.com/site/about/guidelines.xhtml

WRONG!

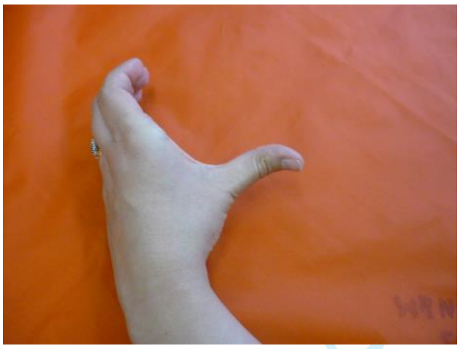

Try **not to bend** the joints of the thumb back.

This is the **wrong way** to do this exercise.

Exercise 3

Place your elbow or back of your hand on a table.

Put your thumb against each fingertip in turn making an ‘O’ shape.

Make sure your thumb joints are **always slightly bent** (flexed).

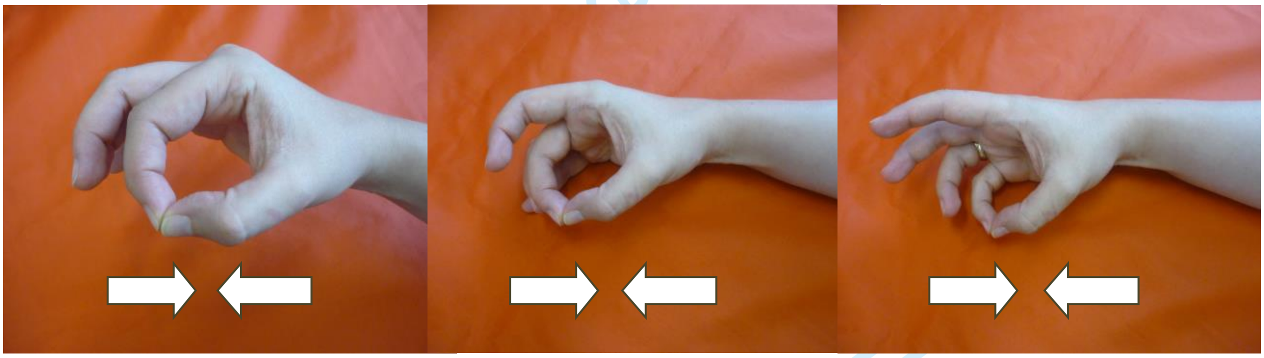

WRONG!

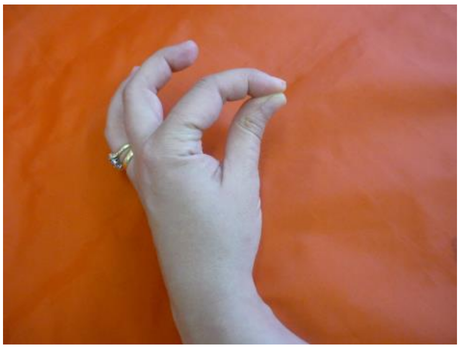

Try **not to bend** the joints of the thumb back.

This is the **wrong way** to do this exercise.

If you can complete Level 1 Exercises easily then move onto Level 2 Exercises. If you cannot complete Level 1 Exercises easily then please do not start Level 2 Exercises

For peer review only - http://bmjopen.bmj.com/site/about/guidelines.xhtml

Level 2 Exercises

Exercise 1

First carry out the **Warm-Up Exercise gently moving your thumb in warm water.**

Perform the rest of the Level 2 Exercises with the strongest rubber band that you can comfortably stretch around your thumb and palm.

Exercise 2

Step 1

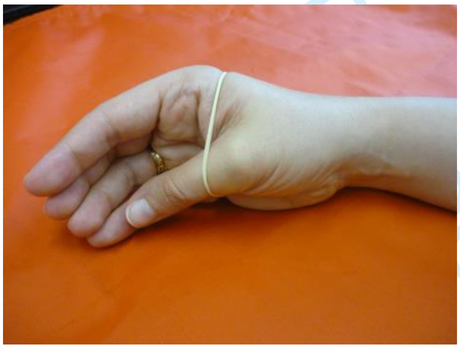

Start with your hand relaxed and a rubber band placed around your hand crossing around the middle of your thumb as in the picture on the left.

Step 2

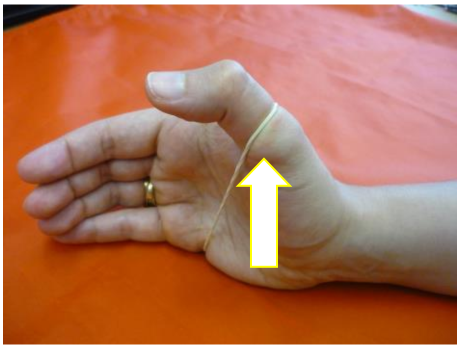

Stretch the band slowly by lifting the thumb upwards as far as possible while keeping the two joints of the thumb slightly bent.

Try to ensure that the wrist **does not bend** forwards during the movement.

Hold for 5 seconds then lower slowly and repeat up to 10 times.

When you can perform this exercise easily, **change** to a stronger band.

WRONG!

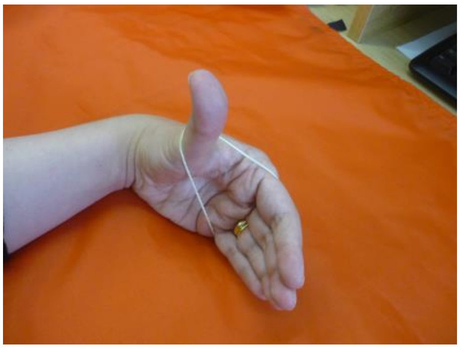

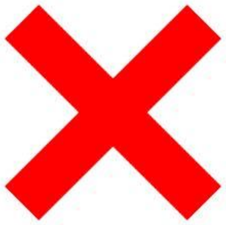

Try **not to bend** the thumb joints back or bring the wrist forward.

This is the **wrong way** to do this exercise.

For peer review only

Exercise 3

Step 1

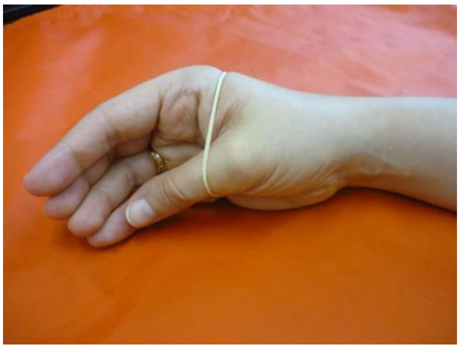

Start with your hand relaxed and a rubber band placed around your hand crossing around the middle of your thumb as in the picture on the left.

Step 2

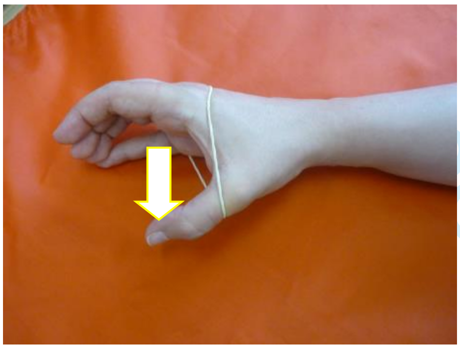

Keep the top **joints of the thumb bent** and lift your thumb up towards your **opposite shoulder** while keeping the thumb as **far away** from the palm as possible.

Step 3

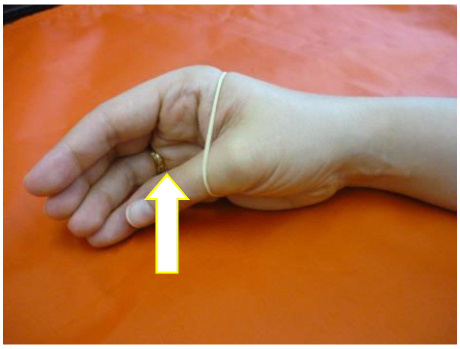

Relax the thumb back to the start position (Step 1) by slowly **lowering the thumb** while keeping both thumb joints a **little bent**.

Try to control this movement and **not let** the band slacken too quickly.

Hold for 5 seconds and repeat up to 10 times.

If you can complete Level 2 Exercises easily then move onto Level 3 Exercises. If you cannot complete these level exercises easily then stop at this level.

For peer review only

For peer review only - http://bmjopen.bmj.com/site/about/guidelines.xhtml

OTTER trial

Level 3 Exercises

Exercise 1

First carry out the **Warm-Up Exercise**.

Exercise 2: Pinch tasks

Practice tasks where you use your thumb to pinch, for example, writing, holding plates, opening clothes pegs, tearing sheets of paper.

When you do these tasks continue to **keep both joints of the thumb slightly bent**, and the **wrist slightly extended**.

Take time to **look at your thumb** and make an effort to **keep your thumb joints in line**. Try **not to let them bend backwards**.

The following pictures show the correct and the wrong way to do Pinch task 1.

Pinch Task 1

Practice writing your name and address using the correct thumb position 5 times

CORRECT!

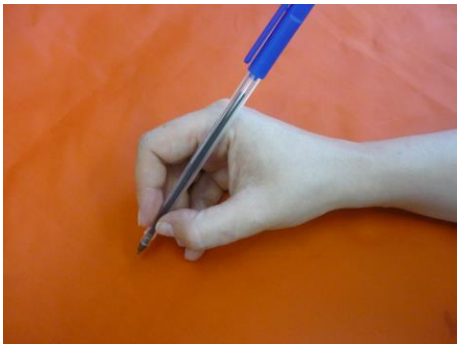

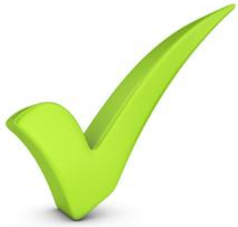

WRONG!

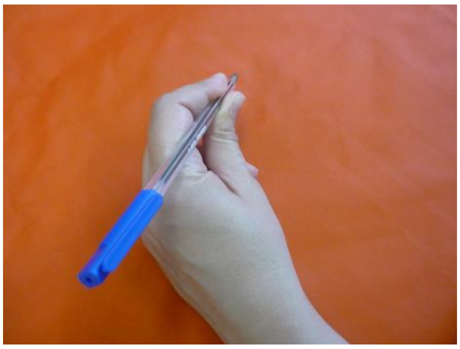

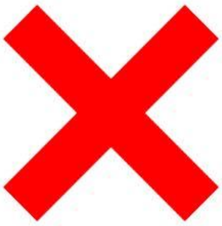

For peer review only - http://bmjopen.bmj.com/site/about/guidelines.xhtml

OTTER trial

The following pictures show the correct and the wrong way to do Pinch Task 2

Pinch Task 2

Practice tearing a piece of paper in half using the correct thumb position 20 times

CORRECT!

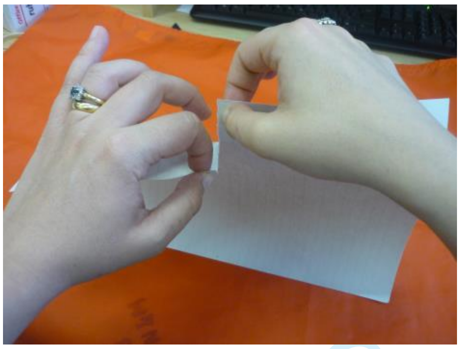

WRONG!

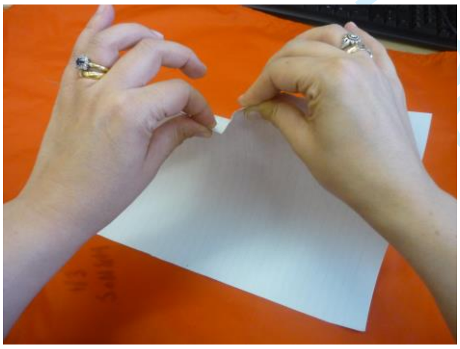

The following pictures show the correct and the wrong way to do Pinch Task 3

Pinch Task 3

Practice holding a clothes peg or a small document clip and squeezing this using the correct thumb position 5 times

CORRECT!

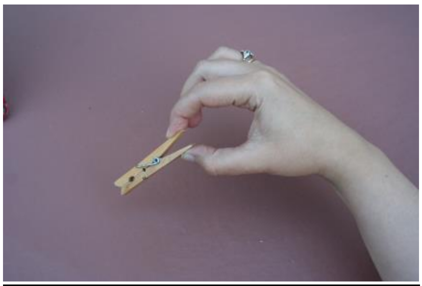

WRONG!

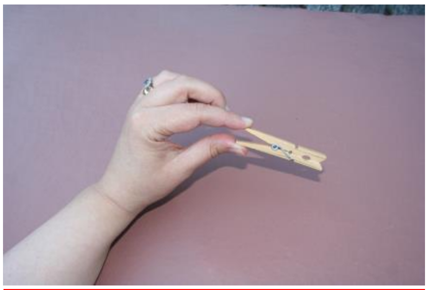

For peer review only - http://bmjopen.bmj.com/site/about/guidelines.xhtml

The following pictures show the correct and the wrong way to do Pinch Task 4

Pinch Task 4

Practice picking up and holding a serving plate using the correct thumb position for 5 times

CORRECT!

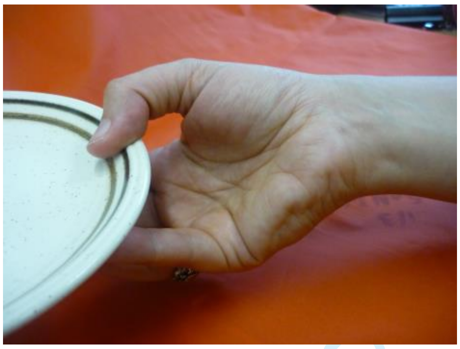

WRONG!

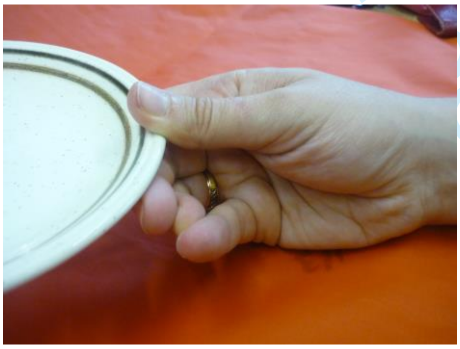

Exercise 3: Grip and turn Tasks

Practice activities which involve turning or twisting, for example, putting nuts on bolts, turning keys in locks, undoing jar tops, turning taps.

During these activities work to maintain a **slight bend (flexion) of both thumb joints** and **avoid the thumb crossing in front of the palm**.

The following pictures show the correct and the wrong way to do Grip and Turn Task 1.

Grip and Turn Task 1

Practice turning a key as if unlocking a door using the correct thumb position 10 times

CORRECT!

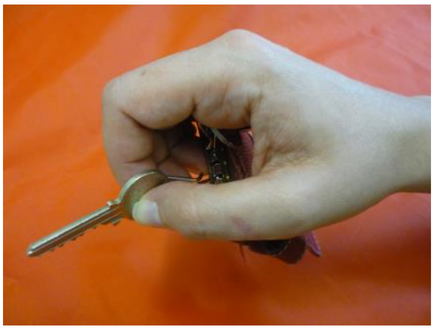

For peer review only - http://bmjopen.bmj.com/site/about/guidelines.xhtml

WRONG!

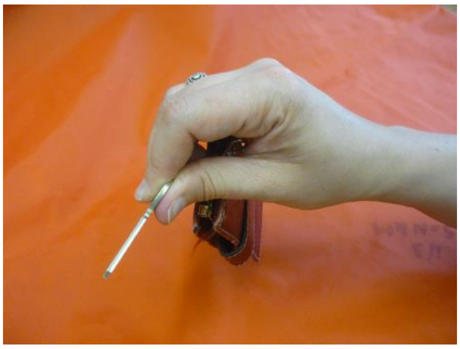

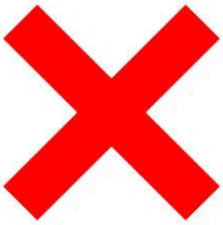

Grip and Turn Task 2

Practice unscrewing and screwing up a bottle top using the correct thumb position five times

CORRECT!

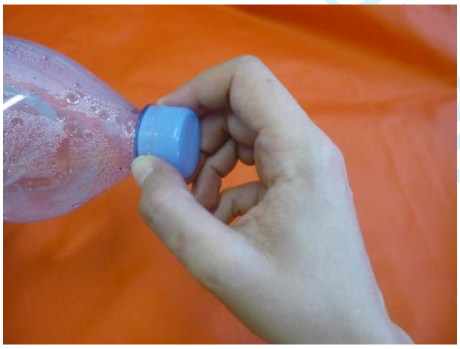

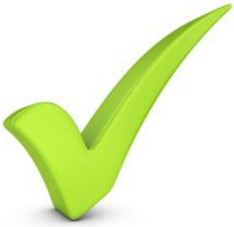

WRONG!

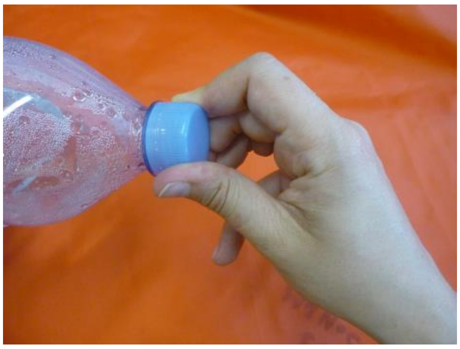

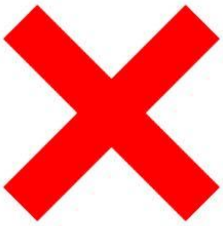

For peer review only

This is the end of the OTTER thumb exercises

If you need to get in touch with the OTTER Trial Research Team, please contact your local occupational therapist or physiotherapist

For peer review only - http://bmjopen.bmj.com/site/about/guidelines.xhtml

STANDARD PROTOCOL ITEMS: RECOMMENDATIONS FOR INTERVENTIONAL TRIALS

The Osteoarthritis Thumb Therapy Trial (OTTER II)

SPIRIT 2013 Checklist: Recommended items to address in a clinical trial protocol and related documents*

Section/item	Item No	Description	Addressed on page number
**Administrative information**			
Title	1	Descriptive title identifying the study design, population, interventions, and, if applicable, trial acronym	1___________
Trial registration	2a	Trial identifier and registry name. If not yet registered, name of intended registry	3___________
	2b	All items from the World Health Organization Trial Registration Data Set	Complete____
Protocol version	3	Date and version identifier	V14.0 dated 23 Aug 2018
Funding	4	Sources and types of financial, material, and other support	23___________
Roles and responsibilities	5a	Names, affiliations, and roles of protocol contributors	1, 22, 23_______
	5b	Name and contact information for the trial sponsor	University of Southampton rgoinfo@soton.ac.uk
	5c	Role of study sponsor and funders, if any, in study design; collection, management, analysis, and interpretation of data; writing of the report; and the decision to submit the report for publication, including whether they will have ultimate authority over any of these activities	None_________

For peer review only - http://bmjopen.bmj.com/site/about/guidelines.xhtml

	5d	Composition, roles, and responsibilities of the coordinating centre, steering committee, endpoint adjudication committee, data management team, and other individuals or groups overseeing the trial, if applicable (see Item 21a for data monitoring committee)	Coordinating centre staff (chief investigator, trial manager, trial administrator, qualitative researcher) will oversee the trial in collaboration with the Oxford trials unit (OCTRU), the co-applicants, the sites, the sponsor, oversight committees (DSMC, TSC and TMG) and regulatory bodies.
**Introduction**			
Background and rationale	6a	Description of research question and justification for undertaking the trial, including summary of relevant studies (published and unpublished) examining benefits and harms for each intervention	4,5__________
	6b	Explanation for choice of comparators	4,5___________
Objectives	7	Specific objectives or hypotheses	5____________
Trial design	8	Description of trial design including type of trial (eg, parallel group, crossover, factorial, single group), allocation ratio, and framework (eg, superiority, equivalence, noninferiority, exploratory)	6____________
**Methods: Participants, interventions, and outcomes**			
Study setting	9	Description of study settings (eg, community clinic, academic hospital) and list of countries where data will be collected. Reference to where list of study sites can be obtained	2, 25, 26____

For peer review only - http://bmjopen.bmj.com/site/about/guidelines.xhtml
For peer review only

Eligibility criteria	10	Inclusion and exclusion criteria for participants. If applicable, eligibility criteria for study centres and individuals who will perform the interventions (eg, surgeons, psychotherapists)	6, 7, ___
Interventions	11a	Interventions for each group with sufficient detail to allow replication, including how and when they will be administered	7, 8, 9________
	11b	Criteria for discontinuing or modifying allocated interventions for a given trial participant (eg, drug dose change in response to harms, participant request, or improving/worsening disease)	9 _______
	11c	Strategies to improve adherence to intervention protocols, and any procedures for monitoring adherence (eg, drug tablet return, laboratory tests)	8,9,15, ___
	11d	Relevant concomitant care and interventions that are permitted or prohibited during the trial	9, 10_________
Outcomes	12	Primary, secondary, and other outcomes, including the specific measurement variable (eg, systolic blood pressure), analysis metric (eg, change from baseline, final value, time to event), method of aggregation (eg, median, proportion), and time point for each outcome. Explanation of the clinical relevance of chosen efficacy and harm outcomes is strongly recommended	2, 3, 5, 12, 13, 14 _____________
Participant timeline	13	Time schedule of enrolment, interventions (including any run-ins and washouts), assessments, and visits for participants. A schematic diagram is highly recommended (see Figure)	12, 13________
Sample size	14	Estimated number of participants needed to achieve study objectives and how it was determined, including clinical and statistical assumptions supporting any sample size calculations	14, 15________
Recruitment	15	Strategies for achieving adequate participant enrolment to reach target sample size	9,15 ___________

Methods: Assignment of interventions (for controlled trials)

Allocation:

Sequence generation	16a	Method of generating the allocation sequence (eg, computer-generated random numbers), and list of any factors for stratification. To reduce predictability of a random sequence, details of any planned restriction (eg, blocking) should be provided in a separate document that is unavailable to those who enrol participants or assign interventions	10__________

Allocation concealment mechanism	16b	Mechanism of implementing the allocation sequence (eg, central telephone; sequentially numbered, opaque, sealed envelopes), describing any steps to conceal the sequence until interventions are assigned	10__________
Implementation	16c	Who will generate the allocation sequence, who will enrol participants, and who will assign participants to interventions	7, 10_________
Blinding (masking)	17a	Who will be blinded after assignment to interventions (eg, trial participants, care providers, outcome assessors, data analysts), and how	6, 9, 10,18_______
	17b	If blinded, circumstances under which unblinding is permissible, and procedure for revealing a participant's allocated intervention during the trial	n/a__________

Methods: Data collection, management, and analysis

Data collection methods	18a	Plans for assessment and collection of outcome, baseline, and other trial data, including any related processes to promote data quality (eg, duplicate measurements, training of assessors) and a description of study instruments (eg, questionnaires, laboratory tests) along with their reliability and validity, if known. Reference to where data collection forms can be found, if not in the protocol	9, 11, 13, 14, 15, 16, 17
	18b	Plans to promote participant retention and complete follow-up, including list of any outcome data to be collected for participants who discontinue or deviate from intervention protocols	16
Data management	19	Plans for data entry, coding, security, and storage, including any related processes to promote data quality (eg, double data entry; range checks for data values). Reference to where details of data management procedures can be found, if not in the protocol	16, 17_________
Statistical methods	20a	Statistical methods for analysing primary and secondary outcomes. Reference to where other details of the statistical analysis plan can be found, if not in the protocol	17, 18, 19, 20
	20b	Methods for any additional analyses (eg, subgroup and adjusted analyses)	18__________
	20c	Definition of analysis population relating to protocol non-adherence (eg, as randomised analysis), and any statistical methods to handle missing data (eg, multiple imputation)	17, 19

Methods: Monitoring

Data monitoring	21a	Composition of data monitoring committee (DMC); summary of its role and reporting structure; statement of whether it is independent from the sponsor and competing interests; and reference to where further details about its charter can be found, if not in the protocol. Alternatively, an explanation of why a DMC is not needed	20, 26. The DSMC in independent from the sponsor and all members will provide competing interest statements.
	21b	Description of any interim analyses and stopping guidelines, including who will have access to these interim results and make the final decision to terminate the trial	16____________
Harms	22	Plans for collecting, assessing, reporting, and managing solicited and spontaneously reported adverse events and other unintended effects of trial interventions or trial conduct	20___________
Auditing	23	Frequency and procedures for auditing trial conduct, if any, and whether the process will be independent from investigators and the sponsor	Subject to audit by national bodies, the trials unit and the sponsor
**Ethics and dissemination**			
Research ethics approval	24	Plans for seeking research ethics committee/institutional review board (REC/IRB) approval	3_____________
Protocol amendments	25	Plans for communicating important protocol modifications (eg, changes to eligibility criteria, outcomes, analyses) to relevant parties (eg, investigators, REC/IRBs, trial participants, trial registries, journals, regulators)	Once approved by sponsor/REC/HRA as appropriate, important modifications will be communicated to site research teams and R&D offices by email.

For peer review only - http://bmjopen.bmj.com/site/about/guidelines.xhtml

Consent or assent	26a	Who will obtain informed consent or assent from potential trial participants or authorised surrogates, and how (see Item 32)	Consent will be obtained by those delegated the task (i.e an NHS therapist trained in OTTER II trial procedures)by the local PI at the site. 11
	26b	Additional consent provisions for collection and use of participant data and biological specimens in ancillary studies, if applicable	11___________
Confidentiality	27	How personal information about potential and enrolled participants will be collected, shared, and maintained in order to protect confidentiality before, during, and after the trial	16, 17________
Declaration of interests	28	Financial and other competing interests for principal investigators for the overall trial and each study site	23___________
Access to data	29	Statement of who will have access to the final trial dataset, and disclosure of contractual agreements that limit such access for investigators	17____________
Ancillary and post-trial care	30	Provisions, if any, for ancillary and post-trial care, and for compensation to those who suffer harm from trial participation	11___________
Dissemination policy	31a	Plans for investigators and sponsor to communicate trial results to participants, healthcare professionals, the public, and other relevant groups (eg, via publication, reporting in results databases, or other data sharing arrangements), including any publication restrictions	21___________
	31b	Authorship eligibility guidelines and any intended use of professional writers	Described in trial Publication Plan
	31c	Plans, if any, for granting public access to the full protocol, participant-level dataset, and statistical code	n/a____________ _

Appendices

For peer review only - http://bmjopen.bmj.com/site/about/guidelines.xhtml

Informed consent materials	32	Model consent form and other related documentation given to participants and authorised surrogates	Not included in this submission – available on request _______________
Biological specimens	33	Plans for collection, laboratory evaluation, and storage of biological specimens for genetic or molecular analysis in the current trial and for future use in ancillary studies, if applicable	n/a_____________ _

*It is strongly recommended that this checklist be read in conjunction with the SPIRIT 2013 Explanation & Elaboration for important clarification on the items. Amendments to the protocol should be tracked and dated. The SPIRIT checklist is copyrighted by the SPIRIT Group under the Creative Commons "Attribution-NonCommercial-NoDerivs 3.0 Unported" license.

For peer review only

BMJ Open

The Osteoarthritis Thumb Therapy Trial (OTTER II): A study protocol for a three arm multi centre randomised placebo controlled trial of the clinical effectiveness and efficacy and cost effectiveness of splints for symptomatic thumb base osteoarthritis

Journal:	*BMJ Open*
Manuscript ID	bmjopen-2018-028342.R2
Article Type:	Protocol
Date Submitted by the Author:	12-Jul-2019

Complete List of Authors:	Adams, Jo; University of Southampton, Faculty of Environment and Life Sciences<br>Barratt, Paula ; University of Southampton, Faculty of Environment and Life Sciences<br>Arden, Nigel; Nuffield Department of Orthopaedics, ;<br>Barbosa Bouças, Sofia; Brunel University , Department of Life Sciences<br>Bradley, Sarah; Poole Hospital NHS Foundation Trust<br>Doherty, Michael ; University of Nottingham<br>Dutton, Susan; University of Oxford, CSM<br>Dziedzic, Krysia; Arthritis Research Campaign National Primary Care Centre<br>Gooberman-Hill, Rachael; University of Bristol, Bristol Medical School<br>Hislop Lennie, Kelly; University of Southampton, Faculty of Environment and Life Sciences<br>Hutt Greenyer , Corinne; University of Southampton, Faculty of Environment and Life Sciences<br>Jansen , Victoria; Royal Derby Hospital, Pulvertaft Hand Centre<br>Luengo-Fernandez, Ramon; University of Oxford, Health Economics Research Centre<br>Meagher, Claire ; University of Southampton, Faculty of Environment and Life Sciences<br>White, Peter; University of Southampton, Faculty of Environment and Life Sciences<br>Williams, Mark; Oxford Brookes University, Sport, Health Sciences and Social Work

**Primary Subject Heading**:	Rehabilitation medicine
Secondary Subject Heading:	Rheumatology
Keywords:	Osteoarthritis, REHABILITATION MEDICINE, RHEUMATOLOGY, Efficacy trial

The Osteoarthritis Thumb Therapy Trial (OTTER II): A study protocol for a three arm multi centre randomised placebo controlled trial of the clinical effectiveness and efficacy and cost effectiveness of splints for symptomatic thumb base osteoarthritis

Corresponding author: Jo Adams. School of Health Sciences, Faculty of the Environement and Life Sciecnes, Building 67, University of Southampton, Highfield Campus, University Road, Southampton, SO17 1BJ, UK. Tel: + 44 (0)23 8059 5287. E-mail: ja@soton.ac.uk

Authors:

Jo Adams School of Health Sciences, University of Southampton, Southampton, UK

Paula Barratt School of Health Sciences, University of Southampton, Southampton, UK

Nigel Arden Nuffield Department of Orthopaedics, Rheumatology and Musculoskeletal Sciences (NDORMS), University of Oxford, Oxford, UK

Sofia Barbosa Bouças Dept of Life Sciences, Brunel University London, Uxbridge, UK

Sarah Bradley Hand Therapy Unit, Poole Hospital NHS Trust, Poole, UK

Michael Doherty School of Medicine, University of Nottingham, Nottingham, UK

Susan Dutton Oxford Clinical Trials Research Unit, University of Oxford, Oxford, UK

Krysia Dziedzic Research Institute for Primary Care and Health Sciences, Keele University, Keele, UK

Rachael Gooberman-Hill Elizabeth Blackwell Institute for Health Research, University of Bristol, Bristol, UK

Kelly Hislop School of Health Sciences, University of Southampton, Southampton, UK

Corinne Hutt Greenyer School of Health Sciences, University of Southampton, Southampton, UK

Victoria Jansen Pulvertaft Hand Centre, Royal Derby Hospital, Derby, UK

Ramon Luengo Fernandez Nuffield Dept of Population Health, University of Oxford, Oxford, UK

Claire Meagher School of Health Sciences, University of Southampton, Southampton, UK

Peter White School of Health Sciences, University of Southampton, Southampton, UK

Mark Williams Dept of Sport, Health Sciences and Social Work, Oxford Brookes University, Oxford, UK

On behalf of the OTTER II collaborative group

Word count: 4467 (excl abstract and tables)

For peer review only - http://bmjopen.bmj.com/site/about/guidelines.xhtml

Abstract

Introduction

The economic cost of osteoarthritis (OA) is high. At least 4.4 million people have hand OA in the UK. Symptomatic thumb base OA affects 20% of people over 55 years, causing more pain, work and functional disability than OA elsewhere in the hand. Most evidence-based guidelines recommend splinting for hand OA. Splints that support or immobilise the thumb base are routinely used despite there being limited evidence on their effectivenss. The potential effects of placebo interventions in OA are acknowledged, but few studies investigate the clinical efficacy of rehabilitation interventions nor the impact of any placebo effects associated with splints.

Methods and Analysis

Participants aged 30 years and over with symptomatic thumb base OA will be recruited into the trial from secondary care occupational therapy and physiotherapy centres. Following informed consent, participants will complete a baseline questionnaire and then be randomised into one of three treatment arms: a self-management programme, a self-management programme plus a verum thumb splint, or a self-management programme plus a placebo thumb splint. The primary outcome is the AUSCAN hand pain scale. The study end point is 8 weeks after baseline. Baseline assessments will be carried out prior to randomisation and outcomes collected at 4; 8 and 12 weeks. Cost effectiveness analysis will be conducted and individual qualitative interviews conducted with up to 40 participants after 8 weeks to explore perceptions and outcome expectations of verum and placebo splints and exercise.

Ethics and Dissemination

South Central - Oxford C Research Ethics Committee approved this study (16/SC/0188). The findings will be disseminated to health professional conferences, journals and lay publications for patient organisations. The research will contribute to improving the management of thumb base OA and help clinicians and patients make informed decisions about the value of different interventions.

Trial Registration Number: ISRCTN 54744256

For peer review only - http://bmjopen.bmj.com/site/about/guidelines.xhtml

Key words: Osteoarthritis; thumb base; splint; hand pain; hand function; placebo; self-management.

Strengths and Limitations

1. This trial is powered to evaluate the clinical benefit and statistical significance of adding splinting to a self-management programme for people with thumb base OA.

2. This is the first trial to use a placebo thumb splint intervention and will add to the understanding of contextual aspects of such visible physical treatment and self-management programmes for OA.

3. The trial has been informed and designed with the input of patients and expert clinicians and the trial outcome measures have been agreed as meaningful by patients.

4. The 12 week trial follow-up period is limited and longer term follow-up would provide further useful outcome data.

For peer review only - http://bmjopen.bmj.com/site/about/guidelines.xhtml
For peer review only

INTRODUCTION

Osteoarthritis (OA) is a leading cause of pain, disability, healthcare utilisation, and productivity loss in the UK. Each year approximately two million adults visit their GP with symptoms of OA [1]. Osteoarthritis is more prevalent in women, the incidence increases with age and recent estimates suggest more than 150 million Europeans have radiographic hand OA and 15 million have symptomatic arthritis [2]. Symptomatic thumb base (1st carpometacarpal and/or scaphotrapezial joint) OA affects approximately 22% of people aged 50 years and over [3].Thumb base OA is likely to become more prevalent in the future since the incidence increases with age [4] and is identified as a priority for treatment as it causes more pain and disability and is associated with a worse prognosis than other hand sites affected by OA [5].

Patients with thumb base OA present with predominantly mechanical usage-related pain over the thumb base [6] and are more likely to have more pain, work disability and reduction in quality of life and function, and to receive more anti-inflammatory drugs and more splinting than participants with OA affecting other hand joints[5] [7] [8] [9]

Thumb base OA affects entire hand function [10] and the overall impact, particularly in older people, can be substantial, with many experiencing difficulties with daily household, caring, work and leisure activities [11]. Despite the scale of this problem, it appears that both patients and practitioners often believe that there is little that can be done [12].

Therapeutic splinting for thumb base OA aims to minimise or eliminate motion at the thumb carpometacarpal joint [13] in order to prevent joint deterioration and/or deformity, decrease pain, and increase overall hand function [14]. The process of designing thumb splints currently lacks detailed reporting but biomechanical principles have been applied to one design to off load the dynamic forces occurring during functional hand use on a symptomatic carpometacarpal joint [15]. It is known that stabilisation of the carpometacarpal joint to reduce pain levels impacts on hand functionality [16] Currently, thumb splints are recommended for patients with thumb base OA [17]. However, the efficacy of splinting based on this approach has not yet been established and forms the rationale for this trial. Some evidence based on small, non-powered samples [18 19] show thumb

For peer review only - http://bmjopen.bmj.com/site/about/guidelines.xhtml

splints can help relieve pain however, evidence to support their effectiveness is not yet fully supported by robust research [20]. There is insufficient evidence to suggest that the combination of splinting delivered alongside hand exercises is more effective than hand exercises alone [21] [22] [23] and the evidence to support splinting in alleviating hand pain in the medium term is supported by low level evidence only [24]. To date, there has also been no examination of any contextual and non-specific patient-practitioner interaction effects associated with splinting in thumb base OA. If this is substantial, optimising the non-specific effects of treatment could improve treatment effectiveness and overall care of people with OA in a safe and cost effective way [25 26]. This randomised controlled trial (RCT) protocol, was designed in accordance with the SPIRIT checklist [27], to evaluate the effectiveness, efficacy and cost effectiveness of thumb splints for people with thumb base OA.

The primary objective of this trial is to determine the clinical effectiveness and efficacy of thumb splints when added to a self-management programme for people with symptomatic thumb base OA. This trial questions:

1. Is there a benefit of adding a thumb base splint to a self-management programme for people with thumb base OA?

2. Is there a difference in benefit between adding a verum or a placebo thumb base splint to a self-management programme for people with thumb base OA?

3. What are patients' views and experiences of the effectiveness, acceptability and adherence to the trial interventions?

METHODS AND ANALYSIS

Patient and Public Involvement

Exploring the effectiveness of orthotic devices for use by patients living with osteoarthritis was identified as a priority area for investigation by an Arthritis Research UK patient stakeholder committee. Two patient and public group meetings were carried out with 8 patient partners living with hand OA to listen to patient experiences of living with hand OA, explore intervention components of the trial and help to inform co-design a placebo splint design [28]. Patient partners discussed with the team what splints they thought should be included in a trial, which splint designs

For peer review only - http://bmjopen.bmj.com/site/about/guidelines.xhtml

that found most credible and which outcomes were important to them. These discussions informed the study design. A national Delphi study with therapy clinicians and patients was conducted, to inform and agree trial processes [29 30]. Clinicians tended to want more focused time efficient measures where patients usually hoped for more comprehensive intervention that was beyond the time allocated to NHS out-patient provision. This helped to consider treatment burden from both clinical and patient's view points. This involvement at an early stage ensured that clinicians felt that they had contributed and had ownership in the trial design and patient input ensured that patients' perspectives had been integral to the study design hopefully making the trial relevant and appealing to prospective patients. We conducted a focus group to explore the support required by NHS therapy clinicians when knowingly delivering placebo splints was completed [31]. A pilot study across five NHS recruitment sites to test recruitment and procedural feasibility and safety and the convincing delivery of a newly designed placebo splint was conducted and reported [32]. The education and support needs identified by therapy clinicians taking part in a placebo controlled RCT was sought and recorded [33]. This informed the trial training for clinicians. Our patient partner and co-author (CHG) provided a patient's perspective in developing recruitment strategies, study conduct and lay dissemination routes.

Trial Design and Setting

OTTER II is a pragmatic, multi-centred, single (participant) blind, superiority randomised controlled clinical effectiveness and efficacy trial. People with symptomatic thumb base OA reporting moderate to severe thumb base pain, will be equally allocated to one of three groups: Group A: Eight weeks of a facilitated self-management programme, Group B: Eight weeks of a facilitated self-management programme plus a verum splint, or Group C: Eight weeks of a facilitated self-management programme plus a placebo splint. All groups will be encouraged to continue their self management and, where appropriate, splint wear until follow up at 12 weeks from baseline. The study will run from 28th February 2017 to 14th March 2019

Participants will be recruited consecutively from referrals to Occupational Therapy or Physiotherapy (Therapy) departments from new, current and review patients of Rheumatology, Orthopaedic, Hand Surgery Units and General Practice at 17 NHS recruitment sites (Appendix 1). Intervention will be

For peer review only - http://bmjopen.bmj.com/site/about/guidelines.xhtml

delivered by a qualified OTTER II trained occupational therapist or physiotherapist who has worked independently in a clinical role treating patients with hand OA and who works within a therapy department that accepts referrals for thumb base OA patients.

Participants

Participant inclusion criteria were decided upon iteratively with our rheumatologists, clinicians and hand surgeons in relation to the literature about prevalence, incidence rates and current treatment by therapy departments in the UK. Clinical tests, that contributed to inclusion criteria, were selected following: review of literature; consideration of the sensitivity and specificity of each test; examination of the practicality and feasibility of the testing procedure for the collaborating clinicians working across different UK hospitals. As there is no clear consensus on the longevity of the impact of intra articular steroid injections into the 1st CMCJ [34] [35] [36] we used local clinical NHS protocols relating to the length of time suggested to repeat 1st CMCJ steroid injections, to inform our inclusion criteria.

Consecutive potential participants will be screened and assessed for recruitment into the trial by the collaborating OTTER II trial trained therapy clinicians using inclusion and exclusion criteria as shown in Table 1. The charactieristics of participants who fulfil inclusion criteria and who decline to take part will be recorded.

**Inclusion Criteria**	
1	Aged 30 years and over.
2	At least moderate hand pain (AUSCAN [37] hand pain score >5) and moderate functional hand disability (AUSCAN [37]hand functional disability score >9).
3	Show signs and symptoms of thumb base OA on clinical enquiry and examination, specifically: hard tissue enlargement of the first carpometacarpal joint (CMCJ) OR squaring of the thumb base OR pain that worsens when pinching OR pain that worsens on span grip (e.g. opening a jar) OR crepitus on movement OR reduction in thumb base range of movement OR positive thumb adduction provocation test [38] OR positive thumb extension provocation test [38] OR pain on palpation of the dorso-radial aspect of the thumb CMCJ.
4	No other household member participating in the trial.

For peer review only - http://bmjopen.bmj.com/site/about/guidelines.xhtml

5	Able to give written informed consent.
6	Available to attend Occupational Therapy/Physiotherapy/Hand Therapy sessions.
**Exclusion Criteria**	
**1**	Consultation with therapy department or treatment for this thumb problem (excluding pain killers and anti-inflammatories) in the previous six months.
**2**	Intra-articular joint injection to wrist, fingers or thumb in the previous two months.
**3**	Fractures or significant injury or surgery to the wrist or hand within the previous six months.
**4**	Red flags i.e. history of serious illness or disease. i.e. any other diagnosed rheumatic conditions: gout, psoriatic arthritis, ankylosing spondylitis, connective tissue disorders (systemic lupus, systemic sclerosis), resulting in inflammatory arthritis in the hand/s, or, progressive neurological signs, or acute swollen hand joint.
**5**	Diagnosis of dementia or other significant disorder likely to affect communication.
**6**	Already received thumb splints for thumb base OA.
**7**	Skin disease that may interfere or contraindicate splint wear.
**8**	Participant of a drug or medical device trial in the last 12 weeks.

Table 1. OTTER II Trial patient inclusion and exclusion criteria

Interventions

The trial's three intervention arms all include 90 minutes of direct therapy intervention delivered by a qualified OTTER II trained occupational therapist or physiotherapist who has worked independently in a clinical role treating patients with hand OA and who works within a therapy department that accepts referrals for thumb base OA patients.

The direct therapy intervention will be delivered in a 60- minute baseline appointment at an NHS secondary care hospital or clinic, when the interventions listed below will be delivered. A 30-minute follow up intervention at week four will be conducted, where progress is reviewed and any necessary adjustments made. A final third hospital visit at week eight is for finalisation of trial procedures only and includes no direct therapy intervention.

For peer review only - http://bmjopen.bmj.com/site/about/guidelines.xhtml

The three intervention arms, delivered at the baseline appointment are:

Group A - An optimal self-management programme

The self-management programme (Appendix 2) includes:

1. Teaching standardised hand exercises (developed from an evidence based review [39]and provision of a trial-specific hand exercise booklet.

2. Provision of a trial-specific booklet about joint protection, activity pacing and general advice about OA followed by a discussion with the therapist of the content.

3. Provision of the Arthritis Research UK Osteoarthritis information booklet.

4. A discussion with the therapist of the facilitators and barriers to engaging with self-management principles.

5. A patient hand exercise diary.

Group B - Optimal self-management programme plus a verum thumb base splint

Group B participants receive:

1. The optimal self-management programme as detailed above in Group A.
2. A verum splint. Therapists and participants will be given an option of two different splints, with the choice guided by a standardised Splint Decision Protocol (Appendix 3).

The splinting options informed by the study's design and development stage will be:

- Procool Thumb CMC Restriction splint (black) (Ref PTRS).

Or

- Orfilight 2.5mm 3/32" micro perforated (beige) trouser leg splint (custom made by the therapist from a pre-cut standardised trial template and standardised strapping protocol).

Both splints will be delivered with instructions on how to wear and use the splint (Appendix 4). The Procool thumb CMC restriction splint comes in packaging with a label providing details of the manufacturer and washing instructions.

3. A discussion with the therapist of the facilitators and barriers to engaging with splint wear (Appendix 5).
4. A patient splint wear diary (Appendix 6).

For peer review only - http://bmjopen.bmj.com/site/about/guidelines.xhtml

Group C - Optimal self-management programme plus a placebo thumb base splint

Group C participants receive:

1. The self-management programme as detailed above in Group A.
2. A placebo splint. There will be the choice of two designs of placebo splint. These are made in a lightweight nylon, secured around the wrist and have phalangeal components but no basal thumb joint support. One is black and the other is beige to match the verum splint options and three sizes will be manfucatured. They have been designed with no known active component and none was detectable during testing [40]. A standardised Splint Decision Protocol about which splint will be most appropriate to issue in which situation will be used (Appendix 7). The placebo splints arrive in packaging with a label providing details of the manufacturer, washing instructions, and a lifestyle education leaflet about how to position and wear the splint. Both splints will be delivered with instructions on how to wear and use the splint.
3. An information sheet to outline when the splint should be worn (Appendix 4)
4. A discussion with the therapist of the facilitators and barriers to engaging with splint wear. (Appendix 5).
5. A patient splint wear diary (Appendix 6)

Trained OTTER II clinicians will deliver a standardised self management package of care using their clinical judgement to apply the content for each individual patient. Participants in group A will receive more time spent on self management than participants in group B and C.

Concomitant care

Any relevant contralateral thumb treatment for participants will be delayed until the end of the trial. There will be no alteration in participants' general concomitant care whilst on the trial, . Additional treatments e.g. joint injection, surgery and reported purchase or use of own splints during the study period will be captured on self-report questionnaires. Criteria for modifying allocated interventions is detailed within the OTTER II Trial Safety Information.

For peer review only - http://bmjopen.bmj.com/site/about/guidelines.xhtml

Investigator Training

Trial training visits will be conducted by the OTTER II trial management team (JA, PB, PW) to carry out standardised instruction and demonstration for OTTER II research clinicians prior to the start of patient recruitment. Training will cover procedures for maintaining clinical assessor and participant blinding, delivery of eligibility tests, interventions, , reporting of safety events, data entry and use of Case Report Forms (CRFs). Sites will also be visited by the trial team (JA, MW, CM) to provide support and guidance on maximising participant recruitment through liaison with surgery and community health teams, and to conduct quality assurance evaluations of intervention delivery as required.

A page of the trial website which is only available to site staff (not participants or the public) will provide training videos on the standardised delivery of all the trial interventions. All clinicians will complete the National Institute of Health Research Good Clinical Practice (GCP) training inorder to have the skills and knowledge necessary to comply with the international ethical, scientific and practical standards to which all clinical research is conducted. Compliance with GCP provides public assurance that the rights, safety and wellbeing of research participants are protected and that research data are reliable. [41].

Participant identification and baseline assessment

Potential participants will receive a letter about the study and a Participant Information Sheet at the hospital clinic or by post. For interested patients an out-patient therapy department appointment will be made at Week 0 (Baseline). Following eligibility screening, formal written consent will be obtained and participants will complete the baseline assessments. Consent will be obtained by an NHS therapist trained in OTTER II Trial procedures.

Randomisation

Eligible patients will be enrolled into the study via the Oxford Clinical Trials Research Unit (OCTRU) online randomisation service and this system will record eligibility and stratification data.

When participants are randomised to a treatment arm this constitutes the date of start of treatment. Participants will only be randomised once and for the treatment of one thumb. For participants with symptoms of bilateral thumb base OA the most painful thumb will be treated first within the trial. If

For peer review only - http://bmjopen.bmj.com/site/about/guidelines.xhtml

at the end of the trial participants require treatment on a contralateral thumb then participants will be invited back by the clinical service to undergo routine (i.e. non-trial) clinical intervention at the end of the trial.

Randomisation will be on a 1:1:1 basis. The first 30 participants will be randomised using a simple random list with varying block sizes to seed the subsequent minimisation algorithm. This random list is generated by the trial statistician and concealed from all other members of the trial team to ensure future treatment allocations cannot be predicted. Subsequent participants will be randomised using a validated computer randomisation program (RRAMP) with a minimisation algorithm, which will ensure balanced treatment allocations across the stratification factors (centre, baseline AUSCAN hand pain score (Category 1= AUSCAN pain score 6 to 12 and Category 2= AUSCAN hand pain score 13 to 20) and treated hand dominance). The minimisation system includes a random element (0.8) to minimise predictability of treatment allocations for new participants.

Participants will be blind to treatment allocation. Strategies to maximise participant blinding include, training for trial clinicians in how to present and discuss the treatment arms positively without disclosing other treatment options and routine assessment included in the quality assurance trial monitoring visits of clinician's communication and interaction in presenting treatment options with participants. The Participant Information Sheet (PIS) has been carefully worded to state that an element of the self- management intervention may be placebo but does not state what aspect this may be. The study will be described as a comparison of self-management interventions and not divulging detail about placebo splinting in any trial of clinician communication. Trial participants will not receive group intervention and therefore cannot compare their interventions received and we will not include participants who have already received thumb base splints. All of these strategies will help to maintain participant blinding. Contamination between treatment arms will be limited by identifying on hospital notes that the patient is part of the OTTER II Trial and therefore should not receive any further hand therapy treatment from the hospital team. It is not possible to prevent participants allocated to Arm A or B purchasing their own splints during the trial period, however, the data will be captured in the final study questionnaire and will be used to assess any degree of contamination.The OTTER II placebo splints will not be able to purchased privately.

For peer review only - http://bmjopen.bmj.com/site/about/guidelines.xhtml

This approach will support participant blinding, in addition to the training that treating therapists will receive about delivering placebos and responding to participant questions. The success of participant blinding will be assessed via patient questionnaires at 12 weeks.

Treatment and follow up

Figure 1 flowchart shows the participants' progress through the trial. After randomisation participants will receive their allocated intervention during a 60 minute appointment (week 0). All participants in each treatment arm will receive a 10 minute telephone call at two weeks from the therapist to check for adherence to the self-management programme and to discuss any problems identified by the participant as relevant. In addition for Group B and C participants the therapist will check splint wear and comfort. Participants will attend for a 30 minutes appointment 4 weeks after randomisation, to review treatments and make adjustments where necessary and an assessor blind to treatment allocation will administer the objective Grip Ability Test [42] (final hospital visit occurs 8 weeks after randomisation. A paper questionnaire will be posted to each participant at 12 weeks to collect follow-up patient reported outcomes. After the 12 week follow-up has been completed (study end point) all patients will be offered the option of a further follow up appointment for any further care that is needed. If patients have received verum splints then they may keep these, if patients have received placebo splints or self management alone then they will be offered the opportunity to receive a verum splint if required.

Qualitative Interviews

Between weeks 8 and 12 a qualitative telephone interview will take place for a subgroup of patients in intervention arm B and C. The researcher will be blinded for the qualitative interviews in order to prevent bias related to treatment allocation during data collection. In order to make this practical, the researcher carrying out data collection will be independent from the RCT and will be unfamiliar with the splints and differences between them. The methodological approach for the qualitative study will be a framework analysis. Purposive sampling will be used and based on male to female

For peer review only - http://bmjopen.bmj.com/site/about/guidelines.xhtml

ratio (as per the study sample); age and AUSCAN hand pain index at baseline. Interviews will be conducted with up to 40 participants. Interviews will be semi-structured, using a topic guide informed by patient partners, to elicit experience of splint and self-management interventions, splint preference, adherence and views about reasons for effectiveness. There will be a password for each participant that has agreed to take part in the interview study so that the participant can be correctly identifed on the telephone. Consent for participation in the qualitative interview will be sought and given at the time of entry into the main study.

For peer review only

The schedule of enrolment, interventions and a summary of assessments are shown in **Table 2**. Detailed information about baseline assessments and outcome measures are listed in **Table 3.**

For peer review only - http://bmjopen.bmj.com/site/about/guidelines.xhtml

	STUDY PERIOD							
	Enrolment	Allocation	Post-allocation					Close-out
**TIMEPOINT:**	**Baseline Day 0**	**Baseline Day 0**	**2 wks**	**4 wks**	**8 wks**	**9-11 wks**	**12 wks**	**After 12 wks**
**ENROLMENT:**								
**Eligibility screen***	X							
**Informed consent***	X							
**Randomisation/ Allocation**		X						
**INTERVENTIONS:**								
**Optimal NHS self-management care**		X						
**Optimal NHS self-management care plus verum splint**		X						
**Optimal NHS self-management care plus placebo splint**		X						
**Telephone call check of progress**			X					
**Review of self-management care and splint wear (if applicable)**				X				
**ASSESSMENTS:**								
**Baseline Assessments: See Table 3**	X							
**Outcomes: Grip Ability Test [42]**				X	X			
**Outcomes: See Table 3**					X			
**Outcomes at follow up: See Table 3**							X	

For peer review only

For peer review only - http://bmjopen.bmj.com/site/about/guidelines.xhtml

**Exercise adherence**		⟷	⟷	⟷	⟷	⟷	⟷	
**Splint adherence**		⟷	⟷	⟷	⟷	⟷	⟷	
**Qualitative Interviews (selected participants)**						X		
**Patient request for further treatment**								X

Table 2. Schedule of enrolment, interventions, and assessments

*The eligibility screen and informed consent can optionally be carried out prior to time point 0.

Measure	Screening	Baseline	4 weeks	8 weeks	12 weeks
**Work Productivity and Activity Impairment Questionnaire (WPAI)** [43]		✓		✓	✓
**Generic Quality of Life (SF12-V2)** [44]		✓		✓	✓
**EuroQol 5 Dimensions 5-Levels questionnaire (EQ5D-5L)** [45]		✓		✓	✓
**AUSCAN hand stiffness** [37]		✓		✓	✓
**Michigan Hand Questionnaire (MHQ)** [46]		✓		✓	✓
**Thumb pain over the last week**		✓		✓	✓
**Disability of the arm, shoulder, hand questionnaire (DASH)** [47]		✓		✓	✓

For peer review only - http://bmjopen.bmj.com/site/about/guidelines.xhtml
For peer review only

**Leisure section only**					
**Arthritis Self-Efficacy Pain Scale** [48]		✓		✓	✓
**AUSCAN hand pain** [37]	✓			✓	✓
**AUSCAN hand function** [37]	✓			✓	✓
**Global assessment of change** [49]				✓	✓
**Health utilisation questionnaire**				✓	✓
**The objective clinician assessed Grip Ability Test (GAT)** [42]		✓	✓	✓	

Table 3. Baseline assessment and outcome measures.

For peer review only

The Global Assessment of Change [49] question adapted for the OTTER II study asks “With respect to your thumb base pain how would you describe yourself now as compared to the start of your OTTER trial therapy treatment” The answer is given on a 5 point Likert scale, that that response range from “very much worse”, “worse”, over “no change” to “better” and “completely recovered”. The AUSCAN hand index for pain [37] recorded at baseline and 8 weeks is the primary outcome measure. The CRFs for this study can be obtained from the first author (JA)

Sample Size

The sample size has been calculated based on undertaking a global Analysis of Covariance (ANCOVA) for the primary outcome, AUSCAN hand pain [37] at 8 weeks, adjusting for the baseline pain score and stratification factors (including centre and treated hand dominance) across all three treatment arms using the power and sample size package, PASS 11 (PASS 11. NCSS, LLC. Kaysville, Utah, USA. www.ncss.com). Assuming 80% power, a 5% 2-sided significance in order to detect a standardised mean difference of 0.4 (a moderate effect size [50]based on a difference in the AUSCAN hand pain score of 2 points and assuming a standard deviation of 5, based on data from the OTTER pilot study

For peer review only - http://bmjopen.bmj.com/site/about/guidelines.xhtml

[31], requires 92 participants per arm. Allowing a 20% loss to follow-up at 8 weeks inflates this to 115 participants per arm, giving a total of 345 participants.

The sample size has taken into account the global comparison of the null hypothesis that there is no difference between the three treatment arms, and pairwise comparisons will only be undertaken if this global comparison is statistically significant. No further adjustment for the sample size has been undertaken to allow for multiple testing.

Clustering is not a consideration in sample size as "Centre" is a stratification factor, and although there may be more than one therapist per centre, this should largely ensure balance across treatment arms within each centre. The Data and Safety Monitoring Committee (DSMC) (Appendix 8) will review the assumptions underlying this sample size calculation after approximately 50% of the participants have been recruited and followed up for 8 weeks, if still within the recruiting period.

Recruitment strategy

Recruitment targets and procedures have been tested in the pilot study [32] and are based on these data. Recruitment start dates for the collaborating sites will be staggered over a 6-month period.

Data collection, management and analysis

The trial will use a series of CRFs to record trial activities and RCT site staff will ensure that each CRF is completed properly and stored in the Investigator’s Site File (ISF). Site staff will make photocopies and post CRFs to the OTTER Trial team using FREEPOST envelopes, according to a standard operating procedure (SOP), so that both sites and the trial centre will have a copy of CRFs.

Participants will complete questionnaires at baseline, 8 weeks and 12 weeks. Participants will be asked to record the frequency for which hand exercises were completed for at least 20 minutes using a daily diary. Splint wear diaries will be used to capture hours per day of splint wear and will contribute to per protocol analysis of data. Participants will be provided with FREEPOST envelopes in order to return questionnaires and diaries to the trial centre.

For peer review only - http://bmjopen.bmj.com/site/about/guidelines.xhtml

Where postal follow-up questionnaires are not received when expected, first they will be chased by post, and if there is no response key endpoint data will be collected over the phone. Up to three phone calls will be made to obtain key endpoint data. If participants wish to withdraw from their randomised intervention, they will still be included in the trial follow-up, unless they request to be excluded from follow-up. Where available, reasons for withdrawal from follow-up will be collected.

The OTTER Research Team will make copies of CRFs, questionnaires and diaries created by or received directly at the trial centre and post them to the relevant site to be filed in the ISF. The original documents will be secured in the Trial Master File.

Eligibility and stratification data will be entered directly onto the OCTRU online randomisation system by site staff. All other data will be entered into the trial database by the OTTER Research Team. The OTTER trial team will check each CRF for completeness and where appropriate contact the site for missing data. A Data Management and Sharing Plan contains fully comprehensive information about all aspects of data management. All data will be processed according to the Data Protection Act 2018. All study specific documents, except for the signed consent form and letters to participants, will refer to the participant with a unique study participant number/code and not by name.

Quantitative data will be stored on an OpenClinica trial-specific database prepared and managed by OCTRU. Qualitative data will be stored in QRS NVivo and word documents. The database has inbuilt data validation checks and a trial management system for managing data queries. Peer review of data entry will be conducted on 5% of CRFs. Data discrepancies will be reviewed on a regular basis to help clean the data. All data will be securely stored only accessible by authorised personnel agreed by the Principal Investigator, Legal services at the University of Southampton and The OTTER II Trial Steering Committee. Data will be backed up, and participant identifiable data will be stored separately from study data. Trial documentation will be retained for 10 years after completion of study-related activities and managed in accordance with the University of Southampton and the University of Oxford research data management policies.

For peer review only - http://bmjopen.bmj.com/site/about/guidelines.xhtml

Audio-recordings from the interviews will be transcribed verbatim, and participants allocated an ID number. The text of the interviewer notes from the telephone interviews will be anonymised and linked to the interview through the ID number. Names of participants, the names of any people discussed, health care employees and hospitals will be removed from transcripts and replaced with pseudonymns. Participants will be sent a letter to thank them for taking part in the telephone interview. A 'future use of interview' question is included on the consent form which gives copyright to the Universities of Southampton and Oxford to use the material in research, teaching, publications and broadcasting.

Statistical analysis

The statistical analysis will be carried out by the OTTER II Trial OCTRU trial statistician. As is usual practice for the Oxford Cinical Trials Unit the statistician will not be blind to treatment allocation. There are rigourous checks and balances in place to ensure that the trial statistician cannot bias outcome.

This three-arm trial will first assess the global comparison of differences between the three treatment arms at the 5% level. The primary (global) Null Hypothesis is that there is no difference between the three arms. Only if this null hypothesis is rejected at the 5% (2-sided) significance level will the pairwise comparisons be carried out in order to explore where the difference lies:

· Verum splint + self-management programme (Group B) versus self-management programme alone (Group A)

· Placebo splint + self-management programme (Group C) versus self-management progamme alone (Group A)

· Verum splint + self-management programme (Group B) versus placebo splint + self-management programme (Group C)

It is anticipated that there will be an adjustment for multiple testing at this stage, and Bonferroni or a less conservative method will be used. The main analysis will be intention to treat. A per-protocol

For peer review only - http://bmjopen.bmj.com/site/about/guidelines.xhtml

analysis of the primary endpoint will be performed as part of the sensitivity analyses. A Statistical Analysis Plan will provide full details of all planned analyses.

The statistical analysis of the primary outcome, AUSCAN hand pain score, will be performed using ANCOVA, adjusting for baseline pain and stratification factors. Continuous secondary outcomes will be analysed using similar methods to the primary outcome. The unadjusted secondary binary variables will be compared using chi-squared tests with logistic regression being used to adjust for stratification and important prognostic factors in a multi-variable framework.

Baseline characteristics for the three groups will be presented using the appropriate descriptive statistics. Baseline characteristics of participants who completed the trial and those who dropped out will be presented and explored in order to ascertain patterns of loss to follow-up. Subgroups will be examined to look for consistency of any observed treatment effects (using interactions). This analysis will be considered of an exploratory nature potentially providing hypotheses for future studies. The study is not powered to examine these in detail and will not therefore report p-values for these.

Qualitative analysis

Forty interviews will be carried out by a researcher blind to participant treatment allocation. Audio-recordings of the 40 interviews will be transcribed verbatim, anonymised and imported into analysis software QRS NVivo. The researcher will conduct primary coding of the narrative data and the five stages of framework analysis as described by Ritchie and Lewis [51] will be followed: familiarisation, identifying a thematic framework, indexing, charting and mapping/ interpretation. Using a thematic approach [52] data will be inductively coded, codes grouped to create categories and a descriptive account will be produced. Up to 10 interviews will be double coded to increase the validity of the findings. Double coding means more than one researcher independently assigning pre-specified codes to the qualitative data. NVivo software (QSR International) will be used to facilitate the double coding process. This allows for coding comparisons to be run between different coders. The results are returned according to the Kappa coefficient score and the percentage of agreement between coders. After double coding transcripts, we plan to use this coding comparison for

For peer review only - http://bmjopen.bmj.com/site/about/guidelines.xhtml

discussion and reflection on the data. The numeric measure will be used as a method of comparison to gauge if there is agreement and understanding of the definitions of the codes between team members. Any discrepancies identified will then be discussed until an agreement is reached and any differences resolved. The account will provide structured descriptions of participants' experience of self-management and splints, including reasons for their views about effectiveness as well as information about acceptability and adherence.

Quality of Life Analysis

The EQ-5D-5L [53] will be administered to participants at baseline, 8 weeks and 12 weeks. Responses will be converted into utilities using tariffs estimated from a representative sample of the UK population [54]. Survival information collected from the trial will be combined with EQ-5D utilities to generate Quality Adjusted Life-Years (QALYs).

Economic Evaluation and Analysis

The perspective adopted in the economic analysis will be that of the NHS. Costs associated with the following healthcare resource categories over the 8 week intervention period and follow-up period (12 weeks) will be included.

· Intervention provision (including: splints and clinical staff time required for splinting); and

· Primary care contacts, including surgery and home visits by GPs, nurses, and out-of-hours medical services, and community therapists; and

· Hospital care services, including scheduled and unscheduled inpatient admissions, surgery, accident and emergency visits and outpatient care contacts.

Primary and hospital care resource use will be obtained from patient questionnaires administered at 8 weeks and at 12 week follow-up. Healthcare resources will be valued using unit cost schedules such as Personal Social Services Research Unit and NHS Reference costs. Costs associated with splints and other disposables will be obtained from the manufacturers and the NHS Supply Chain catalogue. Using the WPAI [43] the number of work days lost by study participants and the impact that OA had on the levels of productivity/activity and unpaid work will also be measured over both study periods.

For peer review only - http://bmjopen.bmj.com/site/about/guidelines.xhtml

For peer review only

An economic evaluation adherent to guidelines for good economic evaluation practice [55] will be undertaken A within-trial cost-utility analysis will explore the incremental cost per QALY gained by splinting of the thumb base and a self-management programme when compared to: 1) placebo-splinting and a self-management programme; and 2) a self-management programme alone. The analyses will be conducted at eight weeks and at 12 weeks. Cost and effect results will be reported as means with standard deviations, with mean differences between the two patient groups reported alongside 95% confidence intervals. Depending on the amount of missing cost and quality of life data, missing data will be imputed using recommended multiple imputation methods [56], with results from this analysis being presented as an additional sensitivity analysis.

Incremental cost-effectiveness will be calculated by dividing the difference in costs by the difference in effects. Uncertainty around the incremental cost-effectiveness ratio will be explored using non-parametric bootstrapping. A supplementary economic evaluation including non-NHS costs will be conducted in an additional sensitivity analysis. This will include costs of impaired productivity/activity and, any work and non-work days (e.g. leisure or non-paid work) lost due to illness will be valued using mean average wages for those in employment. For participants who are retired or those not in employment, loss in activity levels (e.g. leisure, caring or non-paid work activities) due to illness will be valued using minimum wages.

ETHICS AND DISSEMINATION

South Central - Oxford C Research Ethics Committee approved the study and the OTTER II trial will undergo regular monitoring. The quality assurance evaluation of trial processes and intervention fidelity using a trial monitoring template will be carried out on 50% of the recruiting sites by the research team. Should there be no noted issues no follow-up monitoring will be conducted. There are no criteria that will flag a need to initiate a quality evaluation, however, continual scrutiny of the CRFs received from sites by the study team, will alert the research team to possible issues (e.g. incomplete completion of CRFs, missing data or mis-randomisation) that may subsequently require

For peer review only - http://bmjopen.bmj.com/site/about/guidelines.xhtml

quality assurance evaluation. A standard risk assessment will be conducted and a risk-based proportionate monitoring plan will be put in place, which will include central monitoring.

Adverse event and safety oversight will comply with OCTRU SOPs. Adverse Responses/Reactions, Adverse Device Effects, Serious Adverse Responses/Reactions, Serious Adverse Device Effects, Suspected Unexpected Serious Adverse Responses/Reactions (SUSARs) and Device Deficiencies will be recorded as study outcomes. Sites will be required to report all serious events that are related to a trial intervention within 24 hours and all related serious adverse events must be assessed for causality and reason for seriousness. All Device Deficiencies, Adverse Reactions and Adverse Device Effects must be reported to the trial team within 14 days. All Serious Adverse Event safety monitoring forms will be passed on to the Trial's Nominated Clinician who will perform an independent assessment of causality and will also perform assessment of expectedness based on what is known and documented about the intervention/device. Any SUSARs will be reported to the Research Ethics Committee within 15 days of the Trials Office being made aware of the event

An independent DSMC (Appendix 9) will be convened with two expert clinicians and a statistician to regularly review accumulating data in order to assess patient safety and study conduct following the recommendations of the DAMOCLES study [57] with full details being provided in a charter. They will advise the Trial Steering Committee (Appendix 8) as to whether recruitment should stop early, which is only likely to occur if either intervention is shown to be unsafe for patients. As this is a low risk study no interim efficacy comparative analyses are planned and no safety issues are expected. The OTTER II trial team will have access to the final trial dataset and there will be no public access to patient level data or the statistical code used within the trial.

We believe this to be the first fully powered placebo controlled splinting trial exploring the effectiveness and efficacy of splints in symptomatic thumb base OA. Study results will be disseminated to rheumatology, hand therapy, occupational therapy and physiotherapy national and international conferences and submitted for consideration in international and national academic and professional conferences and journals. Lay publications written in accessible language will be provided to charitable, community and patient facing publications as recommended by our patient

For peer review only - http://bmjopen.bmj.com/site/about/guidelines.xhtml

and public involvement partners. Study participants will be provided regular updates of the study progress through the OTTER II study website.

For peer review only

For peer review only - http://bmjopen.bmj.com/site/about/guidelines.xhtml

Authors' contributions:

Jo Adams: Principal investigator, conceived research project and first author of paper

Paula Barratt: Trial manager, written and reviewed trial protocol and co-author

Nigel Arden: Trial team member contributed to original protocol and co-applicant and co-author

Sofia Barbosa Bouças: Trial team member contributed to pilot development and original protocol, co-applicant and co-author

Sarah Bradley: Trial team member contributed to original protocol and co-applicant and co-author

Michael Doherty: Trial team member contributed to original protocol and co-applicant and co-author

Susan Dutton: Trial team member contributed to original protocol, statistical lead, co-applicant and co-author

Krysia Dziedzic: Trial team member contributed to original protocol and co-applicant and co-author

Rachael Gooberman-Hill: Trial team member contributed to original protocol and co-applicant and co-author

Kelly Hislop: Trial team member contributed to pilot development and original protocol, co-applicant and co-author

Corinne Hutt Greenyer: OTTER patient partner, co-applicant, co-author, reviewed and revised paper

For peer review only - http://bmjopen.bmj.com/site/about/guidelines.xhtml

Vic Jansen: Trial team member contributed to revision and clinical review of original protocol and co-author

Ramon Luengo Fernadez: Trial team member contributed to original protocol and co-applicant and co-author, led health economic input

Claire Meagher: Trial team member, reviewed and revised qualitative component and co-author

Peter White: Trial team member contributed to original protocol and placebo development and co-applicant and co-author

Mark Williams: Trial team member contributed to original protocol reviewed and revised paper

Funding statement: 'This work was supported by Arthritis Research UK Grant Ref 21019 and the funder is listed and complies with the SHERPA JULIET guidelines

http://v2.sherpa.ac.uk/id/funder/14

Competing interests statement. The authors and wider OTTER II trial team have no competing interests

Acknowledgements.

The authors wish to acknowledge the OTTER II collaborative group:

Derby Teaching Hospitals NHS Foundation Trust: Victoria Jansen, Helen McKenna, Ellen Bramall, Carole Henderson, Chloe Kirk, Diane Langford, James Turner, Anna Selby, Linda Tozer, Navdeep Johal, Fernando Parrales, Madina Asif, Kat Hill, Stefania Wigelsworth

Doncaster and Bassetlaw Teaching Hospitals NHS Foundation Trust: Su McIlwaine, Elaine Bonser, Lynn Houghton, Ryan Roberts

Dorset County Hospital NHS Foundation Trust: Maree Dethick-Jones, Sheena Colhoun, Louise Clark, Liz Barnish, Simone Caddy, Josie Goodsell, Sarah Horton, Laura Howell, Simon Sharpe

Hampshire Hospitals NHS Foundation Trust: Kevin Spear, Christina Macleod, Libby Denman, Becky Shaylor, Kathy Whalley, Mark Pulley, Hannah Bolger

Midlands Partnership NHS Foundation Trust: Carol Graham, Nicky Edwards, Jane Rivers-Latham, Yvonne Salt, Tilly Grocott, Alison Williams, Sarah Gibson, Lisa Oakley, Susan Thompson, Susan Woodroffe, Laura Denny, Amy Thompson, Natalie Wheat

North Devon Healthcare NHS Trust: Jo Harness, Jane Hunt, Henrietta Clay, Becky Holbrook, Lucia Stancombe, Martin Howard, Nicholas McGuirk, Mark Bryce

For peer review only - http://bmjopen.bmj.com/site/about/guidelines.xhtml

Pennine MSK Partnership Ltd: Jill Firth, Kathy Kinsey, Helen Light, Charlotte Critchley, David Pilbury, Danielle Burke, Karen Partridge, Tracy Parry, Norah Handley, Kath Spencer
Poole Hospital NHS Foundation Trust: Sarah Bradley, Corinna Rogers, Paula Reynolds, Bridget Ellis, Sharon Page
Portsmouth Hospitals NHS Trust: Caroline Mountain, Gemma Willis, Catherine Coleman, Catherine Kirby, Paula White, Glenn Lake, Julie Williams, Marie White
Royal Devon and Exeter NHS Foundation Trust: Suzannah Blake, Abigail Owen, Emily Rogers, Claire Hughes, Cresta Browning, Jacqueline Fowler, Cristina Burke-Trees
Royal Free London NHS Foundation Trust: Juliette Bray, Nikki Burr, Meera Anadkat, Cherry Kilbride, Francesca Gowing
Royal United Hospitals Bath NHS Foundation Trust: Sandi Derham, Jessica Chipps, Suzanne Green, Helen Gordon-Johnson, Mark Sheriff, Susan Greene, Michelle Lawrence, Belinda Jones, Jack Spence, Ali Champion, Emily Anson
The Robert Jones and Agnes Hunt Orthopaedic Hospital NHS Foundation Trust: Tracy Jones, Michelle Jones, Julie Steen, Daniel Griffiths, Sally van Liefland, Jayne Edwards, Linda Griffith
Yeovil District Hospital NHS Foundation Trust: Helen Truman, Frances Campbell, Helen Jones, Jennifer Harrison, Vickie Ridley, Sue Chesterman, Joanna Allison, Tressy Pitt-Kerby, Kate Beesley

The authors also wish to acknowledge the OTTER II Trial Administrator: Carrie Fanning, University of Southampton, Southampton, UK.

Legend for Figure 1:

Figure 1. Participants' process throughout the trial

For peer review only

For peer review only - http://bmjopen.bmj.com/site/about/guidelines.xhtml

References

1. Abhishek A, Doherty M. Clinical Features of Osteoarthritis In: Watts R, Conaghan P, Denton C, et al., eds. Oxford Textbook of Rheumatology (4 ed). Oxford Oxford University Press, 2013.

2. Maheu E, Berenbaum F, . Time for new outcome measures in hand osteoarthritis? . F Nat Clin Pract Rheumatol 2009;**5():.**(3):136-38

3. Marshall M, Peat G, Nicholls E, et al. Subsets of symptomatic hand osteoarthritis in community-dwelling older adults in the United Kingdom: prevalence, inter-relationships, risk factor profiles and clinical characteristics at baseline and 3-years
Osteoarthritis and Cartilage
2013;**21**(11):1674-84

4. Haugen IK, Englund M, Aliabadi P, et al. Prevlance, incidence and progression of hand osteoarthritis in the general population:the Framingham osteoarthritis study. 2011 doi: 10.1136/ard.2011.150078[published Online First: Epub Date]|.

5. Bijsterbosch J, Visser W, Kroon H, et al. Thumb base involvement in symptomatic hand osteoarthritis is associated with more pain and functional disability. Ann Rheum Dis 2010;**69**(3):585-87 doi: doi: 10.1136/ard.2009.104562. Epub 2010 Feb 2[published Online First: Epub Date]|.

6. Zhang W, Doherty M, Leeb B, et al. EULAR evidence-based recommendations for the diagnosis of hand osteoarthritis: report of a task force of ESCISIT. Ann Rheum Dis 2009;**68**:8-17 doi: doi:10.1136/ard.2007.084772
[published Online First: Epub Date]|.

7. Spacek E, Poiraudeau S, Fayad F, et al. Disability induced by hand osteoarthritis: are patients with more symptoms at digits 2-5 interphalangeal joints different from those with more symptoms at the base of the thumb?
. Osteoarthritis and Cartilage 2004;**12**:366-73

8. Kwok WY, Vliet Vlieland TPM, Rosendaal FR, et al. Limitations in daily activities are the major determinant of reduced health-related quality of life in patients with hand osteoarthritis. Annals of the Rheumatic Diseases 2011;**70**(2):334-36 doi: 10.1136/ard.2010.133603[published Online First: Epub Date]|.

9. Van Den Ende C, Stukstette M, Dekker J, et al. Thumb base involvement in established hand osteoarthritis Annals of the Rheumatic Diseases 2013;**72**(S3):784 doi: 10.1136/annrheumdis-2013[published Online First: Epub Date]|.

10. Poole JU, Pellegrini VD, Jr. Arthritis of the thumb basal joint complex. Journal of hand therapy : official journal of the American Society of Hand Therapists 2000;**13**(2):91-107

11. Zhang Y, Niu J, Kelly-Hayes M, et al. Prevalence of symptomatic hand osteoarthritis and its impact on functional status among the elderly: The Framingham Study. Am J Epidemiol 2002;**156**(11):1021-7

12. Hill S, Dziedzic K, Ong B. The functional and psychological impact of hand osteoarthritis. Chronic Illness 2010;**6**:101-10

13. Bani MA, Arazpour M, Curran S. Design and construction of custom-made neoprene thumb carpo-metacarpal orthosis with thermoplastic stabilization for first carpo-metacarpal joint osteoarthritis. Journal of Hand Therapy 2013;**26**(3):279-81 doi: https://doi.org/10.1016/j.jht.2013.01.005[published Online First: Epub Date]|.

14. Grenier M-L, Mendonca R, Dalley P. The effectiveness of orthoses in the conservative management of thumb CMC joint osteoarthritis: An analysis of functional pinch

For peer review only - http://bmjopen.bmj.com/site/about/guidelines.xhtml

strength. Journal of Hand Therapy 2016;**29**(3):307-13 doi: https://doi.org/10.1016/j.jht.2016.02.004[published Online First: Epub Date]|.

15. Colditz JC. The biomechanics of a thumb carpometacarpal immobilization splint: Design and fitting. Journal of Hand Therapy 2000;**13**(3):228-35 doi: 10.1016/S0894-1130(00)80006-X[published Online First: Epub Date]|.
16. Hamann N, Heidemann J, Heinrich K, et al. Stabilization effectiveness and functionality of different thumb orthoses in female patients with first carpometacarpal joint osteoarthritis. Clinical Biomechanics 2014;**29**(10):1170-76 doi: https://doi.org/10.1016/j.clinbiomech.2014.09.007[published Online First: Epub Date]|.
17. Kloppenburg M, Kroon FP, Blanco FJ, et al. 2018 update of the EULAR recommendations for the management of hand osteoarthritis. Annals of the Rheumatic Diseases 2018 doi: 10.1136/annrheumdis-2018-213826[published Online First: Epub Date]|.
18. Arazpour M, Soflaei M, Ahmadi Bani M, et al. The effect of thumb splinting on thenar muscles atrophy, pain, and function in subjects with thumb carpometacarpal joint osteoarthritis. Prosthetics and Orthotics International 2016;**41**(4):379-86 doi: 10.1177/0309364616664149[published Online First: Epub Date]|.
19. Hermann M, Nilsen T, Eriksen CS, et al. Effects of a soft prefabricated thumb orthosis in carpometacarpal osteoarthritis. Scandinavian Journal of Occupational Therapy 2014;**21**(1):31-39 doi: 10.3109/11038128.2013.851735[published Online First: Epub Date]|.
20. Zhang W, Doherty M, Leeb BF, et al. EULAR evidence based recommendations for the management of hand osteoarthritis: report of a Task Force of the EULAR Standing Committee for International Clinical Studies Including Therapeutics (ESCISIT). Ann Rheum Dis 2007;**66**(3):377-88
21. Kjeken I, Darre S, Smedslund G, et al. Effect of assistive technology in hand osteoarthritis: a randomised controlled trial. Annals of the Rheumatic Diseases 2011;**70**(8):1447-52 doi: 10.1136/ard.2010.148668[published Online First: Epub Date]|.
22. Bertozzi L, Valdes K, Vanti C, et al. Investigation of the effect of conservative interventions in thumb carpometacarpal osteoarthritis: systematic review and meta-analysis. Disability and rehabilitation 2015;**37**(22):2025-43 doi: 10.3109/09638288.2014.996299[published Online First: Epub Date]|.
23. Sanchez K, Jourdan C, Rannou F, et al. Assessing Orthosis:Practical Examples. In: Boutron I, Ravaud P, Moher D, eds. Randomized Clinical trials of Nonpharmacological Treatments: Chapman and Hall/CRC Biostatistics Series 2012:295-307.
24. Buhler M, Chapple CM, Stebbings S, et al. Effectiveness of splinting for pain and function in people with thumb carpometacarpal osteoarthritis: a systematic review with meta-analysis. Osteoarthritis Cartilage 2019;**27**(4):547-59 doi: 10.1016/j.joca.2018.09.012[published Online First: Epub Date]|.
25. White P, Bishop F, Prescott P, et al. Practice, practitioner, or placebo? A multifactorial, mixed-methods randomized controlled trial of acupuncture
Pain 2012;**153**(2):455-62
26. Finniss D, Kaptchuk T, Miller F, et al. Biological,clinical, and ethical advances of palcebo effects The Lancet 2010;**375**:868-695
27. Chan A-W, Tetzlaff JM, Gøtzsche PC, et al. SPIRIT 2013 Explanation and Elaboration: Guidance for protocols of clinical trials. Bmj 2013;**346**

For peer review only - http://bmjopen.bmj.com/site/about/guidelines.xhtml

e7586 doi: https://doi.org/10.1136/bmj.e7586[published Online First: Epub Date]|.
28. Gooberman Hill R, Jinks C, Barbosa Boucas S, et al. Designing a placebo device: involving service users in clinical trial design. Health Expectations 2013;**16**(4):e100–e10 doi: doi:10.1111/hex.12043[published Online First: Epub Date]|.
29. Barbosa Boucas S, Hislop K, Dziedic K, et al. Defining optimal NHS occupational therapy treatment, individualized splint and placebo splint for patients with thumb base OA: a Delphi study. Rheumatology 2013a;**52**(S1):i105
30. Barbosa Boucas S, Hislop Lennie K, Dziedzic K, et al. Differences between service providers and users when defining feasible optimal NHS occupational therapy treatment for patients with thumb base OA: Results from a Delphi study. Annals of the Rheumatic Diseases 2013b;**72**(S3):779-80
31. Jones L, White P, Donovan-Hall M, et al. The Thoughts and Feelings Held by Clinicians about the delivery of a Placebo Thumb Splint in an Osteoarthritis Rehabilitation Trial. Hand Therapy 2013;**18**(3):77-83
32. Adams J, Barbosa Bouças S, Hislop K, et al. The Effectiveness and Efficacy of Splints for Thumb Base Osteoarthritis: A Pilot Randomized Controlled Trial. Rheumatology 2014;**53**(suppl_1):i41-i42 doi: 10.1093/rheumatology/keu090.005[published Online First: Epub Date]|.
33. Adams J, Barratt P, Bradley S, et al. Participating in a musculoskeletal randomised controlled trial: identification of education training needs by occupational therapists and physiotherapists in the uk. Annals of the Rheumatic Diseases 2017;**76**(Suppl 2):1508-08 doi: 10.1136/annrheumdis-2017-eular.1095[published Online First: Epub Date]|.
34. Gossec L, Dougados M. Intra-articular treatments in osteoarthritis: from the symptomatic to the structure modifying. Annals of the Rheumatic Diseases 2004;**63**(5):478 doi: 10.1136/ard.2003.013771[published Online First: Epub Date]|.
35. Trellu S, Dadoun S, Berenbaum F, et al. Intra-articular injections in thumb osteoarthritis: A systematic review and meta-analysis of randomized controlled trials. Joint Bone Spine 2015;**82**(5):315-19 doi: https://doi.org/10.1016/j.jbspin.2015.02.002[published Online First: Epub Date]|.
36. Heyworth BE, Lee JH, Kim PD, et al. Hylan versus corticosteroid versus placebo for treatment of basal joint arthritis: a prospective, randomized, double-blinded clinical trial. J Hand Surg Am 2008;**33**(1):40-8 doi: 10.1016/j.jhsa.2007.10.009[published Online First: Epub Date]|.
37. Bellamy N, Campbell J, Haraoui B, et al. Clinimetric properties of the AUSCAN Osteoarthritis Hand Index: an evaluation of reliability, validity and responsiveness. Osteoarthritis Cartilage 2002;**10**(11):863-9
38. Gelberman RH, Boone S, Osei DA, et al. Trapeziometacarpal Arthritis: A Prospective Clinical Evaluation of the Thumb Adduction and Extension Provocative Tests. J Hand Surg Am 2015;**40**(7):1285-91 doi: 10.1016/j.jhsa.2015.04.012[published Online First: Epub Date]|.
39. Kjeken I, Smedslund G, Moe RH, et al. Systematic Review of Design and Effects of Splints and Exercise Programs in Hand Osteoarthritis. Arthritis Care & Research 2011;**63**(6):834-48 doi: 10.1002/acr.20427[published Online First: Epub Date]|.
40. Loyley MST, Davis L, Worsley P, et al. E054 Comparison of the functional impact of verum and placebo thumb base orthoses: a proof of concept study. Rheumatology

For peer review only - http://bmjopen.bmj.com/site/about/guidelines.xhtml

2019;**58**(Supplement_3) doi: 10.1093/rheumatology/kez110.053[published Online First: Epub Date]|.
41. NIHR. Good Clinical Practice Secondary Good Clinical Practice 2019. https://www.nihr.ac.uk/our-research-community/clinical-research-staff/learning-and-development/national-directory/good-clinical-practice/.
42. Dellhag B, Bjelle A. A grip ability test for use in rheumatology practice. The Journal of Rheumatology 1995;**22**(8):1559-65
43. Reilly MC, Zbrozek AS, Dukes EM. The validity and reproducibility of a work productivity and activity impairment instrument. PharmacoEconomics 1993;**4**(5):353-65
44. Ware JE, Kosinski M, Keller SD. A 12-item short-form health survey, construction of scales and preliminary tests of reliability and validity. . Medical care 1996;**34**(3):220-33
45. Euroqol Group. Euroqol: a new facility for the measurement of health-related quality of life. Health Policy 1990;**16**:199-208
46. Chung KC, Pillsbury MS, Walters MR, et al. Reliability and validity testing of the Michigan Hand Outcomes Questionnaire The Journal of Hand Surgery 1998;**23A**(4):575-87
47. Hudak PL, Amadio PC, Bombardier C. Development of an upper extremity outcome measure: The DASH (Disabilities of the Arm, Shoulder and Hand). American Journal of Industrial Medicine 1996;**29**:602-08
48. Lorig K, Chastain R, Ung E, et al. Development and evaluation of a scale to measure self-efficacy in people with arthritis. Arthritis Rheum 1989;**32**(1):37-44
49. Pham T, van der Heijde D, Altman RD, et al. OMERACT-OARSI initiative: Osteoarthritis Research Society International set of responder criteria for osteoarthritis clinical trials revisited. Osteoarthritis Cartilage 2004;**12**(5):389-99
50. J. C. *Statistical Power Analysis for the Behavioral Sciences*. New York Routledge Academic

1988.
51. Ritchie J, Lewis J. *Qualitative research practice: a guide for social science students and researchers*: Sage, 2003.
52. Charmaz K. *Constructing grounded theory: a practical guide through qualitative analysis* London Sage 2006.
53. EuroQol--a new facility for the measurement of health-related quality of life. Health Policy 1990;**16**(3):199-208
54. van Hout B, Janssen MF, Feng YS, et al. Interim scoring for the EQ-5D-5L to EQ-5D-3L value sets. Value Health 2012;**15**:708-15
55. Drummond MF, Sculpher MJ, Claxton K, et al. *Methods for the Economic Evaluation of Health Care Programmes*. 4th Edition ed: Oxford University Press, 2015.
56. Royston P. Multiple imputation of missing values. Stata Journal 2004;**4**:227-41
57. DAMOCLES Study Group NHS Health Technology Assessment Programme. A proposed charter for clinical trial data monitoring committees: helping them to do their job well. Lancet 2005;**365**:711-22

For peer review only

For peer review only - http://bmjopen.bmj.com/site/about/guidelines.xhtml

OTTER II Trial: flow of participants

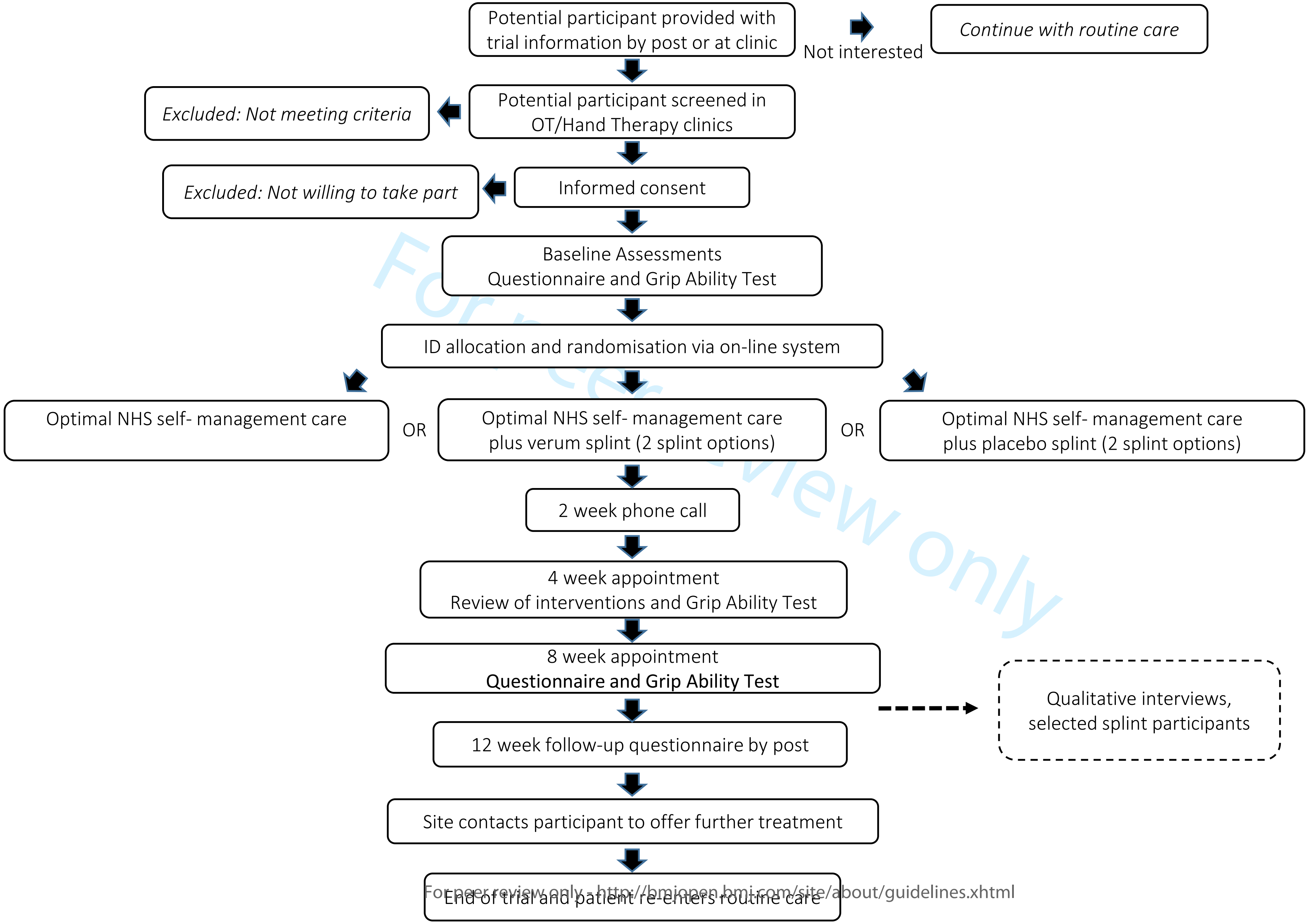

For peer review only

For peer review only - http://bmjopen.bmj.com/site/about/guidelines.xhtml

Appendix 1

NHS Recruitment Sites

Pulvertaft Hand Centre, Royal Derby Hospital, Derby Teaching Hospitals NHS Foundation Trust, Derby, UK

Hand Therapy Department, Dorset County Hospital, Dorset County Hospital NHS Foundation Trust, Dorchester, UK

Hand Therapy Unit, Poole Hospital, Poole Hospital NHS Foundation Trust, Poole, UK

Plastic Surgery Rehabilitation, Royal Devon and Exeter Hospital, Royal Devon and Exeter NHS Foundation Trust, Exeter, UK

Occupational Therapy Department, North Devon District Hospital, North Devon Healthcare NHS Trust, Barnstaple, UK

Rheumatology Therapy Department, Royal National Hospital for Rheumatic Diseases, Royal United Hospitals Bath NHS Foundation Trust, Bath, UK

Therapies Department, Royal United Hospitals, Royal United Hospitals Bath NHS Foundation Trust, Bath, UK

Occupational Therapy Department, Haywood Hospital, Midlands Partnership NHS Foundation Trust, Stoke-on-Trent, UK

Pennine MSK Partnership Ltd., Integrated Care Centre, Oldham, UK

Clinical Therapies Department, Bassetlaw Hospital, Doncaster and Bassetlaw Teaching Hospitals NHS Foundation Trust, Worksop, UK

Bassetlaw Locality Retford Primary Care Centre, Doncaster and Bassetlaw Teaching Hospitals NHS Foundation Trust, Retford, UK

Therapy Department, Yeovil Hospital, Yeovil District Hospital NHS Foundation Trust, Yeovil, UK

Hand Therapy, Basingstoke and North Hampshire Hospital, Hampshire Hospitals NHS Foundation Trust, Basingstoke, UK

Therapy Department, Royal Hampshire County Hospital, Hampshire Hospitals NHS Foundation Trust, Winchester, UK

Occupational Therapy Department, Queen Alexandra Hospital, Portsmouth Hospitals NHS Trust, Cosham, UK

Hand Therapy Unit, The Robert Jones and Agnes Hunt Orthopaedic Hospital, The Robert Jones and Agnes Hunt Orthopaedic Hospital NHS Foundation Trust, Oswestry, UK

For peer review only

Hand Therapy, The Royal Free Hospital, Royal Free London NHS Foundation Trust, London, UK

For peer review only

Appendix 2

Self-Management Package:

Hand Exercise Booklet *(see separate supplementary pdf file as too large a file to include)*

For peer review only

Appendix 2

Self-Management Package: Joint Protection Booklet

Osteoarthritis Thumb Base Therapy Trial

Osteoarthritis
Joint Protection Booklet

Information and advice about reducing pain and protecting your joints

OTTER II trial – REC ref: 16/SC/0188

For peer review only

Osteoarthritis

You have been given this booklet because you have pain or discomfort in one or more of your joints.

Osteoarthritis is a common cause of joint pain effecting 20% of the general population aged over 55 years.

Osteoarthritis can affect many aspects of your everyday life. This booklet has been designed with the help of clinicians in response to questions asked by patients with Osteoarthritis including advice about how to carry on with your normal life as much as possible whilst reducing pain and protecting your joints from further damage.

This booklet contains information on the following:

1. What is Osteoarthritis?
2. Protecting your joints
3. Dealing with fatigue
4. Diet
5. Holistic Medicine

For peer review only

1. What is Osteoarthritis?

A ‘normal’ joint is where two or more bones meet. The joint allows the bones to move freely but within limits.

Osteoarthritis is a disease that affects the body’s joints. The surfaces within the joint are damaged so the joint does not move as smoothly as it should. The main symptoms are pain and sometimes stiffness.

When a joint develops Osteoarthritis, some of the cartilage covering the ends of the bones gradually roughens and becomes thin and the bone underneath thickens. All the tissues within the joint become more active than normal, as if the body is trying to repair the damage.

Sometimes the body’s repairs are quite successful and the changes inside the joint will not cause much pain. If the Osteoarthritis becomes severe, the cartilage can become so thin that it no longer covers the ends of the bones. The bones start to rub against each other and eventually can start to wear away. The loss of cartilage, the wearing of bone and sometimes bony spurs can alter the shape of the joint, forcing the bones out of their normal alignment.

2. Protecting your joints

Joint protection can reduce joint damage, preserve range of motion, and lessen Osteoarthritis pain by reducing the general stress and strain on the joint. Making changes early can help avoid joint problems becoming worse in the future.

Become more aware of how you use the joints that ache, both at home and at work. For example, try watching your actions while you use your hands – for example when you make a hot drink:

- What is happening to your fingers while you are turning the tap? Are they being pushed towards the little finger?
- What happens to your thumbs as you take the lid of the coffee jar? Is there pressure or aching at the base of your thumb?
- What is happening to your wrist and fingers as you lift the kettle? Can you feel aching or pulling at these joints?

For peer review only

You might already have tried picking up the kettle with two hands when your hands are painful, but it is important to do this **all the time**, not just when your hands are hurting. This is an example of joint protection.

For peer review only

The following principles may help you:

- **Respect pain.** If you are experiencing pain after an activity, you must consider that you have been too active or have done too much.
- **Pace your activities throughout the day.** Spread physically hard jobs, such as housework or mowing the lawn, at intervals through the day, rather than tackling them all at once.
- **Avoid any activity that causes pain and find a better way of accomplishing the task.** Avoid a tight grip that strains joints and muscles. Avoid a prolonged or continuous grip. Small joints can take little weight, so gripping a pen or cutlery can be hard work. **Holding a larger object involves more joints, so it spreads the grip.** Picking up an object with two hands halves the load on each hand.

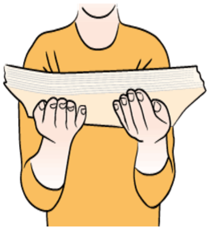

- **Make handles larger with padding,** for example, sponge tubing for pipe lagging. A solid surface is harder to grip than a soft surface, also a larger surface area means less strain. Wear padded gloves when gardening.
- **Use adaptive devices**. There are many devices available on the market to make it easier for you to carry out everyday tasks. Below are examples of some which may make your life easier and reduce the stress on your thumb joint:
    - **Spring loaded scissors** reduce the load on your thumb.

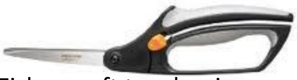

Fiskars soft touch scissors

    - **Jar Keys** can really make a difference as they break the vacuum when opening jars.

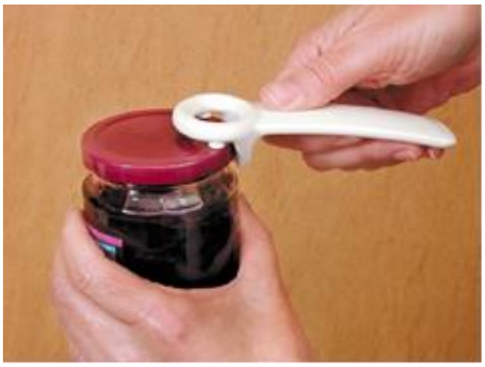

– **L-shaped knifes** do not use the thumb at all.

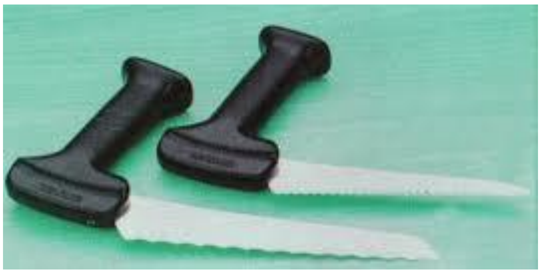

– **Ring pull can openers** like a 'magi pull' available from Lakeland can make opening cans much easier.

– As we age the natural stickiness of our hands decrease and are hands become dryer, this can lead us to have to use 20% more grip strength to open jars, etc. Using a **damp dish cloth** can reduce the amount of grip strength you need to use.

– When straining vegetables, instead of lifting and tipping the pan, **place vegetables in a wire basket in the pan to cook**. When ready they can be lifted and drained in the basket. Leave the saucepan to cool before moving it.

– When using the kettle, **use a plastic jug to fill the kettle from the tap** and only fill the kettle up as required. A **kettle tipper can be used**, or a small lightweight travel kettle. Also, kettles with the handle over the top have been reported to be easier to lift using two hands rather than the kettles with the handle on the side.

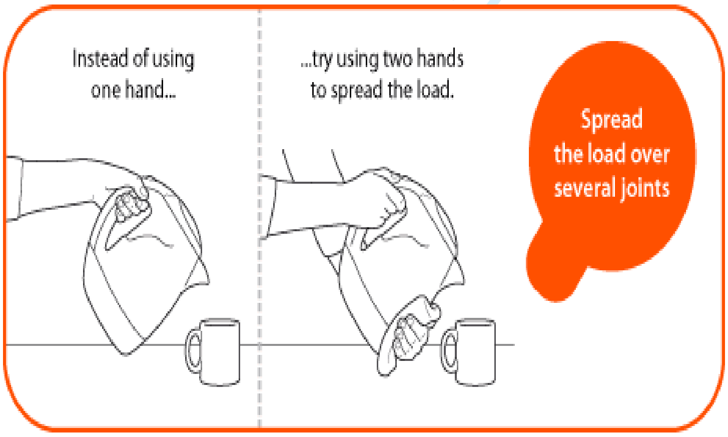

3. Dealing with fatigue

At times people with arthritis may experience fatigue. Fatigue is a feeling of weariness, but it is more extreme than simple tiredness. It can affect you physically, making your limbs seem heavy and causing you to feel exhausted, but it can also affect your concentration and

For peer review only - http://bmjopen.bmj.com/site/about/guidelines.xhtml

motivation. People who experience fatigue may find they struggle to do even small tasks. It often comes on for no apparent reason and without warning. There are many things that can cause fatigue including those listed below:

- Anaemia, which often accompanies inflammation.
- Other long term conditions such as diabetes or thyroid disease.
- Some drugs used to treat arthritis which may cause drowsiness or loss of concentration.
- Pain, especially if it is long term weak muscles, which mean you have to use more energy to do everyday tasks.
- Overdoing things or carrying on with activities for too long.
- Sleep disturbances as a result of pain, late nights or sleeping too much in the day.
- Stress and anxiety.
- Low mood or depression.
- Poor diet or hunger.

For peer review only

What can you do to help yourself?

- **Talk to your GP** or Rheumatology team about getting support or a **review of your medication**.
- Use the **four 'Ps'** – **P**roblem Solving, **P**lanning, **P**rioritising and **P**acing.
- **Tell family, friends and colleagues** about your fatigue so that they can understand and help if needed.
- Gradually **increase your physical exercise**. This will improve your general well-being, strength and energy levels.
- **Deal with stress or anxiety**.
- **Talk to your GP** if you have low mood.
- Improve your **sleeping habits**.
- Eat a **healthy diet**.

4. Diet

Whilst it is not the cause of Osteoarthritis and thumb base pain, being overweight can affect your health in many ways. If you are overweight then losing weight will help reduce the strain on your joints, increase your sense of well-being and reduce your risk of other health problems.

Apart from **reducing sugar** and **fat**, **taking regular exercise,** and ensuring that you **have your five a day of fruit and vegetables**, it has been shown that **increasing** your **intake** of particular vitamins and minerals, particularly **Calcium, Vitamin D** and **Iron**, **eating less saturated fat** and incorporating more **good fats** in your diet such as **Omega 3** can improve symptoms of Arthritis. See table below for a list of good and bad fats.

Saturated Fats (Bad Fats) **Avoid**	Polyunsaturated Fats (Fats that can increase inflammation) **Avoid**	Monounsaturated Fats (Neutral fats but contain a high amount of calories) **Limit your intake**
• Full fat dairy products<br>• Processed foods (for example, cakes, biscuits and pastry)<br>• Chips, if fried in animal fat<br>• Foods cooked using ghee (clarified butter)	• Softer Fats and oils<br>• Corn or sunflower sources of oil	• Olive oil<br>• Rapeseed oil

Omega-3 Polyunsaturated Fatty Acids

These are important to keep in your diet. **Omega-3** has been found to be of benefit to people with Arthritis. It can be found in free range eggs, oily fish and fish oil supplements. The table below shows a list of some oily fish which you should aim to eat at least **twice a week**, but **no more than four times a week**.

Oily Fish<br>**Eat at least two portions a week, but no more than four**		
• Anchovies	• Sprats	• Kippers
• Herring	• Trout	• Pilchards
• Mackerel	• Whitebait	• Sardines
• Salmon	• Eel	• Swordfish

For peer review only

• Tuna (fresh not tinned)

Supplements

There are a range of supplements you can purchase that have been shown to have some effect on pain and inflammation caused by arthritis, these include **Fish Oils** (not to be confused with fish liver oil), **Glucosamine** (check with your GP before taking if you have diabetes), **Vitamin D, Calcium** and **Iron**.

Supplements are a good way to boost your intake of these beneficial substances but they should not be used in place of a healthy diet.

5.Holistic Medicine

Many people who have Arthritis find benefit in using some forms of complimentary or alternative medicine alongside their usual medication.

It is important that you **always discuss any complementary or alternative medicine with your GP** or the Rheumatology team before embarking on a treatment.

There are many alternatives to main stream medicine that may be of benefit to people with arthritis, these include:

- Acupuncture
- The Alexander technique
- Aromatherapy
- Copper bracelets
- Herbal medicine
- Massage
- Relaxation and hypnosis

Holistic medicine incorporating techniques such as these are generally safe but ensure that your therapist is legally registered before starting any treatment.

For peer review only

There is a lot more information on these general techniques and treatments on the Arthritis Research UK website (http://www.arthritisresearchuk.org/).

This booklet includes information written and published by Arthritis Research UK.

Additional information can be obtained from the Arthritis Research UK website (http://www.arthritisresearchuk.org/).

Information was also reproduced with the kind permission of Sarah Bradley and Kirsty Bancroft of Poole NHS Trust, and Christina Macleod of Hampshire Hospitals NHS Trust.

Notes:

For peer review only

Appendix 2

Self-Management Package: Arthritis Research UK Osteoarthritis Information Booklet *(see separate pdf as large file)*

For peer review only

Appendix 2

Self-Management Package: Facilitators and Barriers to Engaging with Self-Management Principles

1. Participant's ID number: _ _ - _ _ - _ _ _ _

2. My general exercise goal is: ....

....

....

....

3. My specific exercise goal – <u>What</u> am I going to do? ....

....

....

....

4. My Confidence

How confident am I that **I <u>will</u> achieve** my specific exercise goal? Please <u>circle</u> the appropriate number below.

Not at all confident 1 2 3 4 5 6 7 8 9 10 **Extremely confident**

5. My Commitment

How committed am I to **achieving** my specific exercise goal? Please <u>circle</u> the appropriate number below.

Not at all committed 1 2 3 4 5 6 7 8 9 10 **Extremely committed**

6. My Exercise Action Plan

It is important to measure and record your progress, so that you can see when you are succeeding as well as to work out what you can change if your plan is not working.

Where am I going to do the exercises? ....

When am I going to do the exercises? ....

**Patient**	**Practitioner**
I will do the exercises – my Specific Exercise Goal – and record my progress in my Exercise Diary and bring the Exercise Diary to my next consultation.	I will discuss with you your exercises (Specific Exercise Goal) and your Exercise Diary and clarify any questions you have.
**Signature:**	**Signature:** ....
**Date:**	**Date:** ....

peer review only

Barriers and Facilitators to doing Exercise

Unhelpful Things/Barriers	Helpful Things/Facilitators
**Places and things:** Is there anything about the things around me or the places I am in that makes it difficult to do the exercises – my Specific Exercise Goal? What **can I do** to change this?	**Places and things:** Is there anything about the things around me or the places I am in that makes it easier to do the exercises – my Specific Exercise Goal? What **can I do** to use these helpful things?
**People:** Are there any people I spend time with who make it difficult to do the exercises – my Specific Exercise Goal? What **can I do** to change this?	**People:** Are there any people I spend time with who make it easier to do the exercises – my Specific Exercise Goal? What **can I do** to ask them to help me?
**Thoughts and feelings:** Is there anything that I am thinking and/or feeling that makes it difficult to do the exercises – my Specific Exercise Goal? What **can I do** to change this?	**Thoughts and feelings:** Is there anything that I am thinking and/or feeling that makes it easier to do the exercises – my Specific Exercise Goal? What **can I do** to encourage these thoughts and feelings?

For peer review only

Appendix 2

Self-Management Package: Exercise Diary

Participant's ID number: _ _ - _ _ - _ _ _ _

Date of Occupational therapy/Physiotherapy Appointment when given the exercises: Date: _ _/_ _/_ _ _ _ (day/month/year)

Please do your hand exercises at least 3 times a week for at least 20 minutes each time. Please record how many times you did your exercise programme each day. If you didn't do your exercises please write 0

**Week**	**You can record notes or dates in this column, however you like, to help you keep track of which week you are in.**	**How many times did you do your exercises today?** Write **0** if not at all.						
		**Day 1**	**Day 2**	**Day 3**	**Day 4**	**Day 5**	**Day 6**	**Day 7**
**Week 1**								
**Week 2**								
**Week 3**								
**Week 4**								
**Week 5**								
**Week 6**								
**Week 7**								
**Week 8**								
**Week 9**								
**Week 10**								
**Week 11**								
**Week 12**								

iew only

For peer review only

Appendix 3

Verum Splint Decision Protocol

PURPOSE:

This OTTER splint decision guideline is to be used for supporting your decision to issue the verum splint for those patients allocated to the second arm in the OTTER trial.

INSTRUCTIONS:

1. **The option for the OTTER trial is to provide either**

- PROCOOL THUMB CMC RESTRICTION SPLINT
  Code: PTRS/(with the remainder of the code relating to the size/ laterality).

OR

- Orfilight 2.5mm 3/32" micro perforated (beige) trouser leg splint (made from a pre-cut template which will be provided)

Trial participants will wear this option for 12 weeks.

2. Splint decision clinical guidelines

We recommend that the choice of the verum splint is made in collaboration and agreement with the trial participant. It is important to ensure that the trial participants know and see that they do have a choice of splint to wear for 12 weeks.

We recommend that the Pro-cool thumb splint is issued when:

a) there is a generalised ache that interferes with function
b) there is mild CMCJ instability
c) a more rigid splint is impractical

We recommend that a tailor made thermoplastic “trousers splint” is made when

a) mechanical joint pain is a consistent feature, suggesting instability
b) There are high demands placed on the thumb, either in work or leisure

3 Splint prescription guidelines

It is recommended that the participant is informed that the splint is worn during ADls that aggravate their pain. As a guideline patients should be aiming to wear their splints for a minimum of 6 hours a day, during waking hours only (<u>not</u> to be worn over- night). This time

For peer review only

can be made up of individual splint wear periods and interspersed with active hand use. It is not expected that patients wear their splint for 6 hours at a time or in one go.

Appendix 4

Splint Wear Guide for Participants (Verum and Placebo Groups)

OTTER II Patient Splint Wear Guidelines

As part of the OTTER Trial you have been given a thumb splint by your therapist. It is important that you wear this splint over the next 12 weeks.

We ask that you wear your splint:

- During all your daily tasks that cause you thumb pain
- For a minimum of 6 hours a day
- During the day only and do not wear overnight or when asleep during the night.

Thank you for following these guidelines.
Please do not hesitate to contact your therapist if you have any questions about how or when to wear your trial splint.

For peer review only

Appendix 5

Facilitators and Barriers to Engaging with Splint Wear

---

1. Participant's ID number: _ _ - _ _ - _ _ _ _

---

2. My general splint wearing goal is:

....

....

....

---

3. My specific splint wearing goal – <u>What</u> am I going to do?

....

....

....

....

---

4. My Confidence

How confident am I that **I will achieve** my specific splint wearing goal? Please circle the appropriate number below.

Not at all confident 1 2 3 4 5 6 7 8 9 10 **Extremely confident**

---

5. My Commitment

How committed am I to **achieving** my specific splint wearing goal? Please circle the appropriate number below.

Not at all committed 1 2 3 4 5 6 7 8 9 10 **Extremely committed**

---

6. My Splint Wearing Action Plan

For peer review only

It is important to measure and record your progress, so that you can see when you are succeeding as well as to work out what you can change if your plan is not working.

Where am I going to wear the splint? ....

When am I going to wear the splint? ....

Patient

I will wear my splint – my Specific Splint Wearing Goal – and record my progress using my Splint Wearing Diary, and bring the Splint Wearing Diary to my next consultation.

Signature: ....

Date: ....

Practitioner

I will discuss with you your splint wearing regime (Specific Splint Wearing Goal) and your Splint Wearing Diary and clarify any questions you have.

Signature: ....

Date: ....

For peer review only

For peer review only

Barriers and Facilitators to Wearing the Splint

Unhelpful Things/Barriers	Helpful Things/Facilitators
**Places and things:** Is there anything about the things around me or the places I am in that makes it difficult to wear the splint – my Specific Splint Wearing Goal? What **can I do** to change this?	**Places and things:** Is there anything about the things around me or the places I am in that makes it easier to wear the splint – my Specific Splint Wearing Goal? What **can I do** to use these helpful things?
**People:** Are there any people I spend time with who make it difficult to wear the splint – my Specific Splint Wearing Goal? What **can I do** to change this?	**People:** Are there any people I spend time with who make it easier to wear the splint – my Specific Splint Wearing Goal? What **can I do** to ask them to help me?
**Thoughts and feelings:** Is there anything that I am thinking and/or feeling that makes it difficult to wear the splint – my Specific Splint Wearing Goal? What **can I do** to change this?	**Thoughts and feelings:** Is there anything that I am thinking and/or feeling that makes it easier to wear the splint – my Specific Splint Wearing Goal? What **can I do** to encourage these thoughts and feelings?

For peer review only

Appendix 6

Splint Wear Diary

Participant's ID number: _ _ - _ _ - _ _ _ _

Date of Occupational therapy/Physiotherapy Appointment when given the splint. Date: _ _/_ _/_ _ _ _ (day/month/year)

Please wear your splint as explained in the **OTTER Patient Splint Wear Guidelines**

Please record how many hours you wore your splint each day. If you didn't wear your splint please write **0**

Week	You can record notes or dates in this column, however you like, to help you keep track of which week you are in.	How many hours did you wear your splint today? Write **0** if not at all.						
		Day 1	Day 2	Day 3	Day 4	Day 5	Day 6	Day 7
Week 1								
Week 2								
Week 3								
Week 4								
Week 5								
Week 6								
Week 7								
Week 8								
Week 9								
Week 10								
Week 11								
Week 12								

iew only

Appendix 7

Placebo Splint Decision Protocol

PURPOSE:

This splint decision protocol is to be used for supporting your decision to issue one of the two DMO splints for those patients allocated to the third arm in the OTTER trial.

INSTRUCTIONS:

1. The option for the OTTER trial is to provide either

1 A Thumb Sleeve Lite DMO splint with cut out thumb sections and a wrist strap - beige

OR

2 A Thumb Sleeve DMO splint with a solid thumb component and a wrist strap - black

Trial participants will be asked to wear this option for 12 weeks.

2. Splint decision clinical guidelines

We recommend that the choice of splint is made in collaboration with the trial participant. Please refer to your training on delivering this dynamic splint option to maintain outcome expectancy.

It is important that participants know that they do have a choice of splint to wear for 12 weeks and we encourage that the two DMO options are shown to them to consider.

We recommend that patients agree one type of splint design to use in collaboration with their therapist. We suggest that you agree with the participant to provide the splint design that they feel fits more comfortably, the one that appears the most appealing and the one which they would prefer to wear.

3. Splint prescription guidelines

It is recommended that the participant is informed that the splint is worn during ADls that aggravate their pain. As a guideline patients should be aiming to wear their splints for a minimum of 6 hours a day, during waking hours only (not to be worn over- night). This time can be made up of individual

For peer review only

splint wear periods and interspersed with active hand use. It is not expected that patients wear their splint for 6 hours at a time or in one go.

4. Fit

Both DMO splint designs should be fitted such that the wrist strap is proximal to the ulnar styloid and does not provide any support or pressure around the wrist joint.

For peer review only

Appendix 8

Data and Safety Monitoring Committee Members

Anisur Rahman (Chair), Department of Rheumatology, University College London, London, UK

Ross Wilkie, Arthritis Research UK Primary Care Centre, Keele University, UK

Ranjit Lall, Warwick Clinical Trials Unit, University of Warwick, Coventry, UK

For peer review only

Appendix 9

Trial Steering Committee Members

Sarah Dean (Chair), University of Exeter Medical School, University of Exeter, Exeter, UK

Cathy Ball, Kennedy Institute of Rheumatology, University of Oxford, Oxford, UK

Lindsay Bearne, Faculty of Life Sciences & Medicine, King's College London, London, UK

Elaine Dennison, MRC Lifecourse Epidemiology Unit, University of Southampton, Southampton, UK

Corinne Hutt Greenyer, School of Health Sciences, University of Southampton, Southampton, UK

Jo Adams, School of Health Sciences, University of Southampton, Southampton, UK

For peer review only

Arthritis Research UK

Condition
Osteoarthritis

Osteoarthritis

This booklet provides information and answers to your questions about this condition.

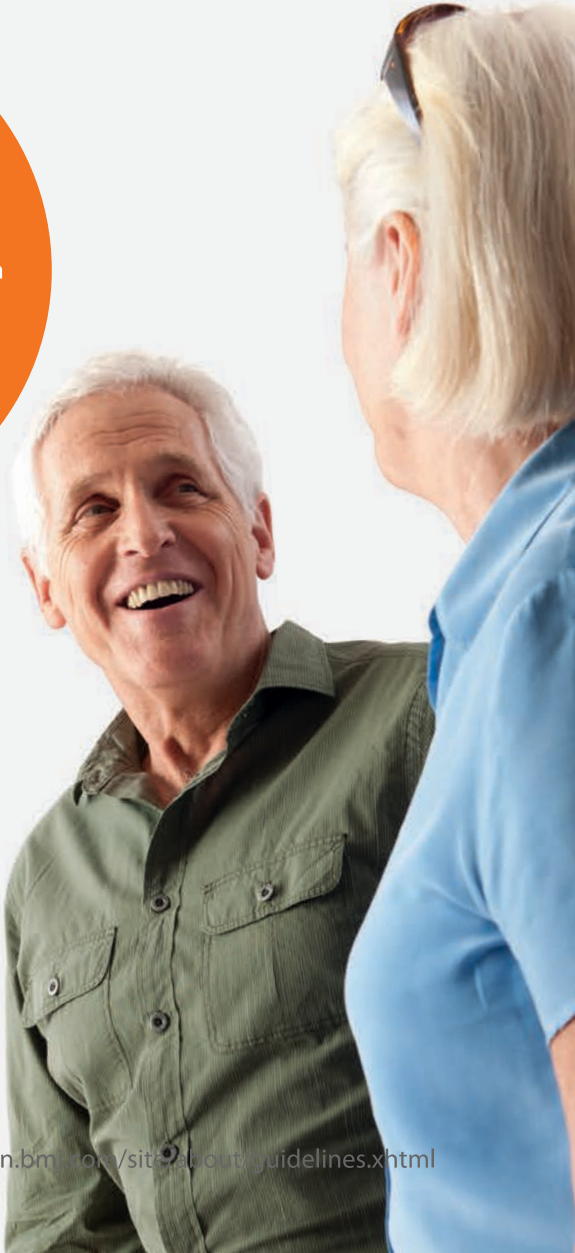

Arthritis Research UK produce and print our booklets entirely from charitable donations.

What is osteoarthritis?

Osteoarthritis is by far the most common form of joint disease. It causes pain and stiffness in the joints and affects approximately 8 million people in the UK. In this booklet we'll explain how osteoarthritis develops, what causes it and how it can be treated. We'll also give some hints and tips to help you manage your arthritis and suggest where you can find out more.

At the back of this booklet you'll find a brief glossary of medical words – we've underlined these when they're first used.

www.arthritisresearchuk.org

For peer review only - http://bmjopen.bmj.com/site/about/guidelines.xhtml

Arthritis Research UK
Osteoarthritis

What's inside?

2 Osteoarthritis at a glance
4 How does a normal joint work?
4 What is osteoarthritis?
6 What are the symptoms of osteoarthritis?
7 What causes osteoarthritis?
8 Which joints are affected?
– The knee
– The hip
– The hand
– The back and neck
– The foot
11 What is the outlook?
12 What are the possible complications?
12 How is osteoarthritis diagnosed?
– What tests are there?
14 What can I do to help myself?
– Exercise
– Weight management
– Tablets and creams
– Reducing the strain on your joints
– Complementary medicine
20 What treatments are there for osteoarthritis?
– Capsaicin cream
– Drugs
– Steroid injections
– Transcutaneous electrical nerve stimulation (TENS)
– Surgery
22 Self-help and daily living
23 Research and new developments
24 Glossary
26 Where can I find out more?
32 We're here to help

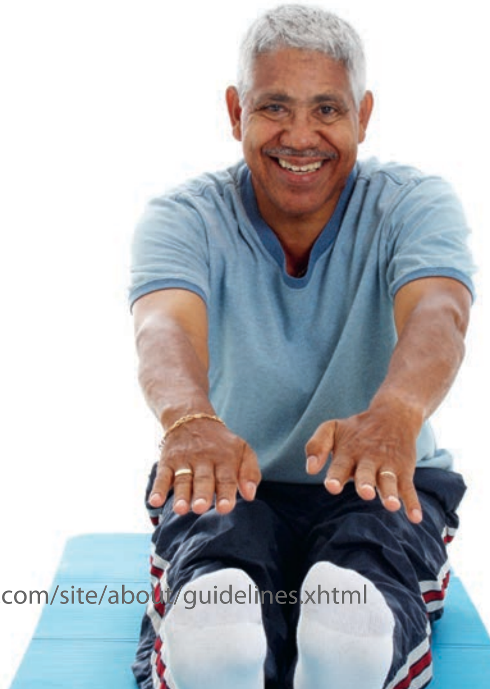

For peer review only - http://bmjopen.bmj.com/site/about/guidelines.xhtml

At a glance
Osteoarthritis

Osteoarthritis is a condition that affects the joints, causing pain and stiffness.

What is osteoarthritis?

Osteoarthritis is a condition that affects the joints, causing pain and stiffness. It's by far the most common form of joint disease, affecting people all over the world and approximately 8 million people in the UK.

What are the symptoms?

Symptoms of osteoarthritis can include:

- pain
- stiffness
- a grating or grinding sensation (crepitus) when the joint moves
- swelling (either hard or soft)
- not being able to use the affected joint normally, which can make it difficult to do certain activities (for example climbing stairs).

Who gets it?

Almost anyone can get osteoarthritis but it's most likely if:

- you're in your late 40s or older
- you're a woman
- your parents have had osteoarthritis
- you're overweight
- you've had a previous joint injury
- you have a physically demanding job where you make repetitive movements
- your joints have been damaged by another disease, for example gout or rheumatoid arthritis.

For peer review only

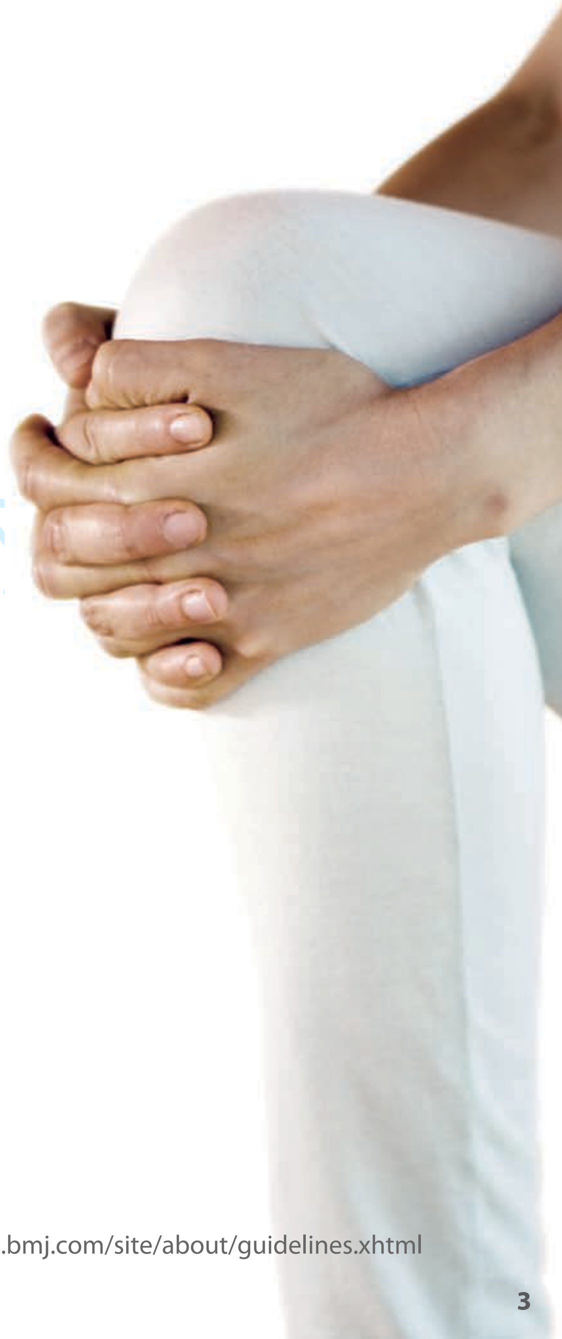

What can I do to help myself?

There are several ways you can help yourself, including:

- exercising regularly (both muscle-strengthening and general aerobic exercise)
- reducing stress on the affected joint (for example by pacing activities, using a walking stick or wearing suitable footwear)
- losing weight if you're overweight
- using over-the-counter painkillers such as paracetamol or low-dose ibuprofen, or pain-relieving creams, gels or sprays.

What treatments are there?

If you still have pain after trying self-help measures, your doctor may recommend the following:

- advice from a physiotherapist about exercise plans and gradually increasing your aerobic exercise
- capsaicin cream
- stronger painkillers, for example tramadol
- steroid injections into the painful joint
- surgery, including joint replacement.

For peer review only - http://bmjopen.bmj.com/site/about/guidelines.xhtml

How does a normal joint work?

A joint is where two or more bones meet (see Figure 1). The joint allows the bones to move freely but within controlled limits. The knees have additional rings of cartilage between the bones. These are called menisci – they act a bit like shock absorbers to spread the load more evenly across the joint.

What is osteoarthritis?

Osteoarthritis is a disease that affects your joints. The surfaces within your joints become damaged so the joint doesn't move as smoothly as it should (see Figures 2 and 3). The condition is sometimes called arthrosis or osteoarthrosis. Older terms are degenerative joint disease or wear and tear.

When a joint develops osteoarthritis, some of the cartilage covering the ends of the bones gradually roughens and becomes thin, and the bone underneath thickens. All the tissues within the joint become more active than normal – as if your body is trying to repair the damage:

- The bone at the edge of the joint grows outwards, forming bony spurs called osteophytes.
- The synovium may thicken and produce extra fluid, which then causes the joint to swell.
- The capsule and ligaments slowly thicken and contract as if they were trying to stabilise the joint.

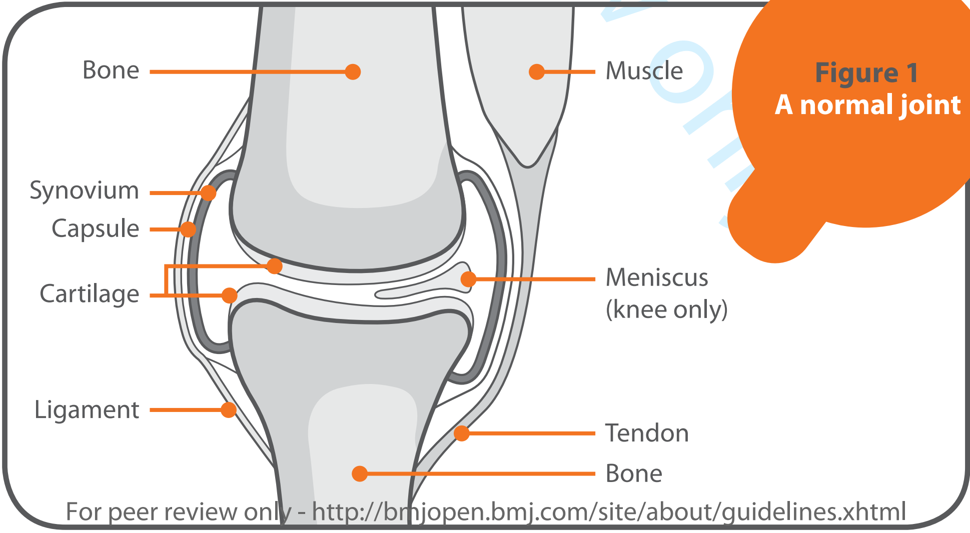

Figure 1
A normal joint

For peer review only - http://bmjopen.bmj.com/site/about/guidelines.xhtml

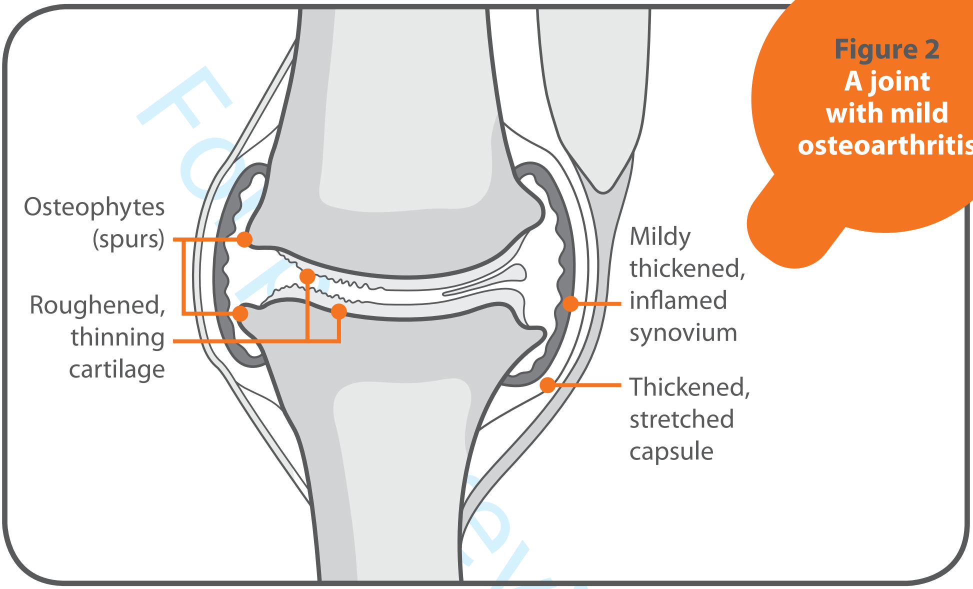

Figure 2
A joint with mild osteoarthritis

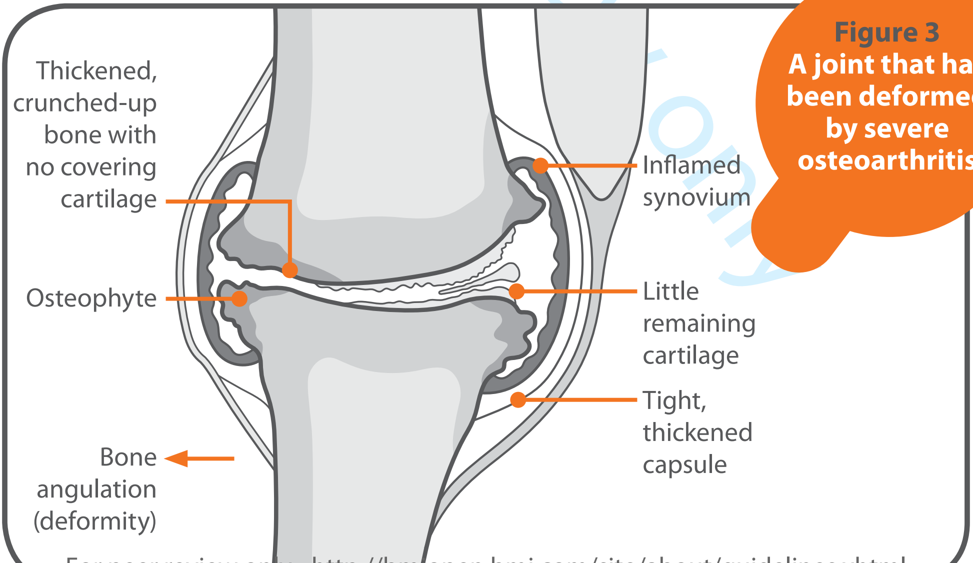

Figure 3
A joint that has been deformed by severe osteoarthritis

For peer review only - http://bmjopen.bmj.com/site/about/guidelines.xhtml

Sometimes your body's repairs are quite successful and the changes inside the joint won't cause pain or problems. In severe osteoarthritis, the cartilage can become so thin that it no longer covers the ends of your bones. The bones start to rub against each other and eventually start to wear away. The loss of cartilage, the wearing of bone and the bony spurs can alter the shape of the joint, forcing the bones out of their normal position.

What are the symptoms of osteoarthritis?

The main symptoms of osteoarthritis are pain and sometimes stiffness in the affected joints. The pain tends to be worse when you move the joint or at the end of the day, and it may make it difficult to get to sleep. Your joints may feel stiff after rest, but this usually wears off after a minute or two as you get moving.

If you have severe osteoarthritis, you may feel pain more often. The joint may not move as freely or as far as normal, and it may creak or crunch as you move. Sometimes it may give way because your muscles have weakened or the joint structure has become less stable, although exercises to strengthen your muscles can help to prevent this.

You may notice that the affected joint looks swollen. The swelling may be hard (caused by osteophytes) or soft (caused by synovial thickening and extra fluid in the joint), and the muscles around the joint may look thin or wasted.

Figure 4 Risk factors for osteoarthritis

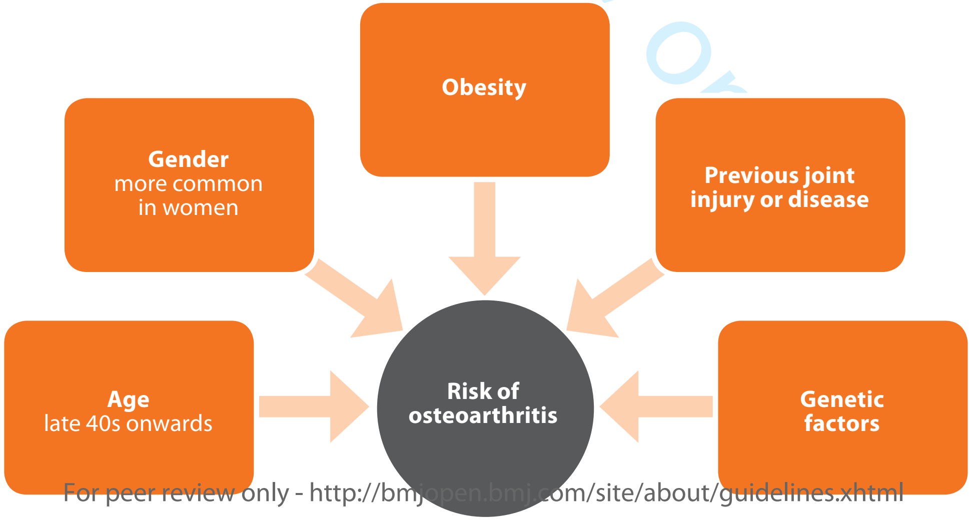

For peer review only - http://bmjopen.bmj.com/site/about/guidelines.xhtml

Symptoms often vary for no obvious reason – you'll probably have good and bad spells. Some people find that changes in the weather make the pain worse, especially damp weather along with falling atmospheric pressure. Others find the pain varies depending on how active they've been.

In more severe cases, the pain may be constant. It may prevent you from sleeping and cause difficulties in your daily activities; for example, osteoarthritis in the knee or hip can make it difficult to climb stairs or get up from a chair.

What causes osteoarthritis?

There are many factors that can increase your risk of osteoarthritis, and it's often a combination of these that leads to the condition (see Figure 4):

- **Age** – Osteoarthritis usually starts from the late 40s onwards. We don't fully understand why it's more common in older people, but it might be due to factors like the muscles weakening and the body being less able to heal itself, or gradual wearing out of the joint with time.
- **Gender** – For most joints, especially the knees and hands, osteoarthritis is more common and more severe in women.
- **Obesity** – Being overweight is an important factor in causing osteoarthritis, especially in the knee. It also increases the chances of osteoarthritis becoming progressively worse.

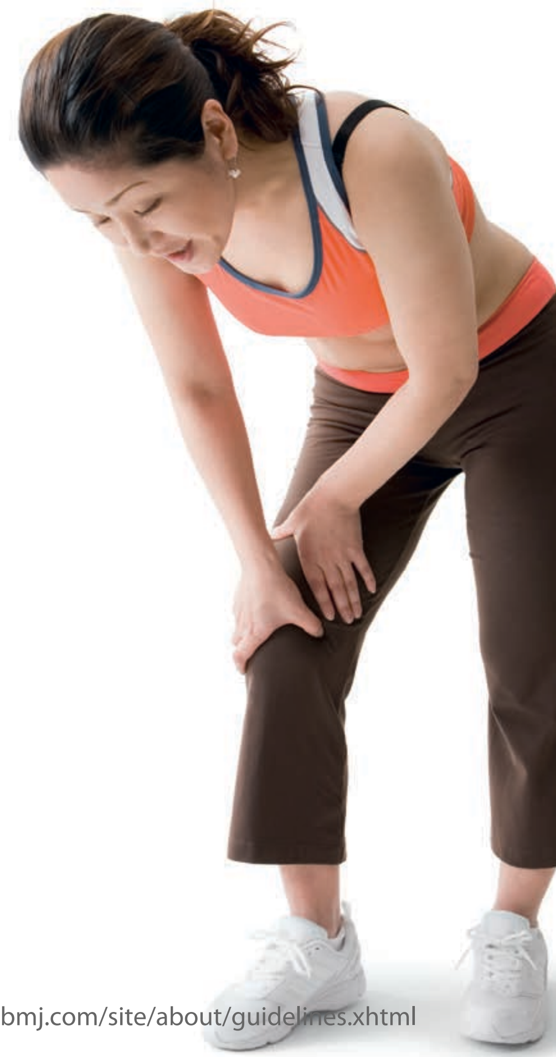

For peer review only - http://bmjopen.bmj.com/site/about/guidelines.xhtml

- **Joint injury** – A major injury or operation on a joint may lead to osteoarthritis in that joint later in life. Normal activity and exercise don't cause osteoarthritis, but very hard, repetitive activity or physically demanding jobs can increase your risk.
- **Joint abnormalities** – If you were born with abnormalities or developed them in childhood, it can lead to earlier and more severe osteoarthritis than usual. Perthes' disease of the hips is an example.
- **Genetic factors** – Nodal osteoarthritis, which particularly affects the hands of middle-aged women, runs strongly in families, although it's not yet clear which genes are involved. And some rare forms of osteoarthritis which start at an earlier age are linked with genes that affect collagen (an essential part of cartilage). Genetic factors play a smaller, but still significant, part in osteoarthritis of the hip and knee.
- **Other types of joint disease** – Sometimes osteoarthritis is a result of damage from a different kind of joint disease, such as rheumatoid arthritis or gout.

Which joints are affected?

Almost any joint can develop osteoarthritis, especially if it's been badly injured, but it most often affects the knees, hips, hands, spine and big toes (see Figure 5):

The knee

Osteoarthritis of the knee is very common. This is probably because your knee has to take extreme stresses, twists and turns.

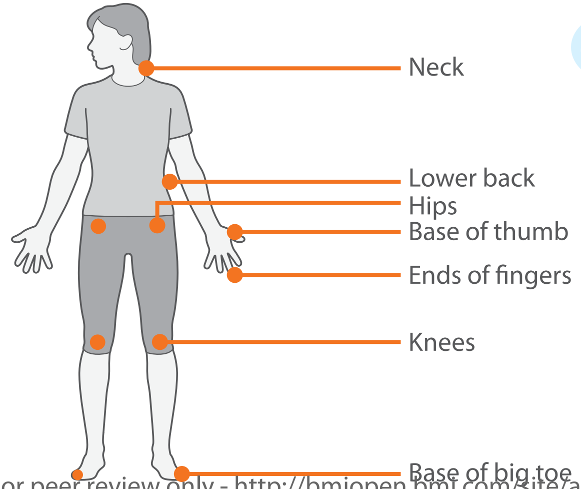

Figure 5
The joints most often affected by osteoarthritis

For peer review only - http://bmjopen.bmj.com/site/about/guidelines.xhtml

Any joint can be affected by osteoarthritis.

Osteoarthritis can affect the main surfaces of the knee joint and also the cartilage underneath your kneecap (patella).

You're most likely to feel pain at the front and sides of your knee. If the osteoarthritis is severe, your knees may become bent and bowed. Your knee joint may also become unstable so that it gives way when you put weight on it. This is usually because of muscle weakness in the thigh but sometimes because of damage to the ligaments.

Osteoarthritis of the knee is twice as common in women as in men and it usually affects both knees. It causes most problems from the late 50s onwards. A number of factors can increase the risk of osteoarthritis of the knee, for example:

- being overweight
- having nodal osteoarthritis (particularly in women)
- a previous sporting injury (such as a torn meniscus or ligament)
- an operation to remove torn cartilage (meniscectomy).

See Arthritis Research UK booklet *Osteoarthritis of the knee.*

The hip

Osteoarthritis of the hip is also very common and can affect either one or both hips. The pain is most likely to be deep at the front of your groin, but you may also feel pain at the side and front of your thigh, in your buttock or down to your knee (this is called referred pain).

If you have severe hip osteoarthritis, you may find the affected leg seems a little shorter than the other because of the bone on either side of the joint being crunched up.

Men and women are equally likely to develop hip osteoarthritis, and it usually starts from the late 40s onwards. The risk may be greater if you had hip problems at birth (congenital dislocation) or abnormal hip development in childhood, such as Perthes' disease. Physical work such as farming may also increase the risk; however, there's often no obvious cause.

The hand

Osteoarthritis of the hands usually occurs as part of nodal osteoarthritis. This mainly affects women and often starts in your 40s or 50s, around the time of the menopause. It usually affects the base of your thumb

For peer review only - http://bmjopen.bmj.com/site/about/guidelines.xhtml

and the joints at the ends of your fingers, although other finger joints can also be affected. At times these joints become swollen and tender, especially when the condition first appears.

Over several years, firm knobbly swellings form on the finger joints. These are caused by osteophytes and are known as Heberden's nodes when they're at the end joints of your fingers (see Figure 6) or Bouchard's nodes when they're at the mid-finger joints. Once the nodes are fully formed, the pain and tenderness often improve. Although the fingers are knobbly and sometimes slightly bent, they usually still work well. Arthritis at the base of your thumb may cause longer-lasting problems.

Having nodal osteoarthritis in middle age means you're more likely to develop osteoarthritis of the knee, and possibly other joints, as you get older. Nodal osteoarthritis tends to run in families much more than other forms of osteoarthritis and it's especially likely to be passed from mother to daughter. It's not yet known which genes are involved so it's not possible to test for this.

The back and neck

Changes that affect the bones of your spine and the discs between the bones are often called spondylosis, but they're very similar to the changes caused by osteoarthritis in other joints. X-rays show that spondylosis is extremely common, but it's not the most common cause of back or neck pain and often doesn't cause any problems at all.

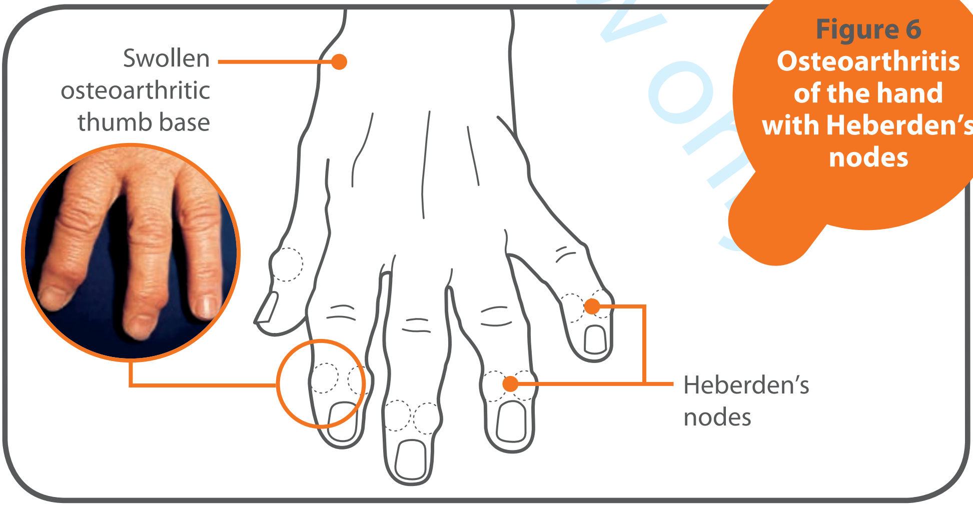

Figure 6 Osteoarthritis of the hand with Heberden's nodes

Photography used with kind permission of Elsevier. © Elsevier 2011. Taken from *Rheumatology*, fifth edition. Edited by Marc C Hochberg, Alan J Silman, Josef S Smolen, Michael E Weinblatt and Michael H Weisman.

For peer review only - http://bmjopen.bmj.com/site/about/guidelines.xhtml

See Arthritis Research UK booklets
Back pain; Neck pain.

The foot

Osteoarthritis of the foot generally affects the joint at the base of your big toe. Eventually your toe may become stiff (hallux rigidus), which can make it difficult and painful to walk, or bent (hallux valgus), which can lead to painful bunions (see Figure 7).

Osteoarthritis of the mid-foot is also quite common, especially in older people, and may cause an obvious bony swelling (osteophyte) on the top of your mid-foot. Ankle osteoarthritis is least common and may cause your heel to move to an unusual angle.

See Arthritis Research UK booklets
Feet, footwear and arthritis.

What is the outlook?

It's impossible to predict how osteoarthritis will develop for any one person. It can sometimes develop over just a year or two and cause a lot of damage to a joint, which may then cause some deformity or disability. But more often osteoarthritis is a slow process that develops over many years and results in fairly small changes in just part of the joint. This doesn't mean it won't be painful, but it's less likely to cause severe deformity or disability. Sometimes the condition reaches a peak a few years after the symptoms start and then remains the same, or it may even improve.

Unlike rheumatoid arthritis, osteoarthritis doesn't affect other parts of your body – it's purely a joint disease. Also, because there's very little, if any, inflammation in osteoarthritic joints, the condition doesn't

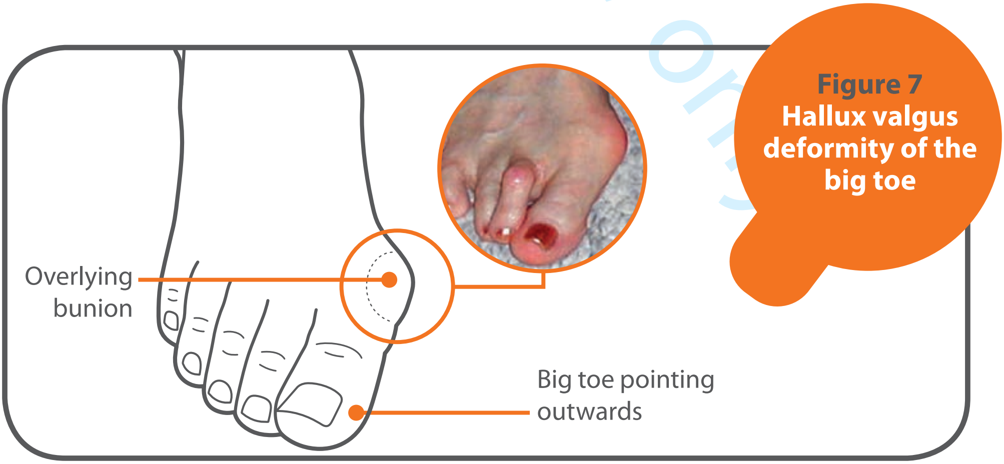

Figure 7 Hallux valgus deformity of the big toe

Photography used with kind permission of Elsevier. © Elsevier 2011. Taken from *Rheumatology*, fifth edition. Edited by Marc C Hochberg, Alan J Silman, Josef S Smolen, Michael E Weinblatt and Michael H Weisman.

For peer review only - http://bmjopen.bmj.com/site/about/guidelines.xhtml

make you feverish or unwell. However, some people with osteoarthritis will develop other illnesses purely by chance.

What are the possible complications?

The changes in cartilage that occur with osteoarthritis can encourage crystals to form within the joint. Gout is a common type of inflammatory arthritis, which is caused by high levels of uric acid that lead to sodium urate crystals forming in and around joints. If you have both osteoarthritis and a high serum uric acid, you're at an increased risk of developing gout. The base of the big toe is a very common site for a painful attack of gout, and this is partly because this joint is the most common joint in the foot to be affected by osteoarthritis.

See Arthritis Research UK booklet *Gout.*

Osteoarthritis doesn't lead to rheumatoid arthritis or other types of joint disease

Osteoarthritis can also encourage calcium pyrophosphate crystals to form in the cartilage. This is called calcification or chondrocalcinosis. It can happen in any joint, with or without osteoarthritis, but it's most likely to occur in a knee already affected by osteoarthritis, especially in older people. The crystals will show up in x-rays and they can also be seen under a microscope in samples of fluid taken from the joint.

Osteoarthritis tends to become more severe more quickly when there are calcium crystals present. Sometimes the crystals can shake loose from the cartilage, causing a sudden attack of very painful swelling called acute calcium pyrophosphate crystal arthritis (acute CPP crystal arthritis), which was sometimes previously called 'pseudogout'.

See Arthritis Research UK booklet *Calcium crystal diseases including acute CPP crystal arthritis (pseudogout) and acute calcific tendinitis.*

How is osteoarthritis diagnosed?

It's very important to get an accurate diagnosis if you think you have arthritis. There are many different types of arthritis and some need very different treatments. Osteoarthritis is usually diagnosed based on your symptoms and the physical signs that your doctor finds when examining your joints. The signs your doctor will be checking for are:

For peer review only - http://bmjopen.bmj.com/site/about/guidelines.xhtml

- tenderness over the joint
- creaking or grating of the joint (crepitus)
- bony swelling
- excess fluid
- restricted movement
- joint instability
- thinning of the muscles that support the joint.

What tests are there?

There's no blood test for osteoarthritis, although your doctor may suggest you have them to help rule out other types of arthritis.

X-rays are the most useful test to confirm osteoarthritis, although they often won't be needed. An x-ray may show changes such as bony spurs or narrowing of the space between the bones. They'll also show whether there are any calcium deposits within the joint.

However, x-rays can't really show how much pain or disability you're likely to have. Some people have a lot of pain from fairly minor joint damage, while others have little pain from more severe damage.

Rarely, a magnetic resonance imaging (MRI) scan of the knee can be helpful. This will show the soft tissues (for example cartilage, tendons, muscles) and changes in the bone that can't be seen on a standard x-ray. Its main use is to identify another joint or bone problem in someone whose symptoms aren't typical of osteoarthritis, for example, in a person with a torn knee meniscus that causes intermittent 'locking' of the knee.

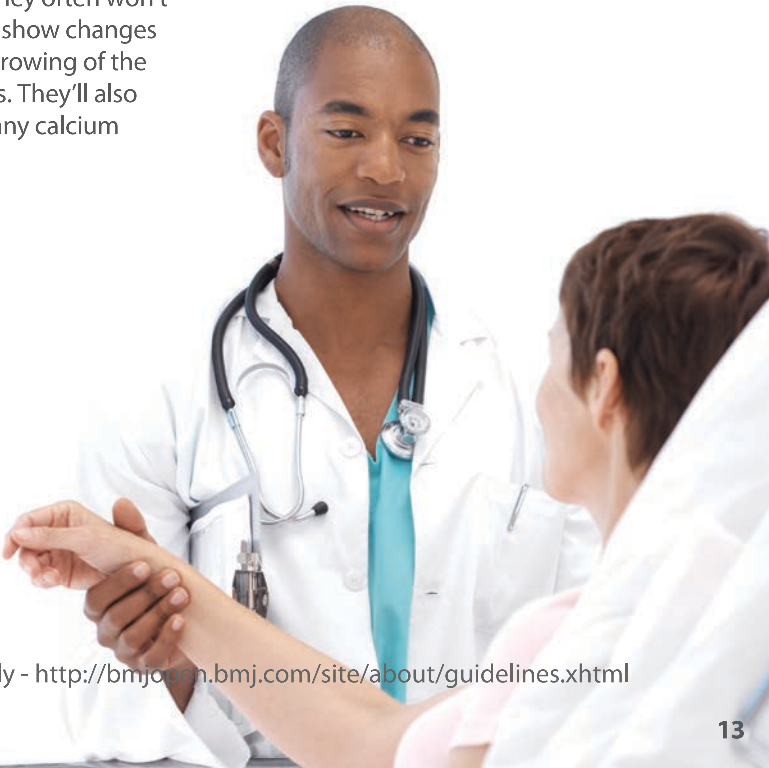

For peer review only - http://bmjopen.bmj.com/site/about/guidelines.xhtml

What can I do to help myself?

There's no cure for osteoarthritis as yet, but there's a lot that you can do to improve your symptoms. Self-help measures play a very important part in relieving the pain and stiffness, and reducing the chances of your arthritis becoming worse.

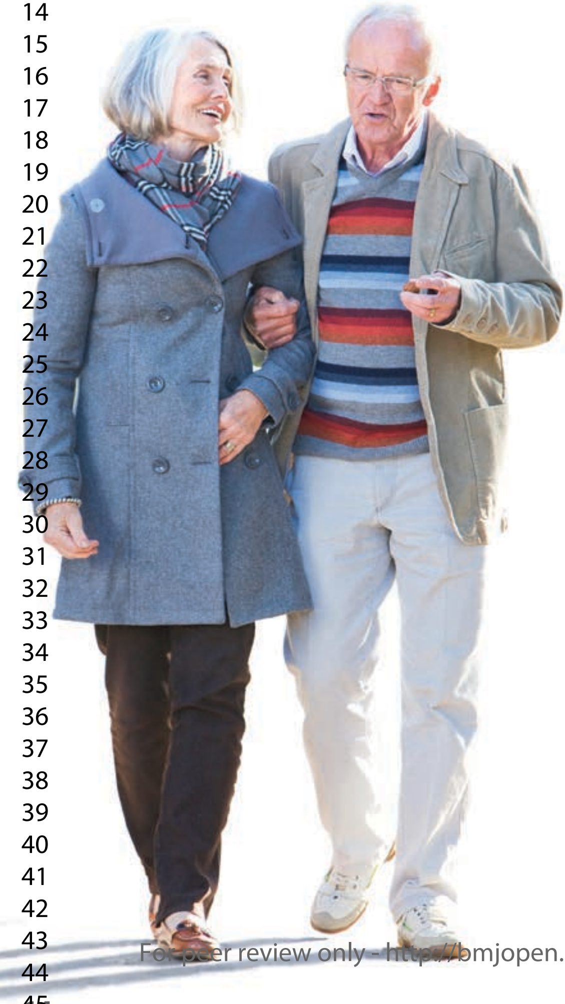

Exercise

It's very important to keep your joints moving. You'll need to find the right balance between rest and exercise – most people with osteoarthritis find that too much activity increases their pain while too little makes their joints stiffen up. Little and often is usually the best approach to exercise if you have osteoarthritis.

There are two main types of exercise that you'll need to do:

Strengthening exercises will improve the strength and tone of the muscles that control the affected joint. Osteoarthritis can weaken these muscles. This is particularly important for the thigh (quadriceps) muscles if you have osteoarthritis of the knee. Regular exercising of the muscles, such as straight-leg raises (see the pull-out section at the back of the booklet), helps to stabilise and protect the joint and has also been shown to reduce pain. It's also particularly helpful in preventing the knee giving way and reducing the chances of stumbling or falling.

Because knee and hip osteoarthritis may come to affect both sides of your body, and because both legs work as a unit when you walk, it's helpful to do strengthening exercises on the muscles on the other leg, even if that knee or hip isn't causing symptoms, and also to do hip exercises if you have knee osteoarthritis (and vice versa).

For peer review only - http://bmjopen.bmj.com/site/about/guidelines.xhtml

Arthritis Research UK
Osteoarthritis

Aerobic exercise is any exercise that increases your pulse rate and makes you a bit short of breath (for example a brisk walk, swimming or using an exercise bike). Regular aerobic exercise should help you sleep better, is good for your general health and well-being, and can also reduce pain by raising the levels of pain-relieving hormones called endorphins.

A physiotherapist can advise you on the best exercises for the type of osteoarthritis you have, but you'll need to build them into your daily routine to get the most from them. You can also talk to your GP about the Exercise on Prescription scheme that's available in some areas.

Swimming can be very good for osteoarthritis. Because the water supports the weight of your body, you won't be putting a lot of strain on your joints as you exercise. Your physiotherapist may also recommend special exercises in a hydrotherapy pool. This can help get muscles and joints working better and, because the water is warmer than in a typical swimming pool, it can be very soothing and relaxing.

T'ai chi is another type of exercise that has been shown to be helpful at reducing the pain from osteoarthritis. Many people find that regular t'ai chi also makes them feel better in other ways, for example, less stressed during the day and better able to sleep at night.

See Arthritis Research UK booklets *Hydrotherapy and arthritis; Keep moving; Physiotherapy and arthritis.*

Weight management

There's a great deal of evidence that being overweight increases the strain on your joints – especially your knees. Research shows that being overweight or obese not only increases your risk of developing osteoarthritis but also makes it more likely that your arthritis will get worse over time.

Because of the way the joints work, the force put through your knees when you walk, especially on stairs and slopes, can be several times your actual body weight. Losing even a small amount of weight can make a big difference to the strain on weight-bearing joints.

No special diet has been shown to help with osteoarthritis, but if you need to lose some weight we would recommend a balanced, reduced-calorie diet combined with regular exercise.

See Arthritis Research UK booklet *Diet and arthritis.*

Tablets and creams

There are a number of tablets and creams that can help osteoarthritis symptoms, and because they work in different ways you can combine different treatments if you need to. Your pharmacist can advise you and supply paracetamol, and some low-dose tablets and creams without a prescription.

For peer review only - http://bmjopen.bmj.com/site/about/guidelines.xhtml

Anti-inflammatory creams and gels are especially helpful for osteoarthritis of the knee or hand.

Painkillers (analgesics) and non-steroidal anti-inflammatory drugs (NSAIDs)

Painkillers (analgesics) often help with the pain and stiffness, although they don't affect the arthritis itself and won't repair damage to the joint. They're best used occasionally when the pain is very bad or when you're likely to be exercising. Paracetamol is usually the best and safest painkiller to try first, but make sure you take the right dose as most people take too little. You should try taking 1 g (usually two tablets) three or four times a day. It's best to take them before the pain becomes very bad but you shouldn't take them more often than every four hours.

Combined painkillers (for example co-codamol) contain paracetamol and a second codeine-like drug, and they may be helpful for more severe pain. They're stronger than paracetamol and are therefore more likely to cause side-effects such as constipation or dizziness.

Over-the counter non-steroidal anti-inflammatory drugs (NSAIDs), such as ibuprofen, can also help. You can use these for a short course of treatment (about 5–10 days), but if they've not helped within this time then they're unlikely to. If the pain returns when you stop taking the tablets, try another short course.

If you're already taking NSAID tablets, speak to your doctor about non-NSAID creams (for example capsaicin cream) to avoid taking too much of one type of drug.

! You shouldn't take ibuprofen or aspirin if you're pregnant, or if you have asthma, indigestion or a stomach (gastrointestinal) ulcer, until you've spoken with your doctor or pharmacist.

If you know you're going to be more active than usual, try taking a painkiller before you start to avoid increased pain later.

---

i **See Arthritis Research UK leaflets** *Non-steroidal anti-inflammatory drugs; Painkillers.*

Anti-inflammatory creams and gels

You can apply anti-inflammatory creams and gels directly onto painful joints three times a day. There's no need to rub them in – they absorb through the skin on their own. They're especially helpful for osteoarthritis of the knee or hand but not for deep joints such as the hip. They're extremely well tolerated as very little is absorbed into the bloodstream. If you have trouble taking tablets then anti-inflammatory creams are a particularly

For peer review only - http://bmjopen.bmj.com/site/about/guidelines.xhtml

good option to try. You can decide if they help your pain within the first few days of trying them.

Reducing the strain on your joints

Apart from keeping an eye on your weight, there are a number of other ways you can reduce the strain on your joints:

- Pace your activities through the day – don't tackle all the physical jobs at once. Break the harder jobs up into chunks and do something more gentle in between.
- Wear low-heeled shoes with soft, thick soles (trainers are ideal). Thicker soles will act as shock absorbers for your feet, knees, hips and back. High heels will alter the angle of your hips, knees and big toe joints and put additional strain on them.
- Use a walking stick to reduce the weight and stress on a painful hip or knee. A therapist or doctor can advise on the correct length and how to put your weight through the stick when you're putting weight on your affected joint.
- Use the handrail for support when climbing stairs – this is particularly important if you have osteoarthritis of the knee.
- Keep your joints moving – in particular, don't keep an osteoarthritic knee still in a bent position for too long as this will eventually affect the muscles.

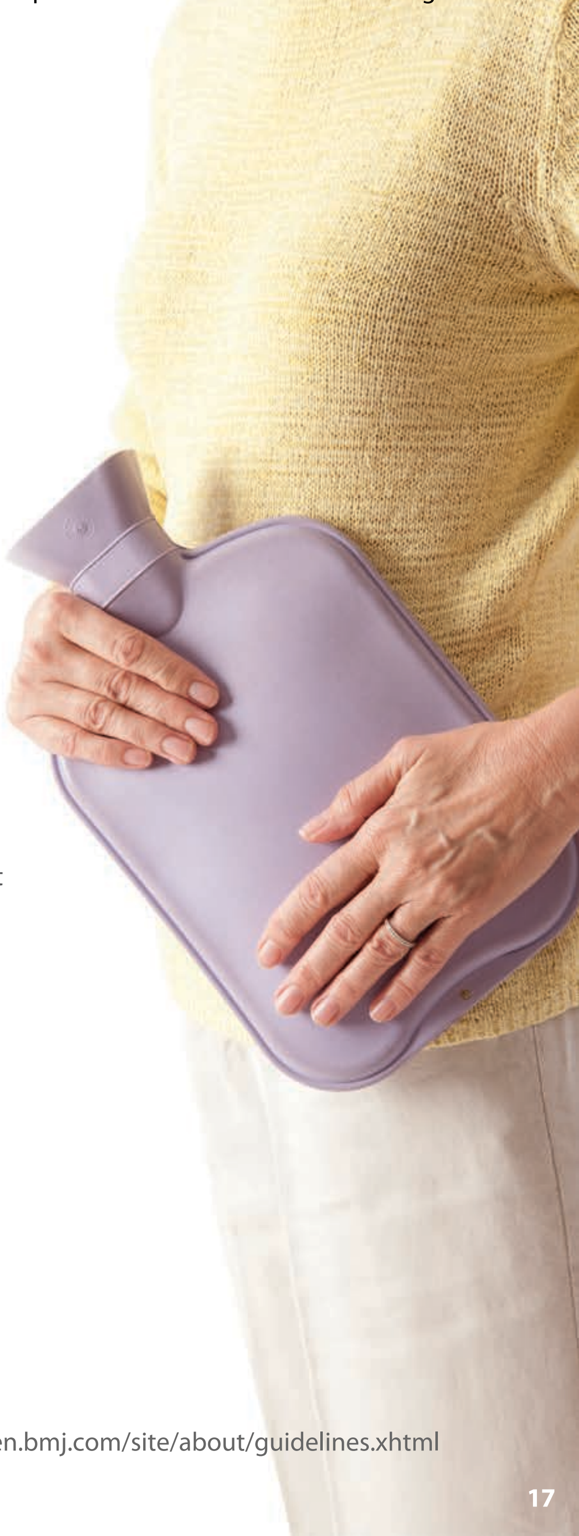

For peer review only - http://bmjopen.bmj.com/site/about/guidelines.xhtml

Reducing strain on your joints can help with osteoarthritis.

- Think about modifying your home, car or workplace to reduce unnecessary strain on your joints. An occupational therapist can advise you on how to protect your joints and on special equipment or gadgets that will make your daily tasks easier.
- Learn to relax your muscles and get the tension out of your body. A physiotherapist or occupational therapist can advise you on relaxation techniques.
- Sex can sometimes be painful, particularly for women with osteoarthritis of the hips. Trying a different position can often help.

See Arthritis Research UK booklets *Looking after your joints when you have arthritis; Occupational therapy and arthritis; Sex and arthritis.*

Applying warmth to a painful joint often relieves the pain and stiffness of osteoarthritis. Heat lamps are popular, but a hot-water bottle or reheatable pad are just as effective. This can be helpful if you have a flare-up of pain when you've done a bit too much. An ice pack can also help, but don't apply either ice or heat packs directly to your skin.

More evidence to support the use of knee braces for osteoarthritis is becoming available. There are several types that can help to stabilise the kneecap and make it move correctly. You can buy knee braces from sports shops and chemists, but you should speak to your doctor or physiotherapist first. They may also be able to provide braces or recommend the best ones for you.

Complementary medicine

There are many different complementary and herbal remedies that claim to help with arthritis, and some people do feel better when they use some complementary treatments. However, on the whole these treatments aren't recommended for use on the NHS because there's no clear evidence that they work. There's no scientific evidence, for example, that copper bracelets can help ease osteoarthritis-related pain.

Glucosamine and chondroitin

Many people try glucosamine and chondroitin tablets. These are compounds that are normally found in joint cartilage, and some studies suggest that taking supplements may improve the health of damaged cartilage. Other studies, however, don't show any benefit so we still

For peer review only - http://bmjopen.bmj.com/site/about/guidelines.xhtml

don't know for sure whether they work or not. Glucosamine and chondroitin, which are similar to each other, are available from your chemist or health food store. You'll need to take a dose of 1.5 g glucosamine sulphate a day, and you may need to take them for several weeks before you can tell whether they're making a difference.

Glucosamine hydrochloride doesn't appear to be effective, so always check that you're taking the sulphate variety.

Most brands of glucosamine are made from shellfish. If you're allergic to shellfish, make sure you use a vegetarian or shellfish-free variety. Glucosamine can affect the level of sugar in your blood, so if you have diabetes you should keep an eye on your blood sugar levels and see your doctor if they increase. You should also see your doctor for regular blood checks if you're taking the blood-thinning drug warfarin.

Homeopathy

Many people are interested in homeopathic remedies, and a number of different types are used for osteoarthritis; however, there's no conclusive scientific evidence that the remedies are effective.

Acupuncture

There's some research showing that acupuncture can sometimes provide relief from arthritis pain, although the effect may be short-lived, which means that you'll need repeat sessions. Other studies show no benefit from acupuncture.

Chiropractic and osteopathy

Manipulation by a chiropractor or osteopath can often help neck and back pain, although the use of manipulation for osteoarthritis in other joints is limited. If you do want to try it, make sure you choose a practitioner who is registered with the appropriate regulatory body.

See Arthritis Research UK booklet and special reports
Complementary and alternative medicine for arthritis; Complementary and alternative medicines for the treatment of rheumatoid arthritis, osteoarthritis and fibromyalgia; Practitioner-based complementary and alternative therapies for the treatment of rheumatoid arthritis, osteoarthritis, fibromyalgia, and low back pain.

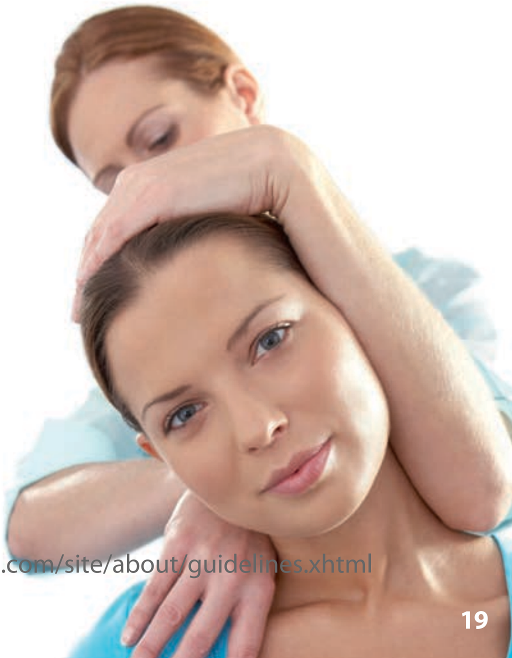

For peer review only - http://bmjopen.bmj.com/site/about/guidelines.xhtml

What treatments are there for osteoarthritis?

Many people find that self-help measures, such as those listed above, are enough to help them manage their symptoms, but your healthcare team will be able to suggest other treatments if you need them.

Capsaicin cream

Capsaicin cream is made from the pepper plant (capsicum) and is an effective and very well-tolerated painkiller. It needs to be applied regularly three times each day to be effective and, like NSAID creams and gels, it's particularly useful for knee and hand osteoarthritis. It's only available on prescription.

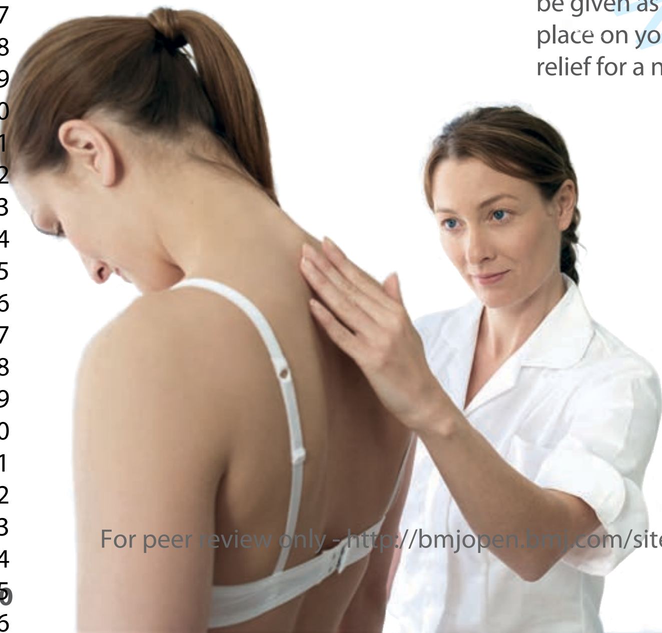

Most people feel a warming or burning sensation when they first use capsaicin, but this generally wears off after several days. The pain-relieving effect starts after several days of regular use and you should try it for at least two weeks before deciding if it has helped.

Drugs

Painkillers

If you have severe pain and other medications aren't giving enough relief, your doctor may recommend stronger painkillers (or opioids) such as tramadol, nefopam or meptazinol. Stronger painkillers are more likely to have side-effects – especially nausea, dizziness and confusion – so you'll need to see your doctor regularly and report any problems you have with these drugs.

Some opioids (for example fentanyl) can be given as a plaster patch which you place on your skin – these can give pain relief for a number of days.

For peer review only - http://bmjopen.bmj.com/site/about/guidelines.xhtml

Non-steroidal anti-inflammatory drugs (NSAIDs)

If inflammation in the joint is causing pain and stiffness, a short course of NSAID tablets (for example naproxen) may be useful.

Like all drugs, NSAIDs can sometimes have side-effects, but your doctor will take precautions to reduce the risk of these – for example, by prescribing the lowest effective dose for the shortest possible period of time.

NSAIDs can cause digestive problems (stomach upsets, indigestion, or damage to the stomach lining) so in most cases they'll be prescribed along with a drug called a proton pump inhibitor (PPI), which will help to protect your stomach.

NSAIDs also carry an increased risk of heart attack or stroke. Although the increased risk is small, your doctor will be cautious about prescribing them if there are other factors that may increase your overall risk – for example, smoking, circulation problems, high blood pressure, high cholesterol or diabetes. NSAIDS can also reduce kidney function so you shouldn't take them if you have known reduced kidney function or are on a water tablet (diuretic).

! If you have trouble opening child-proof containers, your pharmacist will put your tablets in a more suitable container for you. Contact us for our special request card which you can hand to your pharmacist with your prescription.

Steroid injections

Steroid injections are sometimes given directly into a particularly painful joint. The injection can start to work within a day or so and may improve pain for several weeks or even months, especially in your knee or thumb. This is mainly used for very painful osteoarthritis, for sudden, painful attacks caused by the shedding of calcium pyrophophate crystals, or to help people through an important event (such as a holiday or family wedding).

i **See Arthritis Research UK drug leaflet** *Local steroid injections.*

Steroid injections can improve pain for several weeks or even months.

For peer review only - http://bmjopen.bmj.com/site/about/guidelines.xhtml

Transcutaneous electrical nerve stimulation (TENS)

Some people find that transcutaneous electrical nerve stimulation (TENS) can help to relieve pain, although research evidence on its effectiveness is mixed. A TENS machine is a small electronic device that sends pulses to your nerve endings via pads placed on your skin. It produces a tingling sensation and is thought to alter pain messages sent to the brain. TENS machines are available from pharmacies and other major stores, but a physiotherapist may be able to loan you one to try before you decide whether to buy one.

Surgery

Surgery may be recommended if your pain is very severe or you have mobility problems. Many thousands of hip and knee replacements are performed each year for osteoarthritis, and other joint replacements are becoming increasingly common. Surgery can be very good for easing pain in cases where other treatments haven't given enough relief.

Sometimes keyhole surgery techniques may be used to wash out loose fragments of bone and other tissue from your knee – this is called arthroscopic lavage and it's not recommended unless your knee locks.

See Arthritis Research UK booklets
Foot and ankle surgery; Hand and wrist surgery; Hip replacement surgery; Knee replacement surgery; Shoulder and elbow joint replacement.

Self-help and daily living

Sleep

If pain is a problem at night, heat may help. Try a hot bath before going to bed, or use a hot-water bottle, wheat bag (which you can heat in a microwave) or electric blanket. Taking a painkiller two hours before going to bed can ease night-time pain so you can get to sleep more easily.

See Arthritis Research UK booklet
Sleep and arthritis.

Work

Most people with osteoarthritis are able to continue in their jobs, although you may need to make some alterations to your working environments, especially if you have a physically demanding job. Speak to your employer's occupational health service if they have one, or your local Jobcentre Plus can put you in touch with Disability Employment Advisors who can arrange work assessments. They can advise you on changing the way you work and on equipment that may help you to do your job more easily. If necessary, they can also help with retraining for more suitable work.

See Arthritis Research UK booklet
Work and arthritis.

Dealing with stress

Living with a long-term condition like osteoarthritis can lower your morale and may affect your sleep. It's important to

For peer review only

tackle problems like these as they could lead to depression and will certainly make the osteoarthritis more difficult to cope with. It often helps to talk about negative feelings, so it could be useful to speak to your healthcare team, or your family and friends. Support groups are also available – your doctor may be able to tell you about organisations in your area.

See Arthritis Research UK booklets and guide *Fatigue and arthritis; Pain and arthritis; Living with long-term pain: a guide to self-management.*

Research and new developments

Research has already shown the importance of exercise and weight management in reducing the pain of osteoarthritis, particularly of the knee. There are many studies going on around the world to find and test new treatments for osteoarthritis. These include studies funded by Arthritis Research UK looking into the benefits of vitamin D (the VIDEO study) and a large national study to find the genes responsible for causing osteoarthritis (the arcOGEN study) which could lead to new therapies. Arthritis Research UK are also funding early trials of stem cell research, which aims to regenerate cartilage using the body's own cells.

Research has already shown the importance of exercise and weight management in reducing the pain of osteoarthritis, particularly of the knee.

For peer review only - http://bmjopen.bmj.com/site/about/guidelines.xhtml

Glossary

Acupuncture – a method of obtaining pain relief which originated in China. Very fine needles are inserted, virtually painlessly, at a number of sites (called meridians) but not necessarily at the painful area. Pain relief is obtained by interfering with pain signals to the brain and by causing the release of natural painkillers (called endorphins).

Aerobic exercise – any exercise that increases your pulse rate and makes you a bit short of breath.

Analgesics – painkillers. As well as dulling pain they lower raised body temperature, and most of them reduce inflammation.

Bunion – a bony lump on the side of the big toe caused by hallux valgus. Sometimes a swelling or bursa on the foot is also called a bunion.

Cartilage – a layer of tough, slippery tissue that covers the ends of the bones in a joint. It acts as a shock absorber and allows smooth movement between bones.

Chiropractor – a trained specialist who treats mechanical disorders of the musculoskeletal system, often through spine manipulation or adjustment. The General Chiropractic Council regulates the practice of chiropractic in the UK.

Collagen – the main substance in the white, fibrous connective tissue that's found in tendons, ligaments and cartilage. This very important protein is also found in skin and bone.

Gout – an inflammatory arthritis caused by a reaction to the formation of urate crystals in the joint. Gout comes and goes in severe flare-ups at first, but if not treated it can eventually lead to joint damage. It often affects the big toe.

Hallux rigidus – osteoarthritis of the big toe joint with a stiff, often painful, big toe.

Hallux valgus – a condition in which the big toe pushes across towards the other toes. It can cause deformities such as bunions and hammer toes.

Hydrotherapy – exercises that take place in water (usually a warm, shallow swimming pool or a special hydrotherapy bath) which can improve mobility, help relieve discomfort and promote recovery from injury.

Inflammation – a normal reaction to injury or infection of living tissues. The flow of blood increases, resulting in heat and redness in the affected tissues, and fluid and cells leak into the tissue, causing swelling.

Ligaments – tough, fibrous bands anchoring the bones on either side of a joint and holding the joint together. In the spine they're attached to the vertebrae and restrict spinal movements, therefore giving stability to the back.

Magnetic resonance imaging (MRI) scan – a type of scan that uses high-frequency radio waves in a strong magnetic field to build up pictures of the inside of the body. It works by detecting water molecules in the body's tissue that give

For peer review only - http://bmjopen.bmj.com/site/about/guidelines.xhtml

out a characteristic signal in the magnetic field. An MRI scan can show up soft-tissue structures as well as bones.

Manipulation – a type of manual therapy used to adjust parts of the body, joints and muscles to treat stiffness and deformity. It's commonly used in physiotherapy, chiropractic, osteopathy and orthopaedics.

Menisci (singular meniscus) – rings of cartilage, like washers, lying between the cartilage-covered bones in the knee. They act as shock absorbers and help the movement of the joint. Each knee has an inside (medial) and an outside (lateral) meniscus.

Menopause – the time when menstruation ends, usually when a woman is in her 50s. This means the ovaries stop releasing eggs every four weeks, and it's no longer possible to have children. If this happens before the age of 45, it's known as premature menopause.

Nodal osteoarthritis – a form of osteoarthritis that often runs in families, characterised by knobbly finger swellings (Heberden's nodes) and a tendency to get osteoarthritis in several joints (especially knees, big toes).

Non-steroidal anti-inflammatory drugs (NSAIDs) – a large family of drugs prescribed for different kinds of arthritis that reduce inflammation and control pain, swelling and stiffness. Common examples include ibuprofen, naproxen and diclofenac.

Occupational therapist – a trained specialist who uses a range of strategies and specialist equipment to help people to reach their goals and maintain their independence by giving practical advice on equipment, adaptations or by changing the way you do things (such as learning to dress using one handed methods following hand surgery).

Osteopath – a trained specialist who treats spinal and other joint problems by manipulating the muscles and joints in order to reduce tension and stiffness, and so help the spine to move more freely. The General Osteopathic Council regulates the practice of osteopathy in the UK.

Osteophytes – an overgrowth of new bone around the edges of osteoarthritic joints. Spurs of new bone can alter the shape of the joint and may press on nearby nerves.

Perthes' disease – inflammation at the head of the thigh bone (femur) that causes pain and limping, usually in boys aged 5–10 years. It can restrict blood supply to the bone leading to poor growth and deformity and can cause osteoarthritis to develop in later life.

Physiotherapist – a trained specialist who helps to keep your joints and muscles moving, helps ease pain and keeps you mobile.

For peer review only - http://bmjopen.bmj.com/site/about/guidelines.xhtml

Proton pump inhibitor (PPI) – a drug that acts on an enzyme in the cells of the stomach to reduce the secretion of gastric acid. They're often prescribed along with non-steroidal anti-inflammatory drugs (NSAIDs) to reduce side-effects from the NSAIDs.

Referred pain – pain that occurs in a different part of the body from that affected by injury or disease (for example, pain in the thigh or knee resulting from osteoarthritis of the hip). This is sometimes called radiated pain.

Rheumatoid arthritis – a common inflammatory disease affecting the joints, particularly the lining of the joint. It most commonly starts in the smaller joints in a symmetrical pattern – that is, for example, in both hands or both wrists at once.

Spondylosis – the term used to describe mechanical or degenerative changes in the small joints in the neck and back. Most of us will have some degeneration in these joints, which can be seen on x-rays, although often these changes don't cause any problems or symptoms.

Synovium – the inner membrane of the joint capsule that produces synovial fluid.

Transcutaneous electrical nerve stimulation (TENS) – a small battery-driven machine which can help to relieve pain. Small pads are applied over the painful area and low-voltage electrical stimulation produces a pleasant tingling sensation, which relieves pain by interfering with pain signals to the brain.

Where can I find out more?

If you've found this information useful you might be interested in these other titles from our range:

Conditions

- *Back pain*
- *Calcium crystal diseases including acute CPP crystal arthritis (pseudogout) and acute calcific tendinitis*
- *Gout*
- *Neck pain*
- *Osteoarthritis of the knee*

Therapies

- *Hydrotherapy and arthritis*
- *Occupational therapy and arthritis*
- *Physiotherapy and arthritis*

Self-help and daily living

- *Complementary and alternative medicine for arthritis*
- *Complementary and alternative medicines for the treatment of rheumatoid arthritis, osteoarthritis and fibromyalgia (63-page special report)*
- *Diet and arthritis*
- *Fatigue and arthritis*
- *Feet, footwear and arthritis*
- *Gardening and arthritis*
- *Keep moving*
- *Living with long-term pain: a guide to self-management*
- *Looking after your joints when you have arthritis*

For peer review only

Arthritis Research UK
Osteoarthritis

- *Pain and arthritis*
- *Practitioner-based complementary and alternative therapies for the treatment of rheumatoid arthritis, osteoarthritis, fibromyalgia, and low back pain (66-page special report)*
- *Sex and arthritis*
- *Sleep and arthritis*
- *What is arthritis?*
- *Work and arthritis*

Surgery

- *Foot and ankle surgery*
- *Hand and wrist surgery*
- *Hip replacement surgery*
- *Knee replacement surgery*
- *Shoulder and elbow joint replacement*

Drug leaflets

- *Painkillers*
- *Local steroid injections*
- *Non-steroidal anti-inflammatory drugs*

You can download all of our booklets and leaflets from our website or order them by contacting:

Arthritis Research UK
Copeman House
St Mary's Court
St Mary's Gate
Chesterfield
Derbyshire S41 7TD
Phone: 0300 790 0400
www.arthritisresearchuk.org

Osteoarthritis guidelines

The National Institute for Health and Clinical Excellence (NICE) issued guidelines to GPs in 2008 on how to best treat osteoarthritis based on available evidence.

The NICE guidance on osteoarthritis is available at www.nice.org.uk/CG59.

Printed copies of the NICE osteoarthritis patient guide can be ordered from 0845 003 7783 or at emailpublications@nice.org.uk quoting reference N1460.

Related organisations

The following organisations may be able to provide additional advice and information:

Arthritis Care
Floor 4, Linen Court
10 East Road
London N1 6AD
Phone: 020 7380 6500
Helpline: 0808 800 4050
Email: info@arthritiscare.org.uk
www.arthritiscare.org.uk

Arthritis and Musculoskeletal Alliance (ARMA)
Bride House
18–20 Bride Lane
London EC4Y 8EE
Phone: 020 7842 0910/11
Email: info@arma.uk.net
arma.uk.net

For peer review only

DIAL Network (formerly Disability Information and Advice Line or Dial UK)
Phone: 01302 310 123
www.scope.org.uk/dial
An independent network of local disability information and advice services run by and for disabled people, part of Scope.

Disabled Living Foundation
380–384 Harrow Road
London W9 2HU
Phone: 020 7289 6111
Helpline: 0845 130 9177
Email: helpline@dlf.org.uk
www.dlf.org.uk

General Chiropractic Council
44 Wicklow Street
London WC1X 9HL
Phone: 020 7713 5155
www.gcc-uk.org

General Osteopathic Council
176 Tower Bridge Road
London SE1 3LU
Phone: 020 7357 6655
www.osteopathy.org.uk

Links to sites and resources provided by third parties are provided for your general information only. We have no control over the contents of those sites or resources and we give no warranty about their accuracy or suitability. You should always consult with you GP or other medical professional.

For peer review only

Notes

For peer review only

For peer review only - http://bmjopen.bmj.com/site/about/guidelines.xhtml

Notes

For peer review only

For peer review only - http://bmjopen.bmj.com/site/about/guidelines.xhtml

Notes

For peer review only

For peer review only - http://bmjopen.bmj.com/site/about/guidelines.xhtml

We're here to help

Arthritis Research UK is the charity leading the fight against arthritis.

We're the UK's fourth largest medical research charity and fund scientific and medical research into all types of arthritis and musculoskeletal conditions.

We're working to take the pain away for sufferers with all forms of arthritis and helping people to remain active. We'll do this by funding high-quality research, providing information and campaigning.

Everything we do is underpinned by research.

We publish over 60 information booklets which help people affected by arthritis to understand more about the condition, its treatment, therapies and how to help themselves.

We also produce a range of separate leaflets on many of the drugs used for arthritis and related conditions. We recommend that you read the relevant leaflet for more detailed information about your medication.

Please also let us know if you'd like to receive our quarterly magazine, *Arthritis Today*, which keeps you up to date with current research and education news, highlighting key projects that we're funding and giving insight into the latest treatment and self-help available.

We often feature case studies and have regular columns for questions and answers, as well as readers' hints and tips for managing arthritis.

Tell us what you think

Please send your views to:
feedback@arthritisresearchuk.org
or write to us at:
Arthritis Research UK, Copeman House, St Mary's Court, St Mary's Gate, Chesterfield, Derbyshire S41 7TQ.

A team of people contributed to this booklet. The original text was written by Prof. Mike Doherty, who has expertise in the subject. It was assessed at draft stage by consultant rheumatology nurse Diana Finney and consultant physiotherapist Kay Stevenson. An **Arthritis Research UK** editor revised the text to make it easy to read, and a non-medical panel, including interested societies, checked it for understanding. An **Arthritis Research UK** medical advisor, Prof. Anisur Rahman, is responsible for the content overall.

For peer review only

Arthritis Research UK

Get involved

You can help to take the pain away from millions of people in the UK by:

- volunteering
- supporting our campaigns
- taking part in a fundraising event
- making a donation
- asking your company to support us
- buying products from our online and high-street shops.

To get more **actively involved,** please call us on **0300 790 0400**, email us at **enquiries@arthritisresearchuk.org** or go to **www.arthritisresearchuk.org**

Arthritis Research UK

Arthritis Research UK
Copeman House
St Mary's Court
St Mary's Gate, Chesterfield
Derbyshire S41 7TD

Tel **0300 790 0400**
calls charged at standard rate

www.arthritisresearchuk.org

Registered Charity No 207711
© Arthritis Research UK 2012
Published November 2012 2025/OA/12-1

For peer review only - http://bmjopen.bmj.com/site/about/guidelines.xhtml

Arthritis Research UK

Keeping active with osteoarthritis

It's important to stay active when you have osteoarthritis – exercising will help ease stiffness and stop your muscles becoming weak. As well as the simple exercises in this pull-out, you should choose a form of exercise you enjoy and stick at it.

Swimming, walking, yoga and Pilates are all great options if you have osteoarthritis.

www.arthritisresearchuk.org

For peer review only - http://bmjopen.bmj.com/site/about/guidelines.xhtml

Exercises for osteoarthritis

This handy tear-off section contains exercises that are designed to stretch, strengthen and stabilise the structures that support your joints.

Exercises for osteoarthritis

The following exercises are designed to stretch, strengthen and stabilise the structures that support your joints.

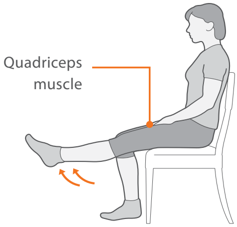

1

Straight-leg raise (sitting): Get into the habit of doing this every time you sit down. Sit well back in the chair with a good posture. Straighten and raise one leg. Hold for a slow count to 10 then slowly lower your leg. Repeat this at least 10 times with each leg. If you can do this easily, try it with light ankle weights and with your toes pointing towards you.

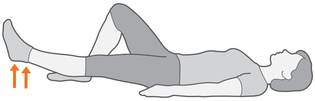

2

Straight-leg raise (lying): Get into the habit of doing this in the morning and at night while lying in bed. Bend one leg at the knee. Hold your other leg straight and lift your foot just off the bed. Hold for a slow count of five then lower. Repeat five times with each leg every morning and evening.

3

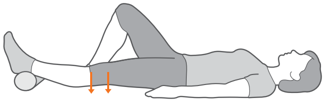

Muscle stretch: Do this at least once a day when lying down. Not only does this exercise help to strengthen the quadriceps muscles, but it also prevents the knee from becoming permanently bent. Place a rolled-up towel under the ankle of the leg to be exercised. Bend the other leg at the knee. Use the muscles of your straight leg to push the back of the knee firmly towards the bed or the floor. Hold for a slow count of five. Repeat at least five times with each leg.

4

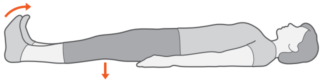

Quad exercise: Pull your toes and ankles towards you, while keeping your leg straight and pushing your knee firmly against the floor. Hold for five seconds and relax. Repeat five times.

For peer review only - http://bmjopen.bmj.com/site/about/guidelines.xhtml

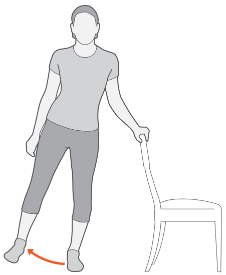

5

Hip abduction: Lift your leg sideways, being careful not to rotate the leg outwards. Hold for five seconds and bring it back slowly, keeping your body straight throughout. Repeat five times on each side. Hold onto a chair or work surface for support.

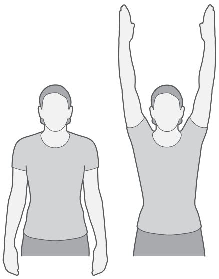

6

Arm stretch (standing): Stand with your arms relaxed at your sides. Raise your arms as far as you can and hold for 5–10 seconds. Lower and repeat five times.

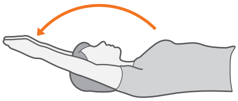

7

Arm stretch (lying): Lie on your back with your arms by your sides. Raise your arms overhead as far as you can and hold for 5–10 seconds. Return your arms to your sides and repeat five times.

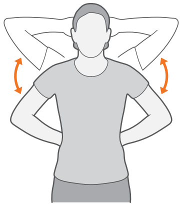

8

Arm lifts: Place your hands behind your head so your elbows are pointing to the sides. Hold for five seconds then place your hands behind your back, keeping your elbows out. Hold for five seconds. Do five sets.

UNIVERSITY OF Southampton

Arthritis Research UK

NHS National Institute for Health Research

Clinical Research Network

Supporting research to make patients, and the NHS, better

Osteoarthritis Thumb Base Therapy Trial

OTTER trial

Thumb Base Pain Exercise Booklet

Information and an exercise programme specifically for people with thumb base pain

OTTER II trial –IRAS 198227

REC ref: 16/SC/0188

For peer review only - http://bmjopen.bmj.com/site/about/guidelines.xhtml

Thumb Base Pain

You have been given this booklet because you have pain or discomfort in your thumb joint.

Pain at the base of your thumb can be due to a number of reasons, one of which can be thumb base Osteoarthritis. Pain at the base of your thumb can affect many aspects of your everyday life. This booklet has been designed with the help of clinicians and patients who have pain at the base of the thumb. It describes a common cause of pain in this area, Osteoarthritis.

This booklet focuses specifically on thumb base pain and provides a programme of exercise devised with the help of national and international therapy clinicians and is supported by published research. You have also been given a Joint Protection Booklet which discusses Osteoarthritis generally, and presents ways to enable you to carry on your normal life as much as possible whilst reducing pain and protecting your joints from further damage.

This booklet contains four main sections:

1. Causes of Thumb Osteoarthritis
2. Symptoms of Thumb Osteoarthritis
3. Treatment of Thumb Osteoarthritis
4. Hand Exercises

For peer review only

For peer review only - http://bmjopen.bmj.com/site/about/guidelines.xhtml

1. Causes of Thumb Osteoarthritis

Osteoarthritis in the carpometacarpal joint (CMC joint) at the base of the thumb is the most common cause of pain in this area.

The CMC joint is formed where the metacarpal bone of the thumb meets the trapezium bone of the wrist.

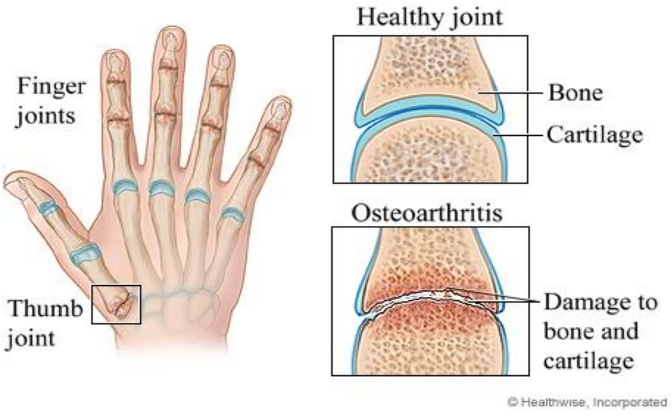

Due to the movement required at the base of the thumb, you rely on your ligaments and bony structures to maintain stability. Damage or overuse can place high loads through the base of the thumb and lead to degeneration. A good point to remember is that any pressures placed through the tip of the thumb during pinching activities are multiplied by around twelve times through the CMC joint.

There are many factors that can lead to pain in the base of the thumb or thumb base Osteoarthritis including a previous injury, repetitive activity of the thumb joint, it can be inherited, and can be affected by gender – women are more likely to have Osteoarthritis than men.

2. Symptoms of Thumb Osteoarthritis

Pain is the primary symptom associated with thumb Osteoarthritis. Initially, pain is present with movement or activity, for example, turning a key, opening a door, lifting a cup. If the Osteoarthritis progresses, pain may be present even during inactivity or rest.

Other symptoms of thumb arthritis include:

- Difficulty gripping objects;
- Swelling, stiffness, or tenderness at the base of the thumb;
- Enlarged appearance and altered posture of the CMC joint;
- Limited range of motion.

For peer review only

For peer review only - http://bmjopen.bmj.com/site/about/guidelines.xhtml

3. Treatment of Thumb Osteoarthritis

Early Osteoarthritis of the thumb can be effectively managed using non-surgical treatment options. These treatments aim to reduce the pain caused by wear and repair of the joint:

- Some medicines can help to reduce inflammation, swelling and pain. You should discuss this with your GP.
- Steroid injections can be given into the joint.
- Self-help measures such as joint protection, diet, complementary therapies etc.

If you have worsening symptoms, you should discuss other options with your GP or consultant.

For peer review only

Additional information can be obtained from the Arthritis Research UK website (http://www.arthritisresearchuk.org/).

We acknowledge the input to this booklet of Sarah Bradley and Kirsty Bancroft of Poole NHS Trust and Prof Ingvild Kjeken from the Norwegian National Advisory Unit on Rehabilitation in Rheumatology.

For peer review only - http://bmjopen.bmj.com/site/about/guidelines.xhtml

4. Hand Exercises

Exercise has many benefits. It can help to ease stiffness, improve movement in your joints and strengthen muscles.

These hand exercises are for you as part of the OTTER II trial. They should benefit you if you have pain at the base of your thumb. These exercises can reduce thumb pain and stiff joints in your hand and strengthen your thumb muscles.

- To achieve the best results **repeat** these exercises at least **3 times a week for at least 20 minutes each time**.
- It is not unusual to experience some slight discomfort or pain in your thumb after doing these exercises. Any discomfort should stop after 24 hours. If you experience discomfort in your thumb or hand when doing these exercises that does not start to feel better after 24 hours, please contact your therapist.
- Always start with the Warm-Up Exercise. To warm up your hand place your hand in a bowl of warm water and gently move your thumb in a circular direction. After one minute you can then change the direction. Carry out these gentle moves for at least 2 minutes. This will help ease your hand and thumb into exercising.
- Follow the Warm-Up Exercise with Level 1 Exercises. When you can easily do Level 1 Exercises then you can go straight from the warm up to Level 2 Exercises. When you can easily do Level 2 Exercises then you can go straight from the warm up to Level 3 Exercises.
- When you do each exercise look carefully at the joints of your thumb. Make sure that your thumb does not bend backwards (or hyperextend) at the joints. Try to make sure that your thumb joints are kept a little bent (or flexed) as you do each exercise, as this will help protect your thumb.

Level 1 Exercises

Exercise 1

Step 1

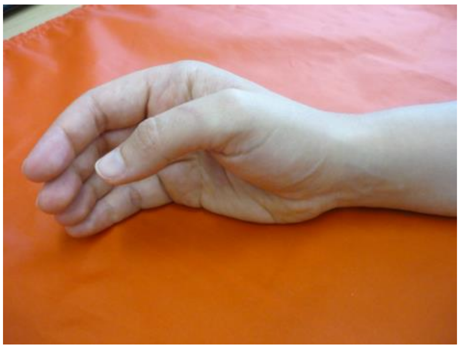

Rest your arm and hand on a table on the little finger side so that your thumb is on top.

For peer review only

Step 2

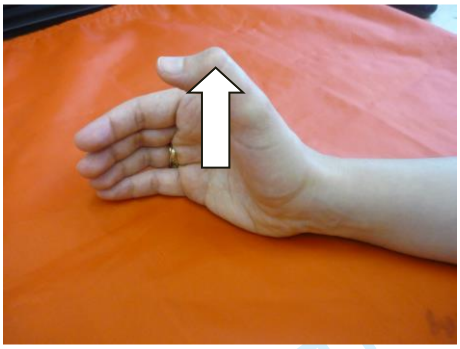

Without help from the other hand lift your thumb upwards as far as possible.

Hold for 10 seconds and repeat up to 10 times.

Make sure that your thumb joints **keep bent** (flexed) when you do this exercise.

Try **not to over extend** any of the thumb joints.

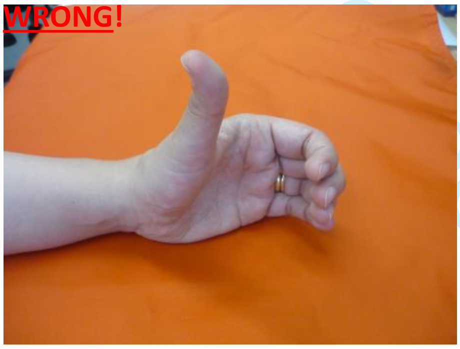

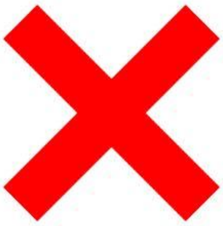

Try **not to over extend** the joints of your thumb. This is **not the right way** to do this exercise.

If your thumb looks like the picture on the left, try the exercise again making sure to **keep your thumb joints bent** (flexed).

Exercise 2

Step 1

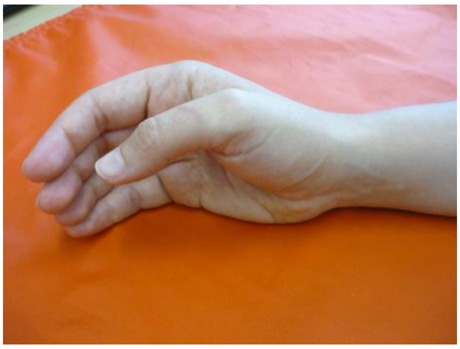

Rest your arm and hand on a table on the little finger side so that your thumb is on top.

Step 2

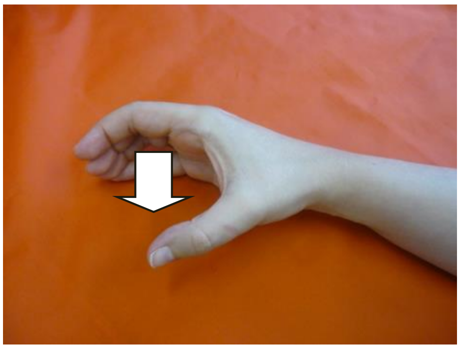

Keeping both joints of the thumb **slightly bent**, keep your thumb as far away from your palm as possible while lifting your thumb up toward the ceiling.

Hold for 10 seconds and repeat up to 10 times.

For peer review only

For peer review only - http://bmjopen.bmj.com/site/about/guidelines.xhtml

WRONG!

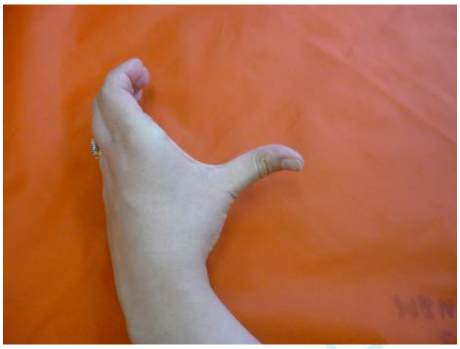

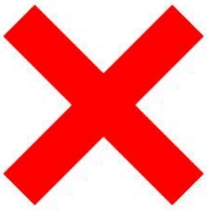

Try **not to bend** the joints of the thumb back.
This is the **wrong way** to do this exercise.

Exercise 3

Place your elbow or back of your hand on a table.
Put your thumb against each fingertip in turn making an ‘O’ shape.
Make sure your thumb joints are **always slightly bent** (flexed).

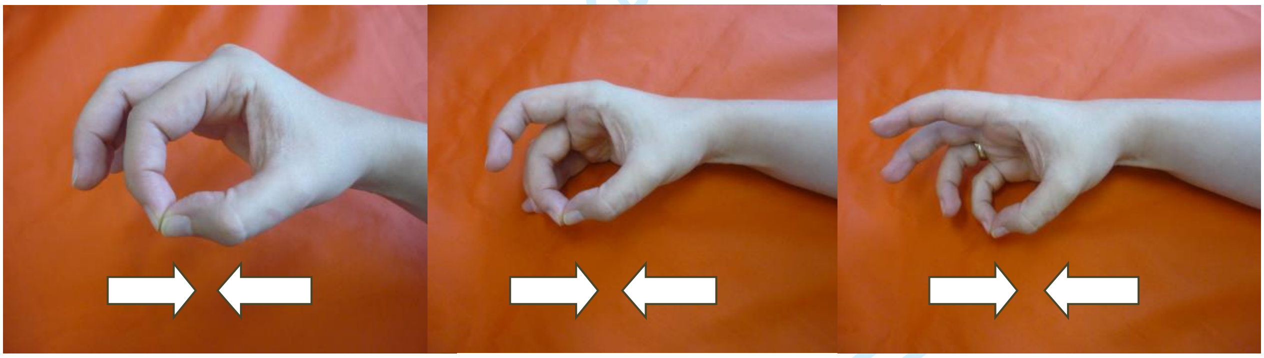

WRONG!

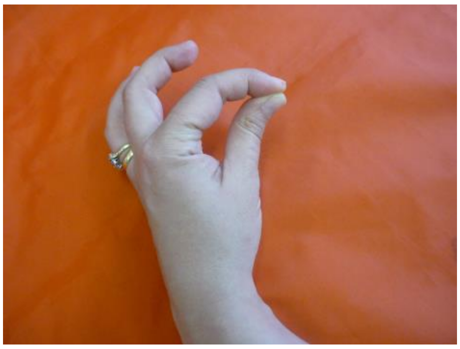

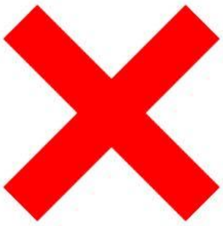

Try **not to bend** the joints of the thumb back.
This is the **wrong way** to do this exercise.

If you can complete Level 1 Exercises easily then move onto Level 2 Exercises. If you cannot complete Level 1 Exercises easily then please do not start Level 2 Exercises

For peer review only - http://bmjopen.bmj.com/site/about/guidelines.xhtml

Level 2 Exercises

Exercise 1

First carry out the **Warm-Up Exercise gently moving your thumb in warm water.**

Perform the rest of the Level 2 Exercises with the strongest rubber band that you can comfortably stretch around your thumb and palm.

Exercise 2

Step 1

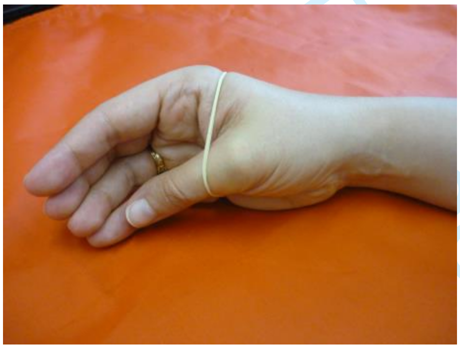

Start with your hand relaxed and a rubber band placed around your hand crossing around the middle of your thumb as in the picture on the left.

Step 2

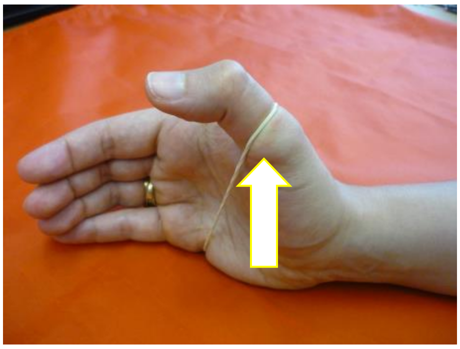

Stretch the band slowly by lifting the thumb upwards as far as possible while keeping the two joints of the thumb slightly bent.

Try to ensure that the wrist **does not bend** forwards during the movement.

Hold for 5 seconds then lower slowly and repeat up to 10 times.

When you can perform this exercise easily, **change** to a stronger band.

For peer review only

WRONG!

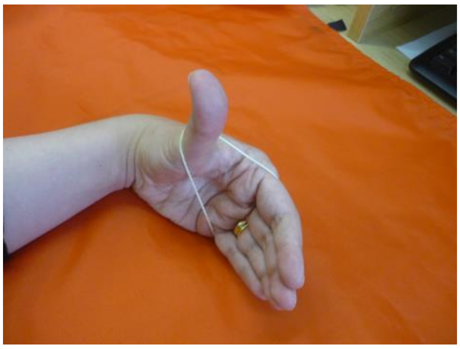

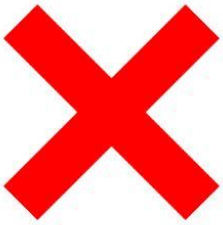

Try **not to bend** the thumb joints back or bring the wrist forward. This is the **wrong way** to do this exercise.

For peer review only - http://bmjopen.bmj.com/site/about/guidelines.xhtml

Exercise 3

Step 1

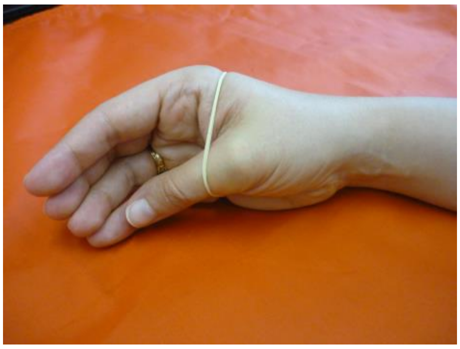

Start with your hand relaxed and a rubber band placed around your hand crossing around the middle of your thumb as in the picture on the left.

Step 2

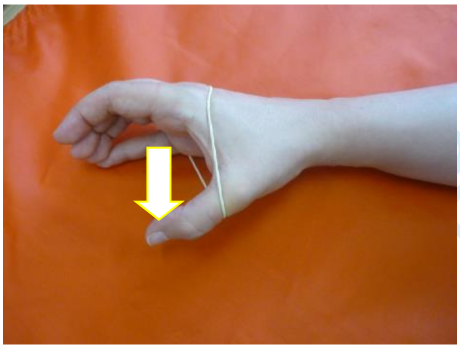

Keep the top **joints of the thumb bent** and lift your thumb up towards your **opposite shoulder** while keeping the thumb as **far away** from the palm as possible.

Step 3

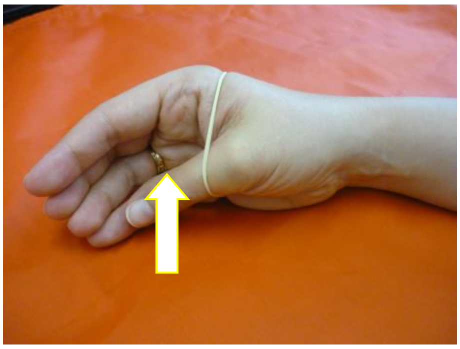

Relax the thumb back to the start position (Step 1) by slowly **lowering the thumb** while keeping both thumb joints a **little bent**.

Try to control this movement and **not let** the band slacken too quickly.

Hold for 5 seconds and repeat up to 10 times.

If you can complete Level 2 Exercises easily then move onto Level 3 Exercises. If you cannot complete these level exercises easily then stop at this level.

For peer review only

Level 3 Exercises

Exercise 1

First carry out the **Warm-Up Exercise**.

Exercise 2: Pinch tasks

Practice tasks where you use your thumb to pinch, for example, writing, holding plates, opening clothes pegs, tearing sheets of paper.

When you do these tasks continue to **keep both joints of the thumb slightly bent**, and the **wrist slightly extended**.

Take time to **look at your thumb** and make an effort to **keep your thumb joints in line**.
Try **not to let them bend backwards**.

The following pictures show the correct and the wrong way to do Pinch task 1.

Pinch Task 1

Practice writing your name and address using the correct thumb position 5 times

CORRECT!

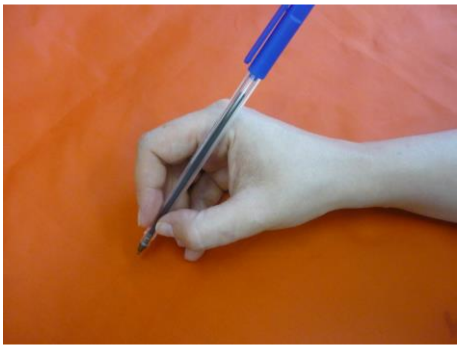

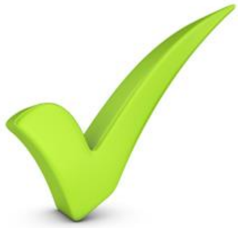

WRONG!

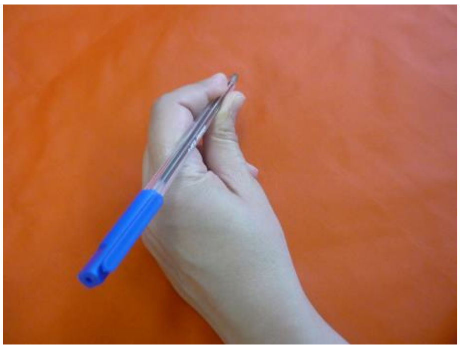

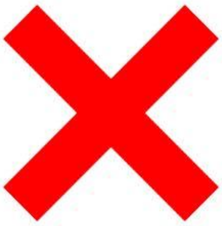

For peer review only

For peer review only - http://bmjopen.bmj.com/site/about/guidelines.xhtml

The following pictures show the correct and the wrong way to do Pinch Task 2

Pinch Task 2

Practice tearing a piece of paper in half using the correct thumb position 20 times

CORRECT!

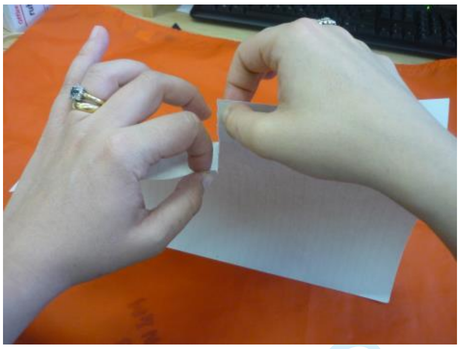

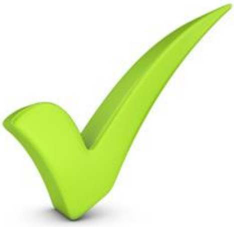

WRONG!

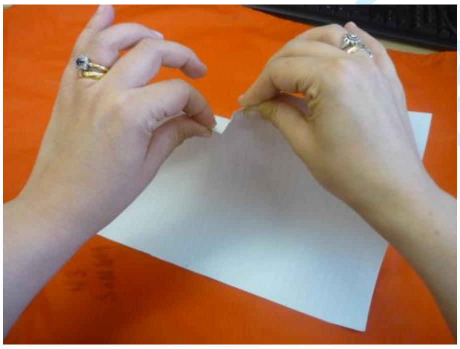

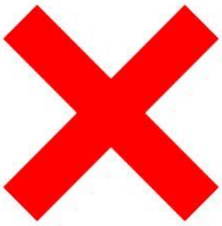

The following pictures show the correct and the wrong way to do Pinch Task 3

Pinch Task 3

Practice holding a clothes peg or a small document clip and squeezing this using the correct thumb position 5 times

CORRECT!

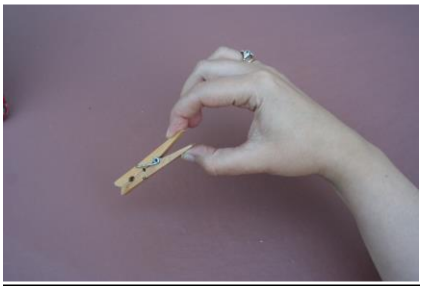

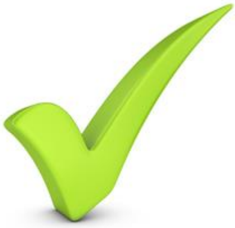

WRONG!

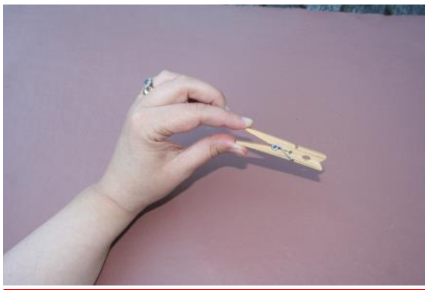

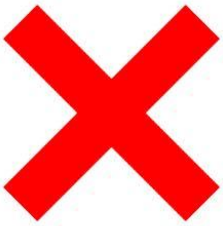

For peer review only

The following pictures show the correct and the wrong way to do Pinch Task 4

Pinch Task 4

Practice picking up and holding a serving plate using the correct thumb position for 5 times

CORRECT!

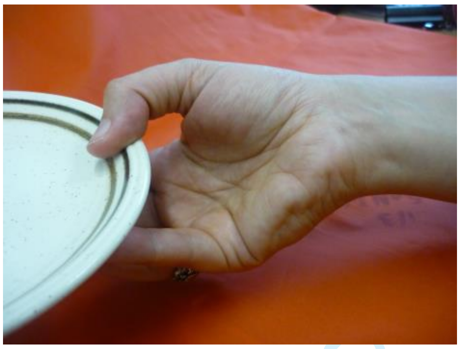

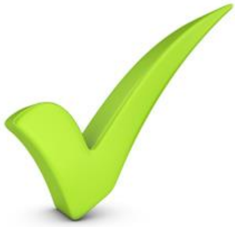

WRONG!

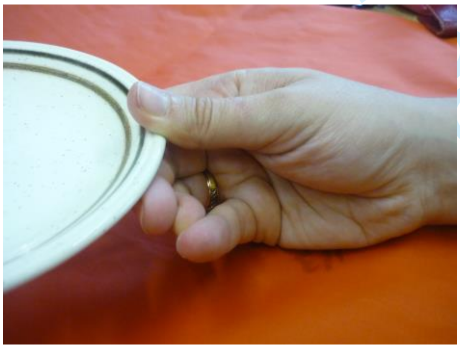

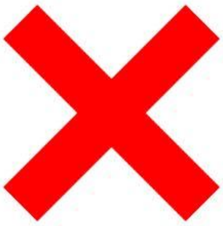

Exercise 3: Grip and turn Tasks

Practice activities which involve turning or twisting, for example, putting nuts on bolts, turning keys in locks, undoing jar tops, turning taps.

During these activities work to maintain a **slight bend (flexion) of both thumb joints** and **avoid the thumb crossing in front of the palm**.

The following pictures show the correct and the wrong way to do Grip and Turn Task 1.

Grip and Turn Task 1

Practice turning a key as if unlocking a door using the correct thumb position 10 times

CORRECT!

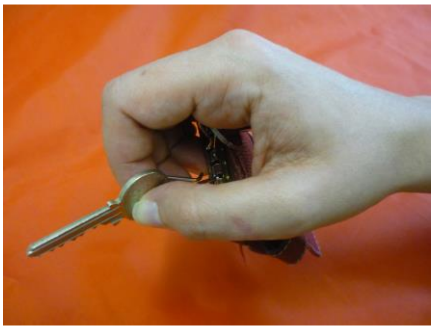

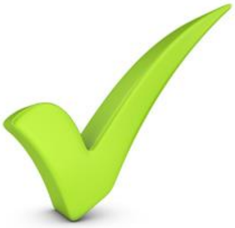

For peer review only - http://bmjopen.bmj.com/site/about/guidelines.xhtml

WRONG!

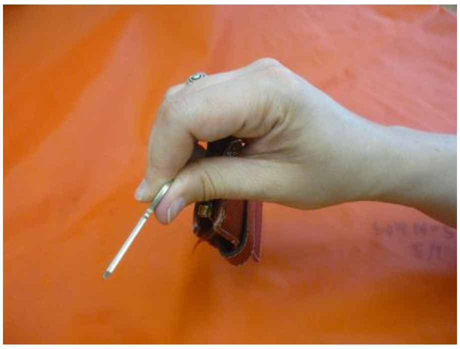

Grip and Turn Task 2

Practice unscrewing and screwing up a bottle top using the correct thumb position five times

CORRECT!

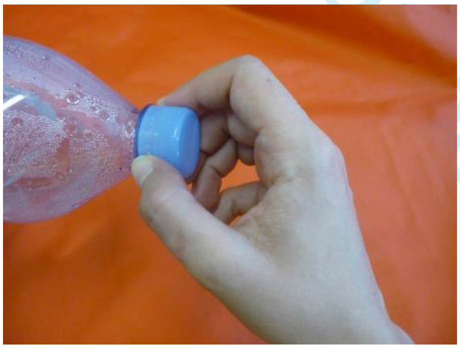

WRONG!

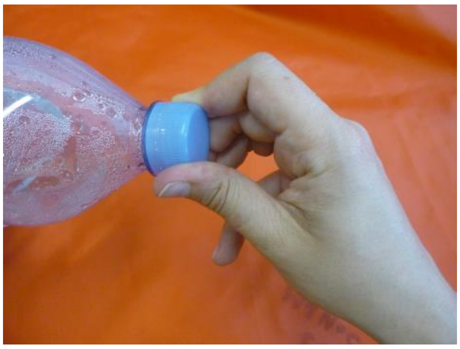

This is the end of the OTTER thumb exercises

If you need to get in touch with the OTTER Trial Research Team, please contact your local occupational therapist or physiotherapist

For peer review only - http://bmjopen.bmj.com/site/about/guidelines.xhtml

STANDARD PROTOCOL ITEMS: RECOMMENDATIONS FOR INTERVENTIONAL TRIALS

The Osteoarthritis Thumb Therapy Trial (OTTER II)

SPIRIT 2013 Checklist: Recommended items to address in a clinical trial protocol and related documents*

Section/item	Item No	Description	Addressed on page number
**Administrative information**			
Title	1	Descriptive title identifying the study design, population, interventions, and, if applicable, trial acronym	1___________
Trial registration	2a	Trial identifier and registry name. If not yet registered, name of intended registry	3___________
	2b	All items from the World Health Organization Trial Registration Data Set	Complete____
Protocol version	3	Date and version identifier	V14.0 dated 23 Aug 2018
Funding	4	Sources and types of financial, material, and other support	23___________
Roles and responsibilities	5a	Names, affiliations, and roles of protocol contributors	1, 22, 23_______
	5b	Name and contact information for the trial sponsor	University of Southampton rgoinfo@soton.ac.uk
	5c	Role of study sponsor and funders, if any, in study design; collection, management, analysis, and interpretation of data; writing of the report; and the decision to submit the report for publication, including whether they will have ultimate authority over any of these activities	None_________

For peer review only

	5d	Composition, roles, and responsibilities of the coordinating centre, steering committee, endpoint adjudication committee, data management team, and other individuals or groups overseeing the trial, if applicable (see Item 21a for data monitoring committee)	Coordinating centre staff (chief investigator, trial manager, trial administrator, qualitative researcher) will oversee the trial in collaboration with the Oxford trials unit (OCTRU), the co-applicants, the sites, the sponsor, oversight committees (DSMC, TSC and TMG) and regulatory bodies.
**Introduction**			
Background and rationale	6a	Description of research question and justification for undertaking the trial, including summary of relevant studies (published and unpublished) examining benefits and harms for each intervention	4,5__________
	6b	Explanation for choice of comparators	4,5___________
Objectives	7	Specific objectives or hypotheses	5____________
Trial design	8	Description of trial design including type of trial (eg, parallel group, crossover, factorial, single group), allocation ratio, and framework (eg, superiority, equivalence, noninferiority, exploratory)	6____________
**Methods: Participants, interventions, and outcomes**			
Study setting	9	Description of study settings (eg, community clinic, academic hospital) and list of countries where data will be collected. Reference to where list of study sites can be obtained	2, 25, 26____

For peer review only - http://bmjopen.bmj.com/site/about/guidelines.xhtml

For peer review only

Eligibility criteria	10	Inclusion and exclusion criteria for participants. If applicable, eligibility criteria for study centres and individuals who will perform the interventions (eg, surgeons, psychotherapists)	6, 7, ___
Interventions	11a	Interventions for each group with sufficient detail to allow replication, including how and when they will be administered	7, 8, 9________
	11b	Criteria for discontinuing or modifying allocated interventions for a given trial participant (eg, drug dose change in response to harms, participant request, or improving/worsening disease)	9 _______
	11c	Strategies to improve adherence to intervention protocols, and any procedures for monitoring adherence (eg, drug tablet return, laboratory tests)	8,9,15, ___
	11d	Relevant concomitant care and interventions that are permitted or prohibited during the trial	9, 10_________
Outcomes	12	Primary, secondary, and other outcomes, including the specific measurement variable (eg, systolic blood pressure), analysis metric (eg, change from baseline, final value, time to event), method of aggregation (eg, median, proportion), and time point for each outcome. Explanation of the clinical relevance of chosen efficacy and harm outcomes is strongly recommended	2, 3, 5, 12, 13, 14 _____________
Participant timeline	13	Time schedule of enrolment, interventions (including any run-ins and washouts), assessments, and visits for participants. A schematic diagram is highly recommended (see Figure)	12, 13________
Sample size	14	Estimated number of participants needed to achieve study objectives and how it was determined, including clinical and statistical assumptions supporting any sample size calculations	14, 15________
Recruitment	15	Strategies for achieving adequate participant enrolment to reach target sample size	9,15 ___________

Methods: Assignment of interventions (for controlled trials)

Allocation:

Sequence generation	16a	Method of generating the allocation sequence (eg, computer-generated random numbers), and list of any factors for stratification. To reduce predictability of a random sequence, details of any planned restriction (eg, blocking) should be provided in a separate document that is unavailable to those who enrol participants or assign interventions	10__________

For peer review only - http://bmjopen.bmj.com/site/about/guidelines.xhtml

Allocation concealment mechanism	16b	Mechanism of implementing the allocation sequence (eg, central telephone; sequentially numbered, opaque, sealed envelopes), describing any steps to conceal the sequence until interventions are assigned	10__________
Implementation	16c	Who will generate the allocation sequence, who will enrol participants, and who will assign participants to interventions	7, 10_________
Blinding (masking)	17a	Who will be blinded after assignment to interventions (eg, trial participants, care providers, outcome assessors, data analysts), and how	6, 9, 10,18_______
	17b	If blinded, circumstances under which unblinding is permissible, and procedure for revealing a participant's allocated intervention during the trial	n/a__________

Methods: Data collection, management, and analysis

Data collection methods	18a	Plans for assessment and collection of outcome, baseline, and other trial data, including any related processes to promote data quality (eg, duplicate measurements, training of assessors) and a description of study instruments (eg, questionnaires, laboratory tests) along with their reliability and validity, if known. Reference to where data collection forms can be found, if not in the protocol	9, 11, 13, 14, 15, 16, 17
	18b	Plans to promote participant retention and complete follow-up, including list of any outcome data to be collected for participants who discontinue or deviate from intervention protocols	16
Data management	19	Plans for data entry, coding, security, and storage, including any related processes to promote data quality (eg, double data entry; range checks for data values). Reference to where details of data management procedures can be found, if not in the protocol	16, 17_________
Statistical methods	20a	Statistical methods for analysing primary and secondary outcomes. Reference to where other details of the statistical analysis plan can be found, if not in the protocol	17, 18, 19, 20
	20b	Methods for any additional analyses (eg, subgroup and adjusted analyses)	18__________
	20c	Definition of analysis population relating to protocol non-adherence (eg, as randomised analysis), and any statistical methods to handle missing data (eg, multiple imputation)	17, 19

Methods: Monitoring

Data monitoring	21a	Composition of data monitoring committee (DMC); summary of its role and reporting structure; statement of whether it is independent from the sponsor and competing interests; and reference to where further details about its charter can be found, if not in the protocol. Alternatively, an explanation of why a DMC is not needed	20, 26. The DSMC in independent from the sponsor and all members will provide competing interest statements.
	21b	Description of any interim analyses and stopping guidelines, including who will have access to these interim results and make the final decision to terminate the trial	16____________
Harms	22	Plans for collecting, assessing, reporting, and managing solicited and spontaneously reported adverse events and other unintended effects of trial interventions or trial conduct	20___________
Auditing	23	Frequency and procedures for auditing trial conduct, if any, and whether the process will be independent from investigators and the sponsor	Subject to audit by national bodies, the trials unit and the sponsor
**Ethics and dissemination**			
Research ethics approval	24	Plans for seeking research ethics committee/institutional review board (REC/IRB) approval	3_____________
Protocol amendments	25	Plans for communicating important protocol modifications (eg, changes to eligibility criteria, outcomes, analyses) to relevant parties (eg, investigators, REC/IRBs, trial participants, trial registries, journals, regulators)	Once approved by sponsor/REC/HRA as appropriate, important modifications will be communicated to site research teams and R&D offices by email.

For peer review only - http://bmjopen.bmj.com/site/about/guidelines.xhtml

Consent or assent	26a	Who will obtain informed consent or assent from potential trial participants or authorised surrogates, and how (see Item 32)	Consent will be obtained by those delegated the task (i.e an NHS therapist trained in OTTER II trial procedures)by the local PI at the site. 11
	26b	Additional consent provisions for collection and use of participant data and biological specimens in ancillary studies, if applicable	11___________
Confidentiality	27	How personal information about potential and enrolled participants will be collected, shared, and maintained in order to protect confidentiality before, during, and after the trial	16, 17________
Declaration of interests	28	Financial and other competing interests for principal investigators for the overall trial and each study site	23___________
Access to data	29	Statement of who will have access to the final trial dataset, and disclosure of contractual agreements that limit such access for investigators	17____________
Ancillary and post-trial care	30	Provisions, if any, for ancillary and post-trial care, and for compensation to those who suffer harm from trial participation	11___________
Dissemination policy	31a	Plans for investigators and sponsor to communicate trial results to participants, healthcare professionals, the public, and other relevant groups (eg, via publication, reporting in results databases, or other data sharing arrangements), including any publication restrictions	21___________
	31b	Authorship eligibility guidelines and any intended use of professional writers	Described in trial Publication Plan
	31c	Plans, if any, for granting public access to the full protocol, participant-level dataset, and statistical code	n/a____________ _

Appendices

For peer review only - http://bmjopen.bmj.com/site/about/guidelines.xhtml

Informed consent materials	32	Model consent form and other related documentation given to participants and authorised surrogates	Not included in this submission – available on request ______________
Biological specimens	33	Plans for collection, laboratory evaluation, and storage of biological specimens for genetic or molecular analysis in the current trial and for future use in ancillary studies, if applicable	n/a_____________ _

*It is strongly recommended that this checklist be read in conjunction with the SPIRIT 2013 Explanation & Elaboration for important clarification on the items. Amendments to the protocol should be tracked and dated. The SPIRIT checklist is copyrighted by the SPIRIT Group under the Creative Commons "Attribution-NonCommercial-NoDerivs 3.0 Unported" license.

For peer review only
